# Clinical Management of Wasp Stings Using Large Language Models: Cross-Sectional Evaluation Study

**DOI:** 10.2196/67489

**Published:** 2025-06-04

**Authors:** Wei Pan, Shuman Zhang, Yonghong Wang, Zhenglin Quan, Yanxia Zhu, Zhicheng Fang, Xianyi Yang

**Affiliations:** 1 Department of Emergency Medicine Taihe Hospital Hubei University of Medicine Shiyan, Hubei China; 2 The Intensive Care Unit The First Dongguan Affiliated Hospital Guangdong Medical University Dongguan, Guangdong China; 3 Cardiopulmonary Rehabilitation Center Taihe Hospital Hubei University of Medicine Shiyan, Hubei China

**Keywords:** artificial intelligence, decision support, emergency medicine, hymenoptera envenomation, natural language processing

## Abstract

**Background:**

Wasp stings are a significant public health concern in many parts of the world, particularly in tropical and subtropical regions. The venom of wasps contains a variety of bioactive compounds that can lead to a wide range of clinical effects, from mild localized pain and swelling to severe, life-threatening allergic reactions, such as anaphylaxis. With the rapid development of artificial intelligence (AI) technologies, large language models (LLMs) are increasingly being used in health care, including emergency medicine and toxicology. These models have the potential to assist health care professionals in making fast and informed clinical decisions. This study aimed to assess the performance of 4 leading LLMs—ERNIE Bot 3.5 (Baidu), ERNIE Bot 4.0 (Baidu), Claude Pro (Anthropic), and ChatGPT 4.0—in managing wasp sting cases, with a focus on their accuracy, comprehensiveness, and decision-making abilities.

**Objective:**

The objective of this research was to systematically evaluate and compare the capabilities of the 4 LLMs in the context of wasp sting management. This involved analyzing their responses to a series of standardized questions and real-world clinical scenarios. The study aimed to determine which LLMs provided the most accurate, complete, and clinically relevant information for the management of wasp stings.

**Methods:**

This study used a cross-sectional design, creating 50 standardized questions that covered 10 key domains in the management of wasp stings, along with 20 real-world clinical case scenarios. Responses from the 4 LLMs were independently evaluated by 8 domain experts, who rated them on a 5-point Likert scale based on accuracy, completeness, and usefulness in clinical decision-making. Statistical comparisons between the models were made using the Wilcoxon signed-rank test, and the consistency of expert ratings was assessed using the Kendall coefficient of concordance.

**Results:**

Claude Pro achieved the highest average score of 4.7 (SD 0.603) out of 5, followed closely by ChatGPT 4.0 with a score of 4.5. ERNIE Bot 4.0 and ERNIE Bot 3.5 received average scores of 4 (SD 0.600) and 3.8, respectively. In analyzing the 20 complex clinical cases, Claude Pro significantly outperformed ERNIE Bot 3.5, particularly in areas such as managing complications and assessing the severity of reactions (*P*<.001). The expert ratings showed moderate agreement (Kendall W=0.67), indicating that the assessments were consistently reliable.

**Conclusions:**

The results of this study suggest that Claude Pro and ChatGPT 4.0 are highly capable of providing accurate and comprehensive support for the clinical management of wasp stings, particularly in complex decision-making scenarios. These findings support the increasing role of AI in emergency and toxicological medicine and suggest that the choice of AI tool should be based on the specific needs of the clinical situation, ensuring that the most appropriate model is selected for different health care applications.

## Introduction

Hymenoptera insects, particularly bees and wasps, are common culprits of venomous stings. Approximately 50% of the population will experience at least one such sting in their lifetime [1]. These stings release various small-molecule compounds that act as allergens and toxins, potentially causing a spectrum of reactions ranging from localized swelling to severe anaphylaxis and in extreme cases, hemolysis or rhabdomyolysis leading to multiorgan failure [1-3]. Currently, there is no specific treatment available for wasp stings. In recent years, the global incidence of wasp stings has been increasing, accompanied by a significant rise in mortality rates [4,5]. For severe cases, recent studies from China, specifically from Sichuan Province, have reported that the mortality rate can reach 37% for patients with more than 30 stings and up to 75% for those with over 100 stings. These findings significantly exceed the previously reported 5.6% mortality rate observed in general cases in Hubei Province [6,7]. Although wasp stings are often viewed as a regional issue, their clinical significance extends beyond geographical boundaries. In Asian countries, particularly China, wasp sting mortality rates significantly exceed those reported in Western nations, where prompt medical intervention is crucial to reducing complications and mortality.

The clinical manifestations following wasp stings vary significantly across regions due to differences in wasp species, resulting in diverse research focuses and the absence of a unified global guideline for diagnosis and treatment. In Southeast Asia [8], mainland China, and Taiwan Province of China [9], wasp stings are the most prevalent. Chinese research primarily focuses on venom composition analysis [10], toxicological mechanisms [11], and improving clinical management strategies [12]. For instance, Quan et al [13] found that early lipid level reduction in patients with wasp stings is associated with clinical severity, suggesting that targeting lipid metabolism could be a novel therapeutic approach. China has established an expert consensus on first aid and treatment for wasp stings tailored to national conditions and has conducted extensive public education and protection training in high-incidence areas [14]. In contrast, in Western countries, honeybee stings are predominant and are one of the most common triggers of allergic reactions [15]. Research in these regions mainly focuses on immunological response mechanisms, epidemiological characteristics [16], and allergic reactions and their immunotherapy [17], following guidelines related to Hymenoptera injuries and allergic reactions [18].

In recent years, artificial intelligence (AI) technology has demonstrated tremendous potential in the health care sector. With advancements in natural language processing, large language models (LLMs) have gained significant attention for their role in assisting clinical decision-making. LLMs such as ChatGPT have been noted for their exceptional performance in processing and generating medical information [19,20]. In emergency medicine, LLMs have been used to support triage decisions and generate discharge summaries [21]. ChatGPT has demonstrated strong accuracy in handling emergency triage and complex medical decision-making [22]. In addition, the multimodal applications of LLMs in health care management have shown unprecedented diversity [23]. In toxicology, LLMs have shown rapid and accurate clinical decision-making capabilities in common organophosphate poisoning cases [24]. However, research on the application of LLMs in the specific area of wasp sting management remains limited.

Although previous studies have evaluated ChatGPT’s performance in terms of medical knowledge and clinical decision support [25], there is a paucity of research on the application of the Chinese ERNIE Bot series and the emerging U.S.-based Claude Pro model in the field of wasp sting injuries. Given that these models are primarily trained on vast amounts of internet data, their accuracy and reliability in addressing wasp sting–related issues require further validation. Moreover, existing research predominantly focuses on general medical knowledge assessment, with insufficient in-depth exploration of specific disease areas, especially acute conditions like wasp sting injuries. Currently, there is a notable gap in LLMs’ clinical decision models specifically for wasp sting management, particularly in areas such as complication prediction, severity assessment, and management of special populations, limiting their practical value in frontline emergency care.

This study aims to address a gap in current research by systematically evaluating and comparing the performance of ERNIE Bot 3.5 and 4.0, Claude Pro, and ChatGPT 4.0 in standardized scenarios of simulated wasp stings and specific clinical contexts. We seek to explore the potential applicability and limitations of these models in clinical toxicology. While these LLMs may excel in certain management tasks, they could be inadequate when dealing with complex or rare cases. The findings of this research will provide a basis for future AI model optimization and contribute to the development of clinical management guidelines for wasp stings.

## Methods

### Study Design and Ethical Considerations

We used a cross-sectional design to systematically evaluate the performance of 4 AI LLMs in the clinical management of wasp stings. As this study did not involve specific patient information or human experimentation, approval from an institutional review board was not required.

#### Expert Panel and Question Development

We assembled a multidisciplinary panel of 8 experts in the field of wasp sting injuries. Of 8 experts, 4 were involved in formulating the “Chinese Expert Consensus on the Standardized Diagnosis and Treatment of Wasp Stings,” while the other 4 were colleagues with extensive clinical experience and research expertise in managing wasp stings. This expert panel was responsible for designing and scoring the evaluation questions.

The question development process involved the following steps:

1. A comprehensive review and analysis of current expert consensus documents and relevant literature on wasp sting management, both domestically and internationally.
2. Extraction of key management steps and critical knowledge points.
3. Development of 50 standardized questions in collaboration with clinical experts, covering 10 key domains: foundational knowledge, early management, anaphylaxis management, complication management, severity assessment, special population management, pharmacological treatment, wound care, long-term follow-up, and public health and prevention.

#### AI Model Evaluation

We evaluated 4 large AI language models: ERNIE Bot 3.5, ERNIE Bot 4.0, Claude Pro, and ChatGPT 4.0. Our study design ensured a rigorous and unbiased assessment: on August 21, 2024, the principal investigator input identical questions into all 4 models, carefully documenting their responses. This approach guaranteed consistent testing conditions and comparable results. We first compared models within their respective families using 50 standardized questions: ERNIE Bot 3.5 versus 4.0, and Claude Pro versus ChatGPT 4.0. The top performer from each pair then underwent further evaluation. In the final phase, we challenged the selected ERNIE Bot 3.5 model and Claude Pro with 20 complex clinical scenarios. This allowed us to assess their capabilities in handling sophisticated medical queries. Throughout the study, we prioritized scientific integrity and methodological precision. By maintaining strict control over input parameters, we ensured highly reliable and comparable findings. This methodology enabled us to objectively evaluate these advanced AI models’ proficiency in addressing diverse linguistic and clinical challenges.

#### Scoring Criteria

The 8 experts independently rated the accuracy and completeness of each model’s responses. To mitigate potential bias, we used an anonymous review process. The ratings were based on a 5-point Likert scale, where:

1. Completely inaccurate or incomplete
2. Mostly inaccurate or incomplete
3. Moderately accurate or complete
4. Mostly accurate or complete
5. Fully accurate or complete

#### Data Analysis

The average, median, and range of the accuracy and integrity scores of each AI model are calculated to establish an overall performance overview. The hierarchical scores of different problem categories are summarized in order to deeply analyze the performance differences. The Wilcoxon signed-rank test is used to compare the performance of the ERNIE Bot series with Claude Pro and ChatGPT 4.0 in terms of accuracy and integrity, to identify the best-performing model in each pair comparison and conduct further comparative analysis. In order to ensure the reliability of scoring, the Kendall consistency coefficient is used to evaluate the consistency of expert scoring. In addition, the double-sample binomial proportional test is used to compare the differences in specific performance of the 3 AI models and significant changes in specific abilities are revealed. All statistical analyses are carried out using the SPSS (version 26.0; IBM Corp) and the *P* value <.05 is considered statistically significant.

### Ethical Considerations

This study was granted exemption from full ethical review by the Ethics Committee of Taihe Hospital, Hubei University of Medicine (reference number 2025MS43).

## Results

### Overview

This study systematically evaluated the performance of 4 LLMs (ERNIE Bot 3.5, ERNIE Bot 4.0, Claude Pro, and ChatGPT 4.0) in the clinical management of wasp sting injuries. We assessed the accuracy and comprehensiveness of their responses across various categories. The results are as follows.

### Overall Performance

Claude Pro demonstrated superior performance in terms of both accuracy and completeness, significantly outperforming other models (*P*<.001). ChatGPT 4.0 ranked second, with particularly strong results in foundational knowledge and early management. ERNIE Bot 3.5 exhibited a relatively balanced performance across categories such as foundational knowledge and allergy management but was inferior to Claude Pro and ChatGPT 4.0 overall. ERNIE Bot 4.0 showed comparatively weaker performance, especially in severity assessment and allergy management, scoring significantly lower than ERNIE Bot 3.5.

### Performance in Specific Categories

The models showed significant differences in their performance across various clinical management categories. The main results are summarized as follows:

1. Basic knowledge: ERNIE Bot 3.5 and Claude Pro performed similarly in terms of accuracy and completeness, both demonstrating strong performance. ERNIE Bot 4.0 had the lowest scores in this category.
2. Early intervention: Claude Pro achieved the highest scores, particularly excelling in accuracy compared to other models (*P*<.05).
3. Allergy management: Claude Pro stood out in this category, significantly outperforming other models, while ERNIE Bot 4.0 showed the weakest performance (*P*<.05).
4. Complication management: Claude Pro led again in managing complex complications, demonstrating its advantage in handling intricate clinical decision-making scenarios (*P*<.05)
5. Severity assessment: Claude Pro showed the best performance in both accuracy and completeness, followed by ERNIE Bot 3.5, with ERNIE Bot 4.0 showing the weakest performance (*P*<.05).
6. Management of special populations: Both Claude Pro and ChatGPT 4.0 performed well in this category, while ERNIE Bot 3.5 had lower scores.
7. Pharmacotherapy: Claude Pro significantly outperformed other models in pharmacotherapy, particularly in accuracy (*P*<.05).
8. Wound care: Claude Pro and ChatGPT 4.0 outperformed the ERNIE Bot series, with a significant difference in scores (*P*<.05).
9. Long-term follow-up: Claude Pro scored significantly higher in both accuracy and completeness than the other models.
10. Public health and prevention: Claude Pro and ERNIE Bot 3.5 performed better in this category, while ERNIE Bot 4.0 and ChatGPT 4.0 were relatively weaker.

For detailed performance in each category, refer to Tables 1 and 2. The responses of each model to the 50 standardized questions can be found in Multimedia Appendices 1-4. Detailed information about Tables 1 and 2 can be found in Multimedia Appendix 5.

**Table 1:** Scores for accuracy for each engine in each category.

Category		ERNIE Bot 3.5 (n=8)	ERNIE Bot 4.0 (n=8)	*P* value	Claude Pro (n=8)	ChatGPT4.0 (n=8)	*P* value	ERNIE Bot 3.5 (n=8)	Claude Pro (n=8)	*P* value
**Basic knowledge**										
	Mean (SD)	21.625 (1.923)	20.0 (1.195)	0.04	20.875 (3.182)	19.125 (1.356)	0.08	21.625 (1.923)	20.875 (3.182)	0.02
	Median (min-max)	21.5 (19-24)	19.5 (19-22)	—^a^	20.5 (15-25)	19.5 (17-21)	—	21.5 (19-24)	20.5 (15-25)	—
**Early management**										
	Mean (SD)	17.875 (1.808)	16.875 (1.642)	0.12	21.75 (3.495)	19.25 (2.55)	0.02	17.875 (1.808)	21.75 (3.495)	0.07
	Median (min-max)	18 (15-20)	17 (15-20)	—	22 (15-25)	20 (14-23)	—	18 (15-20)	22 (15-20)	—
**Allergy management**										
	Mean (SD)	19 (1.69)	17.875 (1.959)	0.13	23.25 (1.832)	19.25 (2.375)	0.01	19 (1.69)	23.25 (1.832)	0.73
	Median (min-max)	18.5 (17-22)	18 (14-21)	—	24 (20-25)	18.5 (17-24)	—	18.5 (17-22)	24 (20-25)	—
**Complication management**										
	Mean (SD)	18.875 (1.458)	18.125 (1.642)	0.16	24 (1.927)	18.375 (1.598)	0.01	18.875 (1.458)	24 (1.927)	0.34
	Median (min-max)	19 (17-21)	18.5 (16-20)	—	25 (20-25)	19 (16-20)	—	19 (17-21)	25 (20-25)	—
**Severity assessment**										
	Mean (SD)	19 (1.512)	16.75 (1.832)	0.02	23.75 (1.753)	20.375 (1.598)	0.02	19 (1.512)	23.75 (1.753)	0.03
	Median (min-max)	18 (18-22)	16 (15-20)	—	24.5 (20-25)	20 (19-24)	—	18 (18-22)	24.5 (20-25)	—
**Special population management**										
	Mean (SD)	17.875 (1.458)	16.75 (1.909)	0.08	22.875 (2.357)	20 (1.927)	0.03	17.875 (1.458)	22.875 (2.357)	0.02
	Median (min-max)	18 (16-20)	16.5 (14-20)	—	23.5 (20-25)	20 (17-24)	—	18 (16-20)	23.5 (20-25)	—
**Pharmacological treatment**										
	Mean（SD）	18.625 (1.768)	17.375 (2.134)	0.11	22.875 (2.295)	20.5 (1.927)	0.06	18.625 (1.768)	22.875 (2.295)	0.04
	Median (min-max)	19 (15-20)	18 (15-20)	—	23 (19-25)	20 (19-25)	—	19 (15-20)	23 (19-25)	—
**Wound management**										
	Mean (SD)	17.625 (2.134)	16.5 (2.878)	0.07	22.375 (1.923)	20.125 (1.808)	0.02	17.625 (2.134)	22.375 (1.923)	0.03
	Median (min-max)	17 (15-21)	15.5 (12-20)	—	22.5 (20-25)	20 (18-23)	—	17 (15-21)	22.5 (20-25)	—
**Long-term follow-up**										
	Mean (SD)	19.875 (2.357)	18.5 (2.07)	0.08	23.125 (1.642)	20.625 (1.408)	0.02	19.875 (2.357)	23.125 (1.642)	0.46
	Median (min-max)	20 (15-23)	19 (16-22)	—	23 (20-25)	20 (20-24)	—	20 (15-23)	23 (20-25)	—
**Public health and prevention**										
	Mean (SD)	19.75 (2.188)	17.875 (2.532)	0.03	24 (1.69)	20 (2.204)	0.02	19.75 (2.188)	24 (1.69)	0.89
	Median (min-max)	20 (15-23)	18 (15-22)	—	24.5 (20-25)	20 (18-25)	—	20 (15-23)	24.5 (20-25)	—

^a^Not available.

**Table 2:** Scores for completeness for each engine in each category.

Category		ERNIE Bot 3.5 (n=8)	ERNIE Bot 4.0 (n=8)	*P* value	Claude Pro (n=8)	ChatGPT4.0 (n=8)	*P* value	ERNIE Bot 3.5 (n=8)	Claude Pro (n=8)	*P* value
**Basic knowledge**										
	Mean (SD)	21.875 (2.642)	19 (1.414)	0.01	21.125 (2.696)	18.625 (1.847)	0.02	21.875 (2.642)	21.125 (2.696)	0.01
	Median (min-max)	22 (17-25)	19 (16-21)	—^a^	21 (18-25)	19 (16-21)	—	22 (17-25)	21 (18-25)	—
**Early management**										
	Mean (SD)	18.75 (1.165)	16.375 (1.302)	0.03	22.625 (2.774)	19 (1.927)	0.01	18.75 (1.165)	22.625 (2.774)	0.92
	Median (min-max)	18.5 (17-20)	17 (14-18)	—	23.5 (18-25)	19.5 (16-22)	—	18.5 (17-20)	23.5 (18-25)	—
**Allergy management**										
	Mean (SD)	19.125 (2.167)	17.75 (2.053)	0.02	23.5 (1.604)	19.125 (2.532)	0.01	19.125 (2.167)	23.5 (1.604)	1
	Median (min-max)	19.5 (15-22)	18 (15-21)	—	24 (20-25)	18.5 (16-24)	—	19.5 (15-22)	24 (20-25)	—
**Complication management**										
	Mean (SD)	18.875 (1.458)	17.5 (2)	0.09	24.125 (1.808)	17.5 (1.927)	0.01	18.875 (1.458)	24.125 (1.808)	0.11
	Median (min-max)	19 (17-21)	17 (15-20)	—	25 (20-25)	18 (15-20)	—	19 (17-21)	25 (20-25)	—
**Severity assessment**										
	Mean (SD)	18.75 (1.753)	16.5 (2)	0.02	24. (1.69)	20.125 (1.959)	0.01	18.75 (1.753)	24 (1.69)	0.03
	Median (min-max)	18.5 (16-22)	15.5 (15-20)	—	24.5 (20-25)	20 (16-23)	—	18.5 (16-22)	24.5 (20-25)	—
**Special population management**										
	Mean (SD)	17.75 (1.669)	16.625 (1.847)	0.07	23.625 (2.066)	19.625 (2.669)	0.01	17.75 (1.669)	23.625 (2.066)	0.03
	Median (min-max)	18 (15-20)	16 (15-20)	—	25 (20-25)	20 (15-24)	—	18 (15-20)	25 (20-25)	—
**Pharmacological treatment**										
	Mean (SD)	17.625 (2.326)	17 (2.268)	0.32	23.875 (1.808)	20.375 (2.2)	0.02	17.625 (2.326)	23.875 (1.808)	0.03
	Median (min-max)	18 (15-20)	16.5 (15-20)	—	25 (20-25)	20 (17-25)	—	18 (15-20)	25 (20-25)	—
**Wound management**										
	Mean (SD)	17.625 (2.264)	16.625 (2.264)	0.07	22.5 (2.33)	19.625 (1.768)	0.02	17.625 (2.264)	22.5 (2.33)	0.04
	Median (min-Max)	17.5 (15-21)	15 (15-20)	—	23.5 (18-25)	20 (16-22)	—	17.5 (15-21)	23.5 (18-25)	—
**Long-term follow-up**										
	Mean (SD)	19.625 (2.446)	17.5 (2.563)	0.04	23.25 (2.435)	21 (1.852)	0.018	19.625 (2.446)	23.25 (2.435)	0.13
	Median (min-max)	20 (15-23)	16.5 (15-22)	—	24 (18-25)	21 (18-24)	—	20 (15-23)	24 (18-25)	—
**Public health and prevention**										
	Mean (SD)	19.5 (2.138)	16.875 (2.696)	0.02	24.25 (1.753)	19.25 (2.964)	0.02	19.5 (2.138)	24.25 (1.753)	0.61
	Median (min-max)	20 (16-23)	15.5 (15-22)	—	25 (20-25)	19 (15-25)	—	20 (16-23)	25 (20-25)	—

^a^Not available.

### Score Distribution

#### Highest Score (5 Points)

Claude Pro received the highest score of 5 in 539 out of 800 ratings (67.4%), significantly outperforming other models (*P*<.001). ChatGPT 4.0 received the highest score in 111 out of 800 ratings (13.9%).

#### Lowest Score (1 Point)

ERNIE Bot 3.5 and ERNIE Bot 4.0 received the lowest score of 1 in 3 out of 800 responses (0.4%) and 2 out of 800 responses (0.3%) respectively. Neither Claude Pro nor ChatGPT 4.0 received the lowest score.

### Performance in Specific Clinical Scenarios

In the evaluation of 20 specific clinical scenarios, Claude Pro demonstrated exceptional performance in both accuracy and comprehensiveness:

1. Accuracy: Claude Pro achieved a mean score of 4.787 (median 5), significantly outperforming ERNIE Bot 3.5, which scored a mean of 3.5 (*P*<.001).
2. Comprehensiveness: Claude Pro’s performance was particularly strong in complex scenarios, demonstrating robust decision-support capabilities in wound management and complication handling. ERNIE Bot 3.5 showed balanced performance across specific clinical scenarios but was slightly inferior to Claude Pro. ChatGPT 4.0 exhibited relatively weaker performance in handling complications and managing special populations.

### Expert Scoring Consistency

The consistency of expert scoring was evaluated using the Kendall coefficient of concordance. The results showed that ERNIE Bot 3.5 and Claude Pro had a higher degree of consistency in their scores, indicating that they received relatively consistent expert feedback on most issues. In contrast, ERNIE Bot 4.0 and ChatGPT 4.0 exhibited lower consistency, reflecting significant variations in expert opinions in certain scenarios.

In summary, Claude Pro demonstrated significantly superior performance in managing wasp sting cases compared to other models, particularly excelling in complex decision-making and complication management with high accuracy and comprehensiveness. Although ERNIE Bot 3.5 showed strong performance in certain categories, it overall lagged behind Claude Pro. ChatGPT 4.0 performed well in basic knowledge and early-stage management but fell short in handling complex scenarios. ERNIE Bot 4.0 had the weakest overall performance among the models evaluated.

## Discussion

### Principal Findings

As senior clinical researchers, we present the first systematic evaluation of 4 mainstream LLMs in the clinical management of wasp stings. Our study comprehensively assessed accuracy, completeness, and performance in specific clinical scenarios. The findings show that LLMs can provide accurate clinical decision-making responses, particularly in toxicology and emergency medicine. LLMs produced diagnostic and treatment suggestions that were consistent with expert ratings in simulated clinical scenarios. This demonstrates the potential of LLMs to assist decision-making in emergencies where quick, reliable responses are essential. Despite some limitations, such as occasional inaccuracies in complex cases, the models’ performance in common clinical situations suggests they could support real-time medical decisions.

### Overall Model Performance

Claude Pro demonstrated superior performance in both accuracy and completeness, particularly excelling in complex decision-support tasks such as complication management and severity assessment. It significantly outperformed other models in these areas, suggesting that Claude Pro can effectively handle complex issues requiring deep medical knowledge and multistep reasoning. This superior performance likely stems from its larger training dataset and advanced natural language processing capabilities. ChatGPT 4.0 performed well in most cases, scoring high in categories such as foundational knowledge and early-stage intervention. However, it fell slightly behind Claude Pro when handling more complex scenarios. In contrast, ERNIE Bot 3.5 showed a more balanced performance, especially in Chinese medical environments, making it a suitable free large model for regions in China where wasp sting incidents are prevalent. Surprisingly, ERNIE Bot 4.0 did not demonstrate significant improvements as an upgraded version, particularly struggling with complex decision-making. This unexpected result may be related to its training data or algorithmic updates.

### Performance Analysis by Category

Different models demonstrated distinct strengths across specific management categories. Claude Pro excelled in complex decision-making tasks, such as managing complications and allergic reactions, while ERNIE Bot 3.5 performed better in fundamental medical areas like basic knowledge and early-stage treatment. These findings suggest that different models have strengths suited to various application scenarios. For emergency medicine contexts, where rapid decision support is critical, Claude Pro provides a robust tool due to its high accuracy and comprehensiveness. On the other hand, ERNIE Bot 3.5 remains highly practical in certain clinical knowledge domains, especially in Chinese-language environments.

### Score Distribution and Expert Consensus

The score distribution results indicate that Claude Pro received most of the highest ratings (5 points) and did not receive any of the lowest ratings (1 point). This further supports Claude Pro’s strong performance in terms of accuracy and completeness. In contrast, both ERNIE Bot 3.5 and ERNIE Bot 4.0 occasionally received lower ratings, with the 4.0 version performing notably weaker, achieving a maximum of only 0.375% (3/800) of the highest scores. This suggests that complete “hallucinations” were very rare [26]. The expert consensus results demonstrate that ERNIE Bot 3.5 and Claude Pro received relatively consistent expert feedback in most scenarios, indicating that these 2 models showed more stable performance.

### Clinical Application Prospects

LLMs, such as ChatGPT, demonstrate great potential in providing high-quality medical information across a wide range of medical conditions [27], including general oncology consultations [28], management of immune-related adverse events [29], and precise diagnosis of complex pediatric cases [30-32]. In our study, the median scores for many questions were 4 or 5, indicating that the information provided is generally accurate and comprehensive. The questions were designed to be open-ended rather than multiple choice, making them closer to real-life or clinical scenarios rather than examination-style questions.

The performance of Claude Pro and ChatGPT 4.0 suggests that LLMs can serve as efficient and reliable support tools for health care professionals in managing wasp sting cases, particularly in complex decision-making and emergencies, by rapidly providing comprehensive information. Baidu ERNIE Bot series shows promising potential as a Chinese-language model for managing wasp stings, with ERNIE Bot 3.5 performing especially well in key areas such as foundational knowledge and early intervention. However, differences in model performance highlight the importance of careful selection and use of LLMs in real-world clinical settings, especially in handling complex cases, as certain limitations may still exist.

The implementation of LLMs in low-resource settings presents both opportunities and challenges. Notably, the increasing accessibility of LLMs offers promising solutions. Many providers now offer free versions of their models and access costs continue to decrease. This trend toward the democratization of AI technology brings several advantages: minimal technical barriers to entry, simple user interfaces requiring basic training, and reliable access to up-to-date medical information across different settings [33]. While infrastructure and internet connectivity remain considerations in some areas, the growing availability of free or low-cost LLMs suggests their potential for widespread adoption in resource-limited health care settings.

Implementation considerations must address several key aspects: Data privacy and security protocols, ethical frameworks for AI deployment in clinical settings, cost-effectiveness analysis for different health care contexts, cultural and linguistic adaptation requirements, and integration with existing clinical workflows.

### Limitations

While this study offers valuable insights into the application of LLMs in the management of wasp stings, there are certain limitations to consider [34,35]. First, the issues of standardization and the limited number of clinical scenarios may not fully capture the complexity of wasp sting management. The study compared the models with expert consensus, we did not compare them with real-time clinical decision-making by human experts. Second, although expert ratings showed a high level of consistency, the model’s performance across different languages and cultural contexts requires further validation. However, our evaluation using both Chinese- and English-language models effectively covers the major geographical regions where wasp stings pose significant public health challenges, as these 2 languages serve the majority of affected populations in both Asian and Western health care settings.

In addition, while our evaluation focused on text-based interactions, we acknowledge that modern LLMs are evolving toward multimodal capabilities. Our current assessment framework did not incorporate these emerging functionalities, such as image analysis for skin reactions, voice recognition for emergency response, or integration with clinical data systems. This study primarily assessed the immediate performance of LLM models in isolated scenarios, without considering their consistency over time, decision fatigue, or adaptability to evolving clinical guidelines.

### Future Research Directions

Future research can further explore ways to optimize LLMs to enhance their applicability in specific medical fields [36], particularly in managing complex acute conditions and rare cases. Future research should explore direct comparisons between LLMs and human experts in real-time clinical decision-making. In addition, investigating how to seamlessly integrate LLMs into the decision-making processes of health care professionals is an important research direction. Continuous training and real-time updates of the models are also crucial for better clinical application. Future research should include larger sample sizes and more complex clinical scenarios to thoroughly evaluate the practical utility of LLMs in various medical fields. Furthermore, ethical considerations and data security issues related to the use of LLMs in health care remain areas that require in-depth exploration.

In addition, investigating how to seamlessly integrate LLMs into clinical workflows remains crucial. This includes studying their interaction with existing electronic health records, clinical decision support systems, and point-of-care diagnostics. Furthermore, ethical considerations, data security, and the cost-effectiveness of implementing these systems in various health care settings require thorough exploration. To improve the reliability of LLMs, future research should conduct longitudinal studies to assess the consistency, fatigue effects, and adaptability to dynamic clinical guidelines over long-term use.

### Conclusion

This study demonstrates that Claude Pro and ChatGPT 4.0 exhibit substantial promise in the clinical management of wasp stings, particularly excelling in complex decision support and information integrity. ERNIE Bot 3.5 shows balanced performance in Chinese contexts, although its upgrade, ERNIE Bot 4.0, did not yield significant improvements. Moving forward, researchers should explore the broader applications of LLMs across various medical specialties and focus on optimizing their seamless integration into clinical workflows. These findings underscore the potential of AI-driven tools to enhance medical decision-making, while also highlighting the need for continued refinement and evaluation in real-world health care settings.

## Data Availability

The datasets generated and analyzed in the current study are available from the corresponding author on reasonable request.

## Appendix

Multimedia Appendix 1 Standardized questions: engine 1.

Multimedia Appendix 2 Standardized questions: engine 2.

Multimedia Appendix 3 Standardized questions: engine 3.

Multimedia Appendix 4 Standardized questions: engine 4.

Multimedia Appendix 5 Scores for accuracy and completeness for each engine in each category.

## Abbreviations

AI: artificial intelligence
LLM: large language model
