# Supplementary material for: Clinical Management of Wasp Stings Using Large Language Models: Cross-Sectional Evaluation Study
Source: J Med Internet Res. 2025 Jun 4;27:e67489. doi: 10.2196/67489 (PMC12177424; doi:10.2196/67489)
Supplement: Multimedia Appendix 1 [file jmir_v27i1e67489_app1.docx]

| Engine 1 | | | | | |
| --- | --- | --- | --- | --- | --- |
|  | Basic Knowledge | Basic Knowledge | Basic Knowledge | Basic Knowledge | Basic Knowledge |
| Question | What are the common species of bee stings worldwide? | What are the main pathophysiological mechanisms of bee stings? | What are the main components of bee venom and their biological effects? | What are the global epidemiological characteristics of bee stings? | What are the common local and systemic reactions after bee sting? |
| Answer Engine 1 (ERNIE Bot 3.5) | Worldwide, there are many kinds of bees that sting people, and they vary according to their geographical distribution and ecological environment. However, based on common data and observations, the following are some of the more common stings worldwide: Bee: Bees are widely distributed and common stings. Their venom is acidic, relatively less toxic, and generally causes only mild local symptoms after the sting. Bee thorns with barbs, which will remain in the skin after the sting, and can not be stung again. Wasp (wasp, wasp): wasp is another common species of stings, including a variety of subspecies, such as bumblebee, wasp, etc. The venom of wasps is usually alkaline and more toxic. They are highly aggressive and nest-protective desires, and, when threatened, make group attacks. Wasnets do not have a barb, so they can repeatedly sting the target. The wasp: The wasp is also a sting species, and although its distribution and popularity may not be as widespread as bees and wasps, it is still common in some areas. They are smaller but equally capable of a sting. Marus bee: Marus bee is another species that may sting people, but its specific characteristics and distribution may vary from region to region. In addition, there are some unique sting bee species in specific areas or ecological environments, such as tiger bees, killer bees, etc., these bee species are usually more toxic and aggressive, posing a greater threat to humans. It should be noted that the bee species listed above may not be comprehensive due to differences in the diversity and geographical distribution of bee species. In addition, different bee species may also differ in toxicity, aggression, and behavioral habits. Therefore, in the encounter of bees, we should be vigilant and take corresponding protective measures. After being stung by bees, the corresponding first aid measures should be taken according to the different bee species. For example, for bee stings, thorns can be removed and alkaline substances such as soapy water to neutralize acidic venom; for wasp stings, acidic substances such as rice vinegar to neutralize alkaline venom. At the same time, if severe allergic reactions or systemic symptoms occur after the stings, seek medical treatment immediately. | The main pathophysiological mechanism of bee sting involves many aspects, mainly including the following aspects: 1. Venom composition and its effects When the tail spurs of bees (such as bees, wasps, etc.) are stabbed into human skin, the venom in its body will be injected into human tissues. These venoms are complex components, mainly including biological amines (such as histamine, serotonin), polypeptides, kinins, and enzymes. These components initiate a range of pathophysiological responses in the human body. Biological amines: such as histamine and seroxytryptamine, they can cause local vascular dilation, increased permeability, leading to local redness, heat pain and other inflammatory reactions. Peptides: They have a variety of biological activities that may be involved in the regulation of immune responses and inflammatory responses. Kinalliins: such as bradykinin, which can increase vascular permeability and promote the release of inflammatory mediators. Enzymes: such as hyaluronidase, which can degrade the tissue matrix, and promote the diffusion of venom in the tissue.2. Local reaction After the venom is injected into the human body, a local reaction occurs first. These reactions include: severe pain: due to the stimulation of the venom on the nerve endings. Numb: It may be caused by a local nerve palsy. Edema: increased tissue fluid exudation due to increased vascular permeability. Local nerve palsy: Some components of the venom may act directly on the nerve tissue, leading to local sensory and motor dysfunction.3. Systemic reaction When the venom enters the body, it may also cause a systemic reaction. These reactions include: acute allergic reactions: systemic allergic reactions, such as urticaria, breathing difficulties, rapid heart rate, etc. Intravascular hemolysis: Some venom components may destroy the red blood cell membrane, cause the rupture of red blood cells, release hemoglobin, and cause intravascular hemolysis. Rhabdomyolysis: Some components of the venom may damage muscle tissue, causing rhabdomyolysis, releasing large amounts of myoglobin and other muscle components. Abnormal blood coagulation function: Some components of the venom may affect the synthesis or function of blood coagulation factors, leading to abnormal blood coagulation function. Multiple organ function damage: including acute renal failure, liver damage, cardiac damage and nervous system damage. These damages may be caused by the effects of multiple components in the venom, May also be related to systemic inflammatory reactions and allergic reactions.4. Other mechanisms In addition, bee sting may cause some other pathophysiological mechanisms, such as reducing arterial blood pressure, affecting intercellular and intracellular signaling, and inducing cell apoptosis. These mechanisms may further aggravate the progression of the disease. In conclusion, the main pathophysiological mechanisms of bee stings involve multiple aspects, including the role of venom components, local reactions, and systemic responses. These mechanisms interact together to cause the clinical manifestations and complications of bee stings. Therefore, when handling bee stings, the effects of these mechanisms should be considered comprehensively, with targeted treatment measures adopted. | The main components of bee venom and their biological effects are a complex and diverse topic. The following is a detailed analysis of the main components of bee venom and their biological effects: 1. The main components of bee venom bee venom, whether bees or wasps, its venom components are quite complex, mainly including the following categories: biological amines: such as histamine, serotonin, etc. After these substances are injected into the body, they can cause local vascular dilation, increased permeability, leading to redness, heat pain and other inflammatory reactions. Polypeptides: bee venom peptide, totidipin, and bee venom peptide are the important polypeptide components in bee venom. These peptides have various biological activities, such as ganglion blocking effect, analgesia, and hemolysis. Enzymes: such as phosphoesterase A2, hyaluronidase, etc. These enzymes play a key role in bee venom, grading tissue matrix, promoting diffusion of venom in tissues, and triggering a range of biological effects. Other components: Bee venom may also contain amino acids, proteins, sugars, organic acids and other components, which also play an important role in the biological effects of bee venom.2. Biological effects of bee venom Local inflammatory reaction: after bee venom is injected into the body, the first local inflammatory reaction. This is mainly due to the stimulating effect of biogenic amines and polypeptide components on the local tissue, leading to the appearance of symptoms such as vascular dilatation, increased permeability, redness, swelling, heat and pain. Ganglion blockade: Polypeptide components in bee venom block the ganglia and can affect the conduction of nerve impulses, thus causing a series of neurological symptoms. Analgesia effect: Some components in bee venom have significant analgesic effect, and its analgesic effect is even better than some traditional analgesic drugs. This may be due to the ability of bee venom to inhibit the release of inflammatory mediators and the conduction of nerve impulses, thereby reducing pain perception. Hemolysis: bee venom peptide and phosphoestase A2 in bee venom have strong hemolysis. They are able to enhance the permeability of the red blood cell wall, leading to the rupture of the red blood cells and the occurrence of the hemolysis phenomenon. This hemolysis can be used clinically in the differential diagnosis of some diseases. Anticoagulant effect: Bee venom is anticoagulation in vitro or in vivoBlood action, which can prolong the coagulation time of blood. This anticoagulant effect may be related to some enzyme-like components in the bee venom. Immunomodulatory effect: bee venom also has an immunomodulatory effect, which can affect the bodys immune response and immune function. This may be related to the polypeptides and other immunoactive components in the bee venom. Other biological effects: In addition, bee venom may also have antibacterial, antiviral, anti-tumor and other biological effects. The specific mechanisms of these effects are not fully understood, but it has been shown that some components in bee venom have these potential effects. In conclusion, the main components of bee venom include many components, such as biological amines, polypeptides, and enzymes, which trigger a series of complex biological effects in the human body. These effects include both short-term effects such as local inflammatory responses and neurological symptoms and long-term effects such as immunomodulatory and antitumor. Therefore, these biological effects of bee venom should be fully considered and treated accordingly. | The global epidemiological characteristics of bee stings mainly include the following aspects: 1. Wide occurrence and high incidence areas global prevalence: bee stings is a common type of injury occurring in the world, with a wide range of impacts. High incidence areas: Although bee stings may occur around the world, the incidence may be higher in some areas due to ecological environment, climatic conditions and human activities. For example, areas with rich natural environment and dense vegetation, as well as areas with developed beekeeping or frequent wild bee activity, are high incidence of bee stings.2. universality of victim characteristics: Bee stings can occur in any age group, including children, adults and the elderly. Increased risk for specific groups: Some people are at higher risk of bee stings due to factors such as occupational characteristics, living habits or activity environment. For example, beekeepers, field workers, campers, and outdoor explorers, etc.3. Seasonal and temporal regularity of the onset: The onset of bee stings has obvious seasonal characteristics, usually with a higher incidence in spring and summer. This is related to the frequent activity of bees and other bees and more human outdoor activity in these seasons. Time rule: There may also be a time rule for bee stings during a day. For example, in the early morning and late evening, bees and other bees may move more frequently, increasing the risk of bee stings.4. Severity of symptoms and Complications Diversity of symptoms: The severity of bee stings varies from person to person; mild cases may only show symptoms such as local redness, pain and itching; in severe reactions, hemolysis, liver and kidney damage and even shock and death. Risk of complications: For some populations, bee stings can cause severe complications. These complications may involve multiple organ systems, such as cardiovascular system, respiratory system, nervous system, etc., which seriously threaten the life safety of patients.5. Epidemiological Data and Trends Global Statistics: It is reported that more than 100 million people are bitten by hymenoptera insects every year, including bees, wasps and wasps. About 56 to 94 percent of people worldwide may be killed by bees in their livesStings such as wasps or wasps, about 10% of which can be life-threatening. Morbidity and mortality: The incidence and mortality of bee stings vary by region, season, population characteristics and other factors. The incidence of bee stings may rise significantly in certain areas or in specific seasons. At the same time, for some people who are highly sensitive to bee venom, bee stings can lead to serious complications and even death. In conclusion, the global epidemiological characteristics of bee sting include widespread occurrence, widespread victims, seasonal and temporal onset, diverse severity of symptoms and possible serious complications, and certain morbidity and mortality. Therefore, it is important to prevent bee stings and improve the public awareness and coping ability of bee stings. | After bee stings, patients often experience a series of local and systemic reactions due to bee venom injection. These reactions can be light or heavy, the specific manifestations are as follows: 1. Local reaction redness: the sting site will immediately appear redness, which is due to bee venom stimulates the skin, resulting in local vascular dilation and increased permeability caused by. The extent and extent of redness vary by individual differences, but usually peak within hours. Pain: After bee stings, patients will experience severe local pain, due to the stimulation of the venom on the nerve endings. The extent and duration of pain also vary from person to person, but generally gradually decreases as the swelling subside. Itch: Some patients will experience local itching symptoms, which may be caused by allergic reactions or local inflammatory reactions. Blae or skin necrosis: In severe cases, blisters or skin necrosis may occur at the sting site. This is due to the strong stimulating and destructive effect of the venom on the local tissue. Central necrosis and suppuration: central necrosis and suppuration may occur in the bee sting site, usually less than 10cm, but severe cases may exceed this range. This necrosis and suppuration may persist for several days and may cause non-infectious lymphangitis and mild systemic symptoms.2. Systemic reaction and fever: Some patients will have fever symptoms after bee stings, which is caused by the systemic immune reaction caused by the venom. The degree and duration of fever varies with individual differences. Nausea, vomiting, abdominal pain, diarrhea: These symptoms are part of the systemic toxic response and may accompany fever. They suggest that the venom already impacts on the digestive system. Dizziness, headache: Some patients will feel dizziness and headache, which may be due to the stimulation effect of venom on the nervous system. Fgety, muscle spasm, coma: In severe cases, patients may appear irritability, muscle spasm and even coma symptoms. These symptoms indicate that the venom has had serious effects on the CNS. Anaphylactic shock: For some patients allergic to bee venom, symptoms of anaphylactic shock may occur immediately after a bee sting. This is a severe systemic allergic reaction and can be life-threatening.sensitivitySymptoms of shock include urticaria, laryngeal edema, wheezing, shortness of breath and even bronchospasm and asphyxia. Acute renal failure: In rare cases, bee stings may cause acute renal failure. This is due to the toxic effect of the venom on the kidney. Acute renal failure is a serious complication that requires immediate medical intervention. Overall, local and systemic responses after bee stings are varied and severity varies by individual differences. For mild reactions, it can generally relieve spontaneously or improve after simple treatment; but for severe reactions or anaphylactic shock, they need immediate medical treatment for emergency rescue. |
|  | Early Treatment | Early Treatment | Early Treatment | Early Treatment | Early Treatment |
| Question | What is the first step after the wasp sting? | What is the difference between bee stings and wasp stings? How to accurately identify and judge it? | How to clean the wound of bee sting correctly? How to choose the cleaning liquid? | How to deal with local pain after a wasp sting? | What is the procedures of field first aid for bee stings? |
| Answer Engine 1 (ERNIE Bot 3.5) | The first step after treating the wasp stings is to pick out the stings immediately. When wasps sting, they may remain in the body, and they often have venom, which can cause a series of uncomfortable reactions. Therefore, after the wasp sting, the first priority is to remove the sting from the wound as soon as possible. When picking out stings, we should pay attention to the following points: Do not squeeze the wound: squeezing the wound may cause the venom to spread in the human body, aggravating the symptoms of poisoning. Therefore, when picking out stings, try to avoid squeezing the wound. Use the right tools: tools such as needles or medical tweezers can be used to pick out the stings. These tools should be disinfected in advance to avoid infection. Soft operation: When picking out the stings, the operation should be gentle to avoid causing secondary injury to the wound. After picking out the sting, the following measures can be taken to further treat the wound: clear the wound: wasp venom is weakly alkaline, so you can use acidic substances such as vinegar to clean the wound, in order to achieve the effect of acid and base neutralization, reduce the number of toxins in the human body. Ice compress: after being sting, the injury may appear redness, pain and other adverse symptoms. At this time, ice can be taken as soon as possible, directly applied to the affected area, to reduce the pain and swelling. Medication: If the pain is severe, take pain medication to relieve the pain. At the same time, if there are allergic reactions, such as rash, nausea and vomiting, palpitation and other symptoms, seek medical advice in time and receive anti-allergy treatment. Please note that if there is a severe systemic poisoning reaction, such as shock, immediately call the emergency number and inform the medical staff of the sting so that they can take timely treatment measures. | There are obvious differences between bee stings and wasp stings in many aspects. The following are three aspects of wound characteristics, identification methods and judgment basis: 1. Wound characteristics of bee sting: bees are straight and barbed, which determines the direction of bee stings is vertical injection. Therefore, when the bees are stung to fly away, the sting needle is not easy to pull out, and often stay on the skin. Wound manifestations: the wound often appears redness, pain and itching symptoms, and because the needle is not easy to pull out, may see the needle left on the skin. In addition, the toxin of honey bees is weakly acidic, and the wound may be accompanied by some acidic reaction. Characteristics of the wasp sting needle: the wasp sting needle is curved and not barbed, and the direction of the sting needle is inclined, so it is easy to pull out the sting needle. The wasps can repeatedly attack humans, and there are generally no poison in the wound. Wound manifestations: the wound may appear redness, pain, erythema, papules and other symptoms. Because the wasp toxin is weakly alkaline, the wound may be accompanied by a certain alkaline reaction. If stung by a large number of wasps, large swelling, blisters, rash, blood stasis, and bleeding may occur around the wound.2. Identification method: If conditions permit, observe whether there is any residue at the wound. If the sting needle is longer and difficult to pull out, it may be a bee sting; if there is no poison thorn to pull at the wound, it may be a wasp sting. Medical history: Check out the history of recent contact with bees or wasps, and the specific situation of stings. Check the wound symptoms: carefully observe the wound redness, pain, itching and other symptoms, and whether there are blisters, rash and other serious manifestations.3. Judging the wound residue: Whether there is a sting needle residue in the wound is an important basis for judging bee stings and wasp stings. Wound symptoms: After bee stings, local redness, pain and itching may be obvious, systemic allergic reactions such as urticaria and dyspnea. Toxin properties: bee toxin is weak acidic, wasp toxin is weak alkaline. This property can be tentatively judged by the acid-base reaction of the wound, but does not combineNot absolutely accurate. In conclusion, bee and wasp stings differ significantly in sting characteristics, wound performance, and identification methods. Bee stings and wasp stings performed by careful wound observation, history examination and wound symptoms can be accurately identified and judged. When dealing with stings, the corresponding treatment measures should be taken according to the venom characteristics of different bees. | Proper cleaning of bee stings is one of the important steps in dealing with such injuries, which helps to reduce the risk of infection and relieve local symptoms. The following steps are how to properly clean bee stings and the selection of cleaning solution: 1. Cleaning steps to remove stings: First, if there is a bee sting in the wound, use tweezers or similar sterile items to clip them out as soon as possible. Be careful not to squeeze the wound with your fingers or tweezers to avoid squeezing out more venom. Clean the wound: Wash the wound carefully with gentle soap and running water. Sosoapy water can help remove contaminants from the wound surface and reduce the risk of infection. Cleaning should be gentle to avoid secondary damage to the wound. Dry the wound: gently dry the wound with a clean towel or paper towel to ensure that the wound is dry.2. Cleaning liquid Choose soapy water: soapy water is one of the most commonly used cleaning liquid. Its alkaline component neutralizes the acidity of the bee venom and helps reduce pain and swelling. Choose a non-irritating mild soap for cleaning. Sodium bicarbonate solution: 5% sodium bicarbonate solution is also an effective cleaning solution. It is more alkaline and can neutralize the venom more effectively, but it should pay attention to the concentration to avoid irritation to the skin. Avoid strong irritant disinfectant: such as iodine, alcohol and other disinfectant although has a bactericidal effect, but the irritation is strong, may aggravate the discomfort of the wound, so it is not recommended to clean the wound of bee stings.3. Note to avoid squeezing the wound: during the cleaning process, avoid squeezing the wound to avoid venom diffusion or aggravating symptoms. Observe the wound changes: after cleaning, we should closely observe the changes of the wound, such as swelling, pain and other symptoms are relieved. If symptoms worsen or allergic reactions occur, seek medical attention immediately. Indications for medical treatment: If the wound has severe swelling, unbearable pain, dyspnea, nausea and other systemic allergic reaction symptoms, you should go to the hospital immediately. In conclusion, the correct cleaning of bee stings is an important measure to reduce symptoms and prevent infection. When choosing a cleaning solution, give priority to alkaline solutions such as soapy water or sodium bicarbonate solution, and avoid the use of highly irritating disinfectant. At the same time, attention should be paid to avoid crush injury during the cleaning processMouth, and closely observe the wound changes. | After wasp sting, local pain is one of the common symptoms, in order to effectively alleviate the pain, the following measures can be taken: 1. Emergency treatment to remove stings: If there is wasp stings remaining in the wound, you should use disinfected tweezers or needles as soon as possible to avoid squeezing the wound to prevent further spread of venom. Wound cleaning: Wash the wound with running water or normal saline to reduce the residue of the venom on the wound surface and to reduce the risk of infection.2. Local treatment cold compress: method: wrap with ice or cold towel and apply around the wound, last for 15-20 minutes each time, repeat every 2-3 hours. Function: Cold compress can shrink blood vessels and reduce local blood circulation, thereby reducing swelling and pain. External drugs: anti-inflammatory ointment: such as erythromycin ointment, a hundred state ointment, applied to the wound, help to reduce inflammatory reactions and pain. Anti-allergy ointment: such as desonide cream, halomyasone cream, etc., suitable for local redness, itching and other symptoms caused by bee venom allergy. Local closure treatment: Circular closure with procaine solution around the sting site can help reduce pain and swelling.3. Oral medication for painkillers: If the pain is unbearable, take non-steroidal oral anti-inflammatory drugs, such as ibuprofen sustained-release capsules and acetaminophen, as prescribed to the doctor to relieve the pain. Antiallergic drugs: such as cetirizine tablets, loratadine, etc., suitable for patients with systemic allergic reaction, can reduce the pain and discomfort caused by allergic reaction.4. Other precautions to avoid scratching: keep the wound clean and dry, avoid scratching the wound with your hands to aggravate infection or cause pain. Diet adjustment: avoid eating spicy and stimulating food, eat more foods rich in vitamin C and vitamin E, such as fruits and vegetables, and help to promote wound healing and reduce pain. Rest and observation: rest properly, avoid strenuous exercise and overwork. Observe the wound changes and systemic symptoms closely, and seek medical attention immediately if there are abnormalities. Indications for medical treatment: if the wound has severe redness, increased pain, dyspnea, nausea and other systemic allergic reaction symptoms, you should immediately go to the hospital to avoid the delay of the condition. In conclusion, local pain after wasp stingTreatment includes emergency treatment, local treatment, oral medication treatment, and other considerations. In the process of treatment, appropriate methods should be selected according to the specific situation of the patient and attention should be paid to observe the condition changes. | The on-site first aid of bee stings should follow the following procedures to ensure that the injured are timely and effectively treated: 1. Preliminary evaluation and judgment evaluation of the condition of the injured: first, observe the state of consciousness, breathing, heartbeat and other vital signs, to determine whether they are in a state of emergency. Identify bee species: identify sting species (such as bees, wasps, etc.) because the properties of different species varies venom.2. Emergency treatment to remove stings: use sterilized tweezers or needles to gently pick out the stings in the wound to avoid squeezing the wound to prevent the spread of venom. If the tools cannot be found in the field and are confirmed to be bee stings, the thorns can be carefully sucked out with your mouth, but pay attention to oral hygiene to avoid infection.proximal end: Ring a 10 cm proximal end of the wound to slow the spread of venom. It should not be too tight, so as not to affect blood circulation. The bandage time should not exceed 2 hours, but 1 minute every 15 minutes to check the limb blood flow.3. Local treatment of wound cleaning: use flowing water or normal saline to wash the wound to reduce venom residue. Choose appropriate cleaning solution according to bee species: bee venom is acidic, and can be cleaned with weak alkaline liquid such as soapy water and 5% sodium bicarbonate solution; wasp venom is alkaline, and weak acidic liquid such as vinegar can be cleaned. Local cold compress: Use ice packs or cold towels to relieve pain and swelling.4. Drug treatment topical drugs: topical calamine lotion, antihistamines, painkillers or corticosteroid ointment, to dissipate inflammation and relieve pain. When the swelling is seriously accompanied by blister exudation, 3% boric acid aqueous solution can be applied wet. Oral drugs: if the pain is unbearable, it can be oral non-steroidal anti-inflammatory drugs such as ibuprofen to relieve the pain. In case of allergic reactions, such as systemic rash, breathing difficulties, take allergic drugs such as cetirizine and loratadine immediately and seek medical advice as soon as possible.5. Observation and close observation: after emergency treatment, closely observe the condition changes of the injured, including the wound swelling, whether the pain is relieved and whether new symptoms appear. Seek medical attention: if the patient has severe allergic reaction, dyspneaWhen nausea and other systemic poisoning symptoms, you should call the emergency number immediately and be sent to the hospital for treatment. Even if mild stings, if the injured appear persistent discomfort or symptoms worsen, should also timely seek medical attention for examination and treatment.6. Prevention and precautions to avoid bee swarm: try to wear long sleeves and light-colored clothes during outdoor activities, and avoid using perfume and other irritating items to reduce the risk of bee stings. Learn first aid knowledge: master the basic first aid knowledge and skills, so as to be able to quickly and effectively conduct self-rescue and mutual rescue in an emergency. In conclusion, the on-site first aid of bee stings should follow the process of preliminary evaluation and judgment, emergency treatment, local treatment, drug treatment, observation and medical treatment, and prevention and precautions. Through timely and effective treatment, can minimize the pain of the injured and complications. |
|  | Allergy Response Management | Allergy Response Management | Allergy Response Management | Allergy Response Management | Allergy Response Management |
| Question | What are the typical manifestations of allergic reactions caused by bee stings? | How to identify and urgently manage the anaphylactic shock caused by bee stings? | What does the drug treatment plan of allergic reaction after bee sting have? What is the first-line medication? | How to assess the risk of allergic reactions in patients with bee stings? | How to prevent and manage late hair-type allergic reaction after bee sting? |
| Answer Engine 1 (ERNIE Bot 3.5) | Allergic reactions from bee stings may have a range of typical manifestations that vary according to individual differences and the severity of allergic reactions. The following are some common typical manifestations: 1. Local symptoms redness, pain, itching: in the bee sting site, there will be obvious redness, pain and itching. This is the normal immune response to bee venom, but it may also predict the occurrence of allergic reaction. Rash, urticaria: with the aggravation of allergic reaction, local rash or urticaria, manifested as red plaques or papules on the skin, accompanied by a strong sense of itching. Blae, ecchymosis: below serious case, local still may appear blister, ecchymosis, this is the performance of bee poison to cause damage to skin tissue.2. Systemic symptoms Fever, headache, dizziness: When the allergic reaction affects the whole body, patients may have fever, headache, dizziness and other symptoms. These symptoms may indicate that the allergic reaction is already more severe and requires attention. Nausea, vomiting, diarrhea: Some patients may have gastrointestinal symptoms as allergic reactions, such as nausea, vomiting, diarrhea, etc. This is the result of the stimulatory effect of bee venom on the gastrointestinal mucosa. Dyspnea, wheezing: If the allergic reaction affects the respiratory system, the patient may develop dyspnea, wheezing and other symptoms. This is a sign of severe allergic reactions such as laryngeal edema or bronchospasm and requires immediate medical attention. Palpitations and arrhythmia: allergic reactions may also stimulate the cardiovascular system, leading to palpitations, arrhythmia and other symptoms. These symptoms may indicate that an allergic reaction already causes damage to the cardiovascular system.3. Severe anaphylactic shock: In rare cases, bee stings may trigger anaphylactic shock. This is a severe systemic allergic reaction, manifested by a sharp drop in blood pressure, confusion, and cold limbs. If not treated in time, it may endanger the life of the patient. Multiple organ failure: In cases of very severe allergic reactions, patients may develop symptoms of multiple organ failure. This is a result of bee venom causing damage to multiple organ systems that requires immediate rescue treatment. Attention Bee stings should be done immediatelyMedicine, especially when the systemic symptoms or severe allergic reactions occur. Before seeking medical treatment, some emergency measures can be taken, such as removing stings, cleaning wounds, local cold compress, etc., to reduce symptoms and prevent venom spread. For people known to be allergic to bee venom, attention should be paid to avoid contact with bees and other insects when engaged in outdoor activities, and carry anti-allergy drugs for emergency. | Identification and emergency management of anaphylactic shock caused by bee stings is crucial as this may threaten the patient of the patient. The following are detailed identification and emergency treatment steps: 1. Identification of anaphylactic shock 1. Typical symptoms Identification of vital signs: pulse speed or not palpable, blood pressure drop (systolic pressure <90mmHg), or even shock blood pressure (<70 / 50mmHg). The patient may have pale face, cold limbs and cold sweat. Neurological symptoms: Patients may show irritability, extreme fear, and even changes in consciousness, such as drowsiness, coma, etc. There may also be severe skin itching, accompanied by numbness, or peripheral neuritis symptoms such as acupuncture or ant walking. Respiratory symptoms: dyspnea, laryngeal edema, wheezing, suffocation, cyanosis, and even suffocation. Other symptoms: skin flushing, extensive urticaria and (or) angioneurotic edema, may also be accompanied by watery nose, hoarseness, irritating cough, continuous sneezing, nausea, vomiting, abdominal pain, diarrhea and other symptoms, severe cases can appear urine and feces incontinence.2. Auxiliary examination Although a comprehensive auxiliary examination may not be possible during on-site first aid, a preliminary judgment can be made by observing the patients vital signs and symptoms. If conditions permit, blood routine, blood biochemistry, electrocardiogram and other tests can be carried out as soon as possible to understand the systemic condition of the patient.2. Emergency treatment 1. Stop contact with bees immediately: make sure the patient does not continue to be stung and move away from the area where the bee is.2. Remove stings: Use sterilized tweezers or needle tips to gently pick out the stings in the wound to avoid squeezing the wound to prevent the venom from spreading.3. Keep the respiratory tract unobstructed: untie the patients collar and belt, remove the mouth and nose secretions, and keep the respiratory tract unobstructed. If the patient has symptoms such as dyspnea or laryngeal edema, oxygen inhalation should be treated immediately, and emergency measures such as endotracheal intubation or tracheotomy should be prepared.4. Indrinephrine injection: Adrinephrine is the drug of choice for anaphylactic shock, which can quickly contract blood vessels, increase blood pressure, improve cardiac function, relieve bronchospasm, etc. Adrinephrine injection should be given by subcutaneous or intramuscular injection as soon as possible, at the dose according to the patients weight andThe condition depends on.5. Establish venous access: quickly establish venous access for the infusion of fluids, drugs, etc. Anti-allergic drugs such as dexamethasone sodium phosphate injection can be given to relieve allergic reactions. At the same time, patients vital signs and condition changes should be closely monitored.6. Close observation: In the process of first aid, the changes of the patients condition should be closely observed, including changes in vital signs such as blood pressure, heart rate and respiration, as well as changes in allergic symptoms such as skin and mucous membrane. Once the condition changes or the symptoms worsen, appropriate emergency measures should be taken immediately.7. Transfer to the hospital: After the initial emergency treatment, the patient should be transferred to the hospital for further treatment as soon as possible. In the process of transport, we should continue to closely observe the changes of the patients condition, and maintain communication with the hospital, so as to timely inform the patients condition and treatment. In conclusion, the anaphylactic shock caused by bee stings should be quickly identified and treated urgently, so as to minimize the pain and complications of patients. At the same time, in daily life should be paid to the prevention of bee stings, avoid with bees and other insects. | The drug treatment plan of allergic reaction after bee stings mainly includes the following aspects, and the first-line drugs are mainly antihistamines: 1. Drug treatment plan 1. Effect of antihistamines: antihistamine drugs can reduce the itching, redness and other symptoms caused by allergic reaction. First-line medication: commonly used antihistamines include cetirizine, loratadine, ebastine, etc. These drugs can be taken orally, have a faster effect, and have a better therapeutic effect for mild to moderate allergic reactions. Usage: The dose is usually determined based on the condition and the patients weight, usually once or more daily oral doses.2. Corticosteroid action: Corticosteroids have strong anti-inflammatory effects, which can quickly control local inflammatory reactions, and are suitable for severe local redness, pain, and systemic allergic reactions. Method: It can be used externally (such as hydrocortisone ointment) or injected (such as dexamethasone injection). Note: Long-term use of corticosteroids may have some side effects, so they should be used under the guidance of a doctor.3. Effect of immunomodulators: Immunomodulators can regulate the immune response of the body, reduce excessive immune response, and have a certain therapeutic effect on severe allergic reactions. Commonly used drugs: such as cyclosporine A, methylprednisolone, etc. Precautions: The use of these drugs requires strict control of the indications and contraindications to avoid abuse.4. Antibiotic action: When the infection is caused by improper local wound treatment after bee stings, antibiotics are needed for treatment. Commonly used drugs: such as amoxicillin, cefixime, etc. Usage: Choose appropriate antibiotics and dose according to the degree of infection and patient condition.5. Analgesic drugs: analgesic drugs can relieve the pain caused by bee stings. Common drugs: such as ibuprofen sustained-release capsules, acetaminophen tablets, etc. Usage: These over-the-counter pain medication are effective enough for mild to moderate pain.2. Other treatment measures 1. Local treatment to remove stings: Use sterilized tweezers or needles to gently pick out stings in the wound. Clean the wound: Wash the wound with flowing water or normal saline to reduce the venom residue. Cold compress: Use ice packs or a wet towel to cool the wound, To relieve the pain and swelling.2. Anti-allergic treatment For severe allergic reactions, such as anaphylactic shock, it is necessary to inject adrenaline and other emergency drugs immediately, and send them to the hospital for further treatment as soon as possible.3. Precautions Read the instructions carefully and follow the instructions before using any medication. Closely observe the changes in the condition, and seek medical attention in case of aggravation or new symptoms. Avoid scratching the wound to aggravate the infection or cause other complications. Pay attention to the prevention of bee stings in outdoor activities, such as wearing long sleeves and trousers, avoid the use of perfume and other irritating items. In conclusion, the drug treatment plan for anaphylaxis after bee stings takes antihistamines as the first-line drug use, and the appropriate corticosteroids, immunomodulators, antibiotics and analgesic drugs are selected for comprehensive treatment according to the needs of the condition. In the process of treatment should pay attention to observe the changes in the disease and timely treatment | Assessing the risk of anaphylaxis in patients with bee stings is a comprehensive process, considering multiple factors. Here are some key assessment steps and considerations: 1. Medical history Ask: Ask patients about a history of allergy, especially to bee or other insect stings. Patients with a history of allergies are more likely to have severe allergic reactions. Basic diseases: Learn if the patient has underlying diseases such as cardiovascular and cerebrovascular diseases and endocrine system diseases, which may increase the risk of anaphylactic shock after bee stings. Family history of allergy: ask the patient for a history of allergic disease in his family, because the allergic reaction has a certain genetic tendency.2. Clinical evaluation of local symptoms: observe the severity of redness, pain, itching and other symptoms in the site of bee stings. Although local symptoms do not necessarily have a direct indication of systemic allergic reactions, they are one of the important indicators to assessing the risk of allergic reactions. Systemic symptoms: To for systemic symptoms such as fever, headache, nausea, vomiting, abdominal pain, dyspnea, and throat and tongue swelling. The appearance of these symptoms may indicate that the patient is experiencing a systemic allergic reaction.3. Laboratory tests Serum-specific IgE test: the sensitivity of patients to bee venom can be assessed by measuring the serum level of specific IgE antibodies to bee venom. This is one of the most important laboratory tests for the diagnosis of allergic reactions to bee stings. Skin test: conduct bee venom skin prick test or subcutaneous injection test to observe whether the patient has local redness, itching and other allergic reactions. The results of skin tests can help doctors determine how sensitive a patient is to bee venom.4. Comprehensive evaluation of allergic reaction grade: According to the patients clinical manifestations and laboratory examination results, allergic reactions are divided into different levels (such as common local reactions, large local reactions, systemic severe allergic reactions, etc.). This helps doctors to develop personalized treatment options and preventive measures. Risk assessment: To assess the risk of anaphylaxis after bee stings, considering the patients allergy history, underlying disease, family allergy history, clinical manifestations and laboratory findings. For high-risk patients, more close observation and treatment should be givenTimely treatment.5. Preventive measures to avoid recontact: For patients with a known bee venom allergy, try to avoid recontact with bees or other insects. In outdoor activities, should wear long sleeves and light colored clothes, avoid using perfume and other stimulating items. Carrying emergency medication: It is recommended for patients allergic to bee venom to carry anti-allergic drugs (such as antihistamines) and adrenaline autoinjectors (such as EpiPen) in order to provide prompt self-treatment in the event of an allergic reaction. In conclusion, assessing the risk of anaphylaxis in patients with bee stings requires comprehensively considering multiple factors, including medical history inquiry, clinical performance evaluation, laboratory tests, and comprehensive evaluation. Through a comprehensive evaluation, a personalized treatment plan and preventive measures can be developed to reduce the wind of allergic reactions in patients. | The prevention and management of late-onset anaphylaxis after bee stings requires many aspects, including preventive measures, emergency treatment, and follow-up management. Here are some specific recommendations: 1. Avoid contact with insects such as bees: wear long sleeves, trousers and light-colored clothing outdoors, and avoid bright or floral clothes to reduce the attention of insects such as bees. Avoid cosmetics with a strong smell, such as perfumes and hair gel, which may attract bees. Do not stay or play near the hive to avoid disturbing the normal life of the bees. Keep the environment clean: regularly clean outdoor environments such as courtyards and gardens, and reduce food residues and water traces to reduce the probability of bee contact with people when foraging or drinking water. Secure or remove unnecessary garbage bins, empty cans and other containers to avoid becoming a place for bee nests. Enhance self-protection awareness: in outdoor activities, pay attention to observe the surrounding environment, especially trees, flowers and other places where bees may be hidden. If encountered by bees and other insects close to them, try to keep calm, do not panic, to avoid causing their attack.2. Emergency treatment, leave the scene quickly: Once stung by bees, leave the scene immediately to avoid further attack. Remove stings: Carefully examine the wound, and if a bee sting remains in the skin, gently pick it out using sterilized tweezers or needle tips. Avoid squeezing the wound directly with your hand to avoid venom spread. Clean the wound: Clean the wound with clean water or soapy water to remove residual venom and dirt. Wash when the action should be gentle, to avoid aggravating the wound injury. Local cold compress: Use ice packs or cold towels for cold compress on the wound to help reduce pain and swelling. But pay attention to the cold compress time should not be too long, so as to avoid frostbite skin. Observe the changes of the condition: after being stung by bees, they should closely observe the changes of their own condition. If allergic reaction symptoms (such as itchy skin, redness, breathing difficulties, etc.), seek medical attention immediately.3. Follow-up management for medical treatment: If there are symptoms of delayed allergic reaction, such as rash, urticaria, breathing difficulties, etc., please seek medical treatment immediately. Doctors will give the corresponding anti-allergy treatment according to the condition,Such as the use of antihistamines, corticosteroids, etc. Avoid re-contact: During recovery, avoid re-exposure to substances such as bees and their venom. Keep the skin clean: During the recovery period, the affected skin should be kept clean and dry to avoid infection. If necessary, use topical drugs as prescribed. Psychological support: for patients with anxiety, fear and other psychological problems due to bee stings, appropriate psychological support should be given and counseling. Help them to establish the correct cognitive concept, relieve the psychological pressure. In conclusion, the prevention and management of late-onset anaphylaxis after bee stings requires multiple aspects, including preventive measures, emergency management, and subsequent management. Through scientific and reasonable preventive measures and timely and effective emergency treatment measures, the risk of allergic reaction and its symptoms can be reduced. At the same time, you need to keep your skin clean, avoid reexposure to allergic substances and receive the necessary psychological support during your recovery. |
|  | Complication Management | Complication Management | Complication Management | Complication Management | Complication Management |
| Question | What serious complications may occur after a bee sting? | What are the causes of respiratory failure caused by bee stings? How to deal with? | What is the management strategy for renal insufficiency after bee stings? | What does the reason that bee sting secondary hair anemia have? How to deal with? | What is the way of blood purification treatment after bee sting? How to choose? |
| Answer Engine 1 (ERNIE Bot 3.5) | Various serious complications that can occur after bee stings, which can be severe and even life-threatening. The following are some major serious complications: 1. Symptoms of systemic allergic reactions: rash in the affected area, laryngeal edema, dyspnea, decreased blood pressure, sweating, chest tightness, etc. These symptoms often develop rapidly and can be life-threatening. Treatment: As soon as a systemic allergic reaction occurs, intravenous fluids must be given immediately, with large amounts of rehydration, and promethazine, epinephrine, and corticosteroid hormones. If the dyspnea cannot be relieved, emergency treatment such as tracheotomy is required.2. Multiple organ function injury Liver and kidney injury: bee venom may lead to multiple organ function injury such as liver and kidney, such as jaundice, decreased appetite, oliguria and even anuria. In this case, the patient needs to be given a blood purification treatment. Nervous system damage: bee venom may induce encephalitis, cerebrovascular accident, etc., leading to consciousness disorders, dizziness, headache, delusion and other symptoms. Respiratory injury: In addition to laryngeal edema and dyspnea, it may also lead to tracheal spasm from allergic reactions and even acute respiratory distress syndrome (ARDS). Circulatory system injury: palpitations, chest tightness, chest pain and other symptoms may occur, and may cause coronary artery spasm, hypotensive shock due to allergic reactions, resulting to arrhythmia and other serious consequences. Digestive system damage: mild manifestations for nausea, vomiting, abdominal distension, diarrhea; severe cases may appear hematemesis, melena, jaundice and other symptoms. Blood system injury: possible subcutaneous bleeding points, ecchymosis, hematemesis, hematochezia, and hematuria in non-sting sites, as well as abnormal coagulation function.3. Local malignant lesions If the toxicity is more serious, it will lead to ulceration, necrosis, etc. in the site, and scars may be formed after the later healing.4. Allergic reaction intensifies bee stings is often a process of sensitization. Once allergens appear in the body, the next bee sting may cause more serious allergies.5. Muscle injury Some patients will have damage to the central nervous system or peripheral nervous system after bee STings, which may lead to muscle spasm, rigidity and other manifestations, Thus pulling on the muscles. In conclusion, the serious complications that may arise after bee stings involve multiple systems and often develop rapidly. Therefore, after bee stings, appropriate first aid measures should be taken immediately and seek medical attention as soon as possible for further treatment and observation. At the same time, for people with allergies, we should pay special attention to avoid connecting with bees and other insects in outdoor activities. | The causes and treatment methods of respiratory failure caused by bee stings are analyzed as follows: 1. Causes of respiratory failure The role of neurotoxins in bee venom: bee venom contains a variety of bioactive ingredients, including neurotoxic substances. These neurotoxins are able to affect the function of the nerve-muscle joints, causing muscle contraction weakness and even paralysis, including the weakness of the respiratory muscles. Neurotoxins may also cause central nervous system inhibition, resulting in respiratory paralysis that further develops to respiratory failure. Allergic reaction: Some people are allergic to bee venom. When the bodys immune system recognizes and attacks these foreign substances, it will release a large number of inflammatory mediators and cytokines, leading to systemic allergic reaction. Severe allergic reactions can cause laryngeal oedema, bronchospasm, etc., resulting in respiratory tract stenosis, and then affect the respiratory function, resulting in respiratory failure. Respiratory system injury: after bee stings, local toxins may cause direct damage to the respiratory system, such as causing inflammatory reactions such as trachea, bronchial mucosal congestion, edema and other inflammatory reactions, which may affect the respiratory function.2. Emergency treatment: immediately transfer the patient to a safe area to avoid continued contact with bees or other substances that may cause allergic reactions. If patients develop respiratory failure symptoms such as dyspnea and cyanosis, cardiopulmonary resuscitation measures such as artificial respiration or chest compressions should be performed immediately to maintain blood circulation and oxygen supply. Antiallergy therapy: Antiallergy therapy should be given as early as possible in patients with definite or suspected bee venom allergy. Commonly used allergic drugs include antihistamines (such as cetirizine, loratadine, etc.) and glucocorticoids. If the patient has developed a severe allergic reaction (such as anaphylactic shock), adrenaline should be given immediately and sent to the hospital for further treatment as soon as possible. Maintain airway patency: In patients with laryngeal edema or bronchospasm, oxygen inhalation and aerosol bronchodilators (such as salbutamol) should be given to relieve the symptoms of dyspnea. If the patient has an emergency such as asphyxia, surgical procedures such as endotracheal intubation or tracheotomy should be performed immediately to establish an artificial airway and maintain respiratory function. Systemic treatment: to give patientsAppropriate rehydration and nutritional support therapy to maintain water and electrolyte balance and energy supply. Comprehensive treatment measures such as anti-infection and anti-shock were given according to the specific situation of the patient to improve the overall condition of the patient and promote rehabilitation. Observe the changes of the condition: in the process of treatment, the changes of the patients condition should be closely observed, including the changes of vital signs such as respiratory rate, heart rate and blood pressure, and whether there are new allergic symptoms. Once the abnormal conditions are found, they should be timely handled and adjusted to the treatment plan. In conclusion, respiratory failure caused by bee stings is a serious complication, which requires emergency treatment measures, timely anti-allergy treatment, airway patency, and systemic treatment to save the lives of patients. At the same time, attention should also be taken to avoid contact with bees to reduce stings. | The management strategy of renal insufficiency after bee sting is a comprehensive treatment process designed to rapidly control the condition, reduce symptoms, restore renal function and prevent complications. The following are some major management strategies: 1. Emergency treatment and initial assessment to quickly get away from the scene: after being stung by bees, patients should immediately stay away from the swarm to avoid further injury. Local treatment: Remove the residual stings from the stings and clean the wound with water or soapy water to reduce local symptoms. Preliminary assessment: Observe the patients symptoms, especially for signs of renal insufficiency, such as reduced urine volume, edema, nausea, vomiting, etc.2. Fluid and Electrolyte Management Fluid resuscitation: In patients with shock or circulatory instability, fluid resuscitation should be performed immediately to maintain blood pressure and organ perfusion. Electrolyte monitoring and adjustment: when renal dysfunction occurs, patients may experience electrolyte disorders, such as hyperkalemia, hyponatremia, etc. Therefore, the electrolyte levels need to be closely monitored and adjusted as needed.3. Kidney function support therapy Blood purification: For patients with severely impaired renal function, blood purification treatment, such as hemodialysis or peritoneal dialysis, may be required to remove toxins and excess water in the body and maintain the stability of the internal environment. Diuretic therapy: In the case of renal function is not completely lost, diuretics can be tried to promote urine discharge, reduce edema and relieve renal function damage.4. Drug treatment Anti-allergic treatment: for allergic reactions caused by bee stings, anti-allergic treatment should be given, such as antihistamines and glucocorticoids, to reduce systemic symptoms and further damage of allergic reactions to the kidney. Anti-infective treatment: If the patient shows signs of infection, anti-infective treatment should be given to prevent increased damage to the kidney. Antidotes therapy: According to the type and nature of bee venom, the corresponding antidote can be considered to neutralize or eliminate the residual poison in the body.5. Nutrition support and diet management Nutrition support: For patients with renal insufficiency, appropriate nutritional support, such as enteral or enteral or parenteral nutrition, is needed to maintain the normal metabolism and immune function of the body. Dietary management: limit protein intake, choose choiceQuality low protein diet to reduce kidney burden. At the same time, the control of water, salt intake, to prevent oedema and hypertension aggravation.6. Prevention and treatment of complications Cardiovascular complications: patients with renal insufficiency are prone to cardiovascular complications, such as arrhythmia, heart failure, etc. Therefore, cardiac function needs to be monitored closely and given preventive and therapeutic measures accordingly. Infectious complications: due to low immunity, prone to infection. Therefore, it is necessary to keep the patients mouth, skin and other parts clean and hygienic to prevent the occurrence of infection.7. Long-term management and follow-up Regular follow-up: Patients need to regularly follow up the changes in renal function and other related indicators after discharge, in order to timely adjust the treatment plan. Health education: to provide health education to patients and their families, to explain the causes of renal insufficiency, preventive measures and matters for attention, etc., to improve patients self-management ability and quality of life. In conclusion, the management strategy of renal dysfunction after bee stings needs to comprehensively consider the specific situation of patients and the severity of the disease, and take comprehensive therapeutic measures to control the disease, reduce symptoms, restore renal function and prevent the occurrence of complications. | The cause of secondary anemia after bee sting is mainly related to the toxin composition in bee venom and its effect on human body. The following is a detailed analysis of the causes and treatment methods: 1. The causes of secondary anemia after bee sting, the role of hemolytic toxin: bee venom contains a variety of toxins, including hemolytic toxin. The toxin is able to destroy the red cell membrane, causing red blood cells to rupture, causing hemolytic anemia. During the process of hemolysis, the hemoglobin in the red blood cells is released into the blood, which may further cause jaundice, hemoglobinuria and other symptoms. Allergic reactions: Some people are allergic to the components of bee venom. Allergic reactions may lead to systemic inflammatory reactions, including inflammatory reactions in the blood vessels. This inflammatory response may damage red blood cells or affect their genesis, which indirectly leading to anemia. Bone Myelosuppression: Some components of bee venom may inhibit hematopoiesis in the bone marrow, leading to reduced erythropoiesis and subsequent anemia. This condition is more common in severe bee stings or bee venom poisoning. Multiple organ function damage: bee sting may also cause liver, kidney and other multiple organ function damage. Damage to these organs may affect erythrocyte generation, metabolism and excretion, which indirectly leading to anemia.2. Emergency treatment: for patients with severe bee stings, emergency treatment should be carried out immediately, including removing residual sting from the stings, cleaning wounds, giving anti-allergy drugs, etc. At the same time, patients vital signs and condition changes should be closely monitored. Blood purification therapy: For patients with severe hemolytic symptoms and obvious hemoglobinuria, blood purification therapy, such as plasma exchange, can be considered. This treatment can quickly remove hemolytic products and toxins in the body, reducing the hemolytic response and kidney burden. Glucocorticoid therapy: Glucocorticoids have anti-inflammatory, anti-allergic and immunosuppressive effects, and can reduce the damage of systemic inflammatory reactions and allergic reactions to red blood cells. Therefore, the application of glucocorticoids is also important in the treatment of secondary anemia after bee stings. Supportive therapy: In patients with severe anemia, supportive therapy such as blood transfusion should be given to correct the anemia status and improve patient outcomes. At the same time, attention should also be paid to maintain the hydroelectrolysisMass balance and nutritional support, etc. Prevention of complications: in the course of treatment, the patients condition changes should be closely monitored to prevent the occurrence of complications. In particular, attention should be paid to preventing serious complications such as infection, liver and kidney failure. Psychological support: patients with secondary anemia after bee stings may have anxiety, fear and other negative emotions due to their severe illness. Therefore, psychological support should also be given to patients in the process of treatment to help them build up the confidence to overcome the disease. In conclusion, the causes of secondary anemia after bee stings are complex and diverse, and the treatment methods should be individualized according to the specific situation of the patients. In the course of treatment, patients should be closely monitored for disease changes and prevent the occurrence of complications. | After bee sting, blood purification therapy is an important treatment to help remove toxins and metabolites from the body, protect vital organ functions, and reduce the risk of complications. The following is a bee sting after common blood purification treatment and its selection principle: 1. The blood purification treatment (HP) principle: the blood from the body, through the extracorporeal circulation system, using the perfusion adsorbent adsorption in blood poison, drugs and metabolites, achieve the purpose of purify the blood. Application: suitable for the early removal of bee sting toxins, especially for the removal of small and medium molecular toxins. Principle of plasma exchange (PE): The patients blood is drawn, the plasma and cellular components are separated by centrifugation or plasma separator, then the plasma containing toxin is removed, and the equal amount of fresh plasma or plasma substitutes (such as albumin, immunoglobulin, etc.) are returned back to the patient. Application: Suitable for patients with severe bee stings, especially when bee venom causes increased macromolecular toxin, intravascular hemolysis, impaired liver function, or severe immune response. Principle of continuous renal replacement therapy (CRRT): By simulating the filtration and reabsorption function of the kidney, we can continuously and slowly remove water, solutes and toxins from the body, while correcting electrolyte and acid-base balance disorders. Applicable: It is suitable for patients with renal dysfunction, acid-base electrolyte balance disorder after bee stings or need to maintain a stable internal environment for a long time. In some cases, combination therapy may require a combination of the above-mentioned blood purification methods to form heterozygous blood purification therapy in order to more fully remove toxins and protect organ function.2. Selection principle condition assessment: first of all, the patients condition needs to be comprehensively evaluated, including the type of toxin, the dose, the way to enter the body, the patients age, basic diseases, complications and other factors. Treatment objective: clear treatment objective, that is, remove toxins in the body as soon as possible, protect the function of important organs, reduce the risk of complications, etc. Individualized selection: according to the specific situation of the patient and the condition evaluation results, choose the most suitable blood purification treatment. For example, for the early stageFor patients with bee stings, plasma replacement or combination therapy may be required for severe patients or in cases of increased macromolecular toxins. Comprehensive treatment: blood purification treatment is only a part of the comprehensive treatment of bee stings, but also needs to combine other treatment means, such as anti-allergy treatment, anti-infection treatment, nutritional support, etc., to comprehensively improve the treatment effect. Dynamic adjustment: In the course of treatment, the patient needs to closely monitor the patients condition changes and treatment response, and dynamically adjust the treatment plan as needed to ensure the effectiveness and safety of the treatment. In conclusion, the choice of blood purification treatment after bee sting should be individualized according to the specific situation of patients and the results of disease evaluation, and follow the original of comprehensive treatment and dynamic adjustment. |
|  | Severity Assessment | Severity Assessment | Severity Assessment | Severity Assessment | Severity Assessment |
| Question | What symptoms after the bee sting suggest that the condition is serious? | How to assess the risk of multiple-organ function damage after a bee sting? | How should bee sting patients respond when appearing soy sauce color urine? | What is the significance of the neurological symptoms in bee sting patients? | How to judge whether bee sting patients need hospitalization? |
| Answer Engine 1 (ERNIE Bot 3.5) | After bee stings, the severity of the disease can usually be judged by a series of symptoms. Here are some common symptoms of severe illness: 1. Systemic allergic reaction: Urticaria: large red, swollen, itchy patches on the skin. Larrox edema: swelling in the throat that may cause dyspnea or suffocation. Wheeze, shortness of breath: shortness of breath, possibly accompanied by wheezing. Bronchiospasm: airway stenosis, resulting in respiratory obstruction. In severe cases, anaphylactic shock may even occur, manifested as a blood pressure drop, pale face, cold sweat, cold limbs, restlessness, and may even be life-threatening.2. Systemic poisoning symptoms: fever: the body temperature increases significantly. Headache, vomiting, abdominal pain, diarrhea: These symptoms may occur with systemic poisoning. Ingety, muscle spasm: the nervous system is affected by toxins, hyperactivity or spasm state. Severe patients may also have coma, shock, pulmonary edema and other serious conditions, and even lead to acute kidney failure or multiple organ failure.3. Local symptoms worsening: Although local redness and pain are common symptoms of bee stings, if the symptoms continue to worsen, such as the expansion of the swelling and the scope of pain is unbearable, especially when multiple stung, it may lead to a large area of obvious edema and severe pain, which also indicates that the disease is serious.4. Sings in special parts: If it is stung around the eye, it may cause high eyelid swelling and affect vision. Lip stings may cause significant swelling or wind in the mouth.5. Other symptoms: Some patients may also appear palpitations, palpitation and other heart discomfort symptoms. For wasp stings, there may also be hemolysis, hemoglobinuria, which may lead to renal failure in severe cases. In conclusion, the changing symptoms of patients should be closely observed after bee stings, especially the appearance of the above symptoms. Once there are signs of serious illness, seek medical advice immediately to avoid delay in treatment. For people with allergic constitution, more need to be vigilant to avoid serious allergic reactions. | Assthe risk of multiple organ function impairment (MODS) after bee sting is a comprehensive process involving multiple considerations. The following are some main assessment methods and risk factors: 1. Clinical performance assessment of systemic symptoms: observe whether the patient has high fever, chills, fatigue, nausea, vomiting, headache, irritability and other systemic poisoning symptoms. The appearance of these symptoms may indicate a severe condition with a risk of developing MODS. Local symptoms: check the redness, pain and blisters of the stings. A rapid expansion of redness, unbearable pain, or multiple stings can increase the risk of MODS. Organ-specific symptoms: nervous system: observe whether the patient is conscious disorder, convulsions, delusion and other neurological symptoms. Respiratory system: pay attention to whether the patient has shortness of breath, dyspnea, laryngeal edema and other respiratory symptoms. Circulation system: monitor the patients heart rate, blood pressure and other vital signs, and observe whether there are circulatory symptoms such as palpitations, chest tightness and chest pain. Digestive system: To evaluate the patient for nausea, vomiting, abdominal pain, diarrhea, jaundice and other digestive system symptoms. Urological system: pay attention to the patients urine volume, urine color, whether oliguria, anuria or hematuria and other symptoms.2. Laboratory examination and evaluation of blood routine: check the white blood cell count (WBC), hemoglobin (Hb), platelet (PLT) and other indicators. Elevated WBC may indicate infection or inflammatory response; decreased Hb may indicate anemia or hemolysis; and decreased PLT may indicate bleeding tendency. Liver and renal function tests: monitor alanine aminase (ALT), alanine transaminase (AST), total bilirubin (TBIL), direct bilirubin (DBIL), blood creatinine (SCr), urea nitrogen (BUN) and other indicators. The abnormality of these indicators may suggest an impaired liver and kidney function. Myocardial injury markers: such as cardiac troponin I (cTnI), creatine kinase isoenzyme (CK-MB), which are used to assess whether cardiac function is impaired. Coagulation tests: including prothrombin time (PT), activated partial thromboplastin time (APTT), etc., to assess the coagulation status of the patients.3. Risk assessment factors Type and number of stings: 10 swarm bee stings or stings, significantly increasing the risk of MODS (source: CNKI space）。Early manifestations after stings: such as urine volume of 400ml within 6 hours after stings, soy sauce colored urine, etc., which also indicates that the condition is serious and has the possibility of MODS (source: CNKI space). Age and basic diseases: older patients with basic diseases (such as hypertension, diabetes, kidney disease, etc.) have weak resistance and are more likely to develop MODS. Anaphylaxis: Patients allergic to bee venom may cause a severe allergic reaction after a second sting, thus increasing the risk of MODS.4. Comprehensive assessment combines the patients clinical performance, laboratory examination results and risk assessment factors. For high-risk patients, early interventions, such as intensive monitoring, active fluid rehydration, use of antiallergy drugs, and blood purification therapy, should be taken to reduce the incidence and death of MODS. | When bee sting patients appear with soy sauce color urine, it should be regarded as a signal of serious illness, and a series of coping measures should be taken immediately.1. Emergency medical treatment immediately: soy sauce urine may be a sign of kidney injury, hemolytic anemia, drug side effects or allergic reactions, which needs to be evaluated and treated by a professional doctor. Medical history: inform the doctor of the process, time, location and other discomfort symptoms when seeking a doctor.2. Initial treatment: patients and family members should keep calm to avoid aggravation of illness caused by excessive strain. Observe vital signs: pay attention to the patients consciousness, heart rate, breathing and other vital signs, and report to the doctor in time.3. Professional treatment to assess renal function: conduct routine urine and renal function tests to assess the degree of kidney damage. According to the examination results, develop a targeted treatment plan. Treatment of kidney injury: if the diagnosis is kidney injury, diuretics (such as furosemide, bumetanide) and other drugs can be used according to the doctors advice to promote urine discharge and reduce the burden on the kidney. In patients with severe kidney injury, blood purification therapy (such as hemodialysis, plasma exchange, etc.) may be required to remove toxins and metabolites from the body. Treatment of hemolytic anemia: in case of hemolytic anemia, blood transfusion and immunosuppressive therapy (such as intravenous human immunoglobulin, cyclosporin A, etc.) should be administered according to the severity of the condition. At the same time, hemoglobin, hematocrit and other indicators should be closely monitored to assess the improvement of anemia. Antiallergic treatment: If it is determined to be caused by allergic reaction, antiallergic drugs (such as cetirizine hydrochloride tablets, loratadine tablets, etc.) should be used in time to relieve allergic symptoms. For patients with severe allergic reactions, emergency drugs such as adrenaline may be required for rescue. Supportive treatment: Maintain the patients water and electrolyte balance and correct the acid-base imbalance. Give nutritional support to enhance the patients physique. Prevention of complications such as infection.4. Follow-up observation and nursing closely observe the changes of the condition: pay attention to the changes of patients urine color and urine volume, and whether there are new uncomfortable symptoms. Regular review: regular review of urine routine, renal function and other indicators,To evaluate the treatment effect and the disease recovery. Keep the wound clean: clean and disinfect the bee stings to avoid infection. In short, patients with bee stings should seek medical treatment and receive professional treatment when they have soy sauce colored urine. In the process of treatment, we should closely observe the changes of the condition and take corresponding nursing measures to promote health. | The significance of neurological symptoms in patients with bee stings is great. It is not only a direct manifestation of bee venom action on the human body, but it also may indicate the severity of the disease and the risk of multiple organ function damage. The following is a detailed analysis of this: 1. The direct significance of the nervous system symptoms of bee venom toxic effects: bee venom contains a variety of bioactive components, such as peptides, enzymes, amines, etc., which have a certain toxic effects on the nervous system. Therefore, the appearance of neurological symptoms is one of the direct manifestations of bee venom poisoning. Indicator of disease severity: as one of the important systems of the nervous body, its damage can often reflect the severity of the condition. Neurological symptoms in bee sting patients, such as headache, dizziness, consciousness disorders, convulsions, etc., may mean that bee venom has caused more serious damage to the nervous system.2. Risk of multiple organ function damage A precursor of multiple organ dysfunction syndrome (MODS): After bee sting, toxin diffusion in the body may lead to impaired function of multiple organs, forming MODS. The appearance of neurological symptoms often indicates that other organs may also be damaged to varying degrees. For example, bee venom may induce encephalitis, cerebrovascular accidents and other neurological diseases, but also may cause damage to the respiratory system, circulatory system, digestive system and other diseases. Key to early detection of complications: The onset of neurological symptoms helps doctors to detect complications early and take corresponding treatment measures. By closely observing the patients neurological symptoms, doctors can adjust the treatment plan in time and reduce the incidence of complications and mortality.3. Specific neurological symptoms and their meaning Headache and dizziness: it may be caused by the direct stimulation effect of bee venom on the nervous system, or it may be the performance of increased intracranial pressure. The appearance of these symptoms suggests that patients may need further examination and treatment. Disorder of consciousness: including drowsiness, coma and other states, is a serious manifestation of nervous system damage. The emergence of consciousness disorders often means that the disease has developed to a more serious stage and requires immediate treatment. Titching: may be caused by neurotransmitter imbalance or damaged brain areas. The onset of tics can not only aggravate the damage to the nervous system, May also cause adverse effects on the respiratory system, circulatory system, etc.4. Seek Medical advice immediately: When bee sting patients develop neurological symptoms, they should seek medical advice immediately and inform the doctor of the relevant medical history and symptoms. Closely observe the changes in the condition: during and after medical treatment, patients should closely observe the changes in their condition, especially the changes of neurological symptoms. Actively cooperate with treatment: Conduct corresponding examination and treatment according to the doctors advice, including drug treatment, blood purification treatment, etc. At the same time, maintaining a good attitude and adequate sleep are also crucial to the recovery. In conclusion, the occurrence of neurological symptoms in bee sting patients has great significance, which is not only a direct manifestation of bee venom poisoning, but also may indicate the severity of the disease and the risk of multiple organ function damage. Therefore, when the relevant symptoms should be timely to seek medical treatment and take effective treatment measures. | To determine whether bee stings need hospitalization, multiple factors usually need to be considered, including the patients symptoms, stings, number of stings, history of allergies, and underlying health conditions. The following are some specific criteria: 1. Symptoms systemic symptoms: If the patient has high fever, chills, fatigue, nausea, vomiting, headache, irritability and other systemic poisoning symptoms, especially when these symptoms continue to worsen or are difficult to relieve, the patient should be highly suspected as serious and may need hospitalization. Nervous system symptoms: such as headache, dizziness, consciousness disorders, convulsions and other neurological symptoms, often indicate the severity of the disease and the risk of multiple organ function damage, need close observation and timely treatment. Local symptoms: Although local redness and pain are common symptoms of bee stings, if the scope of swelling expands rapidly, the pain is unbearable, or multiple stings occur, it may increase the complexity of the disease and the difficulty of treatment, and need to be hospitalized for observation and treatment.2. Stings: If the sting is located near important organs (such as head, neck, chest, etc.) or special sites (such as eyes, lips, etc.), more caution and hospitalization may be considered to prevent complications. Number of stings: In general, 10 stings significantly increase the severity of the disease and the risk of multiple organ damage, so such patients usually require hospitalization.3. Allergy history and basic health status Allergy history: If the patient has a history of bee venom allergy or allergic reactions to other substances, it is more likely to have serious allergic reactions and complications after bee stings, so close observation and timely treatment are needed, and hospitalization if necessary. Basic health status: The patients age, underlying diseases (such as hypertension, diabetes, kidney disease, etc.), and immune status will all affect their tolerance to bee venom and the development of the disease, so doctors will consider these factors when determining whether they need hospitalization.4. Treatment response to other factors: If the patients symptoms do not relieve or worsen after receiving initial treatment, or new symptoms of discomfort appear, the treatment plan should be adjusted and tested in timeIn-patient treatment. Medical resources: In some cases, hospitalization may be required to ensure safe and effective treatment if the patient is unable to manage the wound and observe changes in the condition. In conclusion, it is a comprehensive process to determine whether bee stings patients need hospitalization. The doctor will evaluate and judge according to the specific situation of the patient, and make the corresponding treatment plan. For patients who need hospitalization, hospitalization should be arranged in time and closely observed and treated to prevent the occurrence of complications and the evil of the disease. |
|  | Special Population Management | Special Population Management | Special Population Management | Special Population Management | Special Population Management |
| Question | How can children be specially treated after bee stings? | What are the special nursing measures after bee stings in elderly patients? | What is the emergency treatment strategy after the pregnant mother bee sting? | What should patients with chronic diseases pay attention to after bee stings? | What is the processing point of bee sting after immune deficiency person? |
| Answer Engine 1 (ERNIE Bot 3.5) | After a child is stung by bees, a series of special treatment measures are needed to ensure the safety of the child and reduce the symptoms. The following are the detailed treatment steps: 1. Emergency treatment to remove the sting: first, carefully check the wound, if found that the bee sting is still in the skin, should use tweezers to gently pull it out as soon as possible. Be careful to avoid squeezing the sting to avoid injecting more venom into the body. If there are no tweezers, you can also use the tip of a sterilized needle. Wash the wound: repeatedly rinse the wound with running water or soap to remove venom and dirt from the wound surface. The lush should last for more than 5 minutes to ensure thorough cleaning. If conditions permit, you can also use soda water to wash the wounds, because the bee venom is acidic, while soda water can neutralize acid and help relieve symptoms.2. Local treatment on ice: ice the wound with ice or ice packs for 15-20 minutes, and the next ice after some time. Ice compress can contract blood vessels and reduce the exudation of inflammatory substances, thus reducing redness and pain. Disinfection: After washing the wound, disinfect the wound with iodophor or alcohol. These disinfectants can kill bacteria and viruses on the surface of the wound and prevent infection. Apply drugs: if the wound appears swelling, pain and other symptoms, you can follow the doctors advice to apply an appropriate amount of anti-inflammatory ointment, such as erythromycin ointment, mupirocin ointment, etc. These drugs have anti-inflammatory and antibacterial effects, which can help to reduce symptoms and promote wound healing.3. Observation and close observation for medical treatment: After initial treatment, the symptom changes of children should be closely observed. If symptoms such as redness and pain continue to worsen, or new symptoms (such as dyspnea, chest tightness, palpitations, etc.) appear, seek medical attention immediately. Timely medical attention: If the child has severe allergic reactions (such as systemic rash, breathing difficulties, shock, etc.), call the emergency number immediately and send it to the hospital for treatment. In the process of sending to the hospital, the childs respiratory tract should be kept unobstructed to avoid secondary injuries.4. Avoid scratching: warn children not to scratch the wound, so as not to aggravate the infection or leave scars.bland diet: During the recovery period, children should be provided with light and easily digestible food to avoid spicy and stimulating food intake, so as not to aggravate the symptoms or affect the wound healing. Keep rest: let children keep enough rest and sleep, help the body recovery and immunity. In conclusion, children should be treated urgently and locally immediately after bee sting, and observe symptom changes closely. If necessary, seek medical advice promptly and follow the doctors advice. | Special care measures for elderly patients after bee stings needs to comprehensively consider the patients age, underlying health status, and the severity of bee stings. Here are some key care measures: 1. Emergency treatment to remove the sting: Check the wound carefully to make sure there are no remaining left. If bee spines are found, gently use tweezers or cards to avoid hard squeezing to prevent further venom injection. Local cleaning: Wash the wound with soapy water or a weak alkaline solution (such as sodium bicarbonate solution) to neutralize the acidic component of bee venom and reduce the skin irritation of the venom.2. Local care cold compress: after cleaning the wound, cold compress can be made with a cold towel or ice bag to reduce local swelling and pain. Cold compress time should not be too long, 15-20 minutes each time, to avoid frostbite skin. Drug application: Apply an appropriate amount of anti-inflammatory ointment or anti-allergy ointment to relieve local inflammation and allergic reactions. If the swelling and pain is serious, consider using cupping and other TCM external treatment methods to discharge venom.3. Systemic observation and nursing observation of vital signs: closely monitor the patients vital signs, including blood pressure, heart rate, breathing, etc., so as to detect and deal with possible complications in time. Antiallergy treatment: If the patient has allergic reactions (such as rash, itching, dyspnea, etc.), the antiallergy treatment should be given immediately. Mild allergies can include oral antihistamines (such as loratadine, cetirizine, etc.), and severe allergies can require emergency drugs such as epinephrine. Rehydration and nutritional support: provide appropriate rehydration and nutritional support. Elderly patients are weak and prone to dehydration and malnutrition, so special attention needs to be paid to supplement enough water and nutrients. Infection Prevention: Keep the wound clean and dry to avoid secondary infection. If the wound appears redness, purulent and other infection, antibiotics should be given timely treatment.4. Rest and activities Bed rest: after bee stings, patients should try to rest in bed to avoid strenuous exercise and overwork, so as not to aggravate symptoms or induce complications. Appropriate activities: when the condition is stable, mild activities can be appropriate, such as walking, Taijiquan, to promote blood circulation and the bodyrepeated. However, attention should be paid to avoid overwork and compression of the injured areas.5. Psychological nursing to comfort emotions: elderly patients have poor tolerance to pain, and are prone to anxiety, fear and other negative emotions due to worry about the condition. Therefore, medical staff and family members should give patients enough comfort and care to help them maintain emotional stability. Explain the condition: explain the condition and treatment plan to the patients in detail, so that they can understand their condition and treatment progress, so as to enhance the confidence and cooperation of treatment. In conclusion, the special nursing measures for elderly patients after bee stings include emergency treatment, local nursing, systemic observation and nursing, rest and activities, and psychological nursing care. Through comprehensive measures, it can effectively reduce the symptoms of patients, prevent the occurrence of complications and promote the early health of patients. | Emergency management strategies for pregnant women after bee stings are crucial to ensure maternal and infant safety. The following are a series of detailed emergency treatment steps: 1. Check the wound immediately and remove the sting to check the wound: First, quickly check the sting site to confirm whether there are any bee sting. Remove the sting: If the sting is found, remove it gently using forceps or the tip of a sterilized needle. Be careful to avoid hard squeezing to avoid further venom injection into the body. If there are no tweezers, you can also gently stick or scrape the poison with tape or a hard card (such as ID card or bank card).2. Clean the wound with an alkaline solution: because the venom of bees is acidic, an alkaline solution such as soapy water, soda water or aloe juice should be used to clean the wound. These solutions can neutralize the venom and reduce the pain and redness. Water rinse: If there is no above alkaline solution, you can also use water to wash the wound repeatedly to remove venom and dirt from the surface.3. Local cold compress cold compress: cold compress the wound in time, use cold towel or ice bag, apply 15-20 minutes each time, after a period of time. Cold compress can contract blood vessels and reduce the exudation of inflammatory substances, thereby reducing swelling and pain.4. Observe symptoms and seek medical observation: Pregnant women should closely observe their own symptoms, including local redness, pain, itching and systemic symptoms such as dizziness, dyspnea, nausea and vomiting, etc. These symptoms may indicate the occurrence of an allergic reaction. Seek medical attention immediately: If obvious allergic reactions occur or the symptoms continue to worsen, go to the hospital immediately. In the process of medical treatment, the pregnant woman should inform the doctor of her pregnancy, so that the doctor can take appropriate treatment measures.5. Avoid self-medication with caution: Pregnant women should avoid self-use of drugs during pregnancy, especially oral drugs. Because some drugs may have adverse effects on the fetus. If a medication is needed, it should be done under the guidance of a doctor.6. Prevention measures: In order to avoid repeated bee stings, pregnant women should avoid going to gardens, woodlands or mountainous areas where bees occur during pregnancy. If you must go, wear long sleeves and trousers, avoid wearing bright clothes andApply perfume and other items that may attract bees. In conclusion, emergency management strategies for pregnant women after bee sting include immediate examination of the wound and removal of the sting, cleaning the wound with alkaline solution, local cold compress, close observation of symptoms and timely medical attention, and avoidance of self-medication. At the same time, pregnant women should also take preventive measures to reduce the wind of the sting again. | Patients with chronic diseases after bee sting, need to pay special attention to the following aspects, to ensure the stability of the condition and timely and effective treatment: 1. Emergency treatment and wound care quickly remove sting: use tweezers or disinfection needle tip gently pull out the poison in the wound, avoid hard extrusion, to prevent further spread of venom. Clean the wound: choose the appropriate cleaning solution according to the bee species. The venom of bees is acidic and can be cleaned with weak alkaline liquid such as soapy water and 5% sodium bicarbonate solution; while the venom of wasps is alkaline, it should be cleaned with weak acidic liquid such as vinegar. Wash thoroughly to ensure the venom and dirt from the wound surface. Local cold compress: Use a cold towel or ice pack to cool the wound to reduce swelling and pain. But pay attention to the cold compress time should not be too long, so as to avoid frostbite skin.2. Observe the changes in the disease systemic symptoms monitoring: closely observe the patients systemic symptoms, such as dizziness, nausea, vomiting, dyspnea, palpitations, etc., which may indicate the occurrence of allergic reactions or poisoning symptoms. Special attention is paid to whether there are aggravation or changes in chronic disease-related symptoms. Local symptom observation: pay attention to whether the redness, pain, itching and other symptoms around the wound are gradually relieved or aggravated. If symptoms of local infection (such as pus exudation, swelling, etc.), you should seek medical treatment in time.3. Timely medical treatment and inform the condition timely medical treatment: If the patient has obvious allergic reaction or poisoning symptoms, he should go to the hospital immediately. During the treatment, try to stay calm and describe the symptoms and symptoms of bee stings. Inform the condition: inform the doctor of your history of chronic disease and current medication, so that the doctor can comprehensively consider and develop an appropriate treatment plan. If allergy or detoxifying drugs are needed, tell your doctor which drugs you are allergic to or which drugs are indicated.4. Avoid self-medication Patients with chronic diseases should avoid self-medication for treatment after bee sting. Because some drugs may interact with therapeutic drugs for chronic diseases, affecting efficacy or increasing side effects. Therefore, before and in the process of seeking medical treatment, we should strictly follow the guidance of doctors for medication.5. Psychological support and comfort After bee stings, patients may feel anxiety and fear because of pain and discomfort. Especially for patients with chronic diseases, this mood may be even more severe. Therefore, family members and medical staff should give patients enough psychological support and comfort to help them maintain emotional stability and actively cooperate with the treatment. To sum up, patients with chronic diseases need to pay special attention to emergency treatment and wound care, observation of changes in the condition, timely medical treatment and notification of the illness, avoid self-medication, and psychological support and comfort after being stung. Through comprehensive measures and timely and effective treatment, the pain of patients can be minimized and their safety guaranteed. | Immune compromised people should be treated more carefully and timely because their body is less resistant to toxins. The following are the treatment points: 1. Emergency treatment quickly remove the poison: use tweezers or cards to gently scrape or remove the poison in the wound, avoid squeezing, to prevent further spread of the venom. If it is impossible to determine whether there is residual sting or cannot be handled yourself, seek medical attention. Clean the wound: choose the appropriate cleaning solution according to the bee species. The venom of bees is acidic and can be cleaned with weak alkaline liquid such as soapy water and 5% sodium bicarbonate solution; while the venom of wasps is alkaline, it should be cleaned with weak acidic liquid such as vinegar. Wash thoroughly to ensure the venom and dirt from the wound surface.2. Observation and monitoring Close observation of symptoms: immunocompromised people are more prone to allergic reactions and infection complications after bee sting. Therefore, it is necessary to closely observe the patients symptom changes, including local redness, pain, itching, and systemic symptoms such as dizziness, nausea, vomiting, dyspnea, etc. In case of allergic reactions or signs of infection. Monitoring vital signs: For patients with severe stings or systemic symptoms, vital signs, such as blood pressure, heart rate, breathing, etc., should be monitored so as to detect and handle serious conditions such as shock possible.3. Medical treatment timely medical treatment: After ststing, it is recommended to seek medical treatment in time regardless of the severity of the symptoms. The doctor will make the appropriate treatment plan according to the specific situation of the patient. In the process of medical treatment, to inform the doctor of their own immune dysfunction and bee stings. Anti-allergic treatment: In the event of an allergic reaction, such as rash, dyspnea, etc., the doctor may give anti-allergic treatment, such as oral administration or injection of anti-allergic drugs. Anti-infective treatment: If there are signs of infection, such as swelling of the wound, exudation of pus, the doctor may give anti-infective treatment, such as the use of antibiotics.4. Prevention and precautions Prevention of repeated sting: people with low immune function should try to avoid going to bees and other insects in their daily life. If you must go, wear long sleeves, trousers and shoes to avoid themUse items such as perfume that might attract insects. Enhance immunity: immunocompromised people should enhance their immunity through proper diet, proper exercise and adequate rest to improve the bodys resistance to toxins. Pay attention to medication safety: in the process of medical treatment, the doctor should be informed of his medication in detail to avoid the interaction between drugs affecting the treatment effect. In conclusion, the key points of treatment of immunocompromised patients after bee sting include emergency treatment, observation and monitoring, medical treatment, prevention and matters needing attention. Through timely and effective treatment and monitoring, patients can minimize their pain and ensure their life safety. |
|  | Medication Treatment | Medication Treatment | Medication Treatment | Medication Treatment | Medication Treatment |
| Question | What is the indication that uses antibiotic after bee sting? | What are the commonly used analgesic drugs after bee sting? | What is the basis of the choice of antihistamine drugs after bee sting? | What is the principle of glucocorticoid use after bee sting? How to choose the dose? | How to undertake fill fluid treatment after bee sting? What are the precautions? |
| Answer Engine 1 (ERNIE Bot 3.5) | The indications for antibiotic use after bee stings mainly include the following aspects: 1. Local infection wound redness and swelling intensifies: if there is obvious swelling around the wound after bee stings, and the range of swelling gradually expands, this may be the manifestation of local infection. Pus exudation: If there is pus exudation at the wound, bacterial infection has occurred and antibiotics are needed at this time. Increasing pain: If the wound becomes worse and increases with a burning sensation, it may also be a symptom of infection.2. Symptoms of systemic infection fever: after bee sting, if fever occurs and the body temperature continues to rise, it may be a systemic infection reaction caused by bacterial toxins. Lymph node enlargement: When the infection is severe, it may cause enlargement and pain in the surrounding lymph nodes. Other symptoms: such as nausea, vomiting, headache, fatigue and other general discomfort symptoms, may also be the manifestation of infection.3. Immune dysfunction of high-risk groups: such as the elderly, children, patients with chronic diseases, etc., they are more likely to be infected by bee sting, so prophylactic antibiotics are needed. Allergic constitution: people who are allergic to bee venom may have severe allergic reaction after bee stings. At this time, the use of antibiotics can assist treatment, control infection and reduce allergic reaction.4 Doctors recommend that before taking antibiotics, the most important thing is to consult a doctors advice. The doctor will determine whether antibiotics are needed based on the patients specific conditions (such as wound condition, physical condition, allergy history, etc.), and choose the appropriate type and dose of antibiotics. The commonly used antibiotic species amoxicillin: belongs to penicillin antibiotics, has the characteristics of strong bactericidal effect, strong ability to penetrate the cell membrane. It has a strong bacteriostatic and bactericidal effect on most pathogenic G + bacteria and G-bacteria (including cocci and bacilli). Cefixime: a third-generation cephalosporin antibiotic for oral use, suitable for the treatment of respiratory, urinary and biliary tract infections caused by sensitive bacteria. Levofloxacin: belongs to quinolone antibiotics, with broad spectrum antibacterial effect, strong antibacterial effect, on most of enterobacteriaceae, such as Escherichia coli, Klebsiella, Proteus, Salmonella, Shigella and Gram-negative bacteria such as Haemophilus influenzae, Legionella pneumophila, and Neisseria gonorrhoeae have strong antibacterial activity. | The commonly used analgesic drugs after bee stings mainly include the following types: 1. ibuprofen sustained-release capsule effect: ibuprofen sustained-release capsule is a nonsteroidal anti-inflammatory drug (NSAID), which plays an analgesic effect by inhibiting the synthesis of prostaglandins in the body. It can effectively relieve the local pain caused by bee stings, especially when the pain is more severe. Usage: Oral administration according to the instructions or the instructions.2. Characteristics of profene extended-release tablets: extended-release tablets are a compound composed of ibuprofen and codeine phosphate, in which ibuprofen is a non-steroidal anti-inflammatory drug with analgesic, antipyretic and anti-inflammatory effects; codeine phosphate is a central analgesic with analgesic and sedative effects. Both are used together, and the analgesic effect is strengthened. Application: It is suitable for patients with severe pain after bee sting, but it should pay attention to its addiction and other potential side effects.3. In addition to ibuprofen sustained-release capsules, other NSAIDs such as indomeacin and diclofenac sodium can also be used to relieve pain after bee stings. The mechanism of action of these drugs is similar to that of ibuprofen and both exert analgesic effects by inhibiting the synthesis of prostaglandin. Precautions for use: Follow the doctors advice: When using analgesic drugs, always use them according to the doctors instructions or the dose listed in the instructions to avoid adverse reactions caused by excessive or improper use. Observation reaction: After the use of analgesic drugs, the patients reaction should be closely observed. If the patient is not suitable, stop the drug in time and seek medical treatment. Comprehensive treatment: the treatment of bee stings is not only analgesia, but also other treatment measures such as cleaning the wound, pulling out the sting, preventing infection and so on. Therefore, other corresponding treatments are required along with the use of analgesic drugs. In conclusion, commonly used analgesic drugs after bee stings include NSAIDs such as ibuprofen sustained-release capsules and profenine sustained-release tablets. When using these drugs, follow the instructions or the instructions, and observe the patients response. At the same time, a comprehensive treatment should also be combined with other treatment measures. | The selection of antihistamines after bee stings is mainly based on the following aspects: 1. Degree of allergic reaction mild allergic reaction: If only mild allergic symptoms such as local redness and itching appear after bee stings, oral antihistamines can be chosen for relief. These drugs can quickly block the histamine receptors and reduce the symptoms caused by allergic reactions. Severe allergic reaction: If a severe allergic reaction occurs after bee stings, such as generalized rash, breathing difficulties, laryngeal edema, etc., seek medical attention immediately and more potent anti-allergy drugs or other emergency measures may be required.2. Types and characteristics of drugs: first-generation antihistamines, such as paramine, diphenhydramine, etc. Although these drugs have anti-allergy effects, they are often accompanied by obvious central inhibition, which may cause adverse reactions such as drowsiness and fatigue. Therefore, first-generation antihistamines should be used with caution when staying awake (such as driving, operating machinery, etc.). The second generation of antihistamines: such as cetirizine and loratadine, these drugs have the characteristics of not easy to cross the blood-brain barrier, little sedative effect, almost no anticholine effect, and a long time, reduce the number of use, the dose is relatively small. Therefore, second-generation antihistamines have gradually become the drug of choice for the treatment of allergic reactions such as bee stings.3. Patient specific conditions Age and weight: The response to antihistamine drugs may vary in patients at different ages and weight, so the patient specific situation needs to be considered when choosing drugs. For example, for pediatric patients, drugs with higher safety and less side effects should be selected and dosage adjusted by body weight. Underlying disease and medication history: If patients have an underlying disease or are using other drugs, special attention is paid to the interactions between drugs and potential adverse reactions. When choosing antihistamines, consult a doctor or pharmacist for advice to avoid adverse interactions with other drugs.4. Use used: Antihistamines are usually given orally and the dose according to the order. In the process of use, we should pay attention to follow the doctors advice, take the medicine on time and in the dosage, and avoid increasing or decreasing the dose or stopping the medicine by ourselves. Note: MakeDuring the use of antihistamines, attention should be paid to the reaction of the patient. If there is not suitable, stop the drug in time and seek medical treatment. At the same time, concurrent use with CNS inhibitors such as alcohol should be avoided to increase the risk of adverse reactions. To sum up, the selection of antihistamines after bee stings mainly includes the degree of allergic reaction, the types and characteristics of drugs, the specific situation of patients, and the use of drugs and matters needing attention. When choosing drugs, the above factors should be considered comprehensively and reasonably made under the guidance of doctors. | The use principles and dosage selection of glucocorticoids after bee stings are as follows: 1. Early application: After bee stings, if patients have obvious allergic reactions or poisoning symptoms, glucocorticoids should be used as soon as possible to control the development of the disease. Sufficient application: according to the specific patients condition and physical condition, select the appropriate dose for adequate treatment, to ensure that the drug can play a full role. Gradually reduction: after the condition is controlled, the dose of glucocorticoid should be gradually reduced to avoid adverse reactions caused by long-term heavy use. Individualized therapy: The response to glucocorticoids may vary between different patients, so it should be adjusted according to the specific situation of the patients during the use process. Combination drug: when necessary, it can be combined with other drugs such as antihistamines and antibiotics to enhance the therapeutic effect.2. Dose selection The dose selection of glucocorticoids should be comprehensively considered according to the patients age, weight, severity of disease and other factors. Here are some common dose options: Oral corticosteroids: prednisone acetate: Common doses of 40-60mg in single or gradually reduced doses. Specific dose should be adjusted according to patient condition and physician guidance. Other oral glucocorticoid drugs can also be used according to the needs of the disease, but attention should be paid to the control of dosage and medication time. Glucocorticosteroids for injection: In more severe patients, they may be required. Commonly used drugs include dexamethasone, hydrocortisone, etc. Dose selection should be determined according to the severity of patient condition and physician guidance. Generally, the initial dose may be large to control the condition quickly; the dose should be tapered and discontinued as it improves. It should be noted that although corticosteroids have strong anti-inflammatory, anti-allergic and immunosuppressive effects, long-term heavy use may lead to a series of adverse effects, such as infection, osteoporosis, elevated blood glucose, etc. Therefore, in the process of use, we should strictly follow the doctors advice, pay attention to the monitoring of patients condition changes and adverse reactions, and timely adjust the treatment plan. Moreover, in immunocompromised patients, when using glucocorticoidsMore caution should be taken to avoid further weakening the immune function and increase the risk of infection. At the same time, the immune status of patients should be closely monitored during the process of use, and the corresponding immune support treatment should be conducted as needed. | Rerehydration therapy after bee sting is one of the important measures to manage severe allergic reactions and maintain vital signs. The following are the specific steps and precautions of rehydration treatment: 1. Specific steps of rehydration treatment to evaluate the condition: First, the severity of the patients condition needs to be assessed, including the performance of allergic reactions, blood pressure, heart rate, breathing and other vital signs changes. If the patient has severe allergic reactions, such as dyspnea, laryngeal edema, blood pressure drop, or shock symptoms, such as pale face, wet and cold limbs, fine pulse, etc., fluid rehydration treatment should be performed immediately. Determine the amount of fluid replenishment: Generally speaking, the amount of fluid required is about 20-30 mL per kilogram of body weight. If the patient has severe hemodynamic disturbances, such as hypotension, shock, etc., an increase in rehydration volume may be required to 50 mL per kg of body weight. Select the rehydration types: the commonly used rehydration types include physiological saline, balanced salt solution, etc. These fluids can rapidly replenish blood volume, maintain a water and electrolyte balance and a stable internal environment. Implementation of rehydration: rehydration can be performed by oral or intravenous infusion. In patients with severe allergic reactions or shock, rapid fluid supplementation through intravenous fluids is usually required. In the process of rehydration, it is necessary to closely monitor the patients vital signs and reactions, and adjust the speed and amount of rehydration in time.2. Precautions for timely assessment of the condition: before and during the rehydration treatment, the severity of the patient and the change of vital signs should be constantly evaluated, so as to adjust the treatment plan in time. Reasonable determination of fluid replenishment amount: the determination of fluid replenishment amount needs to be adjusted individually according to the specific situation of patients, so as to avoid the adverse impact of too much or too little fluid replenishment amount on patients. Select the appropriate type: select the appropriate type according to the specific situation of the patient to ensure the maximum effect of rehydration. Close monitoring of vital signs: In the process of fluid rehydration, the vital signs and reactions should be closely monitored, including changes in blood pressure, heart rate, respiration and other indicators. Any abnormal situation should be dealt with in time. Note the infusion speed and volume: Control of the infusion speed and volume is crucial for the effect of rehydration therapy. Too fast or too slowThe speed of the infusion may adversely affect the patient. Therefore, it needs to be individualized adjustment according to the specific situation of patients and the suggestions of doctors. Comprehensive treatment: fluid rehydration treatment is only a part of the comprehensive treatment after bee stings. At the same time of rehydration treatment, comprehensive treatment needs to be conducted out anti-allergy and anti-infection according to the specific situation of the patient. Keep the wound clean: While rehydration treatment, attention should also be paid to keep the wound site clean and dry to avoid infection. In conclusion, fluid rehydration treatment after bee stings requires multiple factors, including the severity of the patients condition, changes in vital signs, and the doctors advice. Through reasonable rehydration therapy, it can effectively relieve the allergic reaction and shock symptoms, and maintain the life safety and health of patients. |
|  | Wound Treatment | Wound Treatment | Wound Treatment | Wound Treatment | Wound Treatment |
| Question | What are the characteristics of skin damage after bee sting and its clinical significance? | How to calculate the number of bee sting sting needles? | Do you need to remove the poison sting after a bee sting? | Is there a need for routine tetanus prevention after bee stings? | How does the cellulitis caused by bee sting handle? |
| Answer Engine 1 (ERNIE Bot 3.5) | The characteristics and clinical significance of skin damage after bee stings are as follows: 1. Characteristics of skin damage Local redness: after bee stings, redness usually occurs rapidly in the injured site. This is due to the release of inflammatory mediators such as histamine and serotonin in bee venom, which cause local vascular dilation and congestion. Pain and pruritus: burning and itching may occur immediately at the injured area. This pain and itching sensation may vary by individual differences but is usually one of the first symptoms after bee stings. Petechiae and blisters: If bee venom causes a strong allergic reaction, petechiae may occur in the injured area, even forming blisters or blood blisters. These blisters usually appear in areas of loose tissue, such as eyelids, lips, and may be associated with high edema. Special site damage: If stings occur in special sites, such as the eye or mouth, it may lead to more serious local damage. Ocular stings may lead to periorbital, conjunctival, corneal edema, extraocular muscle paralysis, anterior chamber congestion and even pyema, iritis, lens barrier changes, optic atrophy, etc. Intraoral stings may cause mucosal edema or even obstruction in the upper airway.2. Clinical significance judgment of injury: the extent and scope of skin damage can preliminarily judge the injury of bee stings. Generally, patients with mild symptoms of local redness, pain, and itching may have only mild local reactions; patients with blisters, skin necrosis or special site damage may have serious injuries and need timely medical treatment. Guide to treatment: Based on the characteristics of skin damage, doctors can choose the appropriate treatment method. For example, for patients with local redness and pain, cold compress and anti-allergy drugs can be used; and for patients with blisters and skin necrosis, debridement and dressing change are required. Prevention of complications: timely observation and treatment of skin damage can prevent possible complications after bee stings. For example, avoid infection by keeping the wound clean and dry, and by closely observing the patients vital signs and condition changes, detect and manage serious complications such as allergic reaction and shock. In conclusion, the characteristics of skin damage after bee sting have certain clinical significanceTo help doctors judge injuries, guide treatment and prevent complications. Therefore, after being stung by bees, you should timely seek medical attention and pay attention to observe the changes of skin damage. | The number of stings after bee stings is mainly based on the number of times the injured person is stung. Since bee stings often involve multiple stings, and each point may be caused by different numbers of bee needles, the following points should be considered in the calculation: 1. Direct observation and counting direct counting method: When the injured person seeks medical treatment, medical staff can count the obvious stings by directly observing the injured site. This method is suitable for ststing, small and easy to identify. Careful inspection: because some stings may be relatively hidden or small, medical staff need to carry out careful inspection to ensure that no leakage. This includes assisting observation with tools such as magnifying glasses, as well as asking the injured person about the specific location of pain or discomfort.2. Patient description: The injured person can usually describe the process of bee stings, including the number and approximate location of stings. This information is of important reference value for determining the number of sting needles. However, due to individual differences and factors such as vague memory.3. Clinical evaluation and diagnostic symptom evaluation: According to the symptoms of the injured person, such as pain, swelling, itching, etc., medical staff can initially assess the severity of stings and the number of stings that may be involved. However, it should be noted that these symptoms do not directly reflect the specific number of stings needles. Medical examination: When necessary, medical staff can further evaluate the injured persons condition and stings through medical examination methods (such as blood tests, allergen tests, etc.). These tests are helpful to understand whether the injured person has allergic reaction, infection and other complications, but do not directly determine the number of sting needles.4. Dealing with multiple stings in special circumstances: If the injured person is injured by multiple stings and the stings are scattered, the calculation of stings needles should be more careful and patient. Medical staff need to check each stings one by one and make cumulative counts. Repeated stings: In some cases, multiple stings may be found to the same site. At this point, the medical staff need to make a comprehensive judgment according to the shape, size and depth and other factors of the wound to determine the specific number of sting needles.5. Timely medical attention: no stingThe number of injured needles, the injured person should be timely to seek medical attention. Medical staff will make corresponding treatment plans according to the specific situation to reduce symptoms, prevent complications and promote recovery. Avoid self-treatment: the injured should avoid improper treatment methods such as removing the busting or applying unknown drugs. These behaviors may exacerbate symptoms or cause complications such as infection. In conclusion, the calculation of sting needles after bee stings needs to be combined with various factors such as direct observation, patient description, clinical evaluation and diagnosis. In practice, the medical staff will take corresponding measures to ensure the accuracy and reliability of the calculation. | After bee stings, they do need to be removed. This is because bees after the sting, the tail of the needle often stay in the persons skin, these needles may carry venom, if not removed in time, may lead to the spread of venom in the skin, aggravating local swelling, pain and other discomfort, and may even cause infection. The specific method to remove the sting is as follows: Use the tool: you can use tweezers or other sharp but not easily broken tools to gently clip the sting from the skin. During the operation process, attention should be paid to avoid excessive extrusion of the affected area, so as not to be squeezed into the body or aggravate the allergic reaction. Avoid free hand removal: Free hand sting removal may cause the sting to break in the skin due to unstable hands or improper force control, increasing the difficulty of subsequent treatment. Disinfection: the wound should be cleaned with water or soap water to neutralize the venom and reduce the risk of infection. If conditions permit, the wound can be further treated with disinfectants such as iodophor and alcohol. It should be noted that if there is a severe allergic reaction, such as dyspnea, laryngeal edema, etc., you should immediately seek a doctor and inform the doctor of the sting, so that the doctor can quickly take corresponding treatment measures. In addition, treatments may be different for different species of bee stings. For example, the venom of a bee is acidic, washing the wound with an alkaline solution (such as soapy water), and the wasp venom is alkaline, with an acidic solution (such as vinegar). Therefore, when dealing with bee stings, the appropriate treatment methods should be selected according to the specific situation. | The need for routine tetanus prevention after bee stings depends mainly on the characteristics of the wound and the patients vaccination history.1. Wound characteristics Wound depth and pollution: Clostridium tetanus is an anaerobic bacterium, which usually grows and reproduces in a deep, heavily polluted and anoxic environment. After bee stings, the wound is usually shallow, and the bee tail sting carries mainly acid, rather than the anaerobic environment required for C. tetani. Therefore, the risk of tetanus infection after bee sting is relatively low in terms of wound characteristics. Wound management: Timely and correct wound management is essential to preventing tetanus. After a bee sting, the wound should be cleaned immediately to reduce pain and discomfort and avoid scratching to prevent infection. Proper management of the wound can further reduce the risk of tetanus infection.2. History of tetanus vaccination: tetanus vaccine is an effective means used to prevent tetanus. If a patient has already received a tetanus vaccine under the recommended immunization schedule and has not exceeded the effective protection period (usually 10 years), an additional tetanus vaccine is generally not required after a bee sting. Vaccination history is unclear: if a patient is not sure of his or her tetanus vaccination, or if the last vaccination has exceeded the effective protection period, it is advised to consult a doctor for the need for a bee sting, especially if the wound is deep or heavily contaminated.3. Conclusion To sum up, routine tetanus prevention is generally not required after bee stings. However, if the wound is deep, heavily polluted, or the patients vaccination history is unclear and exceeds the effective protection period of the vaccine, it is recommended to evaluate the need for tetanus vaccine under the guidance of a doctor. Furthermore, regardless of wound conditions, wounds should be managed promptly and correctly to reduce the risk of infection. | Honeycellulitis caused by bee sting is a more serious skin and subcutaneous tissue infection, which should consider the specific situation of the patient and take comprehensive treatment measures. The following are the specific treatment suggestions for cellulitis caused by stings: 1. Emergency treatment to remove the sting: If the sting remains in the skin after stings, use sterilized tweezers or needles as soon as possible to avoid squeezing the wound to avoid the spread of venom. Wound cleaning: Wash the wound with plenty of water or soapy water to neutralize the venom and reduce the risk of infection. Local cold compress: the initial cold compress can be used to reduce swelling and pain.2. Drug therapy Antibiotic treatment: Cellulitis usually requires oral or intravenous antibiotics to control the infection. Commonly used antibiotics include penicillin, cephalosporins, etc., and the specific drug selection should be determined according to the condition and drug susceptibility test results. Symptomatic treatment: cold compress can be used for physical cooling in high fever. People with difficulty eating need fluids to maintain nutrition and fluid balance. An assisted ventilation such as oxygen inhalation was given during shortness of breath. If the patient unbearable pain, can be added painkillers, such as pain tablets, compound acetylsalicylic acid tablets. Supplement: adequate vitamins, such as vitamin C, complex vitamin B, vitamin E, etc., help to promote body recovery.3. Local treatment of wet compress or application: early cellulitis can be used 50% magnesium sulfate wet compress or apply golden powder, fish stone paste, etc., help to reduce swelling and pain. Cutting and drainage: if an abscess forms, cutting and drainage should be done in time to remove pus and reduce pressure. For cellulitis under the bottom of the mouth and the jaw, it should be cut and decompression as soon as possible to prevent laryngeal edema and compression of the trachea.4. Physical therapy ultraviolet therapy: ultraviolet therapy has the functions of sterilization, analgesic and anti-inflammation, promoting wound healing, and is suitable for some patients with cellulitis. Ultrashort wave physical therapy: Ultrashort wave therapy uses ultra-high frequency electromagnetic wave to act on the human body, which is suitable for the treatment of various inflammation, and has the advantages of non-invasive and no drug side effects.5. General treatment for rest and elevation of the affected limb: If cellulitis occurs in the leg, the affected leg should be raised to help reduce swelling and pain and speed up recovery. Maintain good nutrition status: follow a balanced nutrition, foodDiversified principles that contribute to physical recovery. Pay attention to personal hygiene: keep the local clean and dry, avoid sewage soaking the wound, to prevent the aggravation of infection.6. Timely medical treatment: cellulitis is a serious infection, so timely medical treatment is diagnosed and treated by a doctor. Do not use medication to delay the condition. Avoid reinfection: During treatment, avoid the chance of reoccurring bacterial infection and keep the wound clean and dry. Observe the changes of the condition: closely observe the changes of the patients condition, such as body temperature, breathing, heart rate and other vital signs, as well as the improvement of symptoms such as local redness and pain. If there is any abnormality, seek medical advice immediately. In conclusion, the cellulitis caused by bee stings requires comprehensive treatment measures, including emergency treatment, drug treatment, topical treatment, physical therapy, and general treatment. During the course of treatment, patients should closely cooperate with the doctors treatment plan, and pay attention to personal hygiene and nutritional status. |
|  | Long-term Follow-up | Long-term Follow-up | Long-term Follow-up | Long-term Follow-up | Long-term Follow-up |
| Question | Which patients need long-term follow-up after discharge after bee stings? | What does the content of long-term follow-up after bee sting patient discharge have? | What does the health education content of the rehabilitation period after bee stings include? | How to follow-up and manage patients with secondary renal failure after bee sting? | How to undertake psychological guidance when bee sting injury patient leaves the hospital? |
| Answer Engine 1 (ERNIE Bot 3.5) | After bee stings, certain specific types of patients require long-term follow-up after discharge. These patients mainly include the following categories: 1. Severe patients: patients with severe allergic reaction, shock, and other severe organ damage after bee sting, long-term follow-up is required even after discharge. This is because critically ill patients may use a large number of drugs during the treatment process, such as glucocorticoids, anti-allergy drugs, etc., which may have some side effects on the patients body or need to gradually reduce the drug dosage. Moreover, physical recovery in critically ill patients takes longer, and long-term follow-up allows timely detection and management of possible complications or sequelae.2. Patients with basic diseases: If the patient himself has basic diseases such as hypertension, diabetes and heart disease, he may be more likely to have complications or aggravation after bee sting. Such patients require close monitoring after discharge for control of the underlying disease and adjusting treatment options as needed. Long-term follow-up can help doctors to timely understand the changes in the patients condition and avoid deterioration.3. Patients with strong allergic reactions: some patients will have strong allergic reactions, such as dyspnea, laryngeal edema, hypotension, etc. Such patients may require continued allergy medication or other special treatments after discharge. Long-term follow-up ensures timely and effective treatment and prevents the recurrence of allergic reactions.4. Patients with incomplete treatment: If the patient is poorly treated or has residual problems (such as local infection, incomplete resolution of inflammation, etc.), drug treatment or regular review should be continued after discharge. Long-term follow-up can ensure that patients receive continuous and effective treatment and promote disease recovery.5. Doctors recommend patients with long-term follow-up: when the patient is discharged, the doctor will give advice on whether to need long-term follow-up or not according to the specific situation and treatment effect. If the doctor considers it necessary for the patient to have long-term follow-up, the patient should actively cooperate with the patient on time. In conclusion, patients requiring long-term follow-up after bee stings mainly include severe patients, patients with underlying diseases, patients with strong allergic reactions, and treatmentIncomplete patients as well as patients whose physicians recommend long-term follow-up. Long-term follow-up can timely detect and deal with possible complications or sequelae, to ensure that patients receive continuous and effective treatment and promote recovery. At the same time, patients should also actively cooperate with the doctors follow-up arrangement, return to the patients on time, and follow the doctors advice for treatment and care. | Bee sting patients after discharge long-term follow-up content mainly includes the following aspects: 1. The condition monitoring and assessment symptoms observation: regularly ask patients whether new symptoms or existing symptoms, such as local redness, pain, itching, fever, headache, etc., and whether there is allergic reaction, such as the breathing difficulty, laryngeal edema, etc. Signs examination: At the follow-up time, the patients vital signs are monitored, including body temperature, pulse, respiration, blood pressure, etc., to assess the overall health status of the patient. Laboratory tests: According to the specific condition of the patient, laboratory tests such as routine blood test, urine routine test, liver and kidney function may be required to understand the patients physical recovery.2. Treatment and rehabilitation guidance Drug guidance: For patients who need to continue to take drug treatment, follow-up should understand the medication situation of patients, including drug type, dosage, and usage, and patients should be guided to use drugs correctly to avoid the occurrence of adverse drug reactions. Rehabilitation exercise: according to the rehabilitation needs of patients, develop personalized rehabilitation exercise plan, such as local massage, joint activities, muscle strength training, etc., to promote the recovery of the affected limb function. At the same time, guide patients to carry out appropriate daily activities to avoid overwork. Diet and nutrition: According to the patients nutritional needs and taste preferences, make a reasonable diet plan, encourage the patients to keep a light diet, and avoid the intake of spicy, greasy and stimulating food. At the same time, patients are instructed to drink more water to promote toxin excretion.3. Psychological support and health education Psychological support: Bee stings patients may have psychological pressure due to pain, anxiety, fear and other reasons. During the follow-up, we should pay attention to the psychological state of patients and provide necessary psychological support to help patients establish the confidence to overcome the disease. Health education: popularize the knowledge of bee sting prevention, first aid and rehabilitation to patients and their families, and improve their self-protection ability. Tell patients to avoid contact with bees, wasps and other insects. If stings are caused, correct treatment should be taken immediately.4. Follow-up and time follow-up: follow-up can include telephone follow-up, consultation, door-to-door follow-up, letter contact, etc. The specific method should be determined according to the actual situation of the patients and the regulations of the hospital. Follow-up time: The follow-up time should be based on the patients condition and treatment needs. Generally speaking, patients with large side effects, complex conditions and critical conditions should be followed up at any time after discharge. For chronic patients generally requiring long-term treatment or patients with slow disease recovery, they should be followed up once within 2 to 4 weeks after discharge, and at least once every three months thereafter. The specific follow-up time should be determined by the doctor according to the specific situation of the patient.5. Other precautions record and feedback: During the follow-up visit, the patients condition change, medication status, rehabilitation progress and other information should be recorded in detail, and timely feedback should be given to the doctor. The questions and suggestions raised by the patients should be carefully listened to and properly dealt with. Continuous improvement: Through long-term follow-up, patient needs and expectations can be timely understood, so as to provide reference for hospitals to improve the quality of medical services. At the same time, we can also find out the existing problems and deficiencies in the medical service, and take timely measures to improve them. To sum up, the long-term follow-up of bee stings patients after discharge involves many aspects, such as condition monitoring and evaluation, treatment and rehabilitation guidance, psychological support and health education, follow-up method and time, and other matters needing attention. Through comprehensive and meticulous follow-up work, patients can ensure timely and effective treatment and rehabilitation guidance, and improve the quality of life. | The health education in the recovery period of bee stings mainly includes the following aspects: 1. Psychological guidance and emotional comfort: because the onset after bee stings is more urgent and the symptoms are obvious, the patients and their families are easy to produce tension and anxiety. Medical staff should first comfort the patient and tell them that although bee sting is acute, as long as the treatment is timely, properly handled, the patient is properly coordinated, there is a high cure rate. Knowledge popularization: to introduce bee stings to patients and their families, to stabilize their emotions, and to gain their trust with kind language and cordial attitude.2. Diet guidance Nutrition and easy digestion: it is appropriate to give a diet rich in nutrition, such as milk, water, tea, etc., to promote physical recovery. Avoid stimulating food: avoid drinking and eating pepper, onion, ginger, garlic and other spicy and stimulating food, and it is not recommended for patients to eat a large number of hairy crab, shrimp, beef, mutton and other food, so as not to aggravate poisoning or allergy. High quality protein: guide the patient to eat high quality protein content of food, give light, low salt, high vitamin, low fat, easily digestible diet, a small number of meals.3. Work and rest guide rest and activities: during the dangerous period, rest in bed and let the affected limb brake to slow down blood circulation to slow down the spread of toxins in the body. Recovery period can be gradually slight activity, to do not feel fatigue is appropriate.4. Drug guidance Follow the doctors advice: strictly follow the doctors advice on time and dosage, and report to the doctor and nurses in the process of medication, so as to timely treatment. Drug selection: bee sting can be weak alkaline solution (such as 2-3% sodium bicarbonate, soapy water, light lime water, etc.), to neutralize the acid toxin; wasp sting needs weak acid solution (such as vinegar, 0.1% dilute hydrochloric acid, etc.). For mild symptoms, symptomatic treatment or infusion, intravenous injection of 10% calcium gluconate, or oral snake medicine; for allergic patients, promptly use adrenal cortin and antihistamine.5. Special guidance for wound treatment: at home, you can also use small needles or tape paste method to take out the wasp thorns, but do not squeeze. Local external skin of 10% ammonia or insect bite dermatitis medicine, can also be used 5%~10% sodium bicarbonate solution cold wet compress can reduce pain. Pain management: if the pain is obvious, you can take the corresponding measures to stopPain, such as 1% hydrochloric acid root alkali solution plus 2% lidocaine in the sting proximal or peripheral subcutaneous injection, can quickly relieve pain and swelling.6. Disease observation, guidance and monitoring of vital signs: monitor body temperature, heart rate, heart rhythm, breathing frequency and rhythm, and record the daily fluid volume in and out, so as to detect and handle abnormal conditions in time.7. Life guidance Personal protection: Try to wear long-sleeved clothes, masks and gloves during field activities to prevent bee stings. Try to wear light color clothes when spring to suburban activities, do not apply perfume, to prevent attracting bees. Avoid disturbance: If a colony is found to move nearby, detour to prevent disturbance. If under swarm attack, cover your head with clothes and escape the sphere of influence as soon as possible. Education children: education children not to tease the beehive, found honeycomb to completely destroy, to eliminate wasps and larvae. Strengthen personal protection when destroying hives.8. Regular review: review the blood routine, liver function, kidney function, myocardial enzyme spectrum, etc., to assess the physical recovery. To sum up, the health education in the recovery period after bee stings covers psychology, diet, work and rest, medication, special guidance, condition observation, life and return visit, aiming to help patients fully recover and prevent their recovery. | Follow-up management of patients with secondary renal failure after bee sting is a comprehensive process designed to ensure recovery of renal function, prevent complications, and improve the quality of life. The following are specific recommendations for follow-up management: 1. Follow-up Time Acute Phase Follow-up: Follow-up should be more frequent in the first few months after discharge. It is generally recommended to have weekly visits within 1 month after discharge in order to timely monitor renal function changes and adjust the treatment schedule. Stable follow-up: As the patients condition is stable, the follow-up interval can be gradually extended. Generally, stable patients can visit again every 2-3 months, or according to the doctors advice.2. Follow-up content: Renal function monitoring: regular monitoring of blood creatinine, blood urea nitrogen and other renal function indicators, as well as routine urine, electrolytes and other related tests, to assess the recovery of renal function. Clinical symptom observation: ask the patient for symptoms of edema, changes in urine volume, nausea, vomiting, and other discomfort or complications. Medication guidance: to understand whether the patient takes the medicine according to the doctors advice, especially the treatment drugs for renal insufficiency, such as diuretics, antihypertensive drugs, etc. At the same time, evaluate the efficacy and side effects of drugs, and adjust the medication regimen in time. Lifestyle guidance: guide patients to maintain a healthy lifestyle, such as reasonable diet, moderate exercise, avoid overwork, etc. At the same time, remind patients to pay attention to personal hygiene, to prevent infection. Psychological support: pay attention to the psychological state of patients and provide necessary psychological support and counseling. Encourage patients to maintain a positive and optimistic attitude, and enhance their confidence to overcome the disease.3. Follow-up method Outpatient follow-up: Patients go to the hospital clinic on time, and doctors conduct face-to-face examination and guidance. Telephone follow-up: within a period of time after discharge, the patient can know the condition changes and medication through telephone follow-up, and give timely guidance and advice. Online follow-up: Use Internet platforms, such as the official hospital website, wechat official account, etc., to provide online consultation and return visit services for patients, so as to facilitate patients to understand the condition and obtain guidance at any time.4. Special condition deterioration: if the patient has a sharp decline in renal function, severe edema, highIn emergencies such as kalemia, medical treatment should be sought immediately and corresponding treatment measures should be taken. Management of complications: If patients have infection, anemia, hypertension and other complications, corresponding treatment plans should be formulated according to the specific situation, and follow-up management should be strengthened.5. Personalized follow-up: follow-up management should be personalized according to the specific situation of patients, including follow-up time, follow-up content, follow-up methods, etc. Multidisciplinary cooperation: For patients with complex conditions or requiring multidisciplinary cooperation, doctors in relevant departments should be organized to conduct consultation and discussion, and formulate comprehensive treatment plans. Patient education: strengthen patient education, improve patients cognitive level of the disease and self-management ability, and promote patients rehabilitation. In conclusion, the follow-up management of patients with secondary renal failure after bee stings is a complex and meticulous process, which requires the joint efforts and cooperation of medical staff, patients and their families. Through scientific follow-up management, the changes in the condition can be found and handled in time, and the treatment effect and quality of life can be improved. | The psychological guidance of bee stings patients at discharge is an indispensable part of the rehabilitation process, which is of great significance to relieve the tension of patients and enhance the confidence of rehabilitation. Here are the specific psychological guidance advice: 1. Emotional comfort and cognitive reconstruction emotional comfort: first, medical staff should be kind language and kind attitude to comfort patients, told them although bee sting is severe, but as long as timely treatment, handled properly, with good follow-up rehabilitation measures, usually can get good treatment effect. This positive form of communication helps to reduce patient tension and fear. Cognitive reconstruction: to explain the pathogenesis of bee sting, the treatment process and the matters needing attention in the rehabilitation period, so as to help them establish a correct understanding of the disease. At the same time, emphasize the importance of rehabilitation period, so that patients understand that actively cooperate with treatment and rehabilitation is the key to health recovery.2. Emotional management and psychological support emotional management: guide patients to learn self-emotional management, such as deep breathing, meditation, listening to light music and other ways to relieve tension, anxiety and other negative emotions. Patients are encouraged to share their feelings with family and friends and gain emotional support and understanding. Psychological support: to provide the necessary psychological support, such as psychological counseling, psychological intervention, etc. For patients with large mood fluctuations, professional psychological counselors can be invited to provide face-to-face counseling to help them better cope with psychological pressure.3. Enhance rehabilitation confidence and self-care ability to enhance rehabilitation confidence: to enhance patients rehabilitation confidence by sharing successful cases and encouraging words. Having patients see examples of other patients successfully recovering after experiencing similar conditions serves to stimulate their determination to overcome the disease. Improve self-care ability: before discharge, the necessary rehabilitation education and self-care skills training. Such as guiding them on how to use drugs correctly, how to observe changes in the condition, and how to maintain a healthy lifestyle. The mastery of these skills helps to improve the patients self-care ability and promote the rehabilitation process.4. Family and social support Family support: encourage patients families to actively participate in the rehabilitation process, and provide emotional support and life care for patients.familyThe understanding, care, and support of the court members are crucial to the patients recovery. Social support: Social support resources for patients, where possible. For example, the introduction of relevant rehabilitation institutions, support groups, so that patients can feel the warmth and care from the society. In conclusion, the psychological guidance of bee sting patients at discharge should focus on emotional comfort, cognitive reconstruction, emotional management and psychological support, enhancing rehabilitation confidence and self-care ability, and family and social support. Through comprehensive psychological intervention measures, help patients through the recovery period, recover as soon as possible. |
|  | Public Health and Prevention | Public Health and Prevention | Public Health and Prevention | Public Health and Prevention | Public Health and Prevention |
| Question | How to carry out public health education in areas with high incidence of wasp sting? | What are the high-incidence seasons of bee stings and the high-risk groups? | What content should the emergency treatment plan for mass bee stings include? | What are the environmental assessment and improvement measures after wasp stings? | Is it recommended for the removal and management of beehives in areas with a high incidence of bee stings? How to implement it? |
| Answer Engine 1 (ERNIE Bot 3.5) | Public health education in the areas with high incidence of wasp stings is an important measure to prevent and control the wasp stings and protect the health of the people. The following are a series of specific public health education strategies: 1. Clear educational objectives to improve public awareness: enhance residents understanding of wasps, and understand the habits and activities of wasps. Prevention knowledge popularization: teach residents how to prevent wasp stings, including daily protection, avoid disturbing wasps and other measures. Emergency handling ability: improve residents emergency handling ability when encountering wasp stings, including self-rescue and mutual rescue skills.2. Develop educational content Basic knowledge of wasps: the species, distribution and habits of wasps. The activity pattern and peak period of wasps (for example, the high incidence period is in August, September and October). Prevention: Reduce exposure: Wear long-sleeved clothing, trousers and hats to avoid excessive skin exposure. Avoid attraction: do not use strong scented cosmetics, perfumes, etc., to reduce the attention of wasps. Environmental management: trim the courtyard trees and loosen the soil to reduce the wasp nesting opportunities; avoid planting succulent plants before and behind the house. Avoid disturbance: in the wild, avoid disturbing wasps, especially do not actively attack or drive wasps. Emergency treatment: leave the scene quickly: once stung by wasps, leave immediately to avoid further attack. Check the wound: check whether there are poison thorns in the skin, if the wound should be removed. Clean the wound: Clean the wound with soap and clean water to remove the residual venom. Cold compress: Apply ice cubes or a cold towel to the wound to relieve pain and swelling. Medical attention: If systemic allergic reactions occur or the symptoms continue to worsen, seek medical attention immediately.3. Choose educational methods Media publicity: use TV, radio, newspaper, Internet and other media platforms to widely publicize the knowledge of prevention and treatment of wasp stings. Make vivid and easy to understand popular science publicity materials, such as posters, folding pages, animations, etc., to improve the public awareness. Community activities: hold lectures, consultations, free consultations and other activities in community centers, parks, schools and other places, and invite professional doctors or health education experts to explain the relevant knowledge of wasp stings. Distribute publicity materials and provide face-to-face consultation and guidance. School education: will be waspsThe knowledge of prevention and treatment of stings is incorporated into the school health education curriculum, and students self-protection ability can be improved through classroom teaching and practical activities. Training professionals: to provide professional training for medical staff, community workers and school teachers to improve their emergency handling ability and health education level.4. Strengthen monitoring and evaluation and establish a monitoring system: establish a monitoring system for wasp stings, collect and analyze relevant data in time, and understand the occurrence and trend of wasp stings. Assof educational effectiveness: regularly evaluate the effect of public health education to understand the publics knowledge of wasp stings and behavior changes. Adjust educational strategies and methods in time according to the evaluation results to improve the pertinacity and effectiveness of education. Through the implementation of the above public health education strategy, it can effectively improve the awareness and prevention awareness of the residents in the areas with high incidence of wasp stings, reduce the occurrence of wasp stings, and protect the health and safety of the people. | The high incidence season and high-risk group of bee stings are mainly as follows: 1. High incidence season The high incidence season of bee stings is mainly concentrated in summer and autumn, especially from August to November every year. During this period, due to the warm weather, lush flowers and fruits, bees, wasps (also known as wasps or wasps) and other bees had frequent activities, people were prone to bee bites in outdoor activities such as outing, adventure, gardening, etc., which led to the occurrence of stings.2. High-risk group outdoor activity enthusiasts: People who like to conduct outdoor adventure, hiking, camping and other activities are likely to become high-risk groups for bee stings in complex areas when living in mountainous areas, rural areas and suburban areas. Specific occupational groups: sanitation workers: in the garbage cleaning, pruning the green belt and other work, easy to disturb the bees hidden in the grass, trees. Green workers: in afforestation, pruning branches and other work, may destroy the bee nests or disturb the swarm. Farmers and agricultural workers: When working in the field, they may be exposed to beehives or attacked by bees. Other outdoor workers: such as electric workers, construction workers, etc., may also experience bee stings while working outdoors. Children: Due to their strong curiosity and lack of self-protection awareness, children are prone to attack when they are exposed to bees, so they are also one of the high-risk groups of bee stings. Parents should strengthen the education and supervision of their children to avoid approaching beehives or provoking bees. Allergic people: Some people are allergic to bee venom. Once they are stung, they may have severe allergic reactions, even life-threatening. Therefore, people with allergic constitution should be more vigilant and take protective measures in outdoor activities. In conclusion, in order to prevent the occurrence of bee stings, people should improve their awareness of prevention, reduce outdoor activity time or take protective measures in summer and autumn. Meanwhile, specific occupational groups and high-risk groups such as children should strengthen their awareness of self-protection, to avoid contact with bees. | The emergency treatment plan for swarm bee stings should be a comprehensive, rapid and effective response plan, aiming at minimizing injuries, protecting the life safety of patients, and providing timely and necessary medical treatment. The following is a detailed emergency treatment plan: 1. Preparatory risk assessment and early warning: assessment of local bee activities, especially in the high-incidence seasons such as summer and autumn. Warning signs for possible mass bee stings (such as parks, orchards, mountain areas, etc.). Training and drill: bee sting first aid knowledge training for medical staff, first aid staff and community workers. Organize regular emergency drills for mass bee stings to improve the emergency response capacity. Material preparation: prepare sufficient first aid drugs and instruments, such as anti-allergy drugs, adrenaline, painkillers, disinfectant, tweezers, thorn drawing tools, etc. Ensure that the emergency vehicles and communication equipment are in good condition.2. On-site disposal immediate alarm and help: in case of mass bee stings, immediately call 120 emergency number and report to the local health department and emergency management department. Evacuation and isolation: quickly evacuate the injured to a safety area to avoid more injuries. Preliminary classification of the injured, to distinguish the heavy and the wounded. Initial first aid treatment: remove the poison sting: carefully remove the poison sting on the skin to avoid squeezing the venom sac. Clean the wound: Wash the wound with soapy water or clean water to reduce the venom residue. Local treatment: according to the different bee species (bee venom is acidic, wasp venom is alkaline), select the appropriate weak alkaline or weak acidic solution for local treatment. Cold compress: use ice or cold towel to cold compress the wound to reduce pain and swelling. Vital signs monitoring: Vital signs monitoring for the seriously injured, including blood pressure, heart rate, breathing, etc. Find and handle severe symptoms such as shock and dyspnea in time.3. Transport and treatment priority transport of seriously injured: use emergency vehicles priority transport of seriously injured to hospital for treatment. Ensure that the patients vital signs are stable during the transport process. Hospital treatment: after arriving at the hospital, immediately conduct a comprehensive examination and evaluation. According to the patients condition, anti-allergy, anti-shock, protection of important organs and other symptomatic treatment was given. On the outPatients with serious complications such as acute renal failure should take timely treatment measures such as hemodialysis.4. Follow-up treatment and condition tracking: tracking the condition of the injured to understand the recovery. Timely adjust the treatment plan for patients with complications or disease deterioration. Psychological intervention: psychological intervention for the injured people, to relieve their fear, anxiety and other emotions. Provide the necessary psychological support and comfort. Summary and feedback: summarize and analyze the group bee stings to find out the causes and loopholes. Give feedback to relevant departments and put forward suggestions for improvement. Public education and publicity: to strengthen the public awareness and prevention awareness of bee stings. Through the media, community billboards and other channels to popularize bee stings first aid knowledge. To sum up, the emergency treatment plan of mass bee stings should comprehensively consider many aspects, such as preliminary preparation, on-site disposal, transport and treatment, and follow-up treatment, to ensure that the event can be carried out quickly and effectively. | Environmental assessment and improvement measures after a wasp sting is a key step to prevent the recurrence of similar events in the future. The following is a detailed description of these two aspects: 1. Environmental assessment of wasps activity area identification: assess the species, number and activity habits of wasps in the area. Identify common nesting sites for wasps, such as trees, eaves, rock crevices, etc. Analysis of environmental factors: analysis of climatic conditions, such as temperature, humidity, light, etc., these factors may affect the activity frequency of wasps. To investigate the surrounding vegetation, succulent and flowering plants may attract wasps to forage. To assess the impact of human activities on the wasp habitat, such as building construction, agricultural activities, etc. Risk assessment: Assess the risk of wasp stings in different areas and time periods according on the active area and habits. Special attention is paid to childrens play areas, outdoor rest areas and other crowded and exposed areas.2. Improvement measures to reduce wasp habitat: Trim trees and bushes to reduce the opportunity of wasp nesting. Clean up garbage and waste from the courtyard and surrounding areas to avoid becoming a foraging place for wasps. Removed or replacing plant species such as succulent and flowering plants. Set a physical barrier: set a fence or net cover in the area with frequent wasp activity to prevent wasps from entering. Install screen Windows or awnings in crowded areas such as outdoor rest areas and childrens play areas to reduce exposure risk. Strengthen publicity and education: popularize the knowledge and prevention methods of wasp stings to the public through billboards, posters, manuals and other ways. Educate the public to wear long-sleeved clothes, trousers and hats for outdoor activities, and avoid highly scented cosmetics and perfumes. Remind the public not to deal with the wasps when they are found and report to the relevant departments in time. Establish emergency response mechanism: formulate emergency plan for wasp stings, clarify emergency response process and responsible person. Equipped with necessary first-aid drugs and instruments, such as anti-allergy drugs, epinephrine, disinfectant, etc. Regular emergency drills will be organized to improve the emergency response capacity and treatment level. Professional treatment of hu bee nests: for the hu bee nests found, we should contact the professional bee extinguishing company or fire department for treatment. Avoid handling the wasps by yourselfLair to avoid causing wasp attack and damage. Continuous monitoring and evaluation: continuous monitoring of the area after the implementation of improvement measures to evaluate the improvement effect. According to the monitoring results, timely adjust the improvement measures to ensure the long-term effective prevention of wasp stings. In conclusion, the environmental assessment and improvement measures after wasp stings need to comprehensively consider multiple aspects of wasp activity habits, environmental factors and human activities. By reducing the wasp habitat, setting up physical barrier, strengthening publicity and education, establishing emergency response mechanism, professional treatment of wasp nests, and continuous monitoring and evaluation, similar incidents can be effectively prevented in the future. | In areas of high incidence of stings, the removal and management of beehives is a problem to be considered carefully considered. The following is a detailed analysis and implementation suggestions for this problem: 1. Whether to recommend removal and management is recommended for removal and management, but should be based on the following considerations: Risk assessment: First, the size and location of the honeycomb need to be evaluated to determine whether it poses a potential threat to the surrounding population. Demolition and management is necessary if they are located near crowded areas or traffic arteries, and the colony is frequent. Professional treatment: because the removal of honeycomb is dangerous, priority should be given to asking a professional bee removal service company or fire department for treatment. These professionals have extensive experience and professional tools to remove the hives safely and effectively. Long-term prevention: After the removal of beehives, a series of measures should be taken to prevent the formation of new beehives, such as regular cleaning of garbage, food debris, etc., to reduce the habitat and foraging opportunities of bees and other insects.2. 1 Preliminary preparation Contact professionals: contact the bee removal service company or fire department through professional channels to make an appointment for door-to-door service. Prepare protective equipment: If you decide to handle it by yourself (not recommended), prepare protective equipment such as long-sleeve clothing, gloves, trousers, anti-bee clothing, as well as removal tools such as pesticides and torches. Evacuation: Before handling the honeycomb, the surrounding crowd should be evacuated to avoid accidents.2.2 Reconnaissance and evaluation of the removal process: conduct detailed reconnaissance and evaluation of the location, size and surrounding environment of the honeycomb to determine the removal plan. Selection method: according to the specific situation of the honeycomb, select the appropriate removal method, such as bagging method, fire attack method, water attack method, etc. It should be noted that the safety and environmental protection should be considered when choosing the methods to avoid causing fire or pollution of the environment. Implement the removal: perform the removal operation according to the scheduled protocol. Keep calm during the operation to avoid disturbing the swarm. In case of swarm attack, take protective measures and evacuate the site.2.3 Post-treatment and cleaning site: After removing the honeycomb, the site should be carefully cleaned to ensure that there is no residual swarm or venom. Destroy the hive: burn the removed hive by fire or dig it deepBury it and avoid becoming a habitat for insects such as bees again. Continuous monitoring: Regular monitoring of the surrounding environment to prevent new hives from formation.2.4 Preventive measures to reduce attraction sources: timely cleaning up of garbage, food residues, etc., and do not provide opportunities for bees and other insects to perch and forage. Closed entrances: Repair potential entrances to roofs, walls and underground, reducing the opportunity for bees and other insects to find their nests. Raise public awareness: strengthen publicity and education, improve the public awareness and awareness of bee sting prevention. In conclusion, it is necessary for honeycomb removal and management in areas of high sting incidence, but it should be ensured under professional guidance and effective precautions should be taken to reduce the shape of future hives. |
