# Supplementary material for: Clinical Management of Wasp Stings Using Large Language Models: Cross-Sectional Evaluation Study
Source: J Med Internet Res. 2025 Jun 4;27:e67489. doi: 10.2196/67489 (PMC12177424; doi:10.2196/67489)
Supplement: Multimedia Appendix 2 [file jmir_v27i1e67489_app2.docx]

| Engine 2 | | | | | |
| --- | --- | --- | --- | --- | --- |
|  | Basic Knowledge | Basic Knowledge | Basic Knowledge | Basic Knowledge | Basic Knowledge |
| Question | What are the common species of bee stings worldwide? | What are the main pathophysiological mechanisms of bee stings? | What are the main components of bee venom and their biological effects? | What are the global epidemiological characteristics of bee stings? | What are the common local and systemic reactions after bee sting? |
| Answer Engine 2 (ERNIE Bot 4.0) | Bees: Bees are a familiar species of bees. Although they are relatively less toxic, they can still cause local pain and swelling after being stung. The venom of bees is acidic, and the sting usually remain in the skin after being stung. Wasp (wasp, wasp): wasps are widely distributed and highly toxic. When they feel a threat, they attack in attacks. After being stung by wasps, severe allergic reactions may occur and even lead to death. The bee venom of wasps is basic. Mine bee: This is a very toxic bee, mainly distributed in Guangdong, Hainan, Jiangxi and other areas of the mountain jungle areas. After being stung by landmine bees, there will be a greater risk of life, the wound will quickly appear pain, dizziness, numbness and other symptoms. Tiger bee, killer bee, ox horn peak: these bees are also very toxic species. They have poison glands in the posterior segments of the abdomen, which communicate with the tubular tails of bees, emitting venom and injected into tissue. Overall, while bees generally do not actively attack humans, they may attack when threatened or misidentify humans as a threat. Therefore, you should be vigilant during contact with bees and seek medical attention immediately if stings. Note that not all species are listed above, and species distribution and toxicity may vary. When contacting and handling bees, professionals should be consulted and necessary safety measures taken. | The main pathophysiological mechanism of bee stings can be summarized as the following points: venom injection and composition: bees (such as bees, wasps, etc.) when sting the body, the tail of the poison will stab into the skin, and inject the venom into human tissues. These venoms are complex components, including biogenic amines, polypeptides, kininins, and enzymes. Local reaction: The venom causes severe pain, numbness, swelling, and edema in the local tissue. This is due to the direct stimulating and toxic effects of the components in the venom on the skin and subcutaneous tissues. Systemic reaction: After bee venom enters the blood circulation, it can trigger a series of systemic reactions. These reactions include, but are not limited to, acute allergic reactions (e. g., urticaria, dyspnea, etc.), intravascular hemolysis (causing anemia and jaundice), rhabdomyolysis (possibly causing acute renal failure), abnormal coagulation function (bleeding tendency), and multiple organ function impairment (e. g., liver, heart, nervous system, etc.). Serious complications: In severe cases, bee stings can lead to life-threatening complications such as shock, acute renal failure, and cardiac arrest. These complications are often associated with a systemic venom-triggered inflammatory response and multiple organ failure. In conclusion, the pathophysiological mechanisms of bee stings involve local venom irritation and systemic toxic effects, which may lead to severe local and systemic reactions and even life-threatening. Therefore, when dealing with bee stings, measures should be taken quickly to reduce the damage of venom to the human body, and seek medical treatment in time. | The main components and biological effects of bee venom can be summarized as follows: 1. Main components: Bee venom (especially the venom of bees and wasps) mainly contains a variety of proteins, enzymes and organic chemicals. Specifically, its components may include histamine, serotonin, bradykinin, hyaluronidase, peptides, and proteins, as well as specific toxins such as honeybee toxins. In addition, it may contain some special enzyme lines and low molecular protein hemolytic peptide and phosphatase.2. Biological effect: Local reaction: After the bee venom touches the human body, it usually immediately causes local reactions such as redness, pain, itching and skin redness at the stabbed site. This is due to the inflammatory reactions and allergic reactions resulting from the components in the venom. Systemic reactions: In more severe cases, bee stings may cause systemic reactions such as difficulty breathing difficulties, numbness of hands and feet, nausea, vomiting, headache, fainting and anaphylactic shock. These responses may be due to the effects of some components of the venom on the nervous system, cardiovascular system, and immune system. Neurotoxicity: Some components of bee venom, such as bee venom peptide, are neurotoxic and can affect the nerve conduction and the normal function of the nervous system. Hemolysis and anticoagulant effect: Bee venom also has a strong hemolytic effect, which can cause red blood cell rupture. At the same time, it also has an anticoagulant effect, which can prolong the blood clotting time. Anticancer effects: Recent studies have found that certain types of wasp venom contain specific toxins (such as "MP1") to fight cancer cells, which provides a new idea for the development of future cancer drugs. Overall, the biological effects of bee venom are complex and diverse, including both local stimulatory and inflammatory responses and broad effects on various systems throughout the body. It should be noted that the venom composition and effects of different bee species may vary, and therefore the specific responses will vary between bee species. | The global epidemiological characteristics of bee stings are mainly reflected in the following aspects: universality: More than 100 million people are bitten by hymenopteran insects every year, including bees, wasps and other wasps. This suggests that bee stings are a global public health problem. High incidence: It is reported that about 56% to 94% of people in the world may be stung by bees and wasps in their lifetime, showing the high incidence of bee stings. Bee stings are more common in South Asia, Southeast Asia and southern China. Seasonal: Bee stings mainly occur in July to November each year, which may be related to the frequent outdoor production and recreational activities during this period, increasing the chance of contact with bees. Hazardous: Bee stings can not only cause local skin damage, but also may cause systemic allergic reactions and tissue and organ function damage. In severe cases, it may even cause fever, headache, nausea, vomiting, and even lead to shock and death. About 10% of the worlds annual bee stings can be life-threatening, showing the serious dangers of bee stings. In conclusion, bee stings have global epidemiological characteristics such as universality, high incidence, seasonality and harmfulness. Therefore, the public should strengthen the awareness of bee stings, understand the first aid measures, in order to reduce the harm of bee stings. | The common local and systemic reactions after bee stings can be summarized as follows: 1. Local reaction: redness: the stings will quickly appear redness phenomenon, which is due to the inflammatory reaction caused by bee venom stimulation of the skin. Pain: After bee stings, patients usually experience severe pain, and the degree of pain may vary by individual differences and bee species. Pruritus: Local skin may appear itching, patients may not resist scratching, but this may aggravate skin damage. Blares or skin necrosis: In a few cases, bee stings may cause blisters or necrosis on the skin.2. Systemic reaction: allergic reaction: some patients may have an allergic reaction to bee venom, with urticaria, laryngeal edema, wheezing and other symptoms. Severe cases may even appear bronchospasm, asphyxia and other life-threatening reactions. Poisoning symptoms: After bee venom enters the blood, it may cause a systemic immune response, leading to nausea, vomiting, abdominal pain, diarrhea and other symptoms. Severe cases may also appear muscle spasm, coma, shock and other toxic symptoms. Organ function damage: In rare cases, bee stings may lead to acute renal failure, hemolysis and other multiple organ function damage. It should be noted that responses after bee stings vary by individual differences, with some individuals may develop only mild local symptoms while others may experience severe systemic reactions. Therefore, after being stung, we should observe the symptom changes closely and timely medical treatment. |
|  | Early Treatment | Early Treatment | Early Treatment | Early Treatment | Early Treatment |
| Question | What is the first step after the wasp sting? | What is the difference between bee stings and wasp stings? How to accurately identify and judge it? | How to clean the wound of bee sting correctly? How to choose the cleaning liquid? | How to deal with local pain after a wasp sting? | What is the process of field first aid for bee stings? |
| Answer Engine 2 (ERNIE Bot 4.0) | The first step after the wasp sting mainly includes the following points: Remove the bee needle: after the wasp sting, the wound should be checked first to confirm whether there is residual bee needle. If there are bee needles, they need to be removed as soon as possible. When removing the bee needle, the extrusion wound should be avoided to prevent the toxin from spreading in the human body. The needle can be gently picked using a needle or medical forceps. Clean the wound: After removing the bee needle, you need to clean the wound. Because the venom of wasps is weakly alkaline, acidic substances (such as vinegar) can be used to neutralize the venom, reduce the amount of toxins in the human body, and prevent the occurrence of malignant poisoning. After these steps are completed, professional medical help should be sought as soon as possible to further address possible allergies, poisoning, or other complications. If the injury appears redness, pain and other symptoms, measures such as ice compress can be taken to relieve the symptoms. If severe systemic reactions occur, such as shock, immediate emergency treatment is needed. | There are indeed some differences in appearance and symptoms between bee and wasp stings. The following is a summary and comparison of the wound characteristics of these two stings to help accurately identify and judge: 1. Bee stings: Wound appearance: After bee stings, the wound is usually red and may be surrounded by slight swelling. Stinger residue: because the bee sting needle has a barb, the sting needle is easy to stay on the skin after stings. Therefore, the finding of residual stings in wounds is a distinctive feature of bee stings. Venom properties: Bee venom is acidic, which affects how wounds are handled. Symptoms: Bee stings usually cause local pain, redness, and itching. People allergic to honey bee venom may develop more severe allergic reactions.2. Bee sting: wound appearance: after the honeybee sting, the wound may be more red and swollen, and the range of swelling may be larger. Posting: unlike bees, the wasp sting needle is not barbed and easy to pull out, so the wasp usually does not leave a sting in the wound after sting. Venom properties: The venom of wasps is alkaline, which is different from the venom properties of bees, and can also affect the treatment of the wound. Symptoms: Bee stings may lead to more intense local pain and redness, as well as more severe allergic reactions, such as urticaria, breathing difficulties, etc. In addition, wasp stings may also cause systemic symptoms, such as nausea, vomiting, and dizziness. In conclusion, we can observe the appearance of the wound and determine whether there are residual stings, the presence of venom, the nature of the venom and the severity of symptoms. When treating wounds, corresponding neutralization measures should be taken according to the venom properties of different bee species, and timely medical treatment should be sought to reduce symptoms and prevent complications. | Correct cleaning of bee stings is an important step to reduce symptoms and prevent infection. The following are the correct ways to clean bee stings and the selection of cleaning solution: 1. Cleaning method: First, ensure that you are in a safe environment to avoid being attacked by bees again. Carefully wash the wound and the surrounding area with gentle soap and water. Soap water is able to help remove contaminants from the wound surface and reduce the risk of infection. When cleaning, avoid squeezing or scrubbing the wound to aggravate the damage or spread the venom. After washing, gently dry the moisture around the wound with a clean towel.2. Selection of cleaning solution: Alkaline solution: because the bee venom is acidic, the use of alkaline solution (such as soapy water, sodium bicarbonate solution) can neutralize the acidity of the venom, thus reducing pain and swelling. However, it should be paid to avoid excessive concentration when using alkaline solution to avoid irritation to the skin. Hydrogen peroxide or normal saline: These cleaning solutions can also clean wounds and reduce the risk of infection. But also need to pay attention to the concentration and the use of methods, so as not to cause unnecessary damage to the skin. In general, a mild cleaning solution should be selected to avoid the use of highly irritating disinfectant (such as iodine, alcohol, etc.). Keep a gentle approach during the cleaning process to avoid aggravating the wound injury. After cleaning, the wound condition should be closely observed. In case of serious symptoms or allergic reactions, seek medical treatment in time. | The treatment of local pain after wasp sting can be summarized as the following points: remove the sting: first, check the wound, if there is a sting residue, it should be removed as soon as possible. This can be picked out gently by using tweezers or fine needles, but to avoid hard squeezing to prevent the spread of the venom in the human body. Clean the wound: repeatedly rinse the wound with running water to minimize venom residue. This step is critical because timely cleaning is effective in reducing the risk of infection and reducing pain. If possible, acidic liquid (such as vinegar) can be used to neutralize the alkali of wasp venom, but the concentration should not be too high to cause secondary damage to the skin. Cold compress: use ice cubes or cold towel to cold compress the wound, apply 15-20 minutes each time, every few minutes to check the condition of the skin, to prevent frostbite. Cold presses are effective in reducing pain and swelling. Use drugs: If the pain is severe, use appropriate painkillers or anti-inflammatory ointment, either orally or externally. However, note that it is best to consult a doctor or pharmacist for advice before using any medication. Observe the changes of the condition: the wound condition and systemic symptoms should be closely observed after treatment. For severe allergic reactions or poisoning symptoms, such as dyspnea, palpitations, dizziness, etc. Overall, the management of local pain after wasp stings requires multiple considerations, including the removal of poisons, wound cleaning, cold compress, and the use of drugs if necessary. At the same time, it is also very important to closely observe the changes in the condition. If the symptoms do not relieve or have serious reactions after treatment, seek medical attention and professional treatment. | First aid for bee stings should follow the following procedures: bandage: bandage 10 cm proximal to the heart of the wound to block the reflux of venom into the heart by veins and lymph. However, the bandage should not be too tight, and every 15 minutes should be relaxed for 1 minute, and the total time of the bandage should not exceed 2 hours to prevent limb ischemia and necrosis. This step helps to reduce the venom diffusion. Pick and suction toxins: Check the wound carefully, with a needle tip or sterile forceps. Note not to remove the poison directly with tweezers or squeeze the wound hard to avoid injecting more venom into the body. If a needle or forceps cannot be found, the thorn can be carefully sucked out with the mouth, but pay attention to oral hygiene and subsequent disinfection. Washing wound: When bee sting, because the venom is acidic, the wound can be washed with weak alkaline liquid such as soapy water, 5% sodium bicarbonate solution or 3% light ammonia to neutralize the venom. If stung by wasp or wasp, its venom is alkaline, the wound can be washed with 1% acetic acid or weak acid liquid such as household vinegar. If it is impossible to determine what kind of bee sting it is, rinse the wound with running water. Local cold wet compress: use cold towel or ice bag to the wound cold wet compress, apply 15-20 minutes each time, can reduce pain and swelling. Use of drugs: local redness can be topical calamine lotion or white lotion to dissipate inflammation. When the pain is severe, analgesics can be used. In case of allergic reaction, antihistamine drugs, adrenal corticosteroids should be used in time. Observation and medical treatment: after first aid treatment, the patients condition changes should be closely observed. In case of severe systemic reactions, such as rash and rapid expansion, dyspnea and other allergic symptoms, seek medical attention immediately. Following this procedure allows effective emergency measures after bee stings to reduce pain and prevent deterioration. |
|  | Allergy Response Management | Allergy Response Management | Allergy Response Management | Allergy Response Management | Allergy Response Management |
| Question | What are the typical manifestations of allergic reactions caused by bee stings? | How to identify and urgently deal with the anaphylactic shock caused by bee stings? | What is the drug treatment plan of allergic reaction after bee sting? What is the first-line medication? | How to assess the risk of allergic reactions in patients with bee stings? | How to prevent and manage late hair-type allergic reaction after bee sting? |
| Answer Engine 2 (ERNIE Bot 4.0) | The typical manifestations of allergic reactions caused by bee stings can be summarized as follows: 1. Skin symptoms: redness and swelling: the rapid redness and swelling phenomenon occurs in the area of bee stings. Pain and pruritus: The wound usually feels intense pain, accompanied by itching feeling. Rash or urticaria: Some people may develop a rash, and in severe cases, they may develop systemic or localized urticaria.2. Gastrointestinal symptoms: abdominal pain, nausea and vomiting: Due to an allergic reaction and irritation of the gastrointestinal mucosa, patients may feel abdominal pain and develop symptoms of nausea and vomiting. Diarrhea: Some people may also have the symptoms of diarrhea.3. Respiratory symptoms: throat swelling and difficulty breathing: allergic reactions can lead to throat swelling, which can cause difficulty breathing. Wheeze and cough: Patients may develop symptoms of wheezing and cough. Shorger of breath: Respiratory rate may be accelerated due to airway obstruction.4. Cardiovascular system symptoms: decreased blood pressure: when the allergic reaction is severe, the blood pressure may drop, leading to dizziness, cold sweat and other symptoms. Palpitations and arrhythmia: The heart may be stimulated, with palpitations and arrhythmia.5. Systemic symptoms: In rare cases, bee stings can cause severe systemic reactions, such as anaphylactic shock or multiple-organ failure. These symptoms may vary by individual variation, and may vary in severity. Seek immediate medical attention for allergic reactions, especially severe symptoms such as dyspnea, palpitations, or a drop in blood pressure. At the same time, for the people with known allergy to bee venom, we should try to avoid contact with bees, and carry emergency drugs to prevent accidents. | Anaphylactic shock from a bee sting is a severe allergic reaction that can be life-threatening. Symptoms of anaphylactic shock may include a rapid drop in blood pressure that can cause dizziness, black eyes, or even syncope. Pallor, cold limbs, which is due to breathing difficulties caused by poor blood circulation, may be accompanied by laryngeal edema, chest tightness, palpitations and other symptoms. Change of consciousness, such as irritability, confusion, and even drowsiness, coma, etc.2. Emergency measures Once symptoms of anaphylactic shock are recognized, the following measures should be taken immediately: Stop contact with the allergens: leave the colony as soon as possible and remove the bees. Keep the airway open: move the patient to a safe area to ensure that the airway is clear and receive oxygen if necessary. Indrinephrine injection: If the patient has severe allergic reactions, such as laryngeal edema, dyspnea, epinephrine injection should be given immediately. Adrenaline can ts blood vessels and reduces allergic reactions. Antiallergic drug treatment: Antiallergic drugs, such as antihistamines, are given as soon as possible to reduce allergic reactions. Supplementary blood volume: If the patient has shock symptoms such as decreased blood pressure, venous access should be established immediately and fluid infusion to supplement blood volume and maintain stable blood pressure. Closely observe the changes in the condition: in the first aid process, should closely observe the patients breathing, pulse, blood pressure and other vital signs, as well as the changes in the state of consciousness. Timely medical treatment: after the initial emergency treatment, the patient should be sent to the hospital for further treatment as soon as possible. Please note that the above measures should be conducted under the guidance of a professional medical personnel. For serious allergic reactions such as anaphylactic shock, timely medical attention is the key. At the same time, prevention is also very important, and you should try to avoid exposure to known allergens. | Drug treatment options for anaphylaxis after bee stings mainly include the use of antihistamines, corticosteroids, immunomodulators, antibiotics, and analgesic drugs. The following is a detailed explanation of these drugs: 1. Antihistamines: Action: Reduce itching, redness and other symptoms by blocking histamine receptors. Commonly used drugs: cetirizine, loratadine, etc. Usage: for skin symptoms and mild systemic reactions caused by mild to moderate bee venom, it can be used as a first-line medication.2. Corticosteroids: Function: it has a strong anti-inflammatory effect, and can quickly control the local inflammatory response. Commonly used drugs: hydrocortisone, dexamethasone, etc. Use: Suitable for severe local redness, pain, and systemic allergic reactions, which can be used by external use or injection.3. Immunomodulator: Function: it can regulate the bodys immune response and reduce excessive immune response. Commonly used drugs: cyclosporin A, methylprednisolone, etc. Use: When the patient has severe allergic reaction, but under the guidance of a doctor.4. Antibiotics: Role: prevention or treatment of complications caused by infection. Commonly used drugs: amoxicillin, cefixime, etc. Usage: If you find signs of wound infection or concerns about secondary infection, take it as prescribed.5. Analgesic drugs: Function: to relieve the pain caused by bee stings. Commonly used drugs: ibuprofen sustained-release capsules, acetaminophen tablets, etc. Usage: Suitable for mild to moderate pain. First-line medication: For first-line medication of allergic reactions after bee stings, antihistamines such as cetirizine or loratadine are usually preferred because they are significantly effective for symptoms caused by mild to moderate bee venom and have relatively small side effects. However, specific medication should be determined according to the patient and the guidance of the doctor. | Assessment of the risk of allergic reaction in patients with bee stings can be conducted from the following aspects: 1. Medical history inquiry: Ask the patient for a history of allergy, especially to bee venom or other insect toxins. Understand the patients response after bee sting, such as whether there is redness, itching, dyspnea and other symptoms.2. Clinical observation: Observe the local and systemic symptoms of patients after bee sting, such as the range of swelling, pain degree, urticaria, dyspnea, etc. Pay attention to whether the patient has the influence of the circulatory system, such as palpitation, accelerated heart rate, decreased blood pressure, etc. Observe the effects of the nervous system, such as dizziness, confusion, etc.3. Laboratory examination: Conduct basic blood routine and routine urine examination to assess the overall health status of the patients. Patient sensitivity to bee venom can be determined by serum-specific IgE (sIgE) testing or skin testing.4. Allergy grade assessment: According to the symptoms and signs of each system after bee sting, allergic reactions can be divided into different grades, such as grade I~, so as to facilitate a more accurate assessment of the risk of allergic reactions.5. Special population considerations: For the elderly, children, pregnant women, or patients with chronic diseases (e. g., heart disease, respiratory disease), they may have a higher risk of allergic reactions and need special attention. In conclusion, assessing the risk of anaphylaxis in patients with bee stings requires a comprehensive consideration of medical history, clinical symptoms, laboratory tests, and special population factors. Through a comprehensive evaluation, the risk of allergic reaction can be judged more accurately, and corresponding preventive and therapeutic measures can be taken. | The prevention and management of late-onset allergic reaction after bee stings can be carried out from the following aspects: 1. Preventive measures to avoid inducing factors: avoid the use of fragrant cosmetics, such as perfume, hair gel, etc., to reduce the possibility of attracting bees. It is best to wear light-colored and smooth clothes and hats when going out to reduce the stay and attack of bees. Keep sweets and sugary drinks sealed to avoid attracting bees. Environmental management: Regularly clean courtyards, bins and other areas to avoid leaving food or drink residues, thus reducing the chance of bees to feed or drink water. Avoid activities in areas with frequent bee activity, such as gardens, orchards, etc.2. Management measures On-site first aid: Once stung, leave the sting area immediately and stay away from more bees. Remove the poison as soon as possible, but do not squeeze the sac to avoid the spread of venom. Wash the wound with water or weakly acidic substances (e. g. vinegar) to neutralize the alkalinity of bee venom. Drug treatment and observation: Use appropriate allergic drugs, such as antihistamines, corticosteroids, under the guidance of a doctor to reduce allergic reactions. Closely observe the patients symptoms, such as severe allergic reactions (such as dyspnea, palpitations, etc.), and seek medical attention immediately. Follow-up care: Keep the wound clean and dry, to avoid secondary infection. Drinking more water can help to expel toxins from the body and reduce allergic symptoms. Avoid scratching wounds to aggravate symptoms or cause infection. In conclusion, the prevention and management of late anaphylaxis after bee stings starts from multiple aspects, including avoidance of inducing factors, environmental management, on-site first aid, drug treatment and observation, and follow-up care. These measures can help reduce the risk of bee stings and properly manage allergic reactions after stings. |
|  | Complication Management | Complication Management | Complication Management | Complication Management | Complication Management |
| Question | What serious complications may occur after a bee sting? | What are the causes of respiratory failure caused by bee stings? How to deal with? | What is the management strategy for renal insufficiency after bee stings? | What does the reason that bee sting secondary hair anemia have? How to deal with? | What is the way of blood purification treatment after bee sting? How to choose? |
| Answer Engine 2 (ERNIE Bot 4.0) | Serious complications that may occur after bee stings mainly include: 1 systemic allergic reaction: rash. Throx edema, may cause difficulty breathing. Blood pressure drops, sweating, chest tightness, these symptoms can be life-threatening. Anaphylactic shock, manifested by a sharp drop in blood pressure, confusion, etc.2. Multiple organ function damage: liver injury, may appear jaundice, decreased appetite and other symptoms. Kidney injury, presenting as oliguria or anuria, may require blood purification therapy. Nervous system damage, may induce encephalitis, cerebrovascular accident, lead to consciousness disorders, dizziness and so on. Impaired respiratory system, shortness of breath, wheezing, and severe cases, acute respiratory distress syndrome (ARDS). Impaired circulatory system, palpitations, chest tightness, chest pain, in severe cases, coronary artery spasm and hypotensive shock due to allergic reactions.3. Local malignant lesions: bee stings may appear ulceration and necrosis, and scars may form after healing.4. Muscle injury: Bee venom may cause damage to the central nervous system and peripheral nervous system, which can cause muscle spasm and rigidity, causing muscle strain. It should be noted that the severity and probability of these complications may vary according to individual differences, sting sites, bee species toxicity and other factors. Therefore, after being stings, we should closely observe the changes of the condition and timely seek medical attention to prevent the occurrence and development of complications. | The causes of respiratory failure caused by bee stings mainly include the allergic reaction caused by bee toxicity and the direct toxic effects. The following are detailed reasons and treatment methods: 1. Cause of anaphylaxis: bee venom contains a variety of biological active components, such as histamine, phospholipase A, etc., these substances can cause inflammatory reactions in local tissues. The body has an allergic reaction to bee venom, leading to laryngeal edema and bronchospasm, which can cause dyspnea and respiratory failure. Toxic effect: Some components of bee venom have neurotoxic effect, which can lead to nervous system dysfunction and produce inhibitory effect on the respiratory center. Bee venom can also cause arrhythmia, interfere with the conduction of cardiac electrical signals, affect the heart pumping function, thus indirectly affecting the respiratory function.2. On-site first aid: leave the swarm immediately to ensure that the patient is in a safe environment. Check and clean the wound, remove the residual poison, and avoid squeezing the poison sac. Drug therapy: Use antiallergic drugs, such as antihistamines (cetirizine, loratadine, etc.), to reduce allergic reactions. For severe allergic reactions, corticosteroids (such as dexamethasone) can be used for anti-inflammatory and anti-allergic treatment. If severe dyspnea occurs, a respiratory stimulant may be considered. Respiratory support: Oxygen inhalation therapy was given to improve blood oxygen saturation. In case of respiratory failure, endotracheal intubation or tracheotomy should be performed with mechanical ventilation to assist breathing. Cardiac ECG monitoring and circulatory support: close monitoring of ECG changes, timely detection and treatment of arrhythmia. In case of shock symptoms such as decreased blood pressure, circulatory support measures such as timely rehydration and using pressor drugs. Follow-up treatment and observation: closely observe the changes of the patients condition, and adjust the treatment plan in time. Anti-infection treatment was given to prevent the occurrence of complications. Psychological counseling and support for patients to help them overcome difficulties. In conclusion, the respiratory failure caused by bee stings may result from a combination of allergic reactions and toxic effects. When handling, on-site first aid, drug treatment, respiratory support and ecg monitoring should be taken quickly to ensure the safety of patients. | The management strategies for renal insufficiency after bee stings mainly include the following points: 1. Emergency treatment and preliminary assessment: First, the need to leave the colony immediately and ensure that the patient is in a safe environment. Clean the wound and remove the remaining venom venom, take care to avoid squeezing the sac to reduce further release of the venom. Initial assessment of the patients renal function status, including urine volume, urine color and symptoms of low back pain.2. Drug therapy: Use corticosteroids and other anti-inflammatory drugs to reduce the inflammatory response caused by bee venom, thus reducing the damage to the kidney. Anti-histamines are used under the guidance of a doctor to relieve the increased kidney burden due to allergic reactions. In patients who already have renal dysfunction, doctors may consider using dialysis therapy to eliminate excess toxins and water in the body, thereby reducing the kidney burden.3. Dialysis therapy: If a patient has obvious symptoms of renal insufficiency, such as oliguria or anuria, dialysis therapy may become necessary. Dialysis can help remove toxins and excess water from the body and maintain electrolyte balance, thus creating conditions for the kidneys to recover.4. Monitoring and adjustment of treatment regimen: closely monitor the renal function indicators, such as creatinine, urea nitrogen, as well as electrolyte and acid-base balance state. The treatment plan is adjusted timely according to the monitoring results to ensure the treatment effect and reduce the adverse reactions.5. Nutritional support and rehabilitation: Provide appropriate nutritional support for patients to promote physical recovery and repair of kidney function. In the rehabilitation stage, patients should be guided to take appropriate exercise and rest to avoid overwork to increase the burden on the kidneys.6. Psychological support and education: Provide psychological support to help patients relieve anxiety and fear. Health education for patients to understand the relevant knowledge of renal insufficiency after bee stings, and improve self-management and prevention awareness. In conclusion, the management of renal insufficiency after bee stings requires a comprehensive consideration of medication, dialysis treatment, monitoring and adjusting treatment options, nutritional support and rehabilitation, and psychological support and education. A comprehensive management strategy aims to maximize patient protection of renal function and promote their recovery. | The main cause of anemia secondary to bee sting is hemolysis reaction. When the venom bee releases it, these toxins will invade the cells with the blood circulation, leading to the occurrence of a hemolytic reaction. Mild symptoms may include anemia and dizziness, while severe symptoms may cause bilirubin encephalopathy and persistent fever. The methods of handling anemia secondary to bee stings mainly include the following points: Emergency treatment: First, the need to leave the colony as soon as possible and ensure that the patient is in a safe environment. Clean the wound and remove the residual poison, pay attention to avoid squeezing the sac to prevent further release of the venom. Medication and blood transfusion: For hemolytic anemia, blood transfusion may be required to correct the anemia status, especially when the hemoglobin drops to a low life-threatening level. Use of corticoids and other appropriate drugs to mitigate the hemolytic response and stabilize the erythrocyte membrane. Supportive treatment: Ensure that patients rest adequately and provide appropriate nutritional support to help the body recover. Patients were closely monitored for vital signs and blood indicators, including hemoglobin levels and red blood cell counts. Professional medical care: In the case of severe hemolytic anemia, patients may need to be hospitalized for professional medical care. Medical staff will closely monitor the patients condition and adjust the treatment plan as needed. Follow-up observation and rehabilitation: After treatment, the patient needs to continue under observation to ensure that the anemia improves with no further complications. Carry out rehabilitation activities according to the doctors advice to avoid overwork and not aggravate the condition. In conclusion, the treatment of anemia secondary to bee stings requires a comprehensive consideration of emergency treatment, medication and blood transfusion, supportive care, and professional medical care. Through a comprehensive treatment program, aims to correct the anemia status and promote the recovery. | After bee sting, blood purification treatment is an important treatment method, especially when the patient is in critical. Blood purification treatment mainly includes hemodialysis, blood and fusion and plasma exchange.1. Hemodialysis: principle: Use the principle of semi-permeable membrane to remove harmful substances and excess water from the blood through diffusion and convection. Application: it is suitable for the removal of small molecule toxins and water, and has a good removal effect for some small molecule components in bee venom.2. Hemoperfusion: Principle: draw the patients blood out of the body, adsorbing the toxins in the blood through the adsorbent in the perfusion device, to achieve the purpose of blood purification. Application: it is suitable for the removal of macromolecular toxins, and has a good clearance effect for some macromolecular components in bee venom. Hemoperfusion is mostly used for the removal of early bee sting toxins.3. Plasma exchange: Principle: To separate plasma from whole blood through plasma separation technology, and to supplement equal amounts of fresh plasma or plasma substitutes to remove pathogenic substances from the blood. Application: Suitable for the removal of macromolecular substances, especially the toxins with a high binding rate to plasma proteins. Plasma exchange can not only remove toxins from plasma, but also reduce metamorphosis, reduce myocardial and liver enzymes, and improve coagulation function. Plasmapheresis is a very effective treatment for patients with severe bee stings, especially when intravascular hemolysis occurs. According to statistics, the success rate of plasma exchange in the treatment of severe bee sting patients is relatively high, and the prognosis of patients is good.4. Selection basis: Severity of patients: for mild patients, only hemodialysis or blood perfusion may be needed; for severe patients, especially in cases intravascular hemolysis, impaired liver and kidney function occurs, plasma exchange should be considered. Composition and molecular size of the toxin: different bee venom components and molecular size are different, you need to choose the appropriate blood purification method according to the characteristics of the toxin. For example, small molecule toxins can be cleared by hemodialysis, whereas medium macromolecular toxins are more suitable for using blood perfusion or plasmapheresis. Patients physical condition and tolerability: different blood netIt requires different physical condition and tolerance of patients. Patients should consider the age, underlying disease, and cardiac function. In conclusion, the selection of blood purification treatment after bee sting should be considered according to the specific condition, toxin composition and molecular size, as well as the physical condition and tolerance. In practice, the doctor will develop a personalized treatment side according to the specific situation of the patient. |
|  | Severity Assessment | Severity Assessment | Severity Assessment | Severity Assessment | Severity Assessment |
| Question | What symptoms after the bee sting suggest that the condition is serious? | How to assess the risk of multiple-organ function damage after a bee sting? | How should bee sting patients respond when appearing soy sauce color urine? | What is the significance of the neurological symptoms in bee sting patients? | How to judge whether bee sting patients need hospitalization? |
| Answer Engine 2 (ERNIE Bot 4.0) | After a bee sting, the following symptoms may indicate a severe illness: systemic allergic reactions: such as dizziness, fever, nausea, vomiting, convulsions, etc., which may indicate that the patient has developed a severe allergic reaction to bee venom. Especially when urticaria, laryngeal edema, wheezing, shortness of breath, or even bronchospasm or asphyxia occur, immediate medical intervention is needed. Severe poisoning symptoms: fever, headache, vomiting, abdominal pain, diarrhea, and restlessness, which may rapidly progress to muscle spasm, coma, shock, pulmonary edema, and acute kidney failure. Multiple organ function damage: bee sting may lead to abnormal liver and kidney function, myocardial damage, hemolytic anemia and other multiple organ function damage performance. The occurrence of these complications often indicates that the disease is already quite serious. In conclusion, patients should seek professional medical assistance immediately after the appearance of systemic allergic reaction, severe poisoning symptoms or multiple organ function damage to prevent further deterioration of the condition. At the same time, patients should also pay close attention to the changes in their symptoms and timely communicate with doctors in order to get the most effective treatment. | The assessment of the risk of multiple organ function damage after bee stings can be conducted from the following aspects: Clinical symptoms observation: observe whether the patient has nausea and vomiting, abdominal pain, diarrhea, hemolysis, jaundice and other digestive system symptoms, which may indicate impaired liver function. Note whether the patient has respiratory symptoms such as dyspnea, wheezing, and shortness of breath, which may indicate that lung function is affected. Patients were monitored for changes in heart rate, heart rhythm, and blood pressure, to assess the function of the cardiovascular system. Observe patients for neurological symptoms such as irritability, headache, dizziness, and convulsions, which may indicate neurological damage. Laboratory examination: through blood examination, focus on white blood cell count, hemoglobin level, platelet count, etc., to assess whether the patients blood system and hematopoietic function are affected. Especially when leukocytes are significantly higher, or hemoglobin levels decrease sharply, it may indicate an increased risk of hemolysis or infection. Check the liver function indicators such as transaminase, bilirubin, as well as renal function indicators such as creatinine, urea nitrogen, to determine whether the liver and kidney are damaged and the degree of injury. Cardiac zymogram and ECG changes were monitored to assess whether the myocardium was impaired. Assof stings: The number and location of stings are also important factors in assessing the risk of multiple organ damage. Multiple stings or stings in sensitive areas (such as head, face, neck, etc.) may increase the risk. Comprehensive judgment: Based on the patients clinical symptoms, laboratory examination results and stings, doctors can comprehensively judge the risk of multiple organ function damage, and make corresponding treatment plans. Please note that the above assessment method is not absolutely accurate, and the specific situation should be comprehensively analyzed according to the patients actual condition and the doctors clinical experience. During the evaluation process, close attention should be paid to the patients condition changes and timely treatment adjustment. | When bee sting patients have soy sauce-colored urine, the following measures should be taken: 1. Emergency treatment and leave the colony immediately to ensure that the patient is in a safe environment. Clean the wound and remove the remaining venom venom, take care to avoid squeezing the sac to reduce further release of the venom.2. Seek professional medical help and promptly seek medical attention to assess renal function and blood status. Soy sauce-colored urine may be a sign of serious symptoms such as kidney injury and hemolytic anemia, which require diagnosis and treatment by professional doctors.3. Treatment for possible causes If kidney injury leads to microvascular rupture and bleeding, and blood enters the urine and leads to soy sauce colored urine, diuretics such as furosemide and bumetanide can follow the doctors advice. If soy-colored urine is caused by hemolytic anemia, treatment may include blood transfusion, intravenous immunoglobulin, and application of hormones. At this point, a large number of red blood cells are destroyed, the heme released into the blood, with the urine to form soy sauce urine. If it is determined to be drug-caused hemolytic anemia, the drug should be stopped immediately and timely medical treatment. If the soy sauce-colored urine is caused by allergic reactions, patients should take oral antihistamines such as cetirizine and loratadine to relieve the symptoms. Allergic reactions may lead to increased capillary permeability, tissue edema, and then symptoms of soy sauce colored urine.4. Follow-up observation and rehabilitation to closely monitor urine color changes, and regularly review urine routine to assess the progress of the disease. Keep the wound clean and dry to avoid infection. Follow the doctors advice, appropriate rest and rehabilitation activities, avoid overwork to aggravate the condition. To sum up, when bee sting patients appear with soy sauce urine, they should first undergo emergency treatment, and then seek professional help, and conduct treatment for possible causes. At the same time, keep the wound clean and dry, avoid infection, and closely monitor the progression of the disease. | The significance of the occurrence of neurological symptoms in patients with bee stings is mainly reflected in the following aspects: 1. Indicating the severity of the disease: the occurrence of neurological symptoms often means that bee venom has had a deeper impact on the patients body. Ingredients of bee venom may trigger allergic reactions or directly affect nervous system function, leading to a range of neurological symptoms. The appearance of these symptoms can be used as an important indicator, indicating that doctors and patients may be more serious disease, and need to take corresponding treatment measures.2. Help with diagnosis and differential diagnosis: Neurological symptoms can help doctors more accurately diagnose bee stings and possible complications. For example, headache, dizziness, disturbance of consciousness and others may be direct effects of bee venom on the nervous system, or indirect effects due to allergic reactions, hemolysis, etc. Through these symptoms, the doctor can judge the developmental stage of the disease and the possible range of influence.3. Guide treatment and care: The onset of neurological symptoms may require specific treatment and care measures. For example, in patients with impaired consciousness, close monitoring of vital signs, airway patency, and necessary supportive care may be needed. In addition, treatment targeting neurological symptoms may also help relieve the patients suffering and prevent further deterioration.4. Prognosis assessment: The recovery of neurological symptoms can also be used as an important indicator to assess patient outcomes. If the patients neurological symptoms gradually reduce or disappear, it usually means that the treatment is effective and the condition is improving. Conversely, if symptoms persist or worsen, treatment program modifications or more aggressive interventions may be required. In conclusion, the occurrence of neurological symptoms in bee sting patients is important for indicating the condition, diagnosis, treatment, and prognostic evaluation. Therefore, physicians should pay close attention to the changes in the neurological symptoms of patients and take corresponding medical measures according to the symptoms. | To judge whether bee stings patients need hospitalization, it can be considered from the following aspects: 1. Stings degree: the number and location of stings is an important indicator to judge the severity of the disease. In general, if the number of stings is more or the stings are more sensitive (such as head, face, neck, etc.), the condition may be more serious and requires hospitalization.2. Clinical symptoms: Patients with severe systemic allergic reactions, such as urticaria, laryngeal edema, wheezing, and shortness of breath, may require hospitalization to control the allergic reactions and prevent further deterioration. If the patient has severe poisoning symptoms, such as high fever, headache, vomiting, abdominal pain, diarrhea, coma, hemolysis, acute renal failure, these are clear indications for hospitalization.3. Laboratory examination results: blood examination such as blood routine, liver and kidney function and other indicators can reflect the internal condition of the patient. If there are obvious liver and kidney function abnormalities, hemolytic anemia, hospitalization may be required.4. Basic health status of the patient: the patients age and underlying diseases will also affect the need for hospitalization. For example, older adults, children, or patients with chronic diseases may be less tolerant to bee venom and more prone to severe symptoms, thus requiring closer observation and treatment.5. Doctor advice: The most important thing is to decide whether to need hospitalization based on the doctors advice. The doctor will make a comprehensive evaluation according to the specific situation of the patient, and give professional treatment advice. In conclusion, to determine whether patients with bee stings need hospitalization, the degree of stings, clinical symptoms, laboratory test results, basic health status of the patients, and medical recommendations should be considered comprehensively. Hospitalization may be necessary if the condition is severe or potentially risky. |
|  | Special Population Management | Special Population Management | Special Population Management | Special Population Management | Special Population Management |
| Question | How can children be specially treated after bee stings? | What are the special nursing measures after bee stings in elderly patients? | What is the emergency treatment strategy after the pregnant mother bee sting? | What should patients with chronic diseases pay attention to after bee stings? | What is the processing point of bee sting after immune deficiency person? |
| Answer Engine 2 (ERNIE Bot 4.0) | The special treatment of children after bee stings can be summarized in the following steps: Remove the sting: First, the wound needs to be carefully examined, and if the sting is found to be still in the skin, it should be gently extracted using forceps. Take care to avoid squeezing the sting to further inject the venom into the body or cause infection. Clean the wound: repeatedly rinse the wound with flowing water to help remove the venom from the surface of the wound. If possible, washing with mild soapy water can more effectively neutralize the venom and reduce symptoms. Local disinfection: After cleaning the wound, gently wipe the water with sterile gauze, and then disinfect the wound with iodophor or alcohol to prevent infection. Ice compress: If the wound has swelling, pain and other symptoms, you can use ice packs for ice compress to reduce the exudation of inflammatory substances and help relieve the symptoms. But make sure the ice does not directly touch the skin to prevent frostbite. External drugs: under the guidance of the doctor, you can locally apply some anti-inflammatory, anti-itching ointment, such as erythromycin ointment, to help relieve the discomfort of the wound. Oral medication: If the symptoms are severe, oral treatment may be required with oral allergy drugs or anti-inflammatory drugs under the guidance of a doctor. Observation and medical treatment: after the treatment of the wound, the condition of children should be closely observed. If severe allergic reactions such as dyspnea, shortness of breath, or wheezing occur, or the wound symptoms continue to worsen, seek medical advice immediately. In addition, the following points should also be paid attention to during the treatment process: avoid letting children scratching the wound, so as not to cause infection or aggravating symptoms. Make sure that children wear loose, soft clothing to reduce friction and irritation to the wound. During the recovery period, avoid eating spicy, stimulating food to avoid affecting the wound healing. | The special nursing measures for the elderly patients after bee stings mainly include the following points: 1. Carefully observe the stings: after the elderly patients are stung by bees, they should first carefully check the stings for any bee sting residue. Since the skin of elderly patients may be more vulnerable and more responsive to bee venom, it is particularly important to remove bee spines and reduce venom into the body.2. Local treatment: Rinse the wound with soapy water or other alkaline solutions to neutralize the acidic components of bee venom and reduce pain and redness. Note the washing gently to avoid further damage to the skin of elderly patients. Cold compress can be applied with ice or cold towel to the sting to reduce swelling and pain. But it is also necessary to ensure that the cold compress items do not directly contact the skin to prevent frostbite.3. Prevention and treatment of allergic reactions: Elderly patients may be more sensitive to bee venom, so they should be closely observed for allergic reactions, such as dyspnea, rash, itching, etc. Once these symptoms appear, seek medical attention immediately. If an elderly patient is known to be allergic to bee venom or has developed mild allergic symptoms, antiallergic drugs such as loratadine or cetirizine can be used under the guidance of a doctor.4. Rest and activities: according to the physical condition of elderly patients and the severity of stings. If the injury is severe or an allergic reaction occurs, rest in bed to avoid excessive activity.5. Diet and water supplementation: encourage older patients to drink more water to accelerate the excretion of toxins in the body. Diet should be light, easy to digest, avoid spicy, stimulating food, so as not to aggravate the inflammatory reaction of the wound.6. Continuous observation and timely medical attention: The symptoms of bee stings may be delayed or gradually worsen, so the condition of elderly patients needs continuous observation. Seek any unusual symptoms, such as high fever, chills, vomiting, diarrhea, etc.7. Psychological support: Bee stings may bring some psychological pressure and panic to elderly patients. Therefore, providing the necessary psychological support and comfort is also part of the nursing measures to help patients remain calm and optimistic, conducive to wound healing and body recovery. | The emergency treatment strategy of pregnant women after bee sting can be summarized into the following steps: 1. Remove the sting: First, pregnant women should carefully check the parts of the bee sting to look for whether there are residual sting. If the sting is found, carefully remove it with tweezers, tape, or hard cards (such as ID cards, bank cards) to avoid squeezing the venom sac around the sting to reduce further release of the venom.2. Clean wounds: Wash wounds with soapy water, soda water, or other alkaline solutions, which neutralize the acidity of bee venom and thus reduce pain and redness. If these solutions cannot be found, you can also rinse with clean water. The wash time can last for several minutes to ensure that the wound is thoroughly clean.3. Cold compress: cold compress to the wound, you can use ice cubes or cold towel, apply to the sting site, apply for 15-20 minutes each time, every few minutes to check the condition of the skin, to prevent frostbite. Cold compresses help reduce swelling, pain and itching.4. Use drugs (need to consult a doctor): if the symptoms are more serious, such as a large area of redness, severe pain or itching, or later, you can consider using an ointment containing corticosteroids, such as halomyasone cream, hydrocortisone cream, etc., to reduce inflammation and allergic reactions. But pregnant women must consult a doctor before taking it to ensure the safety of the drug. If pregnant women develop severe allergic reactions, such as dyspnea, dizziness, etc., they should seek medical advice immediately, and may use oral anti-allergy drugs under the guidance of a doctor.5. Observation and medical treatment: After the above treatment, pregnant women should closely observe their own symptoms. If any abnormal reactions occur, such as persistent pain, redness, dyspnea, palpitations, etc., go to the hospital immediately.6. Preventive measures: In order to avoid bee stings, pregnant women should try to avoid going to gardens, woodlands and other areas with frequent bee activity during pregnancy. If you have to travel to these places, it is recommended to wear long sleeves and avoid items such as perfume that may attract bees. Overall, emergency management strategies after maternal bee stings include sting removal, wound cleaning, cold compress, using medication if necessary, and closely observing symptoms and seeking medical attention. At the same time, it is also important to take precautions to reduce the risk of bee sting. | After patients with bee stings with chronic diseases, special attention should be paid to the following aspects: 1. Timely treatment of wounds: Patients should quickly remove the sting, avoid squeezing the wound, and timely wash the wound with alkaline solution (such as soapy water or 3% sodium bicarbonate) to neutralize the acid venom. If stung by wasps, rinse with an acid solution (such as vinegar or 1% acetic acid).2. Prevent complications: Patients with chronic diseases may have lower immunity and are more prone to complications. Therefore, the wound and systemic symptoms should be closely observed, once chills, fever, headache, nausea and other symptoms, should seek medical treatment in time.3. Pay attention to drug interaction: Patients with chronic diseases may take drugs for a long time, and special attention should be paid to the interaction between drugs during the treatment of bee stings to avoid adverse reactions. It is best to consult a doctor before taking the drug.4. Maintain a stable condition: Bee stings can cause pain and tension, which may adversely affect patients with chronic diseases. Patients should be as calm as possible and treated as prescribed to keep their condition stable.5. Strengthen observation and monitoring: As the physical condition of patients with chronic diseases may be more complex, the response after bee stings may also be more severe. Therefore, patients need to strengthen self-observation, once abnormal conditions, should seek medical advice immediately.6. Reasonable diet and rest: after bee sting, patients should maintain a light diet and avoid irritating food. At the same time, ensure adequate rest, to help to restore the body. Overall, patients with chronic diseases need to treat wounds more carefully after a bee sting, prevent complications, pay attention to drug interactions, and remain stable. At the same time, strengthen observation and monitoring, reasonable diet and rest is also essential. In case of any discomfort, seek medical attention immediately. | The treatment points of bee stings after immune deficiency can be summarized as the following points: 1. Pull out the sting in time: after bee sting, the wound should be checked in the first place and the sting left on the skin quickly. You can use tape paste after uncovering, or with tweezers to avoid squeezing the poison, to prevent venom further injection into the body.2. Clean and neutralize the venom: immediately clean the wound with water, soapy water or other suitable solution. Because the venom of bees is acidic, the alkaline liquid such as soapy water and 5%~10% sodium bicarbonate solution can be used to wash the wound to neutralize the venom and reduce the pain.3. Local cold compress: For cold compress on the stings site, use ice packs or cold towels for 15-20 minutes each time to reduce swelling and pain. Be careful to avoid frostbite on your skin.4. Drug treatment and anti-allergy: If severe redness, pain, or allergic reactions occur, topical anti-inflammatory ointment or oral anti-allergy drugs are used under the doctors advice. Immunocompromised people may be more sensitive to bee venom, so they need to pay close attention to symptom changes and seek medical attention promptly.5. Observation and medical treatment: After ststing, systemic symptoms should be closely observed, especially respiratory, heart rate and other vital signs. In case of dyspnea, palpitations, dizziness and other serious symptoms, seek medical advice immediately.6. Prevention of infection: Patients are more susceptible to infection due to low immune function. Therefore, the wound should be kept clean, avoid exposure to pollutants, and antibiotics should be used to prevent infection if necessary. In general, immunocompromised people should quickly remove the sting, clean and neutralize the venom, use topical cold compress, use drug therapy and anti-allergy measures if necessary, and closely observe the symptoms and seek medical attention. At the same time, infection prevention is also an important link in the treatment process. |
|  | Medication Treatment | Medication Treatment | Medication Treatment | Medication Treatment | Medication Treatment |
| Question | What is the indication that uses antibiotic after bee sting? | What are the commonly used analgesic drugs after bee sting? | What is the basis of the choice of antihistamine drugs after a bee sting? | What is the principle of glucocorticoid use after bee sting? How to choose the dose? | How to undertake fill fluid treatment after bee sting? What are the precautions? |
| Answer Engine 2 (ERNIE Bot 4.0) | The indications for the use of antibiotics after bee stings mainly include the following points: 1. Local skin infection: After bee stings, the sting or improper wound care. At this point, the use of antibiotics can effectively control the infection and prevent the spread of inflammation.2. Obvious systemic symptoms: If the patient has fever, chills, general fatigue and other infection and poisoning symptoms, it indicates that there may be systemic infection. In this case, timely antibiotics are needed to control the infection and reduce the symptoms.3. Red, swelling and purulent of the wound: if there is obvious swelling, heat pain and even suppuration in the bee sting, it indicates that the wound has been infected. Antibiotics should now be used to eliminate inflammation and promote wound healing.4. Abnormal blood tests: If the patients routine blood tests show signs of infection, such as an elevated white blood cell count and an increased proportion of neutrophils, antibiotics are also required.5. High-risk groups: For high-risk groups such as immunocompromised and diabetic patients, the risk of infection after bee stings increases. These patients may need prophylactic antibiotics to reduce the risk of infection. It should be noted that when using antibiotics, the appropriate drugs should be selected according to the clinical situation, and the standard treatment should be conducted according to the doctors advice. At the same time, we should closely observe the changes in the condition and adjust the treatment plan in time. In addition, it is important to prevent infection after ststing. It is recommended to remove the sting, clean the wound and avoid scratching or squeezing the wound. If any symptoms of infection occur, seek professional treatment immediately. | Commonused analgesic drugs after bee stings mainly include the following categories: 1. Nonsteroidal anti-inflammatory drugs: These drugs can relieve mild to moderate pain. Although not often as a preferred option in the analgesic treatment of bee stings, it may also be considered by physicians in some cases.2. Opioid analgesics: For more severe pain, sometimes such drugs may be used. However, please note that such drugs have strong side effects and addictive properties, and therefore they must be used under the guidance of a doctor. However, in the common treatment of bee stings, more specific and common analgesic drugs include: 3. ibuprofen sustained-release capsule: This is an over-the-counter drug, often used to treat mild to moderate pain. It can inhibit the synthesis of prostaglandin, play an anti-inflammatory and analgesic effect. After a bee sting, if you feel obvious pain, you can take it in moderation under the guidance of a doctor.4. Lorphenine extended-release tablets: This is a prescription drug containing ibuprofen and codeine phosphate, which has a strong analgesic effect. The drug may be prescribed in the case of more severe pain caused by bee stings. It should be noted that the use of analgesic drugs should be determined according to the severity of the pain and the advice of the physician. Before using any medication, it is best to consult a doctor or pharmacist for advice to ensure safety and effectiveness. In addition, in addition to analgesic drugs, the treatment of bee stings also includes removing the sting, cleaning the wound, using anti-allergy drugs and other steps. If the injury is serious or allergic reaction, seek medical attention immediately. | The selection of antihistamines after bee stings mainly includes the following points: 1. Anti-allergic effect of drugs: Antihistamines are mainly used to relieve allergic reactions caused by bee stings, such as redness, itching and other symptoms. Different antihistamines have different anti-allergy mechanisms and therefore need to be selected according to the specific patient symptoms and the severity of allergic reactions.2. Safety of drugs: When choosing antihistamines, consider their age, health status, and the presence of other drug interactions. For example, some antihistamines may have specific side effects in the elderly or in children, or may interact with other drugs that the patient is using.3. Side effects of drugs: Different antihistamines may have different side effects, such as lethargy, dry mouth, headache, etc. When choosing a drug, it needs to balance its therapeutic effect against the possible side effects and choose the drug with the least impact on the patient.4. Individual differences of patients: There are individual differences in patients response to drugs, so they need to be adjusted according to the specific situation of patients. For example, some patients may be more sensitive to certain antihistamines and can achieve better therapeutic results. Specifically, the antihistamines commonly used for bee stings include epilastine hydrochloride capsules, olotatadine hydrochloride tablets, cetirizine, loratadine, etc. These drugs can reduce symptoms such as itching and redness caused by bee venom by blocking histamine receptors. When choosing a specific drug, the doctor considers the above factors and the specific needs and preferences of the patient. Overall, the selection of antihistamines after bee sting is a comprehensive process considering multiple factors designed to find the best suitable treatment option for the patient. | The use principles and dosage selection of glucocorticoids after bee stings can be summarized as follows: 1. Use principles: anti-inflammatory and anti-allergic: glucocorticoids have powerful anti-inflammatory and anti-allergic effects, and can effectively reduce the local inflammatory reaction and allergic reactions after bee stings, such as redness, pain, itching and other symptoms. Clear indications: The use of glucocorticoids should be based on clear indications, such as severe allergic reactions, hemolysis, or toxic reactions. Avoid abuse: because glucocorticoids have certain side effects, such as immunosuppression, osteoporosis, etc., so unnecessary use should be avoided to reduce the occurrence of adverse reactions. Use under the guidance of doctors: the use of glucocorticoids should be carried out under the guidance of doctors, and the medication norms should be strictly observed to ensure the safety and effectiveness of medication.2. Dose selection: Initial dose: Based on the patients specific condition and body weight, the doctor will determine an appropriate initial dose. In general, the dose is controlled to areas that can effectively relieve symptoms. Gradreduction: After symptom relief, the dose of glucocorticoids should be gradually reduced to avoid rebound caused by sudden withdrawal. Individual differences: Patients differ in sensitivity to glucocorticoids, so dose selection should be adjusted to patient response. It should be noted that the specific dose and use method should be determined according to the patient details and the guidance of the physician. In practical application, doctors will comprehensively consider the patients condition, age, weight and other factors to develop personalized treatment plans. In addition, for the treatment of bee stings, in addition to glucocorticoids, may also need to cooperate with other drugs such as antihistamines, painkillers, as well as local treatment measures such as cold compress, poison removal, etc. Therefore, patients should follow the doctors advice during the medication process, and do not adjust the drug dosage or change the treatment regimen. | The methods and precautions for rehydration treatment after bee stings are as follows: 1. Methods of rehydration treatment: to judge the condition: First, it is necessary to judge the severity of bee stings. For minor stings, rehydration is usually not required; but is crucial for severe allergic reactions such as dyspnea, and hypotension. Choose appropriate rehydration: commonly used rehydration includes physiological saline, glucose solution, etc. These fluids can help maintain blood pressure stability, correct dehydration and improve circulatory status. Determine the amount of rehydration: the amount of rehydration should be determined according to the patients weight and condition. In general, 20 – 30 mL of fluid supplementation may be required per kg of body weight. In the case of severe hemodynamic disturbances, an increase up to 50 mL per kg of body weight may be required. Intravenous infusion: rapid replenishment by intravenous infusion to maintain water and electrolyte balance and stability of the internal environment.2. Note: Close monitoring: In the process of fluid rehydration, the patients vital signs, including blood pressure, heart rate and breathing, should be closely monitored, so as to find out abnormal conditions in time and take corresponding treatment measures. Control the speed of rehydration: the speed of rehydration should not be too fast to avoid aggravating the burden on the heart or cause other adverse reactions. The rehydration speed should be adjusted according to the specific condition of the patient. Observe allergic reactions: During the rehydration, we should also pay attention to observe whether the patient has allergic reactions, such as rash, dyspnea, etc. In case of allergic reaction, infusion should be stopped immediately and corresponding anti-allergy therapy given. Keep the wound clean: In addition to rehydration treatment, pay attention to keep the wound clean and dry to avoid infection. Follow the doctors advice: the rehydration treatment should be carried out under the guidance of the doctor, and the patients should strictly follow the doctors advice, and do not adjust the amount or speed of the rehydration by themselves. In conclusion, the rehydration treatment after bee stings should be carried out according to the specific situation of the patient, with close attention to the monitoring of vital signs, control of rehydration speed and observation of allergic reactions, so as to ensure the safety and effectiveness of the treatment. |
|  | Wound Treatment | Wound Treatment | Wound Treatment | Wound Treatment | Wound Treatment |
| Question | What are the characteristics of skin damage after bee sting and its clinical significance? | How to calculate the number of bee sting sting needles? | Do you need to remove the poison sting after a bee sting? | Is there a need for routine tetanus prevention after bee stings? | How does the cellulitis caused by bee sting handle? |
| Answer Engine 2 (ERNIE Bot 4.0) | The characteristics and clinical significance of skin damage after bee stings are as follows: 1. Characteristics of skin damage, redness and petechiae: After bee stings, redness and swelling usually occur rapidly in the injured site. This is due to the components in the bee venom leading to local vascular expansion and increased permeability. In addition, petechia may appear from the sting, a sign left when the bee goes into the skin. Blares and blood blisters: If bee venom causes a strong allergic reaction, blisters or blood blisters may form at the injured site. These blisters usually result from excessive local tissue reaction to bee venom and may contain clear fluid or blood. In specific areas, such as eyelids, lips, etc. Pain and pruritus: burning and itching may occur immediately after the bee sting. This sensation is due to the stimulation of nerve endings by chemicals in the bee venom.2. Clinical significance judgment of injuries: the characteristics of skin damage can help doctors determine the severity of bee stings. For example, a wide range of redness, pechiae or the formation of blood blisters may indicate a serious injury. Assessment of allergic reactions: skin damage, especially the formation of blisters and blood blisters, may mean that the patient has a strong allergic reaction to bee venom. This helps doctors to assess the patients allergy status and develop a treatment plan accordingly. Guide treatment: Based on the characteristics of skin damage, doctors can choose the appropriate treatment method. For example, for mild redness and swelling, only topical cold application and observation may be required, while for severe blisters and blood blisters, antiallergic medication and topical treatment may be required. Prevention of complications: By observing the changes in the skin damage, doctors can timely detect and handle the possible complications, such as infection, the spread of inflammation, etc. In conclusion, the characteristics of skin damage after bee stings have important clinical significance for judging the injury situation, assessing the allergic reaction, guiding the treatment, and preventing the complications. Therefore, in the case of bee stings, we should closely observe the skin damage, and timely seek medical treatment. | The calculation of bee sting sting count depends on the patients self-report, the doctors examination and the specific condition of the wound. Here are some possible ways to calculate stcount: 1. Patient Self-report: Patients can usually perceive the number of stings, especially when simultaneously or continuously by multiple bees. This direct perception of patients could provide physicians with a preliminary estimate of sting needle number.2. Wound examination: The doctor can calculate the number of sting needles by carefully examining the wound. Each stings usually leaves marks, such as redness, petechiae or small wounds. The number of bee stings can be estimated by counting these apparent wound markers.3. Reference to medical history: If the patient has sought medical treatment and has relevant medical records, the doctor can also refer to these medical history data to understand the patients bee stings, including the time of the stings and the possible number of needles. It should be noted that since bee stings may trigger allergic reactions or local inflammatory reactions, sometimes the appearance of the wound may become blurred or merged, leading to the difficulty in accurately counting each individual sting point. Therefore, when calculating sting shots, physicians need to consider both patient reports, visible evidence of the wound, and any available medical history information. From the actual case report, we can see that the needle number of bee stings can be determined by patient feedback and doctors examination. For example, there are case reports showing that after the patient was bee stung, the doctor determined the specific number of sting ings, which provides important information for further treatment. In general, although a fixed calculation formula cannot be given, the number of stings for bee stings can be estimated relatively accurately based on the patients self-perception, the doctors wound examination and the medical history data. | The question of whether the sting needs to be removed after bee stings can be answered from the following aspects: 1. The influence of ststing The bee sting contains venom, these venom will be injected into the body after stings, causing pain, swelling and other symptoms. If the sting remains in the skin, it will not only continue to release the venom, but may also cause further infection or inflammation.2. The necessity of removing the venom to prevent continued injection: removing the venom can prevent the venom from entering the body, thus reducing the symptoms of stings. Reduce the risk of infection and inflammation: The sting left in the skin may act as a foreign body to cause infection and inflammation, and removing the sting can reduce these risks.3. How to remove the sting When removing the sting, use tweezers or other tools to gently clip the sting, and then pull it out. Avoid hard squeezing or clamping to squeeze more venom into the wound or cause skin damage.4. Treatment after the removal of the sting, the wound should be cleaned and disinfected immediately to reduce the risk of infection. At the same time, depending on the severity of the symptoms, other treatment measures may be needed, such as cold compress, the use of anti-allergy drugs. In conclusion, the removal of venom venom after bee sting is necessary to reduce the continued injection of venom and reduce the risk of infection and inflammation. When removing the sting, attention should be paid to the operation method to avoid causing further damage. After the wound removal, the wound should be properly treated to promote healing. | The question of whether conventional prevention of tetanus after bee stings can be answered from the following aspects: 1. The relationship between bee stings and tetanus bee stings are usually caused by the tail thorns of bees or wasps stabbing into the skin and injecting venom. The wound is generally shallow and does not contain C. tetanus. Tetanus is a disease caused by Clostridium tetanus, commonly found in soil, dust, and feces and entering the body through deep and contaminated wounds.2. Whether routine tetanus prevention is required According to publicly published information, routine tetanus prevention is generally not required after being stung by bees. Because bee stings usually form shallow wounds and do not have the anaerobic environment required for C. tetanus growth. However, in some special cases, such as deeper wounds or the presence of contamination, tetanus prevention may need to be considered. This usually occurs in deep wounds caused by other animals or objects rather not bee stings.3. Preventive measures For general bee stings, the main focus is to reduce allergic reactions and local inflammation, can take wound cleaning, cold compress, the use of anti-allergy drugs and other measures. If the wound is deep or if there is a risk of contamination, seek medical attention promptly and decide against tetanus vaccination based on the doctors advice. In conclusion, bee stings are generally not required for routine tetanus prevention. However, in special circumstances, if the wound is deep or seriously polluted, corresponding preventive measures should be taken according to the doctor. | Cellulitis caused by honey bee stings is an infection of the skin and subcutaneous tissue caused by honey bee stings. Such disease treatment can be carried out from the following aspects: 1. Local treatment: First, the wound should be cleaned to ensure that the wound is clean and prevent further infection. Use 50% magnesium sulfate wet compress or apply golden powder, fish stone paste, to help reduce inflammation and pain. If there is abscess formation in the wound, the drainage should be cut in time to reduce the local pressure and promote healing.2. Systemic treatment: according to the severity of the disease, efficient and sufficient antibiotics, such as penicillin and cefoperazone, are used early to control the infection. Sometimes, appropriate antibiotics should be selected according to the results of pus culture and antibiotic susceptibility test. Supplement enough vitamins, such as vitamin C, complex vitamin B, to enhance the bodys immunity. If the patient is in great pain, painkillers can be added. At the same time, antipyretic drugs can be given according to the situation.3. Supporting treatment: pay attention to improve the patients systemic state and maintain the stability of the internal environment. In case of high fever, use cold compress and physical cooling; maintain nutrition and fluid balance; and give assisted ventilation such as oxygen inhalation during shortness of breath.4. Treatment of special types of cellulitis: for pneumatic subcutaneous cellulitis, it must be isolated in time, and the wound can be washed with 3% hydrogen peroxide solution and iodine volt wet application.5. Medical treatment and monitoring: cellulitis is a serious infection, so it is recommended that patients seek medical treatment in time, and diagnose and treat by doctors, so as not to delay the disease. In the course of treatment, the patients condition changes should be closely monitored, and the treatment plan should be adjusted in time. In conclusion, the management of cellulitis caused by bee stings requires a comprehensive consideration of local management, systemic treatment, supportive care, and the management of specific types of cellulitis. It is important to seek timely medical attention promptly and follow the doctors advice. |
|  | Long-term Follow-up | Long-term Follow-up | Long-term Follow-up | Long-term Follow-up | Long-term Follow-up |
| Question | Which patients need long-term follow-up after discharge after bee stings? | What does the content of long-term follow-up after bee sting injury patient discharge discharge have? | What does the health education content of the rehabilitation period after bee stings include? | How to follow-up and manage patients with secondary renal failure after bee sting? | How to undertake psychological guidance when bee sting injury patient leaves the hospital? |
| Answer Engine 2 (ERNIE Bot 4.0) | After bee stings, the following patients may need long-term follow-up after discharge: 1. Severe allergic reactions: Bee stings may cause severe allergic reactions, including, but not limited to, dyspnea, hypotension, shock, etc. For such patients, long-term follow-up is needed to ensure that allergic reactions do not recur or worsen.2. Patients with multiple organ injuries: bee venom may cause damage to multiple organs, such as liver, kidney, heart, etc. For patients with multiple organ injury, long-term follow-up is needed to monitor the recovery and functional status of the organs.3. Complications during the treatment: If patients have complications during the treatment, such as infection, bleeding, etc., then these patients also need long-term follow-up to ensure that the complications are effectively controlled and prevent their recurrence.4. People with special medical history or basic diseases: for patients with special medical history or basic diseases, such as coronary heart disease, diabetes, etc., bee stings may aggravate the condition. Therefore, such patients need long-term follow-up to ensure that the underlying disease does not worsen by bee stings. In general, patients with severe conditions, allergic reactions, multiple organ injuries, complications, and special medical history or underlying diseases should be followed up for long-term follow-up after discharge to ensure that their health is continuously improved and monitored. This can detect and address potential health problems in time and improve the quality of life of patients. | The contents of long-term follow-up of bee stings patients after discharge mainly include the following aspects: 1. Disease monitoring and evaluation: regularly ask and observe whether the patients have redness, pain, itching and other symptoms again, in order to evaluate the recovery of bee stings. Vital signs such as body temperature, blood pressure and heart rate were monitored to ensure the patients stable physical condition.2. Observation of allergic reactions: pay attention to whether the patient has allergic reactions, such as rash, dyspnea, etc., so as to take timely measures. Special attention should be paid to patients with a known allergy to bee venom for changes in their allergic symptoms.3. Organ function examination: Regular liver and kidney function examination is conducted to ensure that bee venom does not cause long-term damage to important organs. For patients presenting with multiple organ injuries, more frequent monitoring of relevant indicators is needed.4. Drug therapy and adjustment: Adjust the drug therapy regimen according to the recovery of patients to ensure the safety and effectiveness of medication. For patients requiring long-term anti-allergic or anti-inflammatory drugs, attention should be paid to the side effects and dependence of drugs.5. Psychological support and counseling: to understand the psychological state of the patients, and to provide the necessary psychological support and counseling. Help patients to establish a positive mindset and promote recovery.6. Life and dietary guidance: Give patients guidance on life and diet, and suggest to avoid exposure to allergens. Patients are reminded to keep the wound clean and dry and to avoid infection.7. Health education: to popularize the knowledge of prevention, first aid and rehabilitation of bee sting. Teach patients how to manage bee stings correctly and how to seek help in abnormal situations. Through the above long-term follow-up content, the recovery situation of bee stings patients can be fully understood, and the potential health problems can be found and dealt with in time, and the quality of life of the patients can be improved. At the same time, it can also provide continuous support and care for patients, and promote the recovery of their physical and mental health. | The health education in the recovery period of bee stings mainly includes the following points: 1. Psychological guidance: Bee stings may cause pain and discomfort, leading to tension and anxiety in patients. Patients should be comforted and encouraged to inform them that the bee sting has a high cure rate as long as the treatment is timely and properly handled. Through the kind of language and superb nursing technology to obtain the trust of patients and their families, to stabilize their emotions.2. Diet guidance: It is recommended that patients eat nutritious and easily digestible food, such as milk, fruit, etc., which is conducive to recovery. Patients are advised to avoid drinking alcohol so as not to aggravate wound irritation and risk of infection.3. Work and rest and activity guidance: During the rehabilitation period, patients are recommended to rest in bed and reduce the amount of activity to reduce the diffusion rate of toxins in the body. When the symptoms improve significantly, slight activities can be gradually performed, but to avoid overwork.4. Drug use and wound treatment: according to the doctors advice, the correct use of topical drugs and oral drugs, such as anti-allergy drugs, anti-inflammatory drugs, etc. Teach patients how to manage the wound correctly, such as neutralize toxins with weak alkaline or weak acidic solutions, and regular cleaning and disinfection of the wound.5. Observation and response: teach patients how to observe the changes in the wound, such as the range of redness, pain, etc., so as to find abnormal conditions in time. Patients are advised to seek medical attention immediately when abnormal conditions occur (such as wound infection, aggravated allergic reactions, etc.).6. Prevention and self-protection: popularize the knowledge of bee sting prevention to patients, such as wearing long-sleeved clothes in field activities, avoiding using fragrant cosmetics, etc. Teach patients how to protect themselves during a swarm attack, such as covering their head with clothing and fleeing quickly.7. Follow-up and follow-up: emphasize the importance of regular return visits so that doctors can assess recovery and adjust treatment options. Remind patients to pay attention to the follow-up arrangement, and timely communicate with doctors about the rehabilitation progress and problems encountered. Through the above health education content, it can help bee stings patients to better manage their health status during the rehabilitation period, and promote the wound healing and body recovery. | The follow-up management of patients with secondary renal failure after bee stings can be carried out from the following aspects: 1. Regular return visits: Arrange patients to go to the hospital for regular return visits, usually weekly or biweekly, the specific frequency depends on the patients condition. On the second visit, the doctor will evaluate the recovery of renal function, check the healing of the wound, and adjust the treatment plan.2. Renal function monitoring: At each return visit, renal function examination, including blood creatinine and blood urea nitrogen indicators, should be performed to evaluate the recovery of the kidney. Based on the results of the examination, the doctor will adjust the drug dosage and treatment regimen to ensure a gradual recovery of kidney function.3. Drug management: to ensure that patients take drugs on time and in quantity, especially for the treatment of kidney failure, such as diuretics, kidney protection drugs, etc. Check medication side effects regularly, adjust drug dose or change medication in time to reduce adverse reactions.4. Dietary guidance: Patients are advised to eat a low salt, low fat and low protein diet to reduce the kidney burden. Patients are encouraged to eat more vitamin-rich foods, such as fresh fruits and vegetables, to boost immunity.5. Psychological support: Provide psychological counseling services to help patients relieve anxiety and pressure, and enhance rehabilitation confidence. Encourage patients to communicate with their family and friends and share their feelings to gain more social support.6. Health education: Explain the causes, treatment methods and expected effects of renal failure in detail to enhance the patients cognition and understanding. Teach patients how to self-monitor the condition, such as observing urine volume, urine color, etc., so as to find abnormal conditions in time.7. Life guidance: Patients are advised to avoid overwork and strenuous exercise to avoid aggravating the condition. Remind patients to keep good living habits, such as smoking cessation and alcohol restriction, and keep enough sleep, etc. Through the above follow-up management measures, it can effectively monitor the recovery of patients with secondary renal failure after bee stings, and the potential health problems can be found and handled in time, and promote the recovery of patients. At the same time, it can also provide continuous support and care for patients to help them return to a healthy life. | The method of psychological guidance for bee sting patients when they discharge can be summarized into the following points: 1. Soothe emotions: First, to calm the patients emotions, let them know that although bee sting is painful, but as long as the treatment is timely, most people can fully recover. Previous cases of a successful cure can be used to encourage patients and enhance their confidence.2. Explain the condition: explain the causes of bee sting injury, treatment process and possible sequelae of bee stings to patients in detail, so that they can have a clear understanding of their condition. Emphasize the importance of following the doctors advice, including regular return visits, regular medication medication, etc.3. Provide social support: encourage patients to communicate with family and friends, share their feelings and troubles, so as to gain more social support. It is suggested that family members accompany patients more and give them emotional support and encouragement.4. Coping with anxiety and fear: Teach patients simple relaxation techniques, such as deep breathing and meditation, to help them relieve anxiety and fear. Remind patients to keep a positive and optimistic attitude and believe that they can overcome the disease.5. Establish a healthy lifestyle: patients are advised to maintain good work and rest habits, ensure adequate sleep and proper exercise. Encourage patients to participate in some beneficial activities, such as listening to music and reading, to distract their attention and improve their mood. Through the above psychological guidance measures, it can help the bee sting patients to better cope with the psychological challenges after discharge, and speed up the rehabilitation process. At the same time, these measures can also enhance patients self-awareness and self-management ability, and improve their quality of life. |
|  | Public Health and Prevention | Public Health and Prevention | Public Health and Prevention | Public Health and Prevention | Public Health and Prevention |
| Question | How to carry out public health education in areas with high incidence of wasp sting? | What are the high-incidence seasons of bee stings and the high-risk groups? | What content should the emergency treatment plan for mass bee stings include? | What are the environmental assessment and improvement measures after wasp stings? | Is it recommended for the removal and management of beehives in areas with a high incidence of bee stings? How to implement it? |
| Answer Engine 2 (ERNIE Bot 4.0) | The public health education in areas with high incidence of wasp stings can be conducted from the following aspects: 1. Strengthen the knowledge popularization of wasps. Through brochures, propaganda videos, lectures and other forms, popularize the habits, identification characteristics and hazards of wasps to the public, so as to improve peoples cognition of wasps. Emphasize the aggression and toxicity of wasps, so that people can understand the serious consequences of wasp stings, so as to enhance the awareness of prevention.2. Preventive measures Teach the public how to avoid wasp attacks, such as wearing long-sleeved clothes, trousers and hats, avoid using strong scented cosmetics, and not to actively catch or disturb wasps and other insects. Remind the public to wear dark long clothes for protection in densely distributed areas, and pay attention to the surrounding environment to avoid disturbing the honeycomb.3. First aid treatment education and education that the public should wrap the exposed parts with clothes as soon as possible, can squat still, do not run quickly, not to mention repeatedly beat. If attacked by a swarm, leave the site as soon as possible and seek medical attention. At the same time, the public is taught how to do simple first aid treatment, such as cleaning the stings, cold compress, etc.4. Community health education through community activities, lectures and other forms, to popularize the knowledge of wasp sting prevention and control, improve residents awareness of prevention and self-protection ability. Use the community propaganda board, broadcast and other channels, regularly release about the wasp prevention knowledge and early warning information.5. School and childrens education Carry out wasp prevention knowledge courses in schools, so that students can understand the harm of wasps and preventive measures. Through interesting activities or experiments, deepen the students cognition and understanding of wasps.6. Cooperation and training Cooperate with local medical and fire protection departments to jointly carry out the prevention and control of wasp stings. Train relevant personnel to improve their emergency handling capacity and treatment level. Through the above measures, public health education can be effectively carried out in areas with high incidence of wasp sting, improve public awareness and self-protection ability, and reduce the occurrence of wasp stings. | The high incidence season and high-risk group of bee stings can be summarized as follows: 1. High incidence season: summer and autumn season, especially from August to November every year, is the high incidence season of bee stings. This may be related to the frequent activity of bees and more peoples outdoor activity during this time.2. High-risk groups: Outdoor enthusiasts: People who like outdoor activities such as outings, adventure, picnic and other activities are more likely to encounter bees and may be stings. Sanitation workers, green chemicals and other outdoor workers: The nature of these jobs determines that they need to work outdoors, thus increasing the risk of contact with bees and stings. Especially in the garbage cleaning, pruning plants and other work, may accidentally disturb the colony. People living in mountainous or suburban areas may be more active, so people living in these areas may have an increased risk of bee stings. In general, the high incidence season of bee stings is mainly concentrated in summer and autumn, while the high-risk groups mainly include outdoor enthusiasts, outdoor workers and people living in mountainous or suburban areas. To reduce the risk of bee stings, it is recommended that these populations take the necessary protective measures during the relevant seasons and activities. | The emergency treatment plan for mass bee stings should include the following contents: 1. Start of the plan and organizational coordination After receiving the report of mass bee stings, the emergency treatment plan should be activated immediately. Set up a leading group for the emergency response of bee stings, responsible for organizing, coordinating and directing the whole emergency response work. According to the injury and the site situation, the leading group should quickly formulate the rescue plan and allocate resources from all parties.2. On-site rescue and transfer of the wounded The on-site disposal team should arrive at the scene quickly to carry out the treatment and transfer of the wounded. Conduct a preliminary assessment of the injured and conduct graded treatment according to their injuries to ensure that the seriously injured have priority to treatment. Use proper protective equipment to ensure the safety of rescue personnel.3. Medical treatment and drug treatment The medical rescue team shall conduct a preliminary evaluation of the injured, and take corresponding treatment measures, such as wound cleaning, pain relief, anti-allergy, etc. The injured with allergic constitution should be given anti-allergic treatment, and pay close attention to the changes in the condition. Prepare enough antihistamines, painkillers, etc., to deal with possible allergic reactions and pain.4. Logistics Support and Communication Liaison The logistics support team shall be responsible for emergency supplies reserve, vehicle deployment and communication support. Ensure that the food, accommodation and transportation needs of the field rescuers, the injured and their families are met. Keep smooth communication, timely transfer of on-site situation and rescue progress.5. Publicity report and Information Release The publicity report team shall be responsible for the publicity report and information release of the emergency response work. To release the progress of emergency response to the public in a timely manner, guide the public to correctly respond to bee stings, and avoid panic.6. Preventive measures and follow-up work Strengthen publicity and education to improve the publics awareness of prevention and self-rescue ability of bee stings. The site of the incident shall be cleaned up and checked to eliminate potential safety risks. Psychological counseling and care for those involved in the rescue to ensure their physical and mental health. Through the above emergency treatment plan, we can effectively respond to mass bee stings and minimize casualties and losses. | The environmental assessment and improvement measures after wasp sting mainly include the following points: 1. Environmental assessment: site safety assessment: First, to ensure the safety of the site, and to assess whether there are still signs of wasp activity, so as to avoid secondary injury. Injury assessment: to determine the severity of the stings so as to take appropriate treatment measures.2. Improvement measures: Clean up the site: immediately clean up the site and remove the items that may attract wasps, such as sweets and garbage, so as to reduce the aggregation of wasps. Eliminate wasp nests: If wasp nests are found, ask professionals to deal with them to avoid further damage. Personal protection: When working in active areas, wear long-sleeved clothing, trousers and hats to reduce the bare skin and reduce the risk of being attacked by wasps. Avoid chemicals such as perfumes: Some chemicals may attract wasps, so avoid perfumes, cosmetics and other items that may attract wasps in areas where wasps are active. Publicity and education: strengthen the publicity and education to the public, improve peoples awareness of wasps, and understand the habits and preventive measures of wasps. Through the above environmental assessment and improvement measures, the occurrence of wasp sting events can be effectively reduced, and the severity of injury can be reduced. At the same time, these measures can also help to improve the publics awareness and prevention ability of wasps, and ensure peoples safety and health. | 1. Is it recommended to remove and manage beehives in areas with high incidence of bee stings? In areas of high incidence, it is very necessary to remove and manage the honeycomb. This can not only reduce the risk of being attacked by bees, but also effectively reduce the incidence of bee stings. Therefore, it is recommended to remove and manage beehives in areas with a high incidence of bee stings.2. How to implement the honeycomb removal and management? The removal and management of the honeycomb requires following certain steps and precautions to ensure the safety and effectiveness of the operation. Here are some recommended implementation steps: Identify the honeycomb location: Determine the location by observing, listening to resident reports, or using specialized equipment. Assess honeycomb: determine the size, species, and activity of the colony. According to the evaluation results, select the appropriate removal method. Prepare the necessary equipment: wear a full set of anti-bee clothing, gloves, masks and other protective equipment. Prepare the tools needed to remove the honeycomb, such as safety ropes, plastic bags, special tools, etc. Remove the honeycomb: choose the appropriate removal methods, such as bagging method, fire attack method or water attack method. Maintain calm during operation and perform removal quickly and accurately. Place the removed hive into a prepared plastic bag and tie the bag mouth tightly. Handling the removed honeycomb: transfer the removed honeycomb to a safe area for processing. Choose to kill the bees inside by fire or soaking in water, and make sure they dont escape. Cleaning up the site: Clean up the debris and residues generated during the removal process. The site was disinfected to prevent the spread of the disease. Follow-up observation and management: Continue to observe the area for new nests for several days after the removal. Regularly inspect the surrounding area to find and process new beehives in time. In addition, to prevent the emergence of new beehives, the following measures can be taken: regularly check the surrounding environment, especially roofs, trees and other places. Closed trash bins and other places that may attract bees. Keep the surroundings clean and reduce food debris and other substances that attract bees. Overall, the removal and management of the hives requires a careful and scientific approach. In the operation process should wear the necessary protective equipment, selectionSuitable removal method and ensure safe cleaning and follow-up observation tube after treatment. |
