# Supplementary material for: Clinical Management of Wasp Stings Using Large Language Models: Cross-Sectional Evaluation Study
Source: J Med Internet Res. 2025 Jun 4;27:e67489. doi: 10.2196/67489 (PMC12177424; doi:10.2196/67489)
Supplement: Multimedia Appendix 3 [file jmir_v27i1e67489_app3.docx]

| Engine 3 | | | | | |
| --- | --- | --- | --- | --- | --- |
|  | Basic Knowledge | Basic Knowledge | Basic Knowledge | Basic Knowledge | Basic Knowledge |
| Question | What are the common types of bee stings worldwide? | What are the main pathophysiological mechanisms of bee stings? | What are the main components of bee venom and their biological effects? | What are the global epidemiological characteristics of bee stings? | What are the common local and systemic reactions after bee sting? |
| Answer Engine 3（Claude Pro） | Several common stings do exist worldwide. Let me briefly introduce the main species for you: 1. Wasp (Yellowjackets): belongs to the social wasp, small size, black and yellow. Often in North America and Europe.2. Bumblebee (Hornets): larger than the wasp, more aggressive. Asian bumblebees are common in Japan and other places.3. Paper wasps (Paper wasps): long and thin, nests such as paper, widely distributed.4. Bees (Honey bees): Aiculture is common, usually mild, but stung when frightened.5. Mafengfeng (Bumblebees): chubby, fluffy, generally gentle.6. Wood bee (Carpenter bees): large size, often nesting in wooden structures. These are some of the main species, but the specific distribution may vary by region. | The main pathophysiology of bee stings involves multiple aspects. Let me explain to you in detail: 1. Toxin injection: bee stings will inject venom into the skin. This venom is a complex mixture containing a variety of active ingredients.2. Main toxin components: -proteins and peptides: such as phospholipase A2, phospholipase B, hyaluronidase-biological amines: such as histamine, norepinephrine-other small molecules: such as acetylcholine, polyamine 3. Local reaction: -pain: mainly caused by peptides in the toxin, Stimulation of pain receptors-inflammation: substances such as histamine, Increased permeability, 4. Systemic reaction: -Anaphylactic reaction: the production of IgE antibodies to bee venom protein, Leading to the release of mediators from mast cells and basophils-causing anaphylactic shock in severe cases 5. Activation of the immune system: toxin components identified as foreign antigens, Activation of the immune response 6. Nervous system effects: certain toxin components can affect nerve conduction, Cause local numbness or tingling sensation 7. Effect of coagulation system: some toxins may affect coagulation function, But generally affects slightly these mechanistic interactions, Together to the clinical manifestations of bee stings. It is noteworthy that for most people, single bee stings usually cause only local reactions. But to allergic individuals or multiple stings may lead to more severe systemic reactions. | Bee venom is a complex mixture with quite broad composition and biological effects. Let me introduce the main components and their effects in detail: 1. Protein and peptides: A) Phospholipase A2 (PLA2) -Effects: destroying cell membranes, Cause pain and inflammation-is also one of the main allergens b) bee venom peptide (Melittin) -Effect: destruction of cell membranes, Lytic red blood cells-with antibacterial and anti-inflammatory effects c) Apamin-effect: neurotoxin, Blocking potassium channels-possibly causing neurological symptoms d) MCD peptide (Mast Cell Degranulating Peptide) -effect: stimulating mast cells to release histamine-aggravating allergic reactions 2. Biological amines: a) histamine-effect: vascular dilation, Increased permeability, Causing itching and pain b) norepinephrine-effect: vasoconstriction, May aggravate local ischemia 3. Enzymes: a) hyaluronidase-effect: decomposition of the extracellular matrix, Promoting toxin diffusion b) Phospholipase B-effect: synergy with PLA2, Strengthening cell membrane destruction 4. Other small molecules: a) acetylcholine-effect: stimulation of pain receptors, Cause pain b) polyamines (such as spermine and spermidine) -Effect: increased capillary permeability 5. Mineral ions: -calcium, magnesium, copper, etc. -Effect: together with other components, Maintain venom pH biological effect summary: 1. Pain: mainly caused by bee venom peptide, PLA2 and acetylcholine 2. The inflammation: caused by PLA2, histamine 3. Allergic reaction: PLA2 and other proteins is the main allergen 4. Neurotoxicity: mainly caused by Apamin 5. hemolysis: mainly caused by bee venom peptide 6. tissue damage: by a variety of enzymes synergy it is worth noting, The combined effect of these components leads to the complex effect of bee venom. Some components (such as bee venom peptide) also show potential therapeutic effects at low doses, such as anti-inflammatory and antibacterial effects, which are also the basis of bee venom therapy research. | The global epidemiology of bee stings is a complex topic involving multiple aspects. Let me outline the main features for you: 1. Morbidity: -Globally, about 0.5% to 3.3% of the population suffer bee stings every year.-The incidence varies greatly in different regions and in different populations.2. Geographic distribution: -It has been reported almost all over the world, but the incidence is high in tropical and subtropical regions.-North America, Europe, Australia, and some Asian countries have reported more frequently.3. Seasonal: -Temperate areas are mainly concentrated in the warm season (late spring to early autumn).-Tropical regions occur all year, but the rainy season may increase.4. Population characteristics: -Outdoor workers (such as farmers, gardeners, construction workers) are at high risk.-Children due to curiosity and outdoor activity are also high-risk groups.5. Mortality rate: -The overall mortality rate is low, about 0.03-0.48/1,000,000 population / year.-The primary cause of death is anaphylactic shock, accounting for more than 80% of the deaths.6. Allergic reaction: -About 0.3% -7.5% of the population is allergic to bee venom.-Anaphylaxis is the most severe complication and can lead to fatal anaphylactic shock.7. Multiple stings: -Most of them are single stings.-Multiple stings (> 50 times) may cause a toxic reaction, but they are rare.8. Occupational relevance: -Beekeepers are a high-risk group, with about 25-65% developing bee venom allergy.9. Regional differences: -The relative paucity of data in developing countries may underestimate the severity of the problem.-Medical resource prognosis is generally better in rich areas.10. Trends: -Global warming may alter the distribution range of some bee species and affect epidemiological patterns.-The pattern of contact between people and bees is also changing during urbanization.11. Reporting systems: -The lack of uniform reporting systems in many countries may lead to incomplete data.12. Economic impact: -Direct medical costs and indirect losses (such as losses of working days) constitute a social and economic burden. These characteristics reflect that bee sting is a global but a public health problem with obvious regional characteristics. Understanding these characteristics may facilitate the development of targeted prevention and treatment strategies. | Reactions after bee stings can be divided into local and systemic reactions. Let me for you the two types of reaction: local reaction: 1. The immediate response: -severe pain-tingling-burning feeling 2. Early reaction (minutes to hours): -redness-itching-local temperature rise 3. Late reaction (24-48 hours): -swelling may further aggravate-pain may continue or aggravate 4. Local large reaction: -more than 10cm swelling-sustainable days-usually not life-threatening 5. Other local symptoms: -skin discoloration-local lymph node enlargement systemic reaction: 1. Mild systemic reaction: -systemic itching-urticaria, nausea, vomiting-dizziness 2. Moderate systemic reaction: -abdominal pain-diarrhea-vertigo-chest tightness 3. Severe systemic reaction (anaphylactic shock): -dyspnea-laryngeal edema-blood pressure drop-loss of consciousness-arrhythmia 4. Other rare systemic reaction: -vasculitis-nephritis-nervous system symptoms (such as seizures) -blood system abnormalities (such as hemolysis) 5. Delayed systemic reactions: -Serum disease-like reactions (7-14 days later) -fever, arthralgia, rash, etc. 6. Reactions caused by multiple stings: -Rhabdomyolysis-Liver and renal function impairment-abnormal coagulation function Note: -Most people will only have local reactions.-Systemic reactions, especially severe allergic reactions, are rare but can be life-threatening.-People with a past history of systemic reaction had a higher risk of stings.-Some groups (e. g. heart patients, the elderly, children) may be more sensitive to bee stings. |
|  | Early Treatment | Early Treatment | Early Treatment | Early Treatment | Early Treatment |
| Question | What is the first step after the wasp sting? | What is the difference between bee stings and wasp stings? How to accurately identify and judge it? | How to clean the wound of bee sting correctly? How to choose the cleaning liquid? | How to deal with local pain after a wasp sting? | What is the procedure of field first aid for bee stings? |
| Answer Engine 3（Claude Pro） | The first step of treatment after a wasp sting is very important to reduce symptoms and prevent complications. The following are the main steps to be taken: 1. Stay away from the danger area: -Quickly leave the area where the colony is active to prevent another sting.-If outside, get inside or safe as soon as possible.2. Assessment: -Quick check for multiple stings.-Observe for signs of severe allergic reactions (e. g. dyspnea, vertigo, etc.).3. Remove the sting (if present): -Quickly scrape the sting with your nail or credit card edge.-Avoid extrusion using forceps to prevent more venom injection.4. Clean the wound: -gently wash the stings with soap and water.-This helps to reduce the risk of infection and remove the residual venom.5. Cold compress: -Use ice packs or cold and wet towel on the stings.-Cold compress can reduce the pain and swelling.6. Elevate the affected area: -If possible, raise the stings to help reduce the swelling.7. Take oral antihistamines (if any): -If carried around, take over-the-counter antihistamines to reduce itching and mild allergic reactions.8. Monitor symptoms: -Pay close attention to the signs of allergic reactions, such as dyspnea, dizziness, nausea, etc.9. Consider seeking medical attention: -If severe symptoms or systemic reactions occur, seek medical attention immediately.-People with a history of allergies should consider seeking medical attention, even if the symptoms are mild.10. Use an epinephrine autoinjector (if any): -If the patient has a history of severe allergies and carries an epinephrine autoinjector, use it immediately in case of a severe allergic reaction. Note: -Do not squeeze the sting site, which may lead to more venom spread.-Avoid using home folk remedies (such as mud, cigarettes, etc.) that may increase the risk of infection.-If the sting is near the mouth or throat, seek medical attention immediately as it can cause respiratory obstruction. | There do be some differences between bee and wasp stings, but in some cases it may be difficult to fully determine. Let me explain in detail the characteristics of these two stings, and how to identify and judge as accurately as possible: bee stings characteristics: 1. Leave bee sting: -Bees usually leave needles and poison capsules after stings.-The needle is slender and may be difficult to see with the naked eye.2. Single stings: -Bees usually sting only once because stings lose the needle and die.3. Wound characteristics: -usually only a small, precise prick.-White or pale swelling may occur around it.4. Venom release: -The poison sac left on the skin continues to pump into the venom until it is removed. Characteristics of wasp sting: 1. No needle: -The wasp can sting people repeatedly without leaving a needle.2. Multiple stings: -The same wasp may sting multiple times.-Common and multiple stings.3. Wound characteristics: -multiple prick points may occur.-The wound is usually larger and presents a red swelling.4. Pain level: -Usually more painful than bee stings. Accurate identification and judgment methods: 1. Check whether there is a needle: -carefully observe the wound, to see if there is a residual needle.-The use of a magnifying glass may help to detect tiny barbed needles.2. Observe the number of stings: -A single stings is more likely to be bees, and multiple stings are more likely to be wasps.3. Assess the pain level: -Although subjective, wasp stings are usually more painful.4. Observe the swelling characteristics: -Bee stings tend to form a small, localized swelling.-Bee sting may cause a greater range of redness.5. Recall insect appearance: -If you see insects, bees are usually small and fluffy.-The wasps are larger and smooth in appearance.6. Consider environmental factors: -Bees often move near flowers or beehives.-The wasps may nest in trees, under eaves, or underground.7. Professional identification: -In some cases, a professional (such as an entomologist or a toxicologist) may be required. It should be noted that while these methods can help differentiate bee and wasp stings, determining which specific insect stings is not the most in an emergency settingimportant. It is more critical to take appropriate first aid measures quickly, especially to observe the presence of severe allergic reactions. | Proper cleaning of bee stings is important to reduce the risk of infection and alleviate symptoms. Here are the correct steps to clean a bee stings and recommendations for the cleaning solution: Correct cleaning steps: 1. Remove the needle (if it is a bee sting): -Quick quickly with your nail or credit card edge.-Avoid using forceps to squeeze the poison sac.2. Soft rinse: -Wash the wound area with gentle water flow.-Avoid using high-pressure water flow to prevent further spread of the venom.3. Use the cleaning solution: -Select the appropriate cleaning solution (see below).-Gently clean the area around the wound.4. Tap dry: -tap the wound with a clean towel or gauze to avoid friction.5. Observation: -observe the wound after cleaning to ensure that there is no foreign body left. Selection of cleaning solution: 1. Soap and water: -the simplest and most common method.-Use a mild, spice-free soap.-Advantages: easy to obtain and effectively clean.-Note: rinse thoroughly and do not leave soap residue.2. Normal saline: -0.9% sodium chloride solution.-Advantages: mild, non-irritating, suitable for sensitive skin.-It can be purchased at the drugstore or homemade (add 9 grams of salt in 1 liter of water).3. Mild disinfectant: -such as chlorhexidine solution (0.05%) or povidone-iodine solution.-Advantages: have the disinfection effect, can reduce the risk of infection.-Note: Some people may be allergic to these solutions.4. AVinegar solution: -Mix equal amount of white vinegar with water.-Advantages: It may help to neutralize some toxins.-Note: Skin skin in small areas before use.5. The baking soda solution: -Add some baking soda to the water.-Advantages: It may help relieve itching.-Note: Do not be used for open wounds.6. Commercial antihistamine or pain relief gel: -such as products containing diphenhydramine or lidocaine.-Advantages: It can directly relieve the symptoms.-Note: Use by the doctors advice and do not overuse it. Avoid alcohol, hydrogen peroxide or iodine, which may stimulate the wound.-If the wound area is large or the location is special (such as near the eye), consider seeking medical attention.-Even minor bee stings should be treated with caution, May require medical intervention.-After washing, ice can be considered to reduce the pain and swelling. | Local pain after wasp stings can be quite intense. Here are some effective treatments: 1. Ice: -Use ice packs or cold wet towel to the injury.-15-20 minutes each, repeated every hour.-Action: Reduce swelling, paralyze nerve endings, and relieve pain.2. Pain relievers: -Oral over-the-counter pain medication, such as ibuprofen or acetaminophen.-As prescribed, note the dose and contraindications.-Effect: Systemic analgesic effect.3. Antihistamines: -Oral antihistamines such as diphenhyphydramine.-Relieve itching and indirectly reduces discomfort.-Note: It may cause drowsiness.4. Topical analgesic gel: -topical products containing lidocaine or benzocaine.-Apply it directly to the wound area.-Role: local anesthesia, rapid pain relief.5. Mud compress: -Use a paste made of cassia bark powder, baking soda, or soil.-Apply gently to the wound area.-Action: It may help to neutralize the toxin and relieve the symptoms.6. Essential oil: -lavender oil or tea tree oil after dilution.-Gently massage the wound area.-Effect: natural analgesic and anti-inflammatory effects.7. Vinegar compress: -Apply apple cider vinegar or white vinegar to the wound.-Action: may help neutralize the toxin and reduce pain.8. Aloe aloe gel: -Apply fresh aloe gel or aloe vera products directly.-Action: cooling effect, reduce inflammation.9. Raise the affected area: -Raise the injured area if possible.-Effect: Reduce blood flow to the injury, reduce swelling.10. Avoid irritation: -Do not scratch or rub the wound.-Wear loose clothing to avoid friction.11. Garlic paste: -Gently smear the fresh garlic on the wound area.-Role: natural anti-inflammatory and antibacterial effect.12. Honey: -Apply all natural honey.-Action: natural antibacterial, may help in wound healing. Precautions: -If the pain continues to worsen or systemic symptoms occur, seek medical attention promptly.-Even minor stings should be treated with caution in people with known allergies.-Test in a small skin area before trying new treatments to prevent allergic reactions.-If the wound shows signs of infection (e. g. increased redness, swelling, suppuration), seek medical attention immediately. | On-site first aid for bee stings should follow a systematic process to ensure the safety of the injured and to provide appropriate treatment. The following are the recommended first aid procedures: 1. Ensure safety: -Quickly move the injured person away from the colony activity area.-Into a safe indoor environment or open areas away from the colony.2. Assessment: -Quickly check the number and location of stings.-Ask the injured person for a history of bee venom allergy.3. Remove bee sting (if a bee sting): -Remove them with nails, credit card edges or similar tools.-Avoid using forceps to squeeze the poison sac.4. Clean the wound: -Gently wash the stings with soap and water.-If possible, use a mild disinfectant.5. Apply a cold compress: -Apply an ice bag or a cold and wet towel to the injury.-15-20 minutes each, repeated at intervals.6. Pain and pruritus relief: -If available, apply a topical analgesic / antiitching ointment containing diphenhydramine or lidocaine.-Consider oral non-prescription painkillers (e. g., ibuprofen).7. Observe the symptoms closely: -Watch out for any signs of allergic reactions, such as dyspnea, dizziness, nausea, etc.-Monitor the degree of swelling at the sting site.8. Handle allergic reactions (if present): -Assist if the injured person carries an epinephrine autoinjector.-In case of severe allergic reaction, call first aid immediately.9. Elevate the affected area: -If possible, raise the stings area to reduce the swelling.10. Rehydration: -Encourage the injured to drink more water and keep hydr.11. Record the information: -Record the time, place, and number of stings.-Observe and record the changes in the symptoms.12. Follow-up care: -The injured is advised to continue to observe the wound for the following days.-If symptoms persist or worsen, seek medical advice.13. Special case treatment: -If the sting occurs near the mouth or throat, keep ice in the mouth and seek medical attention immediately.-For multiple stings (more than 10).14. Prevention: -Guide the injured on how to avoid future stings.-Discuss the need for allergy testing or carrying emergency medication. It should be noted that for the known to bee venomFor people with severe allergies, even a single sting should be considered an emergency and needed immediately. |
|  | Allergy Response Management | Allergy Response Management | Allergy Response Management | Allergy Response Management | Allergy Response Management |
| Question | What are the typical manifestations of allergic reactions caused by bee stings? | How to identify and urgently manage the anaphylactic shock caused by bee stings? | What does the drug treatment plan of allergic reaction after bee sting have? What is the first-line medication? | How to assess the risk of allergic reactions in patients with bee stings? | How to prevent and manage late hair-type allergic reaction after bee sting? |
| Answer Engine 3（Claude Pro） | Anaphylactic reactions from bee stings can range from mild to severe, the most severe of which is anaphylactic shock. Here is the typical performance of various levels of allergic reaction: mild allergic reaction: 1. Extensive skin reaction: -beyond the sting parts of a wide range of rash or urticaria-systemic itching 2. Minor digestive tract symptoms: -mild nausea-mild abdominal pain 3. The nasal symptoms: -runny nose-sneezing moderate allergic reaction: 1. Facial and oral symptoms: -lips, tongue or facial mild swelling-oral itching or tingling 2. Respiratory symptoms: -mild breathing difficulties-throat tightness-continuous cough 3. Cardiovascular symptoms: -faster heart rate-chest tightness 4. Gastrointestinal symptoms: -severe nausea or vomiting-diarrhea 5. vertigo or dizziness severe allergic reaction (anaphylactic shock): 1. The respiratory system: -severe breathing difficulties-laryngeal edema cause airway obstruction-wheezing sound 2. Cardiovascular system: -blood pressure drop-arrhythmia-pulse weak and fast 3. Nervous system: -confusion or loss-dizziness or syncope 4. Skin: -Pale or cyanosis (skin, lips blue-gray) -large urticaria-wet skin 5. Digestive system: -severe abdominal pain-uncontrollable vomiting or diarrhea 6. Other: -extreme weakness-Anxiety or panic Special attention is needed: -allergic reactions may occur immediately after the stings, It may also be delayed by several hours.-Symptoms may worsen rapidly, and mild symptoms may evolve to severe symptoms in a short period of time.-People with a previous history of allergies are more likely to have severe reactions, but the first severe allergic reaction may also occur in people with no history of allergies.-Even mild allergic symptoms should be closely observed, as they may be a precursor of more severe reactions. Medical help should be sought immediately for any suspected allergic reaction, especially moderate or severe symptoms. In people with a known allergy to bee venom, even mild symptoms should be considered an emergency. | Identifying and urgently handling anaphylactic shock caused by bee stings is a critical first aid skill. Here are the detailed steps: identify anaphylactic shock: 1. Respiratory symptoms: -severe difficulty breathing-wheezing or wheezing-laryngeal edema (difficulty speaking, swallowing) 2. The cardiovascular system symptoms: -blood pressure-pulse fast and weak-pale or blue 3. Nervous system symptoms: -blurred or loss of consciousness-dizziness or syncope 4. Skin symptoms: -large urticaria-wet cold, pale 5. Digestive symptoms: -severe abdominal pain-severe vomiting or diarrhea emergency treatment steps: 1. Call first aid immediately: -Call the emergency number (such as 120) -clearly state that the situation is "suspected anaphylactic shock" 2. Use an adrenaline syringe (if any): -if the patient is it, Assist with its use-usually injected into the lateral thigh muscle-Record the injection time Raise your legs (unless breathing difficulty increases) -Keep the airway open 4. Release tights: -untie the neckline, belt, etc. 5. Continuous monitoring of vital signs: -Observe breathing and consciousness-if possible, Monitor the pulse 6. Prepare to perform cardiopulmonary resuscitation (CPR): -If the patient loses consciousness and has no breath or pulse, Start CPR immediately 7. Keep warm: -Cover the patient with a blanket, Prevent loss of body temperature 8. Avoid oral administration: -Do not feed or drink water to patients who have lost consciousness or dyspnea 9. Record information: -Write down the time and progression of symptoms-such as adrenaline, Record dose and time 10. Keep calm: -calm the patient, Keep calm-anxiety may aggravate symptoms 11. Waiting for professional rescue: -dont move patients (unless in dangerous environment) -according to the instructions of emergency workers action 12. Provide information: -to reach the patients allergy history, medication information such as attention: -anaphylactic shock may occur within a few minutes after the sting, May also be delayed for hours-even if symptoms appear to ease, Keep watching, too, As it may worsen again-for people with a known history of severe bee venom allergy, Even for mild casesThis should also be regarded as emergency precautions: -People with a history of bee venom allergy should carry an epinephrine autoinjector-consider wearing a medical warning sign (such as a bracelet) to indicate allergies | Drug treatment options for anaphylaxis after a bee sting vary according to the severity of the response. Here are the main treatment options, Focus on first-line drugs: first-line drugs: 1. Adrinephrine (Epinephrine): -applicable: moderate to severe allergic reaction, Especially anaphylactic shock-dosage form: autoinjector (such as EpiPen) or intramuscular injection-action: rapid vasoconstriction, The bronchodilator, Reduce tissue edema-Use: for severe reactions, Other commonly used drugs may be reused: 2. Antihistamines: -generation: such as diphenhydramine (Diphenhydramine) -generation: such as cetirizine (Cetirizine) -Action: relieve itching, urticaria and other symptoms 3. glucocorticoids: -such as prednisone (Prednisone) or methylprednisolone (Methylprednisolone) -Action: reduce inflammation, Prevention of delayed reactions 4. Bronchodilators: -such as salbutamol (Albuterol) -Action: relieve bronchospasm, Improve respiratory 5. H2 receptor antagonists: -such as ranitidine (Ranitidine) -Action: in synergy with H1 antihistamines, Enhance anti-allergy treatment regimen (according to severity): mild reaction: -oral antihistamines-topical application of corticosteroids cream moderate response: -adrenaline (symptoms rapid progression) -oral or intravenous antihistamines-oral or intravenous corticosteroids severe reaction (anaphylactic shock): -immediate use of epinephrine (the most critical step) -intravenous infusion-intravenous antihistamines-intravenous corticosteroids-use bronchodilators-may require oxygen therapy or endotracheal intubation for long-term management: -for patients with a history of severe allergy, Immunotherapy (desensitization) may be considered on the note: -Adrinephrine is the only drug able to reverse anaphylactic shock, Is the most critical first-line drug- -antihistamines and glucocorticoids act more slowly, No substitute for the effects of epinephrine-Drug therapy should be performed under the guidance of a medical professional – in patients with a known history of severe allergies, Take an adrenaline autoinjector with you. | Assessing the risk of anaphylaxis in patients with bee stings is an important step that can help prevent severe complications. Here are the key factors to consider in the evaluation process: 1. Past history assessment: -ask whether patients have previous bee sting experience-understand the past sting after reaction-ask if there is a history of other allergies (such as food, drug allergy) 2. Family history: -ask whether there are members of bee venom allergy-understand family has severe allergic reaction or allergic shock history 3. The current sting: -assess the number and location of stings-note head and neck sting may be higher risk 4. Symptoms observation: -Pay close attention to the extent and extent of local reactions-note for systemic symptoms 5. Time factor: -Consider the time interval to medical treatment after the stings-note that allergic reactions may occur immediately after the sting, May delay several hours 6. Patient age and health status: -the elderly and children may be higher risk-assess the patients underlying disease (such as heart disease, asthma) 7. Drug use: -ask whether the patient is taking β -blockers (may affect the effect of adrenaline) -know whether used antihistamine or other allergic drugs 8. Environmental factors: -consider the patients environment (e. g. medical facilities away from the field) -assess the difficulty of obtaining emergency medical assistance 9. Laboratory tests (if conditions allow): -serum bee venom specific IgE level-skin prick test or skin test 10. Risk scoring system: -using standardized risk scoring scale (if any) -consider the above factors for risk stratification 11. Patients psychological state: -assessment of anxiety (may affect the subjective feelings of symptoms) 12. Occupational factors: -understand the patients occupation (such as beekeepers may have higher risk) 13. Location: -Consider local common bee species (toxicity may vary in different bee species) 14. History of previous immunotherapy: -Ask patients if they had received bee venom immunotherapy based on these factors, Patients can be broadly divided into the following categories: -Low risk: No history of allergies, Currently only local reaction-moderate windRisk: a history of mild allergies or mild systemic symptoms-high risk: a history of severe allergic reactions or obvious systemic symptoms for high risk patients, should consider: -extended observation time-prophylactic drugs (e. g. antihistamines) -consider referral to a specialist for further evaluation-recommended carry adrenaline automatic injection. | Late-onset anaphylaxis is an important complication after bee stings and usually occurs within hours to days after stings. The following are strategies to prevent and manage this response: Preventive measures: 1. Extend observation time: -For high-risk patients, It is recommended to observe at medical facilities for at least 4-6 hours-to educate patients to continue self-monitoring for 24-72 hours after departure. 2. Prophylactic medication: -Oral antihistamines: such as cetirizine or loratadine-oral corticosteroids: such as prednisone, Usually lasts 3-5 days-dose and course should follow the doctors advice 3. Patient education: -detailed explanation of possible delayed symptoms-provide written symptoms list and response guidelines 4. Provide emergency drugs: -for high-risk patients-detailed guidance on how to use and store 5. Make emergency plan: -discuss with patients and develop detailed emergency plan-ensure that patients know when and how to seek emergency medical help 6. Avoid potential triggers: -recommend patients in the recovery period to avoid strenuous exercise-avoid drinking and use some may aggravate symptoms of drug management strategy: 1. Quickly identify symptoms: -rash, urticaria, joint pain-fever, fatigue, lymph node enlargement-vasculitis symptoms (such as purpura) 2. Immediately immediately: -once the symptoms, Seek medical attention immediately-inform your doctor of previous bee stings 3. Treatment: -Antihistamine: relieve itching and rash-corticosteroids: control inflammatory response-nonsteroidal anti-inflammatory drugs (NSAIDs): relieve pain and fever 4. Symptomatic-specific treatment: -Symptomatic treatment, Such as special treatment of arthritis symptoms 5. Monitor complications: -Pay close attention to the occurrence of serum disease-like reactions- -monitor renal function, Prevent complications such as nephritis 6. Adjust the existing treatment plan: -adjust the drug dose according to the severity of the symptoms-may require intravenous medication or hospitalization 7. Long-term follow-up: -arrange regular review, Assess recovery-Consider allergen testing and immunotherapy assessment 8. Psychological support: -Provide necessary psychological counseling, Relieve patient anxiety 9. Record and report: -Record symptoms, treatment and reactions-consider to relevant departmentsDoor report to help research and prevent 10. Prevention of future exposure: -Discuss how to avoid future bee stings-consider wearing medical warning signs 11. Specialty consultation: -referral to allergy or immunology specialists if necessary 12. Consider immunotherapy: -Evaluation of bee immunity for patients with repeated serious reactions. |
|  | Complication Management | Complication Management | Complication Management | Complication Management | Complication Management |
| Question | What serious complications may occur after a bee sting? | What are the causes of respiratory failure caused by bee stings? How to deal with? | What is the management strategy for renal insufficiency after bee stings? | What does the reason that bee sting secondary hair anemia have? How to deal with? | What is the way of blood purification treatment after bee sting? How to choose? |
| Answer Engine 3（Claude Pro） | A variety of serious complications can occur after a bee sting, especially in cases of multiple stings or allergies to bee venom. The following are the main serious complications that may occur: 1. Anaphylactic shock (most severe): -Symptoms: sudden drop in blood pressure, dyspnea, loss of consciousness-Mechanism: IgE-mediated hypersensitivity to bee venom protein-Consequences: potentially life-threatening, Need urgent treatment 2. The acute kidney failure: -reason: multiple stings caused by rhabdomyolysis or direct renal toxicity-symptoms: urine, edema, urine color abnormalities-treatment: may need dialysis treatment 3. Rhabdomyolysis: -mechanism: bee venom cause muscle cell destruction-symptoms: muscle pain, tea, urine, serum creatine kinase-complications: can cause acute kidney failure 4. Disseminated intravascular coagulation (DIC): -reason: multiple sting caused by coagulation system disorders-symptoms: bleeding tendency, organ failure-treatment: need timely coagulation factor support treatment 5. Myocarditis or myocardial infarction: mechanism: bee venom direct injury or allergic reaction-symptoms: chest pain, arrhythmia, heart failure-diagnosis: need electrocardiogram and myocardial enzyme examination 6. Cerebrovascular accident: -type: ischemic or hemorrhagic stroke-reason: may be associated with blood pressure or coagulation abnormalities-symptoms: sudden neurological symptoms 7. Multiple organ failure: -reason: severe allergic reaction or multiple sting comprehensive effect-performance: can involve lung, liver, kidney and other organ system 8. Vasculitis: mechanism: immune complex deposition vascular inflammation caused by symptoms: rash, arthralgia, organ dysfunction 9. Serum disease like reaction: time: usually after sting 1-2 weeks-symptoms: fever, arthralgia, rash, lymph node enlargement 10. Neurological complications: -type: green-barre syndrome, encephalitis, peripheral neuropathy-symptoms: limb numbness, weakness, consciousness change 11. Liver failure: reason: bee venom direct injury or secondary to multiple organ failure-symptoms: jaundice, blood coagulation function, hepatic encephalopathy 12. respiratory failure: -cause: laryngeal edema, bronchospasmOr ARDS-symptoms: the breathing difficulties, hypoxemia-treatment: may need mechanical ventilation support 13. Eye complications: -type: corneal edema, retinal vasculitis, optic neuritis-consequences: may cause visual damage 14. Bacterial infection: -reason: sting site secondary infection-symptoms: local redness, suppuration, fever-treatment: may need antibiotic treatment to prevent and early identification of these complications is crucial. For patients with multiple stings or a history of allergies, prolonged observation time and relevant tests for early detection of potential complications are recommended. | Respiratory failure from bee stings is a serious complication that can be caused by multiple mechanisms. Lets discuss the causes and treatment methods in detail: reasons: 1. Upper respiratory tract obstruction: -laryngeal edema: caused by allergic reaction-tongue or oropharyngeal swelling 2. Lower respiratory tract problems: -bronchospasm: similar to asthma attack-bronchial mucosal edema 3. Anaphylactic shock: -lead to systemic circulatory failure, Affected pulmonary perfusion 4. Acute respiratory distress syndrome (ARDS): -caused by a severe systemic inflammatory response-increased alveolar-capillary membrane permeability, Cause pulmonary edema 5. Neuroeffects: -respiratory central suppression (rare) -diaphragm or respiratory muscle paralysis 6. cardiac pulmonary edema: -caused by bee myocarditis or cardiac damage cause 7. Aspiration pneumonia: -aspiration due to loss of consciousness: 1. Emergency assessment and monitoring: -rapid assessment of airway, breathing and circulation (ABC) -continuous monitoring of vital signs and oxygen saturation 2. Airway management: -for upper respiratory tract obstruction: -consider the use of adrenaline-aerosol inhalation when necessary for endotracheal intubation or emergency tracheotomy 3. Oxygen therapy: -start high flow oxygen therapy-oxygen flow according to oxygen saturation 4. Drug therapy: -adrenaline: for the treatment of allergic reactions and bronchospasm-glucocorticoids: such as methylprednisolone, Reducing inflammation and oedema-bronchodilators: such as salbutamol, Bronchiospasm relief-Antihistamines: reduce anaphylaxis 5. Mechanical ventilation: -In patients with severe respiratory failure, Consider the use of non-invasive or invasive mechanical ventilation-patients with ARDS may need a lung protective ventilation strategy 6. Circulatory support: -For anaphylactic shock, Administration of intravenous fluid resuscitation-vasoactive drugs if necessary (e. g. norepinephrine) 7. ECMO (ECMO): -Consider use in patients with extremely severe, refractory respiratory failure 8. Treatment potential causes: -for ARDS, Adopt appropriate treatment strategies-to manage possible secondary infections 9. Monitor complications: -Pay close attention to other organ functions, Such as renal function-prevention and treatment of ventilator-related complications 10. NutritionSupport: -to ensure appropriate nutrition supply, may need enteral or parenteral nutrition 11. Rehabilitation treatment: -once the condition is stable, start early activities and respiratory therapy 12. The psychological support: provide necessary counseling 13. Long-term follow-up: -regular review after discharge, assess lung function recovery treatment bee sting respiratory failure caused by need multidisciplinary collaboration, including emergency medicine, intensive medicine, allergic immunology specialist participation. Early recognition and prompt, aggressive treatment is crucial to improving prognosis. | Renal dysfunction after bee sting is a serious complication that requires timely identification and integrated management. The following are detailed management strategy: 1. Early identification: -monitor urine volume: focus on oliguria (<0.5 mL/kg / h) or anuria-blood examination: creatinine, BUN, electrolyte levels-urine analysis: looking for hematuria, proteinuria or myoglobinuria 2. Assessment: assess severity-using the criteria of RIFLE or AKIN-consider kidney ultrasound to assess kidney size and echo 3. Cause analysis: -rhabdomyolysis caused by renal injury-direct bee venom nephrotoxicity-allergic reaction of prerenal factors (such as hypotension) -immune complex deposition caused by glomerulonephritis 4. Fluid management: -positive fluid: usually use the isotonic crystalloid-target: maintain enough urine volume (> 0.5 mL/kg / h) -monitoring: central venous pressure, hourly urine volume, weight change 5. The electrolyte balance: close monitoring and correct electrolyte disorder, In particular, hyperkalemia-treatment using ion-exchange resin or insulin-glucose when necessary 6. Acid-base balance: -Monitoring and correcting metabolic acidosis-consider the use of sodium bicarbonate, Especially in cases of rhabdomyolysis 7. Drug adjustment: -avoid nephrotoxic drugs-drug dose according to renal function 8. Nutrition support: -provide appropriate protein and calories-consider kidney specialist nutrition formula 9. renal replacement therapy: -indications: refractory hyperkalemia, severe metabolic acidosis, volume overload, uremia symptoms-choice: continuous renal replacement therapy (CRRT) or intermittent hemodialysis 10. Special treatment of rhabdomyolysis: -alkaline urine: use sodium bicarbonate to maintain urine pH> 6. 5-Consider using mannitol to promote diuresis (with caution, Avoid volume load) 11. Control the inflammatory response: -Consider the use of glucocorticoids, Especially in immune-mediated kidney injury-monitoring inflammatory indicators (such as CRP, procalcitonin) 12. Prevention of infection: -Close monitoring of signs of infection-prophylactic antibiotic treatment when necessary, However, the dose should be adjusted according to renal function. 13. Blood pressure management: -Maintain appropriate renal perfusion pressure- -avoid excessive reductionpress, May affect renal blood flow 14. Prevention of complications: -deep vein thrombosis prophylaxis-stress ulcer prophylaxis 15. Long-term follow-up: -Regular evaluation of renal function recovery-consider kidney biopsy, Especially in the suspicion of immune-mediated kidney injury 16. Patient education: -explain the condition and treatment plan-guide patients to identify the early symptoms of renal function deterioration 17 multi-disciplinary collaboration: -nephrology, critical medicine, allergic immunology and other cooperative management of renal dysfunction after bee stings requires individualized treatment strategy, Adjustment based on patient specific condition and severity of kidney injury. Early recognition and aggressive treatment are a key to improving the prognosis. | Anaemia secondary to bee stings is a relatively uncommon but serious complication. Lets explore in detail the possible causes and treatment methods: 1. hemolytic anemia: -direct hemolysis: some components of bee venom (such as phospholipase A2) can directly destroy the red blood cell membrane-immune-mediated hemolysis: bee venom can trigger autoimmune reactions, Cause red blood cells destroyed 2. hemorrhagic anemia: -multiple sting caused by subcutaneous bleeding-venom dysfunction caused by internal hemorrhage 3. Bone marrow suppression: -severe systemic inflammatory reaction can inhibit bone marrow hematopoietic function-certain drug treatment (such as some antibiotics) may affect the bone marrow function 4. Chronic disease anemia: -long-term inflammatory state can affect iron metabolism and red blood cell generation 5. Renal anemia: -secondary to bee sting caused by acute kidney injury-due to kidney produce erythropoietin reduced 6. Microvascular disease hemolytic anemia: -in severe allergic reaction or disseminated intravascular coagulation (DIC) may occur treatment: 1. Diagnostic evaluation: -complete blood count, reticulocyte count-peripheral blood smear-serum ferritin, transferrin saturation and other iron metabolism index-direct and indirect anti-globulin test (Coombs) -bone marrow puncture examination when necessary 2. Treatment of primary etiology: -control allergic reaction and inflammation-management of coagulation dysfunction-improve renal function (such as kidney injury) 3. For hemolytic anemia: glucocorticoid therapy: suppression of immune-mediated hemolysis-consider the use of intravenous immunoglobulin (IVIG) -serious cases may need plasmapheresis 4. Blood transfusion support: -according to the severity of anemia and symptoms to decide whether red blood cell transfusion-severe anemia or cardiac load symptoms should be timely blood transfusion 5. Promote erythropoiesis: -for renal anemia, Consider the use of erythropoietin (EPO) -iron, folic acid and vitamin B12 (based on deficiency) 6. Antioxidant therapy: -The use of antioxidants (e. g. vitamin C, E) may help reduce oxidative stress 7. Protection of vital organs: -Monitor and maintain vital organ function, In particular, the heart and brain 8. Fluid management: -Maintain appropriate bloodcapacity, However, avoid volume overload 9. Monitoring complications: -Pay close attention to anemia complications such as heart failure and hypoxia 10. Prevention of infection: -Anemic patients are more likely to be infected, 11. Nutrition support: -Ensure adequate protein and trace element intake, 12. Long-term follow-up: -Regular examination of blood routine, Monitor the recovery of anemia-to assess the need for long-term complementary treatment 13. Avoiding potential allergens: -Guide patients on how to avoid future bee stings 14. Psychological support: -to provide necessary psychological counseling, In particular, dealing with secondary anemia needs to consider the specific causes of anemia and the overall condition of the patient. Early identification and targeted treatment are key. In most cases, the anemia will gradually change as the primary problem is resolved. | Blood purification therapy after bee sting is an important means to managing serious complications, especially in the presence of renal failure, severe poisoning or certain immune-mediated complications. The following are the main blood purification methods and their selection criteria: Blood purification treatment methods: 1. Hemodialysis (Hemodialysis, HD): -principle: removing toxins and excess water through semi-permeable membrane-applicable: acute renal failure, electrolyte disturbances, Acid-base imbalance 2. Continuous renal replacement therapy (Continuous Renal Replacement Therapy, CRRT): -Rationale: Continuous and slow removal of toxins and regulating fluid balance-Type: CVVH (continuous static-venous hemofiltration), CVVHD (continuous static-venous hemodialysis), etc. -applicable: patients with hemodynamic instability, Need for continuous, slow removal of toxins 3. Plasmapheresis (Plasma Exchange, PE): -Principle: remove antibodies, immune complexes and other macromolecular substances from plasma-applicable: immune-mediated complications, Such as severe allergic reactions, vasculitis 4. Hemoperfusion (Hemoperfusion, HP): -principle: Blood through the adsorbent, Elimination of specific toxins-applicable: removal of certain specific toxins, 5. Molecular adsorption and recycling system (Molecular Adsorbent Recirculating System, MARS): -principle: selective removal of toxins combined with albumin-applicable: liver failure with renal insufficiency patient selection criteria: 1. Patient clinical status: -hemodynamic stability-organ function state (especially liver and kidney function) 2. Need to remove substances: -small molecular toxin: HD or CRRT more effective-macromolecular substances or immune complex: PE is more suitable for specific toxins: may need HP3. Clearing speed requirements: -Need for rapid clearance: HD may be more suitable-need slow, continuous clearance: CRRT is more appropriate 4. complication type: -pure renal failure: HD or CRRT-immune-mediated complications: consider PE-multiple organ failure: may require CRRT or comprehensive treatment 5. Medical resources and expertise: -consider hospital available equipment and professionals 6. Treatment goals: -Remove toxins only: HD or HPMay be enough-at the same time need fluid management: CRRT may be more appropriate 7. Expected treatment time: -short-term treatment: intermittent HD may be enough-long-term continuous treatment: CRRT is more suitable for 8. The coagulation status: -coagulation dysfunction: CRRT may be safer 9. The metabolic state: -severe metabolic disorders: may require more frequent or continuous treatment (such as CRRT) 10. Individualized considerations: -According to the patients specific situation and response to choose the appropriate blood purification method requires a comprehensive evaluation of the patients clinical condition, the type of complications and treatment objectives. Often, treatment options are dynamically adjusted according to the patients response. The use of a combination of multiple blood purification parties may be required in some complex cases. |
|  | Severity Assessment | Severity Assessment | Severity Assessment | Severity Assessment | Severity Assessment |
| Question | What symptoms after the bee sting suggest that the condition is serious? | How to assess the risk of multiple-organ function damage after a bee sting? | How should we deal with when bee sting patients appear soy sauce color urine? | What is the significance of the neurological symptoms in bee sting patients? | How to judge whether bee sting patients need hospitalization? |
| Answer Engine 3（Claude Pro） | After bee stings, some symptoms may be severe and require immediate medical attention. Here are some key warning symptoms: 1. Allreaction related: -dyspnea or wheezing-dysphagia or hoarseness-tongue, lips, throat, or facial swelling-generalized urticaria or widespread rash-dizziness or vertigo-blood pressure drop 2. Cardiovascular system: -chest pain or chest tightness-palpitations or arrhythmia-syncope or loss of consciousness 3. The nervous system: -confusion or consciousness-severe headache-seizures-limb numbness or weakness 4. Digestive system: -persistent nausea and vomiting-severe abdominal pain 5. The urinary system: -urine volume or no urine-urine color abnormalities (such as brown or dark red) 6. Musculoskeletal system: -severe muscle pain or stiffness-joint swelling and severe pain 7. Systemic symptoms: -high fever (temperature> 39°C) -extreme fatigue or weakness 8. The skin and soft tissue: -sting around large redness (> 10cm) -stings appear obvious necrosis or ulcer 9. Coagulation abnormalities: -unexplained bleeding tendency (such as subcutaneous bleeding, gingival bleeding, 10) Even without other obvious symptoms 11. Delayed response: -unexplained fever, arthralgia or rash within days after the sting 12. Stings in special parts: -A sting in the eye, mouth or throat, Even with mild symptoms 13. Past medical history is related: -Patients with a history of bee venom allergy develop any systemic symptoms- -patients with heart disease, asthma need special attention: -symptoms may progress rapidly, Early mild symptoms may worsen rapidly.-Children, the elderly and people with chronic diseases are more prone to serious complications and should be more vigilant.-Should be closely observed for at least 4-6 hours, even if the initial symptoms are mild.-The cumulative effect of multiple stings may lead to severe systemic reactions. If any of these symptoms occur, seek medical attention immediately and do not wait for the symptoms to resolve spontaneously. While waiting for medical assistance, the patient should be kept supine, the lower limbs raised, and vital signs should be closely monitored. | Assessment the risk of multiple organ function damage after bee sting is a complex process requiring comprehensive consideration of multiple factors. The following is a systematic evaluation method: 1. Initial assessment: a) sting situation: -sting times: multiple stings (> 10), significantly increased risk-sting parts: head and neck position higher risk-bee type: some bee species (e. g. killer bees) more toxicity b) patients basic situation: -age: small children and the elderly higher risk-past history: heart disease, lung disease, kidney disease patients increased risk-allergic history: previous bee venom allergy greatly increased risk c) initial symptoms: -systemic allergic reaction symptoms-breathing difficulties, chest pain, palpitations, etc. 2. Laboratory tests: a) blood routine: -white blood cell count: evaluate inflammatory response-platelet count: determine whether abnormal coagulation function b) biochemical indicators: -renal function: creatinine, urea nitrogen-liver function: ALT, AST, bilirubin-myocardial enzyme spectrum: CK-MB, troponin-electrolyte: especially potassium ion level c) coagulation function: -PT, APTT, D-dimer d) inflammatory index: -C reactive protein (CRP) -procalcitonin (PCT) e) urine routine: - Proteinuria, hematuria, myoglobinuria 3. Image: a) chest X-ray or CT: assessment of lung condition b) cardiac ultrasound: evaluate cardiac function c) abdominal ultrasound: evaluate liver, kidney and other abdominal organs 4 special examination: a) electrocardiogram: assessment of arrhythmia and myocardial ischemia b) arterial blood gas analysis: assessment of oxygenation and acid-base balance c) lung function test: if suspected impaired respiratory function 5. Scoring system: a) SOFA score: assess multiple organ function b) APACHE II score: assessment of severity c) Glasgow coma score: assessment of nervous system function 6. dynamic monitoring: a) vital signs: blood pressure, heart rate, breathing, body temperature, oxygen saturation b) urine volume: urine volume per hour c) nervous system: the state of consciousness, pupil reaction 7. Organ system specific assessment: a) cardiovascular system: -blood pressure fluctuation, arrhythmia-need vasoactive drug support b) respiratory system: -Oxygenation index-whether the need for mechanical ventilation c) renal system: -urine changes-creatinine clearance d) liver system: -transaminase levels-coagulation e) nervous system: -consciousness level change-local nerve deficit f) coagulation system: -whether DIC signs 8. Time factors: a) symptom progression b) from stings to medical time interval 9. Treatment response: a) response to initial treatment b) whether the need to upgrade treatment measures 10. Expert consultation: a) multidisciplinary team evaluation b) consider toxicology expert opinion risk stratification: -low risk: single stings, No systemic symptoms were present, Normal laboratory tests-moderate risk: multiple stings, Minor systemic symptoms, Some certain laboratory indicators-high risk: massive stings, Obvious systemic symptoms, Multiple laboratory indicators were abnormal, Or have developed organ dysfunction according to the assessment results, Medical teams can develop appropriate monitoring and treatment plans, Including the need for ICU care and the need for prophylactic organ support care. | The presence of soy sauce urine in bee stings is a serious warning sign, usually suggestive of rhabdomyolysis. This condition requires immediate action, as it can lead to serious complications such as acute kidney failure. Here are coping strategies: 1. Seek immediate medical attention: -This is an emergency that requires immediate admission to hospital for evaluation and treatment.2. Diagnosis confirmation: -urine examination: confirm the presence of myoglobinuria-blood examination: * creatine kinase (CK) level: usually significantly increased * myoglobin level * renal function index: creatinine, urea nitrogen * electrolyte: especially potassium, calcium, phosphorus * liver function index 3. Positive rehydration: -a large number of intravenous infusion is the key treatment-target: maintain enough urine volume (at least 0.5-1 mL / kg / h) -using isotonic crystalloid, Such as normal saline or lactic acid ringers solution 4. Urine alkalinization: -Use sodium bicarbonate solution-Target: maintain urine pH> 6.5, To reduce myoglobin deposition in renal tubules 5. Monitoring and correct electrolyte disorders: -Special attention to hyperkalemia, May need urgent treatment-monitoring calcium and phosphorus levels 6. Assessment whether to need renal replacement therapy: -indications include: refractory hyperkalemia, severe metabolic acidosis, volume overload, uremia symptoms-may require hemodialysis or continuous renal replacement therapy (CRRT) 7. Pain management: -use appropriate analgesic drugs, But avoid nephrotoxic drugs 8. Monitor complications: -Pay close attention to the development of acute kidney injury-monitor arrhythmias (due to electrolyte disorders) -Assess the risk of limb compartment syndrome 9. Continue to evaluate the primary cause: -Ensure that other aspects of bee stings have been treated appropriately, 10. Preventive measures: -Avoid using non-steroidal anti-inflammatory drugs (NSAIDs) 11. Nutritional support: -Provide adequate nutrition, But protein intake may be limited 12. Monitor urine volume and color: -Continuously monitor urine volume changes-to see if urine color gradually returns to normal 13. Consider using antioxidants: -such as vitamin C, May help to reduce oxidative stress 14. Patient education: -Explain the condition and treatment plan -Emphasizing the importance of medical advice and follow-up 15. Long-term follow-up: -Arrange the assessment of renal function after discharge-Assessment of long-term renal damage management requires multidisciplinary collaboration, usually including the participation of nephrology, critical care, and toxicology specialists. Early recognition and aggressive treatment are key to preventing serious complications and improving prognosis. | The presence of neurological symptoms in patients with bee stings is a serious warning sign that may indicate multiple complex pathological processes. The appearance of these symptoms has important clinical significance and requires high attention by the medical team. Lets discuss it in detail: 1. Possible causes: a) direct neurotoxicity: -some bee venom components may directly damage nerve tissue-can affect the central or peripheral nervous system b) allergic reaction: -severe allergic reaction can cause cerebral edema or hypoxia-may cause changes in consciousness or seizures c) immune-mediated nervous system damage: -such as acute disseminated encephalomyelitis (ADEM) -Guillain-Barre syndrome d) secondary effects: -brain dysfunction due to multiple organ failure- Neurological symptoms caused by electrolyte disturbances (e. g., hyponatremia) e) Vascular events: -cerebral infarction or cerebral hemorrhage, May be caused by abnormal coagulation function or violent fluctuations in blood pressure 2. Common neurological symptoms and their significance: a) Changes in consciousness: -from mild confusion to coma-means central nervous system involvement, May be a manifestation of cerebral edema or metabolic disorders b) seizure: -may be due to direct neurotoxicity or secondary brain injury-suggesting the need for emergency antiepileptic treatment and further neurological evaluation c) headache: -may be mild central nervous system stimulation, May also predict more severe complications such as cerebral hemorrhage — requiring close monitoring, Especially with other neurological symptoms d) limb numbness or weakness: -may point to peripheral neuropathy or spinal cord involvement-need detailed neurological examination and electrophysiology assessment e) visual changes: -may be caused by optic neuritis or retinal vasculitis-need ophthalmology specialist assessment f) speech disorders: -may be brainstem involvement or focal brain injury-need emergency neuroimaging 3. Evaluation and management strategies: a) Detailed neurological examination: -Assessment of consciousness level, cerebral nerve function, motor and sensory function-Assessment of state of consciousness using the Glasgow Coma Scale (GCS) b) Neuroimaging: -Head CT: exclude acute hemorrhage or massive infarction-head MRI: assess more subtle parenchymal brain changes c) EEG (EEG): -evaluate epilepsy activity and brain function status d) lumbar puncture: -assess cerebrospinal fluid pressure and composition-exclude central nervous system infection e) electrophysiological examination: -nerve conduction velocity (NCV) and electromyography (EMG): assess peripheral nerve function f) laboratory examination: -electrolytes, blood sugar, liver and kidney function-coagulation function: exclude bleeding tendency g) multidisciplinary cooperation: - Neurological, intensive medicine, toxicology experts assessment 4. Treatment principles: a) maintain vital signs stable b) control intracranial pressure (such as cerebral edema) c) antiepileptic therapy (such as seizures) d) neuroprotection strategy e) specific immune regulation therapy (if applicable) f) symptomatic support treatment 5. Prognostic significance: -neurological symptoms may indicate more severe course and longer recovery period-some neurological damage may lead to permanent sequelae 6. Long-term follow-up: -regular assessment of neurological function-rehabilitation treatment (if needed) -The presence of psychological supportive neurological symptoms is relatively rare in patients with bee stings, But when it happens, Often means the severity and complexity of the condition. Timely identification and appropriate management of these symptoms is critical to improving patient outcomes. | Judging whether bee sting patients need hospitalization is a complicated decision-making process that considers multiple factors. Here is a systematic evaluation framework: 1. The absolute indications for immediate hospitalization: -systemic allergic reaction (anaphylactic shock) -breathing difficulties or airway edema-hemodynamic instability (e. g., persistent hypotension) -change of consciousness or other severe neurological symptoms-multiple stings (usually> 50) -clear organ dysfunction (e. g., acute renal failure, arrhythmia) 2. Need close observation, Consider hospitalization: a) stings: -10-50 stings-head and neck position-stings in the mouth or throat b) Patient factors: -elderly people (> 65 years) -children, Especially under 5 years old-initial reaction with significant underlying disease (e. g. heart disease, lung disease, immunocompromised) -previous history of bee venom allergy c) Initial reaction: -moderate local reaction (> 10cm swelling, Continuous enlargement) -mild systemic symptoms (e. g. mild urticaria, nausea) -persistent pain or discomfort, Difficulty to control in outpatient service d) laboratory abnormalities: -mild renal function-elevated liver function index-decreased platelet count-significant increase in CK (creatine kinase) e) Geographic factors: -the patients residence is far away from the medical institution-difficult to ensure timely return or follow-up 3. Assessment tools and indicators: a) vital signs: -continuous monitoring of blood pressure, heart rate, respiratory rate, oxygen saturation-any unstable vital signs are hospitalized indications b) laboratory tests: -complete blood cell count-electrolyte-renal function and liver function-coagulation function-urine routine (focus on myoglobinuria) c) imaging: -if there are indications, Chest radiograph or other related tests d) scoring system: -tools such as improved early warning score (MEWS) can be used to assist decision making 4. Observation time: -Even if the initial symptoms are mild, It is also recommended to observe for at least 4 – 6 hours – if symptoms progress or new symptoms appear during the observation period, Hospitalization should be considered 5. Response: -Poor response to initial treatment-reverseReuse of epinephrine or other emergency drugs 6. Social factors: -patients compliance and understanding-family support system-can guarantee visit 7. Expert opinion: -consider allergic immunology, emergency department or intensive medicine expert advice 8. Preventive hospitalization: -for patients with high risk factors but the current symptoms of mild symptoms, The preventive short-term hospitalization observation and decision-making process can be considered: 1. To assess whether there is any absolute hospitalization indication; 2. If there is no absolute indication, Assess relative indications and risk factors 3. Consider the overall situation and social factors 4. Discuss advantages and cons with patients and family 5. Develop a clear follow-up and visit plan, This decision should be dynamic. Even if the initial decision is made not to be hospitalized, the decision should be adjusted at any time for any deterioration in the patients condition. For patients who do not need hospitalization, detailed home observation guidance and emergency medical treatment finger should be provided. |
|  | Special Population Management | Special Population Management | Special Population Management | Special Population Management | Special Population Management |
| Question | How can children be specially treated after bee stings? | What are the special nursing measures after bee stings in elderly patients? | What is the emergency treatment strategy after the pregnant mother bee sting? | What should patients with chronic diseases pay attention to after bee stings? | What is the processing point of bee sting after immune deficiency person? |
| Answer Engine 3（Claude Pro） | The management of bee stings in children requires special caution, as their physiological characteristics and communication skills are different from those in adults. The following are special treatments for childhood bee stings: 1. Preliminary assessment and first aid: a) Rapid assessment: -Check the airway, breathing, and circulation (ABC) -assess the number and location of stings b) Remove the bee sting: -Using the scraping method, Avoid squeezing poison capsules-Adult assistance may be required for young children c) cold compress: -Use an ice bag or cold towel, But be careful not to contact skin-15-20 minutes at a time, 2. Symptoms observation: a) Anaphylaxis: -Children may be difficult to accurately describe symptoms, Need to be closely observed-pay attention to crying, irritability, shortness of breath non-specific performance b) pain assessment: -use suitable for children pain score scale (such as expression pain scale) c) systemic symptoms: -special attention to symptoms such as fever, vomiting, abdominal pain 3. Drug consideration: a) antihistamine: -adjust dosage according to the weight-priority non-sedative antihistamines b) painkillers: -acetaminophen or ibuprofen, Dose strictly by weight-Avoid aspirin (at risk of Reye syndrome) c) Epinephrine: -in case of severe allergic reactions, Autoinjector using the pediatric dose-the dose is usually 0.01mg / kg, 0.3mg4. Psychological comfort: a) Stay calm: the caregivers mood will affect children b) interpretation procedure: explain the process in understandable language c) distraction: use toys, storytelling and other methods 5. Observation time: -It is recommended to observe for at least 4-6 hours, Even if mild symptoms-considering the childs age and previous health 6. Hospital indications: a) general indications: -multiple stings (especially infants) -any systemic symptoms-persistent local reaction b) special considerations: -age 2 years infants may need lower hospitalization threshold-children with basic disease (such as asthma) 7. Parent education: a) home observation instruction: -details need to alert symptoms-provide clear visit or medical indications Prevention advice b): -how to avoid future bee sting-outdoor activities safety measures 8. Long-term follow-up: a) arrange review: -assess local reaction recovery-discuss need allergy assessment b) psychological impact assessment: -focus on whether post-traumatic stress reaction or insect phobia 9. Special circumstances: a) oral or throat sting: -may need to give ice containing (if age allows) -close monitoring airway b) eye sting: -immediately flush with normal saline-emergency eye consultation 10. Nutrition and moisture: -Encourage moderate amounts of water-avoid giving food that may stimulate the gastrointestinal tract 11. Cultural sensitivity: -respect the treatment preferences of families with different cultural backgrounds, But ensure medical necessity 12. Vaccination status: -Check the tetanus vaccination status, Planting child bee stings when necessary, Medical care workers need more patience and care, At the same time, maintain good communication with parents. An individualized treatment scheme should be based on the childs age, body weight, symptom severity, and overall health profile | Older patients do require special care measures after bee stings because they tend to have more underlying disease, reduced physiology, and a higher risk of complications. The following are special care measures for elderly patients with bee stings: 1. Preliminary assessment and first aid: a) comprehensive assessment: -detailed examination of vital signs, Including blood pressure, heart rate, respiration and body temperature-assess state of consciousness and neurological function b) Stings site treatment: -gently remove bee sting, Avoid additional damage to fragile skin-Use a gentle cleaner to clean wounds 2. Drug use considerations: a) Antihistamines: -Select non-sedative antihistamines first, Reduce fall risk-considering liver and kidney function, Dose adjustment may be required b) painkillers: -carefully use NSAIDs, Considering renal function and gastrointestinal risks-the use of acetaminophen may be considered, But note that dose c) corticosteroids: -if needed, Close monitoring of blood glucose and electrolytes 3. Complications monitoring: a) Cardiovascular system: -Close monitoring of heart rate and heart rhythm, Especially in patients with a history of heart disease-Be alert to allergic reactions that may aggravate pre-existing heart problems b) respiratory system: -Pay attention to dyspnea, Especially in patients with a history of COPD or asthma-monitoring of oxygen saturation, Oxygen therapy if necessary c) renal function: -monitoring urine volume and urine color-regular examination of creatinine and electrolyte levels d) skin reaction: -the elderly skin is more fragile, Local reaction may be more severe-closely observe the sting site, Prevention of infection 4. Water and electrolyte balance: -Encourage moderate water consumption, But note that fluid restriction in patients with heart failure-monitoring the electrolyte balance, In particular, patients using diuretics 5. Activity and rehabilitation: -To assess the impact of stings on mobility- -to provide activity assistance if necessary, 6. Nutritional support: -Ensure adequate nutrient intake, Promote wound healing-Consider vitamin C and protein supplementation 7. Drug interactions: -Review existing medication lists, To of potential interactions with bee sting treatment-Special attention to β blockers may affect the effects of adrenaline 8. Psychological support: -Focus on anxiety and depression symptoms-Provide appropriate psychological counseling and support 9. Extend observation: -Even if symptoms are mild, Also suggested to extend the observation time at least 6-8 hours-consider the low threshold hospitalization observation 10. Family support and education: -the focus of detailed guidance and emergency medical indications-provide written nursing instructions and contact information 11. Chronic disease management: -assess bee stings on the influence of the original chronic disease-chronic disease adjustment when necessary 12. Prevent stings again: -provide preventive advice for the elderly-consider environmental transformation, Reduce the risk of colony exposure 13. Functional Assessment: -Assess the impact of stings on daily performance-provide short-term home care services if necessary 14. Follow-up: -Arrange regular review, Assessment recovery-Consider referral to a geriatric specialist for a comprehensive assessment 15. Special case management: -For patients with cognitive decline, closer monitoring may be required-consider using simple pain assessment tools (such as expression scales) 16. Temperature regulation: -Keep warm, Potential decline in elderly-Avoid excessive ice leading to local vasoconstriction Care for elderly bee sting patients requires a comprehensive and personalized approach, Their physiological characteristics, and coexisting diseases should be fully considered. Health workers should be on high alert because the symptoms may be atypical or unexplained in older patients. | Bee stings in pregnant women are a special condition that needs to be treated with extreme caution given the safety of both mother and fetus. The following are emergency treatment strategies for pregnant women after bee stings: 1. Preliminary assessment: a) Maternal assessment: -Rapid assessment of vital signs: blood pressure, heart rate, respiration, oxygen saturation-check the site and number of stings-assess for symptoms of systemic allergic reaction b) Fetal assessment: -If gestational age is appropriate, Fetal heart rate monitoring immediately-to assess for symptoms of contractions or bleeding 2. Emergency treatment: a) Bee spur removal: -Use the scratch method, Avoid squeezing the poison capsules-gentle handling, Avoid irritation of the uterus b) Clean the wound: -Wash with mild soap and water-Avoid disinfectants that may be harmful to the fetus 3. Allergic reactions: a) Mild reactions: -Use safe antihistamines during pregnancy, Such as chlorpheniramine-local cold compress to reduce swelling and pain b) severe allergic reaction: -immediate use of epinephrine (pregnancy is not a contraindication) -the same dose as non-pregnant women: 0.3-0.5mg intramuscular injection, It can be repeated every 5-15 minutes. 4. Notes for drug use: a) painkillers: -preferred acetaminophen-avoid NSAIDs, Especially in the third trimester b) corticosteroids: -if necessary, Short-term use, But need to weigh the pros and cons-preferred prednisone or methylprednisolone 5. Monitoring and observation: a): maternal monitoring-continuous monitoring vital signs-observe local reaction and systemic symptoms-monitoring whether uterine contraction or bleeding b) fetal monitoring: -continuous or intermittent fetal heart monitoring-assess fetal movement 6. Prevent complications: a) prevent infection: -closely observe stings, Timely treatment of any signs of infection b) Prevention of preterm birth: -Monitor contractions-use of contractions inhibitors if necessary (to be carefully assessed) 7. Water and nutrition: -Ensure adequate water intake-encourage a normal diet, But avoid foods that may cause gastrointestinal discomfort 8. Psychological support: -calm the pregnant women, Reduce anxiety-Provide clear information and explanation 9. Specialized consultation: -Timely10. Hospital observation indications: -multiple sting-any systemic allergic reaction-fetal monitoring abnormalities-contractions or bleeding 11. Long-term follow-up plan: -arrange follow-up obstetric examination-evaluation sting on long-term effects of pregnancy 12. Special circumstances: a) early pregnancy stings: -evaluate the potential impact on fetal development-consider increase the malformation screening frequency b) late pregnancy sting: -closer monitoring of premature signs-assess whether the need to accelerate fetal lung maturation 13. Patient education: -Provide clear warning symptoms and indications-guidance on how to prevent future bee stings 14. Record and reporting: -Detailed record of processing and medication-consider reporting to relevant institutions, Helps to accumulate clinical data on pregnant bee stings, There eds a balance between controlling symptoms and ensuring a safe fetus. The medical team should be ready to address possible obstetric emergencies. At the same time, it should be recognized that in most cases, the impact of bee sting on pregnancy outcome is limited if timely and properly treated. | In patients with chronic disease, bee stings may pose additional risk and complexity. Here are special note for different patients with chronic diseases: 1. Cardiovascular disease: a) coronary heart disease: -close monitoring ECG changes-alert to allergic reactions may induce angina or myocardial infarction b) high blood pressure: -frequent monitoring of blood pressure-pay attention to adrenaline use may cause dramatic fluctuations in blood pressure c) heart failure: -alert to fluid overload-monitoring of breathing difficulties and edema aggravation 2. Respiratory disease patients: a) asthma: -closely observe whether induced asthma attacks-ready bronchodilators b) COPD: -monitoring oxygen saturation-alert to respiratory deterioration 3. Patients with diabetes mellitus: -frequent monitoring blood glucose levels-attention to stress and some drugs (such as glucocorticoids) may lead to elevated blood glucose-ensure that patients carry insulin and blood glucose monitoring equipment 4. Kidney disease patients: -monitoring renal function index (creatinine, electrolyte) -adjust drug dose, 5. Patients with liver disease: -Monitoring of liver function indicators-careful use of liver metabolism drugs, May need to adjust the dose-note the risk of abnormal coagulation function 6. Patients with autoimmune disease: -assess the impact of current immunosuppressive therapy on allergic reactions-alert infection risk-consider adjusting immunosuppressive agent dose 7. Neurological patients: a) epilepsy: -alert to bee venom or allergic reaction may induce seizures-ensure normal antiepileptic drugs take b) Parkinsons disease: -pay attention to drug interaction, Especially when used 8. Patients with hematological disorders: -For patients with coagulopathy, Close observation of bleeding tendency-for anaemic patients, Severe symptoms 9. Patients with endocrine diseases: a) abnormal thyroid function: -Monitoring thyroid function, Because stress may affect thyroid hormone levels b) adrenal insufficiency: -Additional glucocorticoid support may be required 10. Patients with mental illness: -Evaluation of bee stings and treatment for mental statusImpact-attention certain psychiatric drugs may affect the performance of allergic reaction 11. Obesity patients: -may need to adjust drug dose-alert increased risk of limited respiratory function 12. The cancer patients: -evaluate the current treatment (such as chemotherapy) on the impact of immune response-alert increased risk of infection general precautions: 1. Drug interaction: -carefully review of existing medication list-assess the potential interaction of bee sting treatment drugs and chronic disease medication 2. Close monitoring: -more frequent check vital signs and laboratory indicators-low threshold considering hospitalization observation 3. Personalized treatment plan: -according to the specific chronic disease treatment plan-may require multidisciplinary teamwork 4. Patient education: -detailed explanation bee sting may impact on its chronic diseases-provide clear follow-up and emergency medical guidance 5. Long-term impact assessment: -arrange follow-up follow-up, Assess the long-term impact of bee sting on the management of chronic diseases 6. Prevention strategies: -Develop a personalized prevention plan, Reduce future bee sting risk When dealing with bee sting patients with chronic disease, The key is to fully consider the direct effects of bee stings and the complexity of chronic diseases, Develop personalized treatment and monitoring parties. | Immunocompromised individuals do require particularly careful management after bee stings as they may be at higher risk of complications, especially infections and abnormal immune responses. The following are the key points for handling such patients: 1. Preliminary assessment: a) Comprehensive assessment: -Detailed examination of the site and number of stings-assessment of systemic symptoms, Attention b) even mild symptoms medical history collection: -Understand the specific causes of immunosuppression (such as HIV, chemotherapy, organ transplantation, etc.) -ask about current immunosuppressive drugs 2. Local wound management: -thoroughly but gently clean the wound, Use of a mild antimicrobial solution-consider using topical antibiotic ointment to prevent infection-close observation of wound healing, Guard for any signs of infection 3. Infection prophylaxis: -Low-threshold use of prophylactic antibiotics, Especially for multiple stings-with the selection of broad-spectrum antibiotics, Consider covering skin common pathogens-regular assessment for adjustment or extended antibiotic courses 4. Anaphylaxis management: -vigilance of atypical or delayed allergic reactions-readiness to use antihistamines and epinephrine, Dose may be adjusted-Consider prophylactic use of oral antihistamines, Even if the initial response is slight 5. Immunomodulation: -consult an immune specialist, To ess whether the need to adjust current immunosuppressive therapy – for some patients, Short-term increase in immunosuppressants to prevent excessive immune response-vigilance against immune reconstitution syndrome 6. Monitoring and observation: -prolonged observation, It is recommended for at least 24 hours-close monitoring of vital signs and laboratory indicators-special attention to signs of infection or abnormal immune reactions such as fever, rash, and lymphadenopathy 7. Laboratory tests: -Regular examination of complete blood cell count, CRP, procalcitonin and other inflammatory indicators-monitoring liver and kidney function-consider blood culture, Especially when fever 8. vigilance of complications: -highly alert for local complications such as abscess and cellulitis-pay attention to the possibility of systemic infection, Such as sepsis-vigilance for rare complications, 9. Admission consideration: -Low threshold considering hospitalization observation, Especially for severe casesImmunosuppressed patients-may require isolation measures, Especially in neutropenic patients 10. Nutritional support: -Ensure adequate nutrient intake, Supporting immune function and wound healing-considering supplementation of vitamins and minerals, Especially vitamin C and zinc 11. Psychological support: -Provide psychological counseling, Because these patients may have additional anxiety about complications 12. Patient education: -Detailed guide patients and families to observe signs of infection and anaphylaxis-provide a clear follow-up plan and indications for emergency medical treatment 13. Long-term follow-up: -Arrange regular review, Assessment of long-term complications-considered for immune function assessment, 14. Prevention strategies: -Develop a detailed prevention plan, Reduce the risk of future bee stings-Consider providing a medical warning card or bracelet, 15. Multidisciplinary collaboration: -Joint immunology, infection, dermatology and other specialties to develop treatment plans-consider consulting a toxicology expert, To assess the specific effects of bee venom on immunosuppressed patients 16. Special circumstances: -For patients with HIV, To assess the impact of antiretroviral therapy and possible adjustments needed-for organ transplant patients, Close monitoring of the transplanted organ function, Alert to rejection when handling bee stings in immunocompromised patients, The key is to strike a balance between controlling the risk of infection and managing a potentially abnormal immune response. Personalized treatment options and close monitoring are crucial. |
|  | Medication Treatment | Medication Treatment | Medication Treatment | Medication Treatment | Medication Treatment |
| Question | What is the indication that uses antibiotic after bee sting? | What are the commonly used analgesic drugs after bee sting? | What is the basis of the choice of antihistamine drugs after bee sting? | What is the principle of glucocorticoid use after bee sting? How to choose the dose? | How to undertake fill fluid treatment after bee sting? What are the precautions? |
| Answer Engine 3（Claude Pro） | Antibiotics are used after bee stings and are unconventional, but are necessary in some specific cases. Here are the main indications for the use of antibiotics: 1. Clear signs of infection: -sting site red, swelling, heat, pain aggravation-around the wound with pus secretion-cellulitis symptoms 2. High risk wound: deep sting or multiple sting-wound is located in easy infection (such as hands, feet, joint) -wound is contaminated (such as soil, water, etc.) 3. The immunocompromised patients: -patients with HIV / AIDS-cancer patients are undergoing chemotherapy-organ transplant recipients-long-term use of corticosteroids 4. Sings in special parts: -stings on the face or around the eyes-stings in the oral or throat parts 5. Systemic symptoms: -fever (body temperature> 38°C) -general discomfort, chills and other systemic infection symptoms 6. Patients with underlying diseases: -diabetic patients, Especially for patients with poor blood glucose control-patients with peripheral vascular disease-patients with chronic skin disease (such as eczema) 7. Delayed medical treatment: -more than 24-48 hours after stings, And the wound condition is not good 8. Foreign body remains: -bee thorns cannot be completely removed, Increase the risk of infection 9 secondary trauma: -sting site skin damage caused by scratching 10. Special occupation or environmental factors: -farm workers, gardeners and other high risk occupation-sting in the pollution environment 11. Age factors: -the elderly (> 65) -infants (<2 years) 12. Previous bee sting concurrent infection history: -past bee sting after patients with severe infection 13. Doctor clinical judgment: -based on the overall situation, 1. Choice: -Antibiotics that cover common pathogens in the skin are usually preferred, Such as cephalosporins or penillins – for suspected MRSA infection, Consider clindamycin or TMP-SMX 2. Mode of administration: -Oral antibiotics may be used for mild infection-when severe infection or systemic symptoms are obvious, May require intravenous administration 3. Course: -usually 5-7 days, 4. Monitoring: -Closely observe the treatment response and possible adverse effects5. Resistance considerations: -Local resistance profiles should be considered when choosing antibiotics 6. Rational Use: -Avoid unnecessary use of antibiotics to prevent the emergence of resistant strains. It needs to be emphasized that most simple bee stings do not require antibiotic treatment. The decision to use antibiotics should be based on an individualized risk assessment and clinical performance. At the same time, good wound care and close observation are usually the key to preventing infection. | Pain management after bee sting is an important part of patient comfort and overall treatment. The following are commonly used analgesic drugs and their use: 1. Nonsteroidal anti-inflammatory drugs (NSAIDs): a) ibuprofen (Ibuprofen): -Dose: usually 400-600mg in adults, Every 6-8 hours-Advantages: both analgesic and anti-inflammatory effects-Note: may increase the risk of bleeding, Gastrointestinal adverse effects b) naproxen (Naproxen): -dose: usually 250-500mg in adults, Once every 12 hours-Advantages: Long duration of action-Note: the same risk of gastrointestinal side effects 2. Acetaminophen (Acetaminophen / Paracetamol): -Dose: usually 500-1000mg in adults, Every 4-6 hours, Not more than 4000mg per day-Advantages: fewer gastrointestinal side effects, Suitable for most population-Note: high dose may have hepatotoxicity, Alcoholics should be cautious 3. Local medication: a) Lidocaine gel / patch: -Direct application to sting sites-Advantages: rapid onset, Less side effects-Note: avoid large area use, Especially children b) containing diphenhydramine topical preparations: -both pain relieving and itching effect-note: may cause local skin reaction 4. The opioids (only for severe pain): a) codeine (Codeine): -usually combined with acetaminophen use-note: may cause constipation, drowsiness b) tramimebutin (Tramadol): -for moderate to severe pain-note: may have side effects such as nausea, dizziness, 5. Local anesthetic: a) lidocaine injection: -for local infiltration anesthesia-note: only by medical professionals use note: 1. Individual choice: -according to the patient age, underlying disease and allergic history choose appropriate drugs 2. Dose adjustment: -the elderly, patients with liver and kidney function insufficiency may need to reduce the dose 3. Drug interaction: -attention with patients are using other drug interaction 4. monitoring side effects: -focus on gastrointestinal discomfort, allergic reaction, such as 5. Use time: -usually short-term use, 6. Special population consideration: -pregnancyWomen preferred acetaminophen-children need to be adjusted according to the weight dosage 7. Combination: -can consider different mechanisms of drug combination, such as acetaminophen and NSAIDs8. non-drug measures: -combined with ice, raise the non-drug methods such as enhancement effect 9. Regular evaluation: -assess the pain degree and medication effect, timely adjustment plan 10. Patient education: -guide patients to correct use of drugs, understand the possible side effects for most bee sting, over-the-the-counter analgesic is usually enough. However, for severe pain or special circumstances, a doctor should be consulted to obtain personalized pain management parties. | Multiple factors need to be considered when selecting antihistamines after a bee sting. The following are the main basis for the selection of antihistamines: 1. Drug generation: a) The first generation of antihistamines: -For example: diphenhydramine (Diphenhydramine), chlorpheniramine (Chlorpheniramine) -Advantages: quick effect, Strong sedation-disadvantages: more central nervous system side effects, If lethargy-applicable: acute, severe symptoms; When a sedative effect is required b) second-generation antihistamines: -for example: cetirizine (Cetirizine), loratadine (Loratadine) -advantages: long-lasting effect, Almost no sedative effect-disadvantages: slow onset-applicable: long-term use; Patients who need to remain awake 2. Symptoms severity: -Mild symptoms: optional second-generation antihistamines-moderate to severe symptoms: may require first-generation antihistamines or a combination 3. Route of administration: -Oral administration: most commonly used, Suitable for most cases-intravenous injection: for patients with severe allergic reactions or unable to take orally-topical medication: such as ointment containing diphenhydramine, For local itching 4. Patients with age: -children: preferred higher safety of the second generation antihistamines-the elderly: avoid the use of significant anticholine effect drugs (such as diphenhydramine) 5. Comorbidities: -liver and kidney insufficiency: may need to adjust the dose or choose different metabolism of drugs-heart disease: note some antihistamines may affect the rhythm 6. Drug interactions: -consider the potential interactions with other drugs patients are using 7. Duration of action: -Short-term: suitable for rapid withdrawal after symptom control-Long-term: suitable for continuous control of symptoms 8. Side effects considerations: -Risk of drowsiness: Patients driving or operating machinery should choose non-sedation-dry mouth, constipation: elderly patients may be more sensitive, 9. Previous medication experience: -Consider the efficacy and tolerance of past antihistamines 10. Pregnancy and lactation: -Pregnant and lactating women should choose drugs with better safety data 11. Drug accessibility and cost: -Consider the patients economic situation and the availability of drugs 12. Type choice: -tablets, Liquid preparations, rapid dissolution tablets, etc., Selection based on patient preference and swallowing ability, 13. Expected treatment duration: -short-term use may tend to choose fast drugs-long-term use may be more suitable for less second-generation drugs 14. Combined drug considerations: -sometimes may need combined use of H1 and H2 receptor antagonists to enhance effect 15. Doctors clinical experience: -based on clinical experience and local medication guidelines to choose specific selection example: -acute severe reaction: optional diphenhydramine intravenous injection-mild itching, Work required: optional loratadine oral-pediatric patients: consider cetirizine syrup-need for rapid relief of symptoms: optional rapid dissolution tablets such as desloratadine needs to emphasize, The choice of antihistamines should be personalized, It needs to be adjusted according to the patients specific circumstances and symptoms. For severe allergic reactions, antihistamines are usually only part of the treatment, and other drugs such as adrenaline may also be required. | The use of corticosteroids after bee stings is a treatment option to carefully consider. Here is the use of principles and dose selection details: use principle: 1. indications: -moderate to severe systemic allergic reaction-large local reaction (> 10cm) -persistent or progressive symptoms-poor response to conventional treatment 2. Time: -usually in the initial treatment (such as adrenaline, antihistamines) after use-for serious reactions, Can be used as part of the early treatment 3. Treatment goals: -reduce inflammatory response-prevent delayed allergic reaction-alleviate persistent symptoms 4. Use life: -short-term use (usually 3-5 days) -long-term use to weigh the advantages and disadvantages 5. Pathway of administration: -oral: suitable for most cases-intravenous: for severe reactions or cannot oral patients-local medication: for localized reaction 6: monitoring-closely observe symptom improvement-attention to potential side effects 7. Individualized considerations: -Dose selection adjusted for patient age, weight, underlying disease: 1. Oral prednisone (or other glucocorticoids of equivalent dose): a) Adult: - -to-moderate response: 20-40mg / day, In 1-2 doses-severe response: 40-60mg / day, In 1-2 doses, b) Children: -1-2mg / kg / day, The maximum dose is usually not more than 60mg / day 2. Intravenous methylprednisolone (for severe reactions): a) Adults: -Initial dose: 125-250mg-60-125mg b may be required every 6-8 hours) Children: -1-2mg / kg, Every 6-8 hours 3. Local medication (such as hydrocortisone cream): -the concentration is usually 0.5-2.5%, 1. Symptoms severity: -The more severe the symptoms, Initial dose may be higher 2. Patient weight: -obese patients may need to adjust the dose based on the ideal weight 3. Age factors: -the elderly may need to reduce the dose to reduce the risk of side effects 4. Basic disease: -diabetes patients using close monitoring of blood sugar-renal insufficiency may need to adjust the dose 5. Drug interactions: -consider potential interactions with other drugs 6. Treatment response: -rootDynamic dose adjustment according to symptom improvement 7. Progressive reduction: -Use over 5-7 days, Should consider gradually reduction in order to avoid adrenal suppression use precautions: 1. The side effects monitoring: -pay attention to elevated blood sugar, gastrointestinal discomfort, mood change, such as 2. Risk of infection: -alert potential infection cover or aggravating 3. osteoporosis: -long-term use of calcium and vitamin D supplement 4. Childrens growth: -long-term use may affect childrens growth, 5. Pregnancy and lactation: -Balance the pros and cons, Use under the guidance of a specialist when necessary 6. Previous medical history considerations: -caution in patients with gastric ulcer, glaucoma, osteoporosis, etc. 7. immunosuppression: - The use of corticosteroids after bee stings should be based on an individualized assessment, weigh the advantages and disadvantages. Dose selection requires consideration of multiple factors and adjustment for response during treatment. For complex or serious cases, it is recommended to make it under the guidance of a specialist doctor. | Rerehydration after bee stings is an important treatment step, especially for patients with severe or multiple stings. Here is the details of rehydration treatment and note: rehydration of treatment principles: 1. Instructions: multiple sting (usually> 50) -patients with systemic symptoms-dehydration or circulatory instability-rhabdomyolysis patients 2. rehydration target: -maintain sufficient circulation capacity-promote toxin discharge-prevent kidney injury-correct electrolyte disorders 3. The rehydration mode: -oral rehydration: suitable for mild dehydration and can tolerate oral patients-intravenous rehydration: used in patients with moderate to severe dehydration or unable to tolerate oral rehydration scheme: 1. Initial assessment: -assess dehydration-Check vital signs, In particular, blood pressure and heart rate-Assessment of urine volume 2. Fluid selection: -isotonic crystalloid: such as 0.9% sodium chloride solution or ringers lactic acid solution-for patients with electrolyte disorders, The quid composition may be adjusted 3. Speed and volume of rehydration: a) mild dehydration: -20-30 mL / kg, Within 2-3 hours, b) moderate dehydration: -initial administration of 20-40 mL/kg rapid rehydration-subsequent adjustment according to response c) severe dehydration or shock: -initial administration of 40-60 mL/kg rapid rehydration – close monitoring of the reaction, 4 may require repeated doses. Maintenance hydration: -adjusted to continued loss and underlying demand-usually 1.5-2 mL / kg / hour 5. Special circumstances: -Rhabdomyolysis: more active hydration may be required, Target urine volume 1-2 mL/kg / hour Note: 1. Monitoring: -Regular check of vital signs-monitor urine volume (target> 0.5 mL / kg / hour) -observe for signs of edema or volume overload 2. Electrolyte balance: -Regular check of electrolyte level, Especially potassium, sodium, calcium, -electrolyte correction when necessary 3. Acid-base balance: -Monitoring blood gas analysis, Correct acid-base imbalance 4. Renal function: -Monitoring creatinine and urea nitrogen levels-alert for the occurrence of acute kidney injury 5. Cardiac function considerations: -For patients with cardiac insufficiency, The speed and total amount of rehydration should be carefully controlled-Consider the use of diuretics 6. Age factors: -Elderly patients may be more sensitive to volume loading, needA more careful rehydration-rehydration in children should be calculated accurately based on body weight 7. Complication prevention: -Prevention of deep vein thrombosis: encourage early mobilization or use of prophylaxis-Stress ulcer prevention: consider the use of proton pump inhibitors 8. Nutritional support: -While rehydration, Do not ignore nutritional intake 9. Special rehydration requirements: -For patients with severe allergic reactions, May need colloid-rhabdomyolysis patients can consider adding sodium bicarbonate alkaline urine 10. The dynamic assessment: -according to the patient response and clinical indicators constantly adjust rehydration scheme 11. Excessive rehydration risk: -alert to pulmonary edema, electrolyte disorders such as excessive rehydration complications 12. After discharge guidance: -education patients continue the importance of oral rehydration-provide warning information of dehydration symptoms of rehydration treatment should be individualized, It needs to be adjusted according to the specific condition of the patient. For complex cases, such as patients with multiorgan insufficiency or underlying disease, more specialized fluid management strategies may be needed. |
|  | Wound Treatment | Wound Treatment | Wound Treatment | Wound Treatment | Wound Treatment |
| Question | What are the characteristics of skin damage after bee sting and its clinical significance? | How to calculate the number of bee sting sting needles? | Do you need to remove the poison sting after a bee sting? | Is there a need for routine tetanus prevention after bee stings? | How to deal with the cellulitis caused by bee sting? |
| Answer Engine 3（Claude Pro） | Skin damage after a bee sting is not only a manifestation of a local response, but may also reflect the severity of the systemic response. Understanding these characteristics and their clinical implications is essential for assessing the condition and developing treatment options. Here are detailed analysis: 1. Instant response: characteristics: -puncture point around erythema and slight swelling-local pain and burning clinical significance: -normal immediate response-suggests that bee venom has been injected into the skin 2. Local swelling: characteristics: -sting area around obvious swelling-range may expand to 10cm or more clinical significance: -moderate local reaction-if swelling rapidly expanding or more than 10cm, May indicate a more severe response 3. Urticaria-like reaction: characteristics: -raised, well-defined erythema around the sting site-possibly with pruritus clinical significance: -prompt local histamine release-if urticaria is rapidly spread to other sites, 4. A precursor of systemic allergic reaction. Angioedema: Features: -diffuse swelling of deep skin and subcutaneous tissue-common in relaxed tissues such as eyelids and lips. Clinical significance: -may be a manifestation of severe allergic reaction-if it affects the mouth or throat, May endanger the respiratory tract 5. Skin pale or cyanosis: features: -sting area around the skin color white or blue purple clinical significance: -may represent local blood circulation damage-serious may cause tissue necrosis 6. blister or blister formation: features: -sting site full of liquid bubbles or big bubble clinical significance: -more serious local reaction-increase the risk of secondary infection 7. Bleor ecchymosis: features: -sting site small spot bleeding or larger area ecchymosis clinical significance: -may suggest abnormal coagulation-multiple sting more need to be vigilant 8. Necrosis: features: -sting site skin blackening, hardening-may have obvious inflammation around clinical significance: -prompt serious local tissue damage-may need surgical intervention 9. abscess formation: features: -sting site form pus mass-usually accompanied by pain, fever and other systemic symptoms clinical significance: -said secondary bacterial infection-may need antibiotic treatment or drainage 10. Extensive rash: features: -away from the sting site rash-may appearFor maculopapular rash, erythema and other clinical significance: -prompt systemic allergic reaction-alert to anaphylactic shock may 11. Late skin reaction: characteristics: -sting several days after rash or other skin changes clinical significance: -may be serum disease reaction-prompt immune system 12. Late bee residue: features: -visible small black dot bee thorn embedded in the skin clinical significance: -need to remove to reduce toxin continue to release-residue may increase local reaction and infection risk 13. Lymphangitis: Features: -Clinical significance of the red line extending from the sting site to the proximal lymph node: -suggesting local inflammatory reaction spreading along the lymphatic vessels-Principles of possible assessment and treatment of systemic infection: 1. Comprehensive examination: not only observe the stings site, 2. Dynamic observation: regularly assess skin changes, Especially within 24-48 hours after stings 3. Related examination: serious reaction may need blood routine, coagulation function and other auxiliary examination 4. Personalized treatment: according to the skin damage characteristics choose appropriate local and systemic treatment 5 specialist consultation: complex or serious cases may require dermatology, allergic immunology consultation skin damage characteristics and evolution is crucial to assess the severity of bee sting and prognosis. Medical staff should be familiar with these characteristics and develop appropriate treatment strategies based on the overall clinical performance of the patient. | The calculation of the number of bee stings is an important link in assessing the severity of bee stings. Accurate calculation of stings can help healthcare professionals to assess potential risks and develop appropriate treatment options. The following are the methods of calculate the number of stings and related considerations: calculation method: 1. Direct counting method: -suitable for the number of stings is small (usually <50) -carefully check the patients body skin, 2. Area estimation method: -suitable for large or multiple stings-divide the body into several major areas (e. g., head, trunk, limbs) -select a representative small area (e. g., 10x10cm) for accurate counting-based on the number of stings in this small area, Estimate the number of stings in the whole area-Add the estimates of all areas to get the total 3. Bee count: -Suitable for bee stings (because bees leave the needle) -calculate the number of stings remaining on the skin 4. Image Analysis: -For large stings, Can take photos of the affected area-use image analysis software to count or estimate 2. Distinguish between the types of stings: -Bees usually leave behind the prick needles, 3. Consider the time factor: -Some of the stings may not be obvious at the early stage, Become more visible over time 4. Record method: -Use body charts to mark the location and number of stings-this helps for follow-up observation and assessment 5. Dynamic assessment: -After the initial assessment, Regular recount, Because new response points may occur 6. Consider individual differences: -the body surface area is different in children and adults, 7. Assessment of severity: - More than 50 stings are severe stings-but the patients age, underlying disease and other factors should also be considered. 8. Other clinical manifestations: -The number of stings should be combined with the patients symptoms and signs. 9. Professional assistance: -For large or difficult counting cases, 10. Technical assistance: -If conditions permit11. Special conditions: -For stings in special parts such as the mouth and eyes, 12. Patient self-report: -Combined with patient estimates of the number of stings, 13. Safety considerations: Ensuring patient and healthcare staff safety, Avoid possible live bees 14. Record the degree of response: -In addition to the number, The degree of response (e. g. redness range) of each sting should also be recorded. An accurate calculation of the number of stings is essential to assessing the severity of illness and developing treatment plans. However, this should be part of the overall evaluation, which needs to combine the clinical manifestations, laboratory results and other comprehensive tests. | The need for remove removal after bee sting is an important issue. In general, spurs should be removed as soon as possible. But this process needs to be performed correctly to minimize additional toxin injection. Lets discuss this question in detail: whether the sting need to be removed: 1. Necessity: -for bee stings, Usually need to remove the sting. -wasps, wasps, etc., usually do not leave the sting. 2. Emergency: -should be removed as soon as possible, Ideally, within seconds to minutes after a sting-the poison sac may continuously pump into the toxin, Rapid removal can reduce the amount of toxin injected extraction method: 1. Push scraping (recommended): -Use credit card edge or nail quick scraping-Advantages: reduce the risk of squeezing poison sac, Reduce the likelihood of additional toxin injection 2. Pating (not recommended): -pinch the poison directly with your finger or tweezers to pull out-disadvantages: may squeeze the sac, 3. Lead to more toxin injection 3. Paste method: -use tape or medical tape to stick the sting after quickly tear off-suitable for multiple small area stings Note: 1. Identification: -Confirm whether there is really a sting-bee sting usually appear as small black spots on the skin 2. Clean: -After removing the sting, Wash the stings with soap and water 3. Time consideration: -Even if it is found late, 4. Tool selection: -Avoid using sharp tools, In case of additional damage 5. Multiple stings: -For multiple stings, 6. Allergic reaction: -removing removal does not prevent allergic reaction, 7. Special sites: -Stings should be handled by professional medical staff 8. Child patients: -may require adult assistance, Keep calm to avoid additional damage 9. Subsequent treatment: -After removing the sting, 10. Record: -Record the number and location of the sting, Help to assess the severity of infection 11. Prevention of infection: -After the removal of the sting, Observe the wound for signs of infection 12. Education: -Explain to the patient the importance of removing the sting, And how to identify and manage possible future stings 13. Professional help: -such as14. Preof evidence: -In some cases (for example, forensic investigations), removal may be necessary to remove removed is an important step in the treatment of bee stings, but it is only part of the overall treatment. Even if the sting is successfully removed, close attention should be paid to the possible local or systemic reversal. | The need for routine prevention of tetanus after bee stings needs to be carefully considered: 1. In general, simple bee stings do not require routine tetanus prevention. Bee stings mainly cause local pain, swelling and other allergic reactions, while tetanus is caused by Clostridium tetanus.2. However, if bee stings cause significant skin damage or deep wounds, especially in potentially contaminated environments (such as dirt, dust, etc.), then tetanus prevention may need to be considered.3. The necessity of tetanus prevention also depends on the personal history of tetanus vaccination. If tetanus has been vaccinated in the past 10 years, no additional prophylaxis is usually required.4. If you are not sure about your vaccination, or if you have serious wounds, it is best to consult your doctor. The doctor can assess the need for tetanus prevention on a case-by-case basis.5. For bee stings, it is more important to treat the wound in time, such as cleaning, ice compress, and observe for severe allergic reactions. In general, bee stings alone usually do not require routine prevention of tetanus, but it is recommended to consult a medical professional if circumstances. | Cellulitis caused by bee stings is a serious complication that requires prompt and correct management. The following are the main steps to handle cellulitis: 1. Medical diagnosis First, seek medical treatment immediately. Cellulitis requires diagnosis and treatment by a professional physician.2. Antibiotic therapy-Oral antibiotics are usually the preferred treatment method.-Severe cases may require intravenous antibiotics.-The doctor will choose appropriate antibiotics based on the severity of the infection and the patient.3. Local treatment-Keep the affected area clean and dry.-Warm application may be recommended to promote blood circulation and relieve symptoms.-Drainage of pus may be required accordingly.4. Rest and raise the affected area-Rest helps you recover.-Elevating the infected site reduces the swelling.5. Pain management-Doctors may recommend the use of nonsteroidal anti-inflammatory drugs (NSAIDs) to relieve pain and reduce inflammation.6. Monitor-Watch monitor changes in symptoms.-If the symptoms worsen or become new, seek medical attention immediately.7. Prevention measures-Avoid squeezing or piercing the infected area.-Complete full antibiotic treatment as prescribed, even if symptoms improve.8. Follow-up- -According to the doctors advice to ensure effective control of infection. It should be noted that cellulitis may cause serious complications if not treated promptly. Therefore, once cellulitis is suspected, you should seek medical attention as soon as possible and do not try to handle it by yourself. |
|  | Long-term Follow-up | Long-term Follow-up | Long-term Follow-up | Long-term Follow-up | Long-term Follow-up |
| Question | Which patients need long-term follow-up after discharge after bee stings? | What does the content of long-term follow-up after bee sting patient discharge have? | What does the health education content of the rehabilitation period after bee stings include? | How to follow-up and manage patients with secondary renal failure after bee sting? | How to undertake psychological guidance when bee sting injury patient leaves the hospital? |
| Answer Engine 3（Claude Pro） | After bee stings, most patients can be safely discharged after appropriate treatment.yet, Certain patients may require long-term follow-up, It mainly includes the following categories: 1. Patients with severe allergic reactions: -Patients who have had systemic anaphylactic shock) -Patients allergic to bee venom but not undergoing desensitization treatment 2. Patients with organ function impairment: -Patients with impaired renal function, Need to monitor the recovery of renal function-in patients with abnormal liver function, Need for regular examination of patients with liver function-myocardial injury, Need to track the heart function 3. Multiple bee sting or a large number of bee sting patients: -may be delayed toxicity (delayed toxicity) risk-need to monitor whether late complications 4. immunocompromised patients: -such as HIV infection, patients undergoing chemotherapy-need to pay close attention to wound healing and potential infection 5. Elderly patients or patients with underlying disease: -may be more sensitive to bee venom-need to assess the impact of bee sting on the original disease 6. Patients with atypical or persistent symptoms: -such as persistent pain, itching, swelling-may require further treatment or treatment These patients may require regular follow-up visits, laboratory tests or specialist consultation after discharge, To ensure adequate rehabilitation and to prevent potential complications. The specific follow-up plan should be made by the doctor according to the individual patient situation. | Bee sting patients after discharge long-term follow-up content usually includes the following aspects: 1. Symptoms assessment: -pain, swelling, itching and other local symptoms duration and severity-systemic symptoms such as fever, fatigue, joint pain monitoring-whether new or atypical symptoms 2 physical examination: -sting site healing-lymph node enlargement-skin condition (such as whether secondary infection, scar formation, etc.) 3. Laboratory tests: -blood routine: monitoring white blood cell count, eosinophil proportion-liver function: ALT, AST, bilirubin index-renal function: creatinine, urea nitrogen, electrolytes, myocardial enzyme spectrum: such as CK-MB, troponin (in patients with myocardial injury) -urine routine: check for hematuria, proteinuria, etc. 4. Image examination (as needed): -ultrasound examination: evaluation of soft tissue injury, organ function-X-ray or CT: for suspected deep tissue injury 5. Allergy assessment: -skin test or serological examination: assess the allergic state of bee venom-discuss whether the need for desensitization treatment 6. Psychological assessment: -assess whether patients with post-traumatic stress disorder (PTSD) psychological problems-provide psychological counseling or treatment when necessary 7. Complications screening: -check whether delayed complications, Such as serum disease-like reaction-to evaluate whether the original underlying disease is affected 8. Prevention education: -guide patients how to avoid bee sting again-education patients to identify the early symptoms of allergic reaction-guide patients to correctly use epinephrine autosyringe (if prescribed) 9. Treatment plan adjustment: -evaluate the current treatment effect, Adjust medication when necessary- -to discuss whether the need for long-term emergency medication (such as antihistamines, adrenaline, etc.) is required. 10. Quality of life assessment: -To Assess the impact of bee stings on daily life and work- -the frequency and duration of follow-up with rehabilitation guidance or occupational advice when necessary should be based on the patients specific situation. For example, more frequent follow-up may be required for patients with severe allergic reactions, and only for short periods. The doctor will adjust the follow-up plan according to the results of each follow-up visit. | Health education in the recovery period after bee stings is the key to ensure full recovery and prevent recurrence. These educational contents should include the following aspects: 1. Wound care: -Keep the sting site clean and dry-correctly use the doctor recommended local drugs (such as anti-inflammatory cream, etc.) -avoid excessive scratching, In case of infection and scarring-to observe the wound healing process, 2. Symptom management: -How to correctly use prescription drugs such as painkillers and antihistamines-use physical methods such as ice compress to relieve local symptoms- -identify warning symptoms that require medical treatment, Such as persistent fever, severe pain, etc. 3. Allergic reaction: -understand the symptoms and signs of bee venom allergy-learn to use epinephrine automatic syringe (if the doctor prescribed) -know when and how to seek emergency medical help 4. Prevent again sting: -identify common bees and their habitat-learn how to safely remove honeycomb in the family environment-protective measures in outdoor activities, Like wearing appropriate clothing-avoid using strong scented cosmetics, 5. Lifestyle adjustment: -Diet advice, May include an increased intake of anti-inflammatory foods-moderate exercise, Promote recovery but avoid overstimulation of sting sites-adequate rest, Support the natural physical recovery process 6. Mental health: -ways to deal with possible anxiety or fear-the importance of seeking psychological support if necessary-relaxation techniques, Such as deep breathing or meditation, To reduce stress 7. Long-term health management: -the importance of regular allergy testing-consider the possibility of desensitization treatment and its advantages and disadvantages-keep the immune system healthy habits 8. First aid knowledge: -basic bee sting first aid steps-how to safely remove residual bee sting-family first aid kit should contain items list 9. Environmental safety: -how to conduct home and workplace safety assessment-reduce the factors that attract bees, Such as open food containers or sweet drinks 10. Community resources: -Provide contact information for local allergists-introduce information about support groups or educational courses-recommend reliable sources of health information, In order to enter oneStep learning 11. Travel notes: -before the travel destination local bees-carry the necessary drugs and medical documents-understand the destination of emergency medical services through comprehensive health education, can help patients better manage the rehabilitation process, improve the quality of life, and significantly reduce the risk of another bee stings. Medical personnel should individualized these educational content according to the specific situation, and encourage patients to actively participate in self-management. | Patients with secondary renal failure after bee stings require comprehensive and meticulous follow-up management to monitor recovery of renal function, prevent complications, and provide appropriate treatment. The following are the main contents of the follow-up management of such patients: 1. Follow-up frequency: -initial (1-3 months after discharge): weekly or bi-weekly-intermediate (3-6 months): once every 2-4 weeks-long-term (after 6 months): according on the patients condition, Every 1-3 months 2. Renal function assessment: -serum creatinine, urea nitrogen, electrolyte level detection-estimation of glomerular filtration rate (eGFR) -24 hours urinary protein quantification-urine routine examination 3. Hematology examination: -complete blood cell count-coagulation function-serum albumin level 4. The other organ function assessment: -liver function examination-electrocardiogram and cardiac ultrasound (assess cardiac function) -lung function examination (if necessary) 5. Nutritional status assessment: -body weight monitoring-serum albumin, prealbumin levels-trace element check (such as calcium, phosphorus, iron, etc.) 6. Complications screening: -anemia assessment-bone mineral metabolism disorder screening-cardiovascular disease risk assessment 7. Drug treatment adjustment: -evaluate the efficacy of existing drugs and side effects-adjust drug dose according to the renal function-consider adding renal protection drugs (such as ACEI / ARB) 8. Blood pressure management: -regular monitoring blood pressure-adjust antihypertensive drug regimen 9. Dialysis related management (if patients need dialysis): -assess dialysis adequacy-monitoring dialysis access function-adjust dialysis prescription 10. The lifestyle guidance: -diet advice (low salt, control protein and potassium intake) -moderate exercise program-quit limit 11. Mental health assessment: -screening for depression, anxiety and other psychological problems-necessary counseling or referral 12. Patient education: -kidney disease progression prevention measures-identify the symptoms need emergency-medication compliance education 13. Prevention of re-sting: -emphasize the importance of avoiding recurrence-provide specific preventive measures recommendations 14. Multidisciplinary collaboration: -Regular consultation with nephrology, cardiology, endocrinology and other specialties-referral to related specialties for further evaluation and treatment if necessary15. Long-term prognosis assessment: -regular assessment of renal function recovery-assess whether the need for renal replacement therapy (such as long-term dialysis or kidney transplantation) 16. Social support assessment: -assess the patients social support system-provide information on relevant social resources at each follow-up, the doctor should adjust the management plan according to the specific situation of the patient. The focus of follow-up may be adjusted over time and changes in patient status. It is important to adopt individualized management programs and encourage patients to actively participate in self-management. | Psychological guidance of bee sting patients at discharge is an important part of the recovery process. This guidance is designed to help patients deal with possible psychological problems and promote physical and mental health recovery. The following are the main contents that should be covered when giving psychological guidance: 1. Normalization of psychological responses: -Explain that the psychological reactions that patients may experience (e. g., anxiety, fear) are normal- -emphasizing that these reactions are usually temporary, Over time will gradually reduce 2. Education patients to identify common psychological symptoms: anxiety and panic-fear of bee sting again-sleep-irritability or mood swings-avoid outdoor activities or bee related scene 3. Provide coping strategies: relaxation skills: deep breathing practice, progressive muscle relaxation, meditation-cognitive reconstruction: help patients to identify and challenge unreasonable ideas-progressive exposure: gradually contact cause anxiety situation (such as outdoor activities) 4. Encourage social support: -Highlight the importance of sharing feelings with family and friends-recommend joining support groups or finding people with similar experiences 5. Provide information and education: -Accurate information about bee stings, To reduce unnecessary concerns-practical measures to prevent re-sting, Increased security 6. Highlight the importance of self-care: -keep regular schedule and healthy lifestyle-moderate exercise benefits for physical and mental health-keep a balanced diet and adequate sleep 7. Set realistic recovery goals: -help patients develop short-term and long-term recovery goals-emphasize the importance of gradually return to normal life 8. Discuss possible post-traumatic stress disorder (PTSD): -explain the symptoms and characteristics of PTSD-emphasize if symptoms continue or worse, The importance of seeking professional help 9. Provide mental health resources: -recommend reliable self-help books or online resources-provide mental health professionals contact information 10. Family member guidance: -educator people how to provide emotional support for patients-guide family to identify warning signals need professional intervention 11. Work and life to adapt: -discuss possible challenges to return to work-provide advice to reintegrate into social life 12. Encourage productExtreme mentality: -emphasis on rehabilitation is a process, need patience-encourage patients to pay attention to the progress of recovery, rather than limited to difficult 13. Make emergency plan: -help patients to deal with the emergency or psychological crisis plan-to ensure that patients know how to timely help when need 14. Follow-up arrangement: -explain the importance of mental health follow-up-arrange regular mental health examination in these psychological guidance, medical personnel should adopt empathy and supportive attitude, according to the specific situation and needs of each patient for personalized guidance. At the same time, it should be emphasized that mental health is equally important as physical health, and patients are encouraged to seek professional mental health clothing when needed. |
|  | Public Health and Prevention | Public Health and Prevention | Public Health and Prevention | Public Health and Prevention | Public Health and Prevention |
| Question | How to carry out public health education in areas with high incidence of wasp sting? | What are the high-incidence seasons of bee stings and the high-risk groups? | What content should the emergency treatment plan for mass bee stings include? | What are the environmental assessment and improvement measures after wasp stings? | Is it recommended for the removal and management of beehives in areas with a high incidence of bee stings? How to implement it? |
| Answer Engine 3（Claude Pro） | Public health education in areas with high incidence of wasp sting is a key measure to prevent bee sting injury. This education should be comprehensive, continuous, and tailored to the needs of different populations. Here is how to conduct the comprehensive scheme of public health education: 1. The target population identification: -the general public-outdoor workers (such as farmers, forestry workers, construction workers) -students and teachers-medical and health personnel-tourism practitioners and tourists 2. Education content: a. wasp ecology knowledge: -wasp species and characteristics-wasp living habits and activity rules-wasp nesting site identification b. Preventive measures: -avoid provoking wasp behavior-correct outdoor dress (long sleeves, trousers, hats, et c.) -how to safely remove or report wasps-use mosquito repellent and other protective equipment c. emergency response: -bee sting correct first aid steps-identify allergic symptoms-when to seek medical help d. Environmental management: -reduce wasp factors (such as open food containers) -family and community environment safety check 3. Education method: a. School education: -wasp safety knowledge into courses-organize lectures and simulation exercises b. Community propaganda: -distributing brochures and posters-community lectures and workshops-using community bulletin boards and electronic display c. media propaganda: -TV and radio public ads-newspaper and magazine features-social media campaign d. outdoor warning: -warning in high-risk areas-provide emergency contact information e. Vocational training: -provide special safety training for outdoor workers-development industry specific safety guide 4. Education resources development: -make easy to understand illustrated promotional materials-develop interactive learning tools and mobile applications-create online learning platform and repository 5. Cooperation and coordination: -cooperation with local governments, health departments, education institutions-invite experts to participate in the education content development and audit-cooperate with environmental protection organizations, 6. Special group protection: -forProvide personalized guidance for allergic patients-design special education content for the elderly and children 7. Emergency response system: -Establish community emergency response network-training volunteers for preliminary emergency treatment 8. Regular assessment and update: -Collect community feedback-analyze bee sting injury data, Assess the effectiveness of education-updating educational content according to new research findings and practical experiences 9. Cultural sensitivity: -Consider local cultural customs, 10. Long-term tracking: -Establish a long-term monitoring system, Tracking bee sting injury incidence-Regular release of public health reports, Maintaining public vigilance through this comprehensive public health education programme, Can significantly increase public awareness and ability to prevent wasp stings, Reduce the occurrence of injury events. The key to the success of education lies in sustainability, universality and pertinence, and at the same time, education policies should be constantly adjusted and improved according to the actual situation. | The occurrence of bee stings has obvious seasonal characteristics, and some people face higher risk due to factors such as occupational or living habits. Understanding this information is essential for the effective prevention and management of bee stings.1. High incidence season: The high incidence season of bee stings is mainly concentrated in the warm months, This may vary slightly depending on location and climate: a. Summer (June-August): -This is the main peak season-warm weather makes bees frequent-people are more outdoors, Increased exposure b. Late spring (April-May): -colonies to be active-increased nesting and reproductive activity c. Early autumn (September-October): -Some areas remain warm-bees reserve food for winter, 2. High-risk groups: Some groups due to occupational, living environment or personal factors, Facing a higher risk of bee sting: a. Occupational-related high-risk groups: -agricultural workers (farmers, fruit farmers) -forestry workers-construction workers, Especially outdoor construction personnel-gardeners and landscape designers-beekeepers-outdoor sports coach (such as mountain climbing, hiking guide) -sanitation workers b. outdoor enthusiasts: -climbers and hikers-camping enthusiasts-outdoor sports participants (e. g., cycling, running) -fishing enthusiasts c. people in a specific environment: -residents living in forests or rural areas-often in the park or garden activities d. Special constitution population: -Individuals allergic to bee venom-people with a previous bee sting (may have been sensitized) -immunocompromised patients e. Age-related high-risk group: -Children (curious, May inadvertently provoke bee colonies) -older people (the response may be slower, Difficult to escape quickly) f. Other high risk groups: -wearing bright clothes or use strong fragrance products-alcoholism (judgment may be impaired) -the environment is not familiar with tourists 3. Note: -high season and high-risk population characteristics may vary due to geographical location and local bee species-climate change may affect bee activity patterns, leadChanges in high-incidence seasons-certain occupations may also face high risk during atypical seasons (e. g., winter forestry operations) -a personal history of allergies is an important factor in assessing risk, Not limited to a specific season or occupation 4. The prevention strategy: -for season to strengthen public education and warning-provide special safety training and protection guidance-in high risk areas set obvious warning signs-encourage high-risk groups to carry first aid supplies (such as adrenaline automatic syringe) -regularly update and release swarm activity information about the characteristics of the season and high-risk groups, It helps to develop more targeted preventive measures and emergency response strategies. For individuals, if they are high-risk groups or have outdoor activities in the peak season, take additional precautions, such as wearing appropriate protective clothing, avoid using strong scented products, and know the basic emergency response side. | Group bee stings is a serious public health emergency that requires rapid and effective response. A comprehensive emergency treatment plan should include the following key contents: 1. Emergency response system: a. Establish command center: -clear command level and the division of responsibilities-set up the field commander role b. The communication system: -establish fast communication channels between various departments-to ensure the reliability of communication equipment and standby plan c. Emergency team formation: -medical rescue group-on-site rescue group-logistics group-security group-information collection and analysis group 2. Field disposal process: a. Risk source control: -isolation swarm activity area-when necessary to take bee measures (performed by professionals) b. Personnel evacuation: -develop rapid evacuation route-designated safe collection point c. casualty classification: -establish rapid triage system (e. g., tertiary triage method) -priority for severe patients d. First aid: -remove residual bee sting-clean wounds-use antiallergic drugs (such as oral antihistamines) -when necessary to use adrenaline automatic syringe e. Transfer arrangement: -determine the receiving hospital and the advance notice-arrange enough ambulances and other transportation 3. Medical resource allocation: a. Medical supplies preparation: -allergic drugs (including adrenaline) -intravenous infusion equipment-endotracheal intubation tools-cardiopulmonary resuscitation equipment b. Medical personnel scheduling: -emergency doctors and nurses-allergy-allergy specialist, intensive medical expert c. backup medical resources: -identify and contact the surrounding medical institutions to obtain support-establish temporary medical plan 4. Information management: a. The casualty information collection: -establish rapid registration system-record key medical information (e. g., allergies) b. public information release: -specify news spokesman-develop information release process and channel c. family notice and comfort: -establish family contact point-provide psychological support service 5. Logistics: a. supplies: medical supplies supply plan-food and drinking water supply B. Traffic security: -ensure rescue channel-arrange the necessary transportation c. Power and communication security: -prepare emergency power equipment-to ensure the stability of the communication network 6. Special circumstances processing: a. deal with bad weather: -prepare rain, sunscreen equipment-adjust the rescue strategy b. processing media attention: -media countermeasures-arrange press conference c. -start regional medical resources linkage mechanism-consider the establishment of the field hospital 7. Practice and training: a. Organize regular simulation exercises: -the whole process of emergency response drills-targeted skills training (such as rapid triage, bee sting processing) b. Emergency plan regular update: -according to the drill feedback adjustment plan-into the new medical technology and equipment 8. Evaluation and improvement: a. Summary meeting: -analyze the effect of emergency response-identify need to improve link b. long-term tracking: -long-term follow-up of severe patients-evaluate the effect of psychological intervention c. Preventive measures to strengthen: -according to the cause of the event, Develop targeted prevention strategies-Strengthen public education and early warning system This comprehensive emergency treatment plan covers all aspects from on-site rescue to follow-up. In the actual implementation, it should be adjusted and improved according to the specific local conditions (such as geographical environment, distribution of medical resources, common bee types, etc.). At the same time, the successful implementation of the plan also relies on regular training and drills to ensure that it can sound quickly and effectively in an emergency | After the wasp sting event, a comprehensive environmental assessment and improvement measures are key to prevent future incidents. This not only helps to reduce the risk of recurrence, but also improves the overall safety of the community. Here are the details of environmental assessment and improvement measures: 1. Environmental assessment: a. honeycomb location: -to the area and the surrounding a comprehensive exploration-using professional equipment (e. g., infrared camera) detection hidden honeycomb-draw honeycomb distribution map b. Ecological environment analysis: -local vegetation type and density-identify may attract wasp food source (if garden, garbage, et c.) -analysis water distribution c. Human Factor Assessment: -Investigate human activity patterns (e. g., common outdoor areas) -assess whether buildings and structures provide nesting sites for wasps-Check abandoned buildings or equipment d. Climate factor analysis: -Collect local meteorological data, Analysis of association with wasp activity-assess the impact of seasonal changes on wasp behavior e. Historical data collection: -organize past sting records-analyze the spatial and temporal distribution pattern of events 2. Improvement measures: a. Honeycomb management: -safe removal of discovered beehives (performed by professionals) -for beehives that cannot be removed, Set up warning signs and isolation measures b. Environmental transformation: -trim too dense vegetation, Reduce hidden places for wasps- -Clean up food sources that may attract wasps (e. g. timely handling of rotting fruit in orchards) - -Block gaps and holes in buildings that can become nesting points. c. Early warning system establishment: -Install wasp activity monitoring equipment (e. g. sound or motion sensors) - -Establish a community reporting system, Encourage residents to report their wasp activities in time. d. Safety area Settings: -Set up obvious warning signs in high-risk areas- -create a "safety buffer zone", Reduce contact with human bees e. Ecological balance maintenance: -Protect their natural enemies (such as some birds) -plant plants that can drive bees (such as mint, lemon grass, etc.) f. Improve waste management: -Optimize waste collection and disposalsystem, Reduce attraction to wasps-Waste that may become wasp habitat g. Architectural design improvements: -Consider bee-proof measures in new building design-renovate existing buildings, Reduce suitable for wasps nesting structure h. Community education to strengthen: -environmental safety awareness education activities-training community volunteers to identify and report potential risks i. professional prevention and control measures: -in high-risk season hire professionals for regular inspection-using environmental repellent in specific areas build barrier j. Perfect emergency facilities: -set first aid station in public places, Equipped with anti-allergy drugs-ensure that emergency communication system covering all high-risk areas 3. Long-term monitoring and evaluation: a. Establish long-term monitoring system: -regular environmental patrol-continuous collection of wasp activity data b. effect assessment: -regular analysis of sting incidence changes-assess the cost effectiveness of improvement measures c. adaptive management: -according to the monitoring results and new research adjustment management strategy-exchange experience with other areas, 4. Cross-departmental cooperation: a. Establish a cooperation mechanism: -Cooperate with environmental protection departments, Ensure the eco-friendliness of the measures-working with urban planning authorities, Including bee prevention considerations in urban design b. Scientific support: -In cooperation with research institutions, Developing wasp ecology research-Exploring new and more effective prevention and control technologies Implementing these environmental assessments and improvement measures requires systematic approaches and sustained efforts. It not only involves actual environmental modification, but also requires community engagement and multi-sectoral collaboration. Through comprehensive environmental management, the risk of wasp stings can be significantly reduced, while also improving the environmental safety intention of the whole community. | In areas of high incidence of bee sting, the removal and management of hives is indeed an important preventive measure, but this requires a careful and professional approach to implement. The following is a comprehensive analysis and suggestions for this problem: 1. Do you recommend removal and management? The answer is: conditionally. Honeycomb removal and management should be based on the following considerations: a. Security risk assessment: -honeycomb location (close to human activity area) -colony size and aggression-historical bee sting incident record b. Ecological impact assessment: -bee role in the local ecosystem-potential impact on pollination and agriculture-whether the protection of species c. Laws and regulations consider: -local regulations on wildlife protection-honeycomb treatment regulations 2. How to implement honeycomb removal and management? If honeycomb management, The following steps should be taken: a. Preparation: -Professional assessment: * hire a professional entomologist or beekeeping expert to conduct on-site evaluation * Determine bee species, Assessment of swarm size and behavior characteristics-plan: * according to the results of the detailed processing plan * consider time, methods, required equipment and personnel-safety measures: * prepare appropriate protective equipment (body protective clothing, mask, etc.) * ensure the first aid equipment and anti-allergy drugs present-notification and coordination: * inform local residents and related departments * set necessary temporary isolation b. Honeycomb treatment: -Physical removal: * Suitable for beehives that can be safely approached * Collect bees using professional tools (e. g., vacuum attractor) * Remove the whole honeycomb carefully-Chemical method: * Use only if necessary, Give priority to environmentally friendly bee repellent * Strict control of use, Avoid unnecessary contamination of the environment-Isolation and monitoring: * For beehives that are difficult to remove directly, Quarantine measures may be taken * Set up warning signs, Regular monitoring of colony activity c. subsequent management: -Ecological restoration: * After the removal of the honeycomb, Assess the impact on local ecology * Measures if necessary to restore ecological balance (e. g. growing plants that attract pollinators) -Preventive measures: * Transformationenvironment, Reduce sites suitable for nesting * regularly check high-risk areas, Early detection of new beehives-Education and training: * educate the community on bee knowledge and safety prevention * Train local personnel for routine monitoring and initial treatment-long-term monitoring: * Establish a long-term monitoring system, Track swarm activities * periodically assess the effects of management measures, And timely adjust the strategy d. Special considerations: -for beneficial insects such as bees, Prioritize relocation over destruction-in agricultural areas, Need to balance protection requirements and pollination requirements-consider seasonal factors, Choose the appropriate time for management 3. Eco-friendly alternatives: in some cases, Consider alternatives that do not need to be removed directly: -Setting bee habitats: create a suitable habitat far from human activity areas, Natural migration of bee colonies-Use of sound or light repellent devices: Equipment installed in high-risk areas that do not harm the colony but can effectively drive away-Planting natural bee repellent plants: Planting bee undesirable plants around human activity areas as natural barriers When implementing these measures, The most important thing is to balance the needs of human security and ecological protection. The situation may vary per region, so management strategies should be adjusted to suit. At the same time, continuous monitoring and assessment are also essential to ensure that these measures are effective in the long term. |
