# Supplementary material for: Clinical Management of Wasp Stings Using Large Language Models: Cross-Sectional Evaluation Study
Source: J Med Internet Res. 2025 Jun 4;27:e67489. doi: 10.2196/67489 (PMC12177424; doi:10.2196/67489)
Supplement: Multimedia Appendix 4 [file jmir_v27i1e67489_app4.docx]

| Engine 4 | | | | | |
| --- | --- | --- | --- | --- | --- |
|  | Basic Knowledge | Basic Knowledge | Basic Knowledge | Basic Knowledge | Basic Knowledge |
| Question | What are the common types of bee stings worldwide? | What are the main pathophysiological mechanisms of bee stings? | What are the main components of bee venom and their biological effects? | What are the global epidemiological characteristics of bee stings? | What are the common local and systemic reactions after bee sting? |
| Answer Engine 4 （ChatGPT4.0） | Sings, usually those that cause painful stimuli in humans. There are several common stings around the world, including bees (Apis spp.): Common bees are usually docile, but defend with stings if they feel threatened or their nests are attacked. Honeybee stings have barbs that remain in the skin, causing the bee to die after the sting. Wasps (Vespula spp. and Dolichovespula spp.): These bees are more aggressive and can sting people many times without death. Their stings are more painful and sometimes cause allergic reactions. Hornet (Vespa spp.): Hornets are larger, sting longer, can repeat many times. Their venom is strong, stings are severe and can cause severe allergic reactions. Earth bees (Sphecidae): This type of bees usually do not attack humans, but they can also be stung if they touch their nests or interfere with them. These bees are widely distributed in different geographical environments and are found from urban to rural areas. The best defense against these bees is to avoid disturbing their nests and keep a distance. If stung, the wound should be treated immediately and observed for signs of an allergic reaction, and seek medical help if necessary. | The main pathophysiology of bee stings involves several key aspects, mainly due to the local and systemic reactions caused by bee venom. The following are detailed instructions: 1. The release of chemical substances: bee venom contains a variety of bioactive substances, such as bee venom peptide (such as bee venom kinin), histamine, phospholipase A2, etc. These substances are rapidly released rapidly at the sting site, causing direct cellular damage and inflammatory responses.2. Local inflammatory reaction: vascular expansion and increased vascular permeability: histamine and other inflammatory mediators in bee venom can rapidly dilate blood vessels and increase the permeability of capillaries, leading to local redness and pain. Leukocyte infiltration: inflammatory mediators attract leukocytes (such as neutrophils and macrophages) to the site of stings, which participate in the inflammatory response and the clearance of damaged tissues.3. Systemic reactions: For some people, bee venom may also cause systemic anaphylaxis, or anaphylactic shock. This response involves an overresponse of the immune system to specific proteins in bee venom. Features of anaphylactic shock include extensive vascular dilation, sudden drop in blood pressure, dyspnea, that can lead to death when severe.4. Pain and tissue damage: Some components of bee venom (such as bee kinin) can directly stimulate nerve endings and cause severe pain. Phospholipase A2 can disrupt the phospholipids of the cell membrane, causing cell death and tissue damage. Therefore, the management of bee stings usually involves immediate removal of bee needles (if remaining in the skin), use of cold compress to relieve pain and swelling, application of antihistamines to reduce inflammatory responses, and rapid use of adrenaline and other emergency measures when there is a systemic response. | Bee venom is a complex mixture of various peptides, enzymes, bioactive amino acids, volatile compounds and other small molecules. These components work together to cause local and systemic reactions during bee stings. The following are some of the main components of bee venom and their biological effects: 1. Bee venom peptide (Melittin): Description: The most abundant components of bee venom, accounting for more than 50%. It is a potent and potent hemolytic agent. Biological effects: it can destroy the membrane of red blood cells and other cells, causing cell death. Meanwhile, it is also a strong inflammatory inducer that activates inflammatory pathways in the body and increase the release of cytokines, causing redness and pain.2. Phospholipase A2 (Phospholipase A2): Description: Another major component of bee venom is responsible for the release of arachidonic acid and the generation of prostaglandins and leukotrienes, which are key mediators of inflammation. Biological effects: destruction of cell membrane phospholipids, causing local pain and tissue damage. Moreover, it stimulates the immune system and causing a broader inflammatory response.3. Peptide 400 (Apamin): Description: Small peptides in bee venom that can block certain calcium-dependent potassium channels. Biological effects: it has an effect on the nervous system, and can enhance the excitability of the nerve endings, leading to increased pain sensation.4. Protokinin (Mast cell degranulating peptide, MCD): Description: Promote degranulation of mast cells and release histamine. Biological effects: causing rapid and intense inflammatory reactions, associated with redness, heat and pain.5. Histamine: Description: A small molecule in which mast cells are released when stimulated. Biological effects: causing vascular dilatation and increased vascular permeability, leading to local swelling and redness.6. Dopamine and norepinephrine: Description: These are neurotransmitters, also present in bee venom. Biological effects: it can cause vasoconstriction and may participate in the regulation of local blood flow changes caused by inflammation. The synergistic action of these components creates typical symptoms after bee stings, such as pain, redness, heat, and sometimes allergic reactions. Understanding these components and their mechanisms of action can to more effective management and relief of symptoms when treating bee stings. | Bee stings occur widely worldwide, but the epidemiological characteristics vary by geography, environment, season, and human activity. Here are some key epidemiological features: 1. Geographic Distribution: The wasps are widely distributed around the world, especially in temperate and tropical regions. Different regions may have different bee species, such as wasps in the Americas and honey bees in Europe. Bee are distributed in both urban and rural areas, but bee stings in rural, forest or near natural environments may be more frequent due to more agricultural and outdoor activities.2. Seasonality: Bee stings are usually clearly seasonal, mostly from late spring to early autumn, when bees are most active. In warm months, people are more outdoors, and bees are building nests and foraging, which increases the chance of human contact with bees.3. Population exposure: People working in outdoor jobs such as horticulture, forestry and agriculture have a higher risk of sting. Children and outdoor athletes (such as hikers and campers) are also more vulnerable to bee stings.4. Medical consequences: Most bee stings cause local reactions, such as pain, redness, and itching. However, individuals allergic to bee venom may experience more severe systemic reactions or even fatal anaphylactic shock. It is estimated that anaphylactic reactions represent about a small proportion of all bee sting injuries but pose a serious threat to the victims.5. Prevention and intervention: Strategies to prevent bee stings include wearing protective clothing, avoiding disturbed activities in active bee areas, and using insect repellents when necessary. Carrying an emergency medication such as an adrenaline autoinjector (Epipen) is a recommended prevention measure for people with a known allergy to bee venom. Overall, although bee stings are a global problem, their incidence and severity can be significantly reduced with appropriate preventive measures and education. Timely and appropriate medical intervention is key for stings that have already occurred. | Bee stings can cause a variety of local and systemic reactions that can range in severity from mild to life threatening.1. Local reaction Pain: Sharp pain felt immediately after the bee sting is usually the initial response, as the toxins in the bee venom directly stimulate the nerve.redness and itching: The sting site quickly redness and swollen, because the toxin causes vascular dilation and increased permeability. Itch is due to the release of histamine. Heat and tenderness: The affected area may feel warm and be sensitive to touch, which is related to the inflammatory response. Local bleeding or bruising: If the sting penetrates deeper or touches the blood vessel, there may be minor bleeding or bruising.2. Systemic reaction Anaphylactic reaction (i. e., anaphylactic shock): mild: including rash, urticaria (generalized rash and itching), swollen eyelids and lips. Moderate: dyspnea, hoarseness, throat swelling, possibly accompanied by mild hypotension. Severe: rapid drop in blood pressure, severe dyspnea, loss of consciousness or other symptoms of shock requiring emergency medical intervention. Dizziness and nausea: Some people may experience systemic reactions such as dizziness, nausea or even vomiting. Fever and chills: Under the influence of a systemic inflammatory response. Anxiety or panic: Stings may cause acute anxiety or panic states, especially in children and people who have experienced such events for the first time.3. Local treatment: The wound should be cleaned immediately, and ice may be used to reduce swelling and pain. Avoid stimulating the sting site to reduce further spread of the toxin. Drugs: Antihistamines can help relieve redness and itching. For cases of severe pain, over-the-counter pain medication may be required either orally or locally. Emergency management of systemic reactions: In case of systemic allergic reaction, an adrenaline autosyringe (e. g. Epipen) should be used immediately and sent to the hospital for emergency treatment as soon as possible. In short, bee stings, while mostly mild, can be life-threatening for people with allergies. Therefore, it is very important to understand these responses and respond in a timely manner. |
|  | Early Treatment | Early Treatment | Early Treatment | Early Treatment | Early Treatment |
| Question | What is the first step after the wasp sting? | What is the difference between bee stings and wasp stings? How to accurately identify and judge it? | How to clean the wound of bee sting correctly? How to choose the cleaning liquid? | How to deal with local pain after a wasp sting? | What is the procedures of field first aid for bee stings? |
| Answer Engine 4 （ChatGPT4.0） | The first step after wasp sting should be rapid and careful to reduce pain and prevent infection. Here are the steps to take: 1. Keep calm and stay away from the wasp: First, make sure that yourself and the injured person are kept away from the wasps area to avoid further stings.2. Check and remove the sting needle: If the sting remains on the skin, remove it as soon as possible. The wasp sting needles are do not remain in the skin, but if so, can be gently extracted using forceps. Avoid the use of hand extrusion as this may squeeze more venom into the skin.3. Clean the wounds: Use soap and water to thoroughly clean the stings to remove dirt and bacteria from the surface and reduce the risk of infection.4. Cold compress to relieve pain and swelling: Use ice or a cold compress bag in a clean cloth and apply it to the injured area for about 10 to 20 minutes. Avoid applying ice directly to your skin to prevent frostbite.5. Observe symptoms: Watch closely for signs of systemic reactions, such as widespread rash, dyspnea, dysphagia, or facial swelling. These symptoms may indicate a severe allergic reaction.6. Symptomatic treatment: If the wound is very itchy or painful, use over-the-counter antihistamines or pain relief drugs. If necessary, an ointment containing hydrocortisone can be applied locally to reduce inflammation and pruritus.7. Seek medical help: If the injured person develops symptoms of any systemic reaction or has a known allergy to bee venom, call an ambulance immediately. A doctor should also be consulted if there is no significant allergic reaction, but symptoms do not improve or worsen within a few hours. These measures can effectively manage wasp stings, relieve discomfort, and prevent possible complications. Understanding these emergency steps is equally important outdoors and can respond quickly in an emergency. | There may be some differences between bee and wasp stings, mainly because of their differences in anatomical structure and behavior. Here are the key points to identify and judge these two stings: 1. Bee stings remain in the skin: The bee stings have a barb, so they usually remain in the skin when the sting, and the bee dies after the sting because part of the sting is pulled out. Wound characteristics: A small black spot (sting) may be seen at the sting, which may be surrounded by redness and pain. The bee bag may also continue to inject venom for a few seconds until it is completely removed. Treatment: The first treatment is to use forceps or scratches to gently scrape the sting out or clip out of the skin to avoid squeezing the bee poison bag.2. Bee stings do not remain in the skin: wasps and many other species of wasps can sting multiple times because their sting are not barbed and do not remain in the skin. Wound characteristics: Stings usually present as severe pain, redness and heat, but leave no obvious signs of the sting. Treatment: Treatment of wasp stings is mainly to relieve symptoms, such as cold compress to reduce swelling and pain, and to use antihistamines to treat local inflammatory reactions.3. Accurate identification and judgment To accurately identify bee and wasp stings, a direct indication can be given by checking whether there is a sting left in the wound. Moreover, it is helpful to observe the environment in which stings occur, for example, bees often appear in gardens or near hives, while wasps may be more aggressive and often actively defend territory in multiple environments. If the type of sting is not certain, or if the injured person has allergic symptoms, seek medical help immediately. Correct identification of stings types facilitates the most effective emergency aid and subsequent management. | Proper cleaning of bee stings is very important, which reduces the risk of infection and helps the wound heal. Here are the steps to clean bee stings and the choice of cleaning solution: 1. Step Safety First: Make sure yourself and the injured are away from the possible areas where the bees continue to attack. Remove the sting: If the bee stings remain in the skin, the forceps should be scraped gently on the edge of the credit card. Avoid squeezing the skin near the sting, as this may squeeze more venom into it. Clean the wound: Wash the wound thoroughly with gentle soap and water. Gently wash to avoid overrubbing the wound as this may cause more pain or damage. Cold compress: After washing, you can use a cold compress bag or wrap ice around the cloth to the wound to reduce swelling and relieve pain.2. Choose water and soap: Usually, plain water and mild non-antibacterial soap are enough to clean most injuries, including bee stings. The use of mild soap helps remove dirt and possibly pathogenic bacteria without stimulating already inflamed skin. Saline: If possible, use saline (saline solution) to clean the wound, especially for sensitive skin or when clean water is not available. Saline is a gentle washing option that cleans the wound without causing additional irritation. Avoid use: Intense disinfectants such as iodine, hydrogen peroxide, or alcohol are not recommended to clean bee stings directly because these substances may stimulate the wound and delay the healing process.3. Additional attention to keeping bee stings important after handling and cleaning them dry and clean. Medical help should be sought if the wound shows signs of increased redness, persistent pain, fever, or exudate, which may be a sign of infection. With these steps and recommendations, bee sting wounds can be effectively managed, promote their healing, and reduce the risk of complications. | Methods of managing local pain after a wasp sting include immediate first aid as well as some relief that can be done at home. Here are the detailed steps and suggestions: 1. Take immediate measures to clean the wound: First, clean the wound with soap and water, which helps remove toxins and dirt from the surface and reduce the risk of infection. Cold compress: Apply a cold compress bag or wrap ice cubes in a clean cloth to the injured area. Cold compresses are effective in reducing the pain and swelling. Apply for 10 to 20 minutes, repeat every hour, taking care to avoid placing ice directly on the skin to prevent frostbite.2. Pharmacy Use of over-the-counter painkillers: such as acetaminophen (Tylenol) or nonsteroidal anti-inflammatory drugs (such as ibuprofen or naproxen). These drugs can help reduce pain and fight inflammation. Topical analgesic cream: Use a local anesthetic cream containing lidocaine or benzocaine, which can be applied directly to the wound to relieve pain.3. Further care to avoid scratching: Keep the wound clean and dry, and avoid touching or scratching with your hands, as this may cause further irritation or infection. Observe symptoms: Pay close attention to the development of any systemic reactions, such as dyspnea, extensive rash, and throat swelling, which may be signs of severe allergic reactions and require immediate medical intervention.4. Long-term monitoring for wound healing: Keep watching the wound over the next few days. Medical help should be sought if the wound shows signs of redness, increased heat, persistent pain, or pus discharge. Through the above procedures, the local pain and related symptoms after wasp stings can be effectively handled. These measures can not only help relieve symptoms, but also speed up the recovery process. | The on-site emergency process for bee stings is very important to ensure that the injured are treated promptly and to prevent severe allergic reactions. Here is a recommended set of first aid procedures that can be performed immediately after bee stings occur: 1. Measure safety to ensure that you and the injured are away from the colony. Only after ensuring the safety of the site can the treatment be started. Remove the sting If a bee sting and the sting remains on the skin, remove it as soon as possible. Use scratches, pliers, or nails to avoid squeezing the bee capsule to reduce toxin injection. Clean the wound. Wash the wound thoroughly with soap and water to remove toxin residues and reduce the risk of infection.4. Cold compress Apply a cold bag or ice wrapped in a cloth or towel to the injured area to relieve pain and swelling. Cold compress every 10-20 minutes for several hours.5. Monitor the symptoms for signs of systemic reactions, such as dyspnea, throat swelling, syncope, generalized rash, etc. These symptoms may be harbents of anaphylactic shock.6. Pharmacy For pain and itching, over-the-counter antihistamines (e. g., diphenhydramine) and non-steroidal non-steroidal anti-inflammatory drugs (e. g., ibuprofen) may be considered.7. Emergency Medical Assistance If the victim shows any signs of an allergic reaction, immediately call an emergency call (e. g., 911 or a local emergency service number) for help. If the victim has a known history of insect toxin allergy and carries an adrenaline autosyringe (e. g. Epipen), use it immediately as instructed.8 Record and track During the waiting for the ambulance, record the symptom changes of the injured and any treatment measures already taken, which will help the emergency staff present better understand the situation.9. Follow-up observation Even if symptoms appear to resolve after initial treatment, the injured needs to be monitored for hours to days, as some reactions may be delayed. Through this process, you can effectively perform the initial first aid treatment for bee stings, while ensuring the safety and comfort of the injured person before receiving professional medical assistance. |
|  | Allergy Response Management | Allergy Response Management | Allergy Response Management | Allergy Response Management | Allergy Response Management |
| Question | What are the typical manifestations of allergic reactions caused by bee stings? | How to identify and urgently deal with the anaphylactic shock caused by bee stings? | What is the drug treatment plan of allergic reaction after bee sting? What is the first-line medication? | How to assess the risk of allergic reactions in patients with bee stings? | How to prevent and manage late hair-type allergic reaction after bee sting? |
| Answer Engine 4 （ChatGPT4.0） | Allergic reactions caused by bee stings can manifest from mild to life-threatening symptoms with severity varies depending on the individuals sensitivity to bee venom. Here are some typical manifestations of allergic reactions: 1. Mild to moderate reaction skin reactions: The most common manifestations include redness, itching, and urticaria (namely protruding erythema). These symptoms may be present at the sting site or throughout the body. Swelling: Significant swelling may occur in the sting site or in the surrounding area, such as the arm or ankle. In some cases, swelling of the eyelids, lips, tongue, or throat may also occur, which requires urgent management. Gastrointestinal symptoms: including nausea, abdominal pain, and vomiting. Severe reactions (anaphylactic shock, namely, systemic allergic reaction or anaphylactic shock) dyspnea: this may include wheezing, shortness of breath, or swollen throat obstruction. Cardiovascular symptoms: including decreased blood pressure and rapid heartbeat, which may cause syncope or coma. Sound changes: Swelling of the vocal cords may cause hoarseness or difficulty in speaking. Severe anxiety or panic: This is due to an emergency respiratory and circulatory response.2. Corresponding emergency measures immediate epinephrine: For individuals with symptoms of anaphylactic shock, immediately use an epinephrine autoinjector (such as Epipen), which is the most effective drug for treating anaphylactic shock. Emergency medical services: Call emergency services immediately for further medical assistance. Understanding the manifestations of these allergic reactions and emergency measures is critical to effectively. If you have a history of allergies, carrying an adrenaline syringe and telling people around you how to use it is a necessary prevention. | Anaphylactic shock (also known as systemic anaphylaxis or anaphylactic shock) is a severe systemic allergic reaction that can quickly be rapidly life-threatening. In the management of anaphylactic shock caused by bee stings, rapid recognition of symptoms and taking urgent measures are essential. The following steps are used to identify and handle anaphylactic shock caused by bee stings: 1. Symptoms of anaphylactic shock Symptoms of anaphylactic shock may include difficulty breathing: wheezing, shortness of breath, or respiratory obstruction caused by a swollen throat. Skin reaction: extensive urticaria, erythema, or skin fever and severe itching. Cardiovascular symptoms: a sudden drop in blood pressure, an accelerated heart rate, or dizziness, which can cause syncope. Digestive system reactions: nausea, abdominal pain, and vomiting. Neurological symptoms: anxiety, confusion, or dizziness. Swelling throat and voice changes: hoarseness or difficulty in speaking.2. Emergency treatment steps Immediate action: Once the symptoms of anaphylactic shock are recognized, take immediate action. Use epinephrine: If an available adrenaline autoinjector is available (such as Epipen) is available, use it immediately as indicated. Usually, injections should be given on the outside of the thigh and can be done directly through clothing. Call Emergency: Immediately after adrenaline, call the emergency service (e. g. 911) and indicate that anaphylactic shock has occurred and adrenaline has been administered. Keep the victim flat: If there is no head injury or breathing difficulties, leave the victim flat and have your legs raised to help maintain blood pressure. If there is difficulty in breathing, the injured person can be half seated to help with breathing. Keep the injured warm and comfortable: Keep the body temperature with a blanket or clothing. Continuous monitoring: Continuously monitor the breathing and state of consciousness before the ambulance arrives. Prepare for a second injection: If symptoms do not improve or symptoms recur within 5 minutes of the first injection and a second adrenaline autoinjector is available, a second injection can be performed. The key to emergency management of anaphylactic shock caused by bee stings is prompt recognition of symptoms and prompt use of adrenaline. Keep calm, act quickly, and ensure the injured are helped by professional medical professionals. For people with a history of allergies, bring an epinephrine autoinjector and inform them aroundHow the people use it is very important. | Anaphylaxis after bee stings can range from mild local reactions to severe systemic reactions, including anaphylactic shock. Drug regimen regimen based on the severity of the response. The following are some commonly used drug regimens and first-line drug options: 1. Mild to moderate anaphylaxis antihistamines, such as diphenhydramine (Benadryl), cyproheptadine (Zyrtec), fexofenadine (Allegra), and loratadine (Claritin). These medications can relieve itching, redness, and other mild allergic symptoms. Corticosteroids: Oral corticosteroids such as prednisone can be used.2. Severe anaphylactic reactions (including anaphylactic shock). Adrinephrine: it is the first-line medication for handling anaphylactic shock. Adrephrine rapidly reverses the symptoms of anaphylactic shock, including throat swelling, severe asthma, hypotension, and cardiovascular collapse. It is usually injected through an epinephrine autoinjector (such as a Epipen). More drug support: After epinephrine, additional drug support may be required, including: further antihistamines: intravenous antihistamines such as diprodrug treatment. Corticosteroids: Intravenous corticosteroids, such as methylprednisolone, can help reduce the ongoing inflammatory response. Bronchodilators: If severe dyspnea exists, use bronchodilators, such as salbutamol inhalants. The immediate use of epinephrine: The first and most critical treatment for any possible symptoms of anaphylactic shock is the immediate use of epinephrine. Emergency medical assistance: After epinephrine use, emergency medical services must be contacted immediately for further treatment and monitoring. Continuous monitoring: Allergic reactions may be recurrent and require continuous monitoring up to a few hours in medical facilities to ensure that the symptoms are fully controlled. In conclusion, treatment options need to be adjusted for the severity of the response. For severe anaphylactic shock, epinephrine is the only recommended first-line emergency medication. | Assof the risk of allergic reactions in bee sting patients involves careful questioning and analysis of the patients history, the presentation of symptoms, and the history of allergy. This information can help medical professionals or first responders predict and, appropriately, cope with possible allergic reactions. Here are some key steps to assess the risk of anaphylaxis: 1. Ask about the medical history of previous anaphylaxis: Ask the patient for a history of previous anaphylaxis caused by bee stings, especially severe systemic reactions, such as anaphylactic shock. Family allergy history: Ask if a family member has a history of allergies to insect bites or stings, because there may be a genetic tendency for allergic reactions. Other allergic symptoms: Ask the patient for other types of allergy symptoms, such as known allergies to drugs, foods, or other substances.2. Observe the size of symptom development: assess the severity of local reactions, such as redness greater than ordinary local reactions (more than 10 cm in diameter) may indicate a higher risk of systemic response. Rapid progression of symptoms: monitor for the rapid deterioration of symptoms, such as extensive urticaria, swelling, and dyspnea in a short period of time.3. Assess respiratory and circulatory conditions Respiratory problems: Be aware of any signs of difficulty breathing, such as wheezing, shortness of breath, or swollen throat, which are important indicators for urgent treatment. Circulating status: Observe for decreased blood pressure or other signs of an affected circulatory system.4. Use assessment tools and test skin tests: For patients who have had an allergic reaction to insects, sensitivity to specific bee venom can be assessed by skin allergy tests in non-emergency situations. Blood tests: Measuring specific IgE antibody levels can also help assess the risk of allergic reactions to bee venom.adrenaline autoinjectors: For patients with a history of severe systemic allergic reactions, it is recommended to take epinephrine autoinjectors with them and ensure that they and their family and friends know how to use them. Medical warning bracelet: It is recommended to wear a medical warning bracelet or necklace to inform emergency medical personnel of their allergies. Through these assessment steps, the medical providers and the patients themselves can better understand the risk of allergic reactionsTake appropriate preventive and coping measures to reduce the potential severity of allergic reactions and promote safe treatment. | Late-onset anaphylaxis is a condition that can occur after bee stings, usually a few hours to days after stings. The prevention and management of this response requires careful observation and appropriate response. Here are some key steps to prevent and manage late anaphylaxis after bee stings: 1. Preventive measures Avoid contact: Wear long-sleeved clothes and trousers, wear hats and gloves, especially when working in gardens or outdoors in areas where bees are active. Use repellant repellent or other bee protection measures such as honeycomb traps. Understanding and identifying bees: Understand common bees and their behavior, identify possible hives or active areas, and avoid these areas. Give a special warning to people with a history of allergies: if you are known to be venom, carry an adrenaline autosyringe (e. g., Epipen) and ensure that you and those around you know how to use it in an emergency. Consider conducting educational training to understand how to identify and manage allergic reactions.2. Management strategy to monitor symptoms: After bee stings, patients should closely monitor any unusual symptoms, especially in the first few days. Late-onset anaphylaxis may present with redness, pain, itching, or more severe systemic symptoms. Record changes in symptoms: To document any new symptoms, including their development time, duration, and the effects of any self-management measures. Use: For mild to moderate local symptoms, oral antihistamines (e. g., diphenhydramine, cyproheptadmidine) and / or non-steroidal anti-inflammatory drugs (e. g., ibuprofen) can be used to manage pain and inflammation. Medical attention should be sought for more severe symptoms and may require more aggressive treatment such as corticosteroids. Emergency medical assistance: If any systemic symptoms, such as dyspnea, extensive skin rash, throat swelling, or any signs of decreased blood pressure (e. g. dizziness or syncope) occur, immediately seek emergency medical assistance and use an adrenaline autoinjector. Through these preventive and management measures, the risk of delayed anaphylaxis after bee stings can be effectively reduced and used to provide patients with necessary support and intervention to avoid serious consequences. |
|  | Complication Management | Complication Management | Complication Management | Complication Management | Complication Management |
| Question | What serious complications may occur after a bee sting? | What are the causes of respiratory failure caused by bee stings? How to deal with? | What is the management strategy for renal insufficiency after bee stings? | What does the reason of bee sting secondary hair anemia have? How to deal with? | What is the way of blood purification treatment after bee sting? How to choose? |
| Answer Engine 4 （ChatGPT4.0） | 1. Bee sting causes only mild local reactions in most cases, but in some cases it can cause serious complications. These complications may include: anaphylactic shock (anaphylactic shock): This is the most severe reaction, an acute systemic reaction that can quickly be very life-threatening. It includes severe skin reactions, dyspnea, throat swelling, significant blood pressure drop, and fainting. And, if not treated quickly, can be fatal. Severe local reaction: Large redness in the sting, which usually lasts more than 24 hours and can be very painful and uncomfortable. Infection: Bacterial infection may occur if improperly improperly. Secondary infection can cause redness, warmth, accumulation of pus, and fever. Serum disease: Serum disease is an immune complex disease that usually occurs about a week after bee stings. Symptoms may include fever, joint pain, skin rash, and swelling. Symptoms: Bee venom itself contains a variety of toxins that may affect the heart and nervous system. In rare cases, a strong toxin reaction can cause dizziness, difficulty breathing, rapid heartbeat, or other cardiovascular problems. Neurological response: In some rare cases, bee stings may trigger a neurological response, such as the persistence of pain, or even develop encephalitis symptoms.2. Prevention and treatment In order to prevent and reduce serious complications, appropriate measures should be taken immediately after bee stings: ensure that the wound is cleaned quickly and cold compress to reduce inflammation. Use appropriate over-the-counter pain medication and antihistamines to relieve symptoms. For individuals with a known history of allergies, an adrenaline autoinjector should be carried and used in the first onset of a systemic reaction. If the symptoms persist or worsen, medical help should be sought immediately. With timely response and appropriate treatment, bee stings can be effectively managed and reduce the risk of severe complications. | Respiratory failure caused by bee stings is usually due to anaphylactic shock or a severe allergic reaction that may rapidly affect the respiratory system. This condition is urgent and potentially life-threatening and requires immediate action.1. Cause of throat swelling: An allergic reaction may cause rapid swelling in the soft tissue of the throat and trachea, blocking the air passage. Bronchospasm: Bee venom can trigger spasm of the trachea, narrowing the airway, which is especially dangerous for patients with asthma. Pulmonary edema: Severe allergic reactions may cause fluid build-up in the lungs and affect normal gas exchange. Anaphylactic shock: A systemic allergic reaction causes a sudden drop in blood pressure and reduced heart output, thus reducing blood flow to the lungs and affecting the oxygen supply.2. Treatment Inchose epinephrine: For patients who start to have dyspnea or other anaphylactic shock symptoms, immediately use an epinephrine autoinjector (e. g., Epipen). Adrinephrine can quickly relieve airway inflammation, open the airway, and stabilize blood pressure. Call emergency medical service: Call the emergency call immediately for help. Even if the symptoms are temporarily relieved, further evaluation and treatment will require by the medical personnel. Keep the respiratory tract open: Try to keep the patient in a semi-seated posture, which helps in breathing. If the patient loses consciousness, measures should be taken to prevent the posterior tongue drop from blocking the airway. Oxygen: If conditions permit, oxygen can help improve oxygenation and support breathing. Close monitoring: Closely monitor the patients respiratory status and awareness level while waiting for the ambulance to arrive. Prepare for cardiopulmonary resuscitation (CPR) if the patient stops breathing or heartbeat. Avoid sensitization: ensure the patient is away from further bee stings or other possible allergens.3. Preventive measures For people known to be allergic to bee venom, carrying an epinephrine autoinjector and wearing a medical warning bracelet are important measures to prevent severe allergic reactions. In addition, how educators, friends and colleagues use an adrenaline autoinjector in an emergency is also crucial. In conclusion, respiratory failure induced by bee stings is a medical emergency that needs to be rapid and effectiveEmergency response to save lives. | Renal dysfunction caused by bee sting is a rare complication that may arise due to anaphylactic shock or direct toxic effects of bee venom. Handling of this condition requires close monitoring and treatment in the hospital setting. The following are management strategies for renal dysfunction after bee stings: 1. Timely assessment and monitoring in the emergency room or hospital, with a comprehensive physical assessment, including detailed measurements of vital signs (e. g. blood pressure, heart rate, respiratory rate) and laboratory tests. Blood tests may include renal function indicators (blood urea nitrogen and creatinine levels), electrolytes, complete blood cell counts, liver function tests, and inflammatory markers. Urine analysis may help to assess the extent and type of kidney injury.2. Maintaining blood flow and blood pressure Ensuring adequate circulating blood volume and maintaining a stable blood pressure are the key to managing renal insufficiency. The use of intravenous fluid resuscitation and vasoactive drugs may be required.3. Avoid nephrotoxic drugs and substances. Avoid the use of drugs that may aggravate kidney injury, such as certain antibiotics, non-steroidal anti-inflammatory drugs, etc.4. Symptomatic supportive treatment According to the specific symptoms and renal function indicators of patients. For example, controlling for hypertension, adjusting for electrolyte imbalance, and using appropriate antibiotics for secondary infections.5. Renal replacement therapy If renal function deteriorates dramatically and cannot be restored with conventional therapy, renal replacement therapy, such as dialysis, may need to be considered.6. Monitor complications Closely monitor possible complications, such as electrolyte imbalance, abnormal acid-base balance, and uremic symptoms.7 Multidisciplinary teamwork The management of renal dysfunction may require multidisciplinary teamwork, including emergency physicians, nephrologists, pharmacists, nurses, and other health professionals.8 Patient and family education educate the patient and family members about information, treatment options and prognosis of renal insufficiency, and how to identify potential complications. Management of renal dysfunction caused by bee stings requires a high degree of alertness and timely intervention. Ensuring appropriate assessment and treatment in medical facilities is essential to prevent deterioration and to maximise restoration of renal function. | Secondary anemia caused by bee stings is a relatively rare situation, and the main reasons may include the following aspects: 1. Cause of immune-mediated hemolysis: bee venom can cause abnormal reaction of the immune system, leading to the body to mistakenly attack its own red blood cells, causing hemolytic anemia. Acute inflammatory response: Acute inflammation caused by bee stings can interfere with iron metabolism, leading to the so-called inflammatory anemia. Direct toxic effects: Some components of bee venom may directly damage the red blood cells, leading to the rupture of the red blood cells.2. Treatment method for the secondary anemia caused by bee stings, the treatment method should be based on the specific reasons, usually including: medical assessment: after the occurrence of anemia symptoms (such as pale skin, fatigue, rapid heartbeat, etc.), should immediately seek medical treatment for a comprehensive assessment. The doctor may request laboratory tests for a complete blood cell count, reticulocyte count, hemoglobin level, and serum iron and ferritin levels. Symptomatic supportive care: Depending on the severity of anemia, blood transfusion may be needed to rapidly raise red blood cell levels. Using iron and vitamin supplements, especially in the case of inflammatory anemia, helps with the recovery of red blood cells. Specific treatment for hemolytic anemia: If the anemia is caused by hemolysis, the use of steroids or other immunosuppressants may be required to reduce the attack of the immune system on red blood cells. In some cases, plasmapheresis may be required to remove circulating immune complexes and bee venom components. Monitoring and follow-up: Continue to monitor changes in blood parameters and symptoms and adjust treatment options. Focus on any possible complications, such as impaired renal function.3. Preventive measures Preventing bee stings is critical for individuals known to be allergic to bee venom. This includes avoiding high-risk areas, wearing protective clothing, and using bee repellents when necessary. Carry emergency medication, such as epinephrine autoinjectors, to respond quickly in the event of severe allergic reactions. In general, for anemia occurring after bee stings, it is important to identify and seek medical help as soon as possible in order to receive appropriate timely treatment and intervention. | The use of blood purification treatment after bee stings is considered in rare cases, cases of severe complications or severe poisoning. These conditions may include severe allergic reactions, toxin-mediated organ failure, or immune-mediated responses such as hemolytic anemia. The goal of blood purification therapy is to quickly remove toxins, immune complexes or inflammatory mediators from the blood to help restore normal physiological function. The following are some available blood purification treatments and their options: 1. Blood purification treatments 1.1 Plasma exchange (Plasmapheresis) plasma exchange by replacing the patients plasma, removing antibodies, circulating immune complexes, toxins and other pathological substances. It is commonly used to manage immune-mediated hemolytic anemia or other immunocomplex symptoms due to bee venom.1.2 Hemodialysis Hemodialysis removes waste and excessive water through a semi-permeable membrane and is usually used to handle acute or chronic renal failure caused by bee stings. Dialysis is able to help deal with metabolic abnormalities due to toxin accumulation.1.3 Continuous Renal Replacement Therapy (CRRT) CRRT is a continuous blood purification therapy for patients with hemodynamic instability and allows more gentle removal of toxins and inflammatory mediators in the body. For the treatment of acute kidney injury and other severe metabolic disorders.2. How to choose the appropriate blood purification method depends on several factors: 2.1 Clinical condition of the patient: the haemodynamic stability of the patient, renal function status, and the degree of damage to the organ system involved.2.2 Nature and severity of specific complications: The specific type of complications (e. g., hemolysis, renal injury, severe allergic reaction) will guide which purification technology is used.2.3 Emergency of treatment: Rapid deployment, such as plasma exchange, of therapy may be needed to quickly remove circulating toxins in the emergency.2.4 Available medical resources: Hospital equipment and technical capabilities will also influence the treatment options available for adoption.2.5 Professional judgment and suggestions of doctors: provide personalized treatment suggestions according to doctors assessment and experience of the condition and the specific situation of patients.3. Conclusion Considering blood purification treatment after bee stings is severe toxicity in patientsSelection when response or life-threatening complications. Treatment decisions should be made by an experienced medical team based on the patients specific condition and treatment response. In this case, work closely with professional healthcare providers to ensure that patients receive the most appropriate and effective treatment. |
|  | Severity Assessment | Severity Assessment | Severity Assessment | Severity Assessment | Severity Assessment |
| Question | What symptoms after the bee sting suggest that the condition is serious? | How to assess the risk of multiple-organ function damage after a bee sting? | How should bee stings patients appear when soy sauce color urine respond? | What is the significance of the neurological symptoms in bee sting patients? | How to judge whether bee sting patients need hospitalization? |
| Answer Engine 4 （ChatGPT4.0） | After a bee sting, if the following symptoms occur, it indicates that the condition may be serious and requires immediate medical treatment or emergency treatment measures. These symptoms often indicate a risk of anaphylactic shock, severe local reactions, or organ damage.1. Symptoms of dyspnea: wheezing, shortness of breath, throat swelling, hoarseness, or chest tightness. Note: These symptoms may indicate airway obstruction or anaphylactic shock, requiring the immediate use of an adrenaline autoinjector and a call for emergency medical services.2. Severe swelling symptoms: The swelling at the sting site rapidly expands beyond the normal local reaction range, especially around the face, tongue, throat, or eyes. Note: It may cause respiratory tract obstruction or other serious complications and require emergency management.3. Systemic reaction symptoms: extensive urticaria (rash), itching, skin flushing, or generalized erythema. Note: These may be early signs of systemic allergic reactions and require close monitoring and rapid intervention.4. Symptoms of hypotension and syncope: dizziness, weakness, rapid heart rate, confusion, or loss of consciousness. Note: A sudden drop in blood pressure may indicate the occurrence of anaphylactic shock, with immediate use of adrenaline and seeking emergency medical assistance.5. Persistent or severe pain symptoms: The pain at the site of the sting continues to worsen for more than a few hours, or is accompanied by severe redness, tenderness, or purulent signs of infection. Note: Severe local infection or tissue damage may occur, requiring medical assessment and treatment.6. Reduced urine volume or no urinary symptoms: significantly reduced urine volume or stop urination. Note: It may be a manifestation of impaired renal function, especially after multiple bee stings or severe reactions.7. Symptoms of nausea, vomiting, and abdominal pain: severe nausea, vomiting, or severe abdominal pain, along with other systemic symptoms. Note: These symptoms may indicate systemic toxicity or organ damage.8. Symptoms of jaundice or pallor: yellow skin or eyes, or pale skin all over the body. Note: It may be signs of hemolytic anemia or impaired liver function.9. Symptoms of changing consciousness: confusion, lethargy, coma, or other neurological symptoms. Note: It may indicate a severe systemic response, requiring urgent medical intervention. If inAny of the above symptoms occurring after a bee sting may indicate that the condition may be severe, and the patient should take immediate emergency measures, such as the use of an adrenaline autoinjector, and promptly seek medical assistance. Early recognition of these symptoms and rapid management is critical to preventing serious complications and ensuring patient safety. | Assof the risk of multiple organ function impairment after bee sting requires consideration of the patients clinical presentation, laboratory findings, and past medical history. Multiple organ function impairment (MODS) usually occurs after severe allergic reactions or systemic toxicity caused by bee venom, so timely identification and assessment of risks is critical. The following are the assessment methods and steps: 1. Assess the medical history and initial reaction past allergy history: Understand the patients past history of severe allergic reaction (such as anaphylactic shock). If so, this may increase the risk of developing multiorgan functional impairment. Number and location of stings: Patients with multiple stings or stings in sensitive sites (e. g. face, neck) are at higher risk. Initial symptoms: To assess symptoms immediately after a bee sting, such as dyspnea, hypotension, extensive rash, nausea, vomiting, which may indicate a systemic response.2. Vital signs Monitoring blood pressure: Persistent hypotension may indicate damage to the circulatory system. Heart rate: Tachycardia may be a sign of shock or heart damage, while bradycardia may indicate severe systemic poisoning. Respiratory rate and oxygen saturation: tachypnea or hypoxemia may indicate impaired lung function or the risk of respiratory distress syndrome (ARDS).3. Laboratory tests: Complete blood count: leukocytosis or decrease, thrombocytopenia may indicate systemic inflammation or myelosuppression. Hemoglobin and reticulocyte count: evidence of hemolytic anemia. Liver function test: transaminase (ALT, AST) and bilirubin levels: increased impaired liver function or hepatocyte necrosis. Renal function test: Serum creatinine and blood urea nitrogen (BUN) levels: an increased level indicates impaired renal function. Urine analysis: Proteinuria or hematuria may be an early marker of kidney injury. Cardiac function test: myocardial enzymes (e. g. troponin): elevation indicates myocardial injury. Electrolyte and acid-base balance: potassium, sodium, calcium levels, and arterial blood gas analysis: the imbalance may indicate multiple organ dysfunction.4. Organ function evaluation kidney: decreased urine volume (oliguria or anuria) suggests acute kidney injury (AKI). Liver: jaundice, liver enlargement, ascites and other signs indicate liver insufficiency. Heart: palpitations, chest pain, abnormal abnormalitiesSuggesting impaired cardiac function. Lung: Dyspnea and low oxygen saturation suggest the possibility of acute respiratory distress syndrome (ARDS). Nervous system: confusion, coma, or seizures indicate central nervous system damage.5. Imaging chest X-ray or CT scan: used to assess for acute injury to the lung, such as pulmonary edema or ARDS. Abdominal ultrasound or CT: evaluate of internal organs such as liver and kidney.6. Multidisciplinary consultation In severe cases, consultations from multiple specialists (e. g., intensive care, nephropathy, cardiology, respiratory departments, etc.) may be required to comprehensively assess and manage multiple organ function injuries.7. Continuous monitoring For high-risk patients, continuous monitoring of vital signs, urine volume and laboratory indicators, early identification of deterioration of organ function and appropriate intervention measures. Through the above assessment methods, the medical team can quickly identify the possible risk of multiple organ function damage after bee stings, and take corresponding treatment and management measures. This comprehensive assessment is essential to prevent the occurrence of serious complications and helps to improve patient outcomes. | Soy sauce-colored urine after bee sting is a serious symptom, usually suggesting the presence of hemolytic anemia or rhabdomyolysis. Both conditions can lead to the release of hemoglobin or myoglobin into the blood and eventually discharge through the urine, resulting in dark urine (e. g., soy sauce). This condition requires an emergency medical assessment and management. The following are the steps: 1. Seek immediate medical attention After soy sauce, patients should seek medical attention immediately after finding soy-colored urine. This symptom suggests that there may be serious medical problems and cannot be ignored.2. Medical and medical history: Ask the developmental history of symptoms, including injury site, number of stings, initial reaction and other symptoms (such as fatigue, chest pain, abdominal pain, etc.). Physical examination: The doctor will perform a detailed physical examination to assess for other systemic involvement, such as the liver, kidney, heart, and muscle.3. Laboratory urinalysis: to confirm the presence of hemoglobin or myoglobin in the urine, or to exclude or confirm hematuria. Blood test: Creatine kinase (CK) level: significantly increase suggesting rhabdomyolysis. Hemoglobin and reticulocyte count: used to assess the degree of hemolytic anemia. Renal function index (e. g. serum creatinine and urea nitrogen): used to assess whether renal function is impaired. Electrolyte and acid-base balance: abnormal electrolyte levels may indicate impaired renal function or severe metabolic problems.4. Early treatment of massive intravenous fluids: massive rehydration is a key step in the treatment of rhabdomyolysis and to prevent acute kidney injury (AKI). Rehydration can help to dilute the urine, reduce the toxic effects of myoglobin on the kidney, and maintain the normal function of the kidney. Alkalinizing urine: Alkalinizing urine with a sodium bicarbonate solution can help prevent the deposition of myoglobin in the renal tubules and further protect the kidneys. Surveillance and supportive care: Depending on the condition, correcting the electrolyte imbalance and diuretic administration (e. g., furosemide) may be required to increase urine volume.5. Further management and close monitoring of urine volume and renal function: Continuous monitoring of urine volume during treatment is an important indicator to evaluate the treatment effect and renal function during treatment. Electrocardiographic monitoring: If electrolytes are abnormal (such as hyperkalemia), ECG monitoring may be required to prevent arrhythmia. P. r. nDialysis: If acute renal failure occurs, dialysis may be an necessary intervention.6 Multidisciplinary collaboration in complex cases may require cooperation of multiple departments, including nephrology, emergency department, intensive care, and internal medicine. The presence of soy sauce-colored urine after a bee sting is a very serious warning sign indicating the possibility of hemolytic anemia or rhabdomyolysis. At this point, seek medical attention quickly for emergency assessment and treatment. Early intervention and appropriate management can prevent renal failure and other serious complications and greatly improve patient prognosis. | The onset of neurological symptoms after bee sting is a signal worth high attention, suggesting that several serious pathological mechanisms may exist. Different neurological symptoms may reflect different pathological processes, and the following are some possible conditions and their significance: 1. Anaphylactic shock and hypoxemia symptoms: confusion, dizziness, syncope, disorientation, seizures, etc. Implications: Hypotension and hypoxemia due to anaphylactic shock may affect oxygen supply to the brain, leading to these neurological symptoms. The persistence of hypoxemia causes brain damage that must be managed immediately.2. Neurotoxicity symptoms: numbness of the limbs, muscle weakness, facial paralysis, language difficulties, blurred vision, etc. Significance: Some components of bee venom, such as honeyvirus peptide (Melittin) or phospholipase A2, may have direct neurotoxic effects, leading to neurological dysfunction. Severe toxic reactions may affect the central nervous system or the peripheral nervous system.3. Acute renal failure caused by rhabdomyolysis, along with symptoms of metabolic disorders: changes of consciousness, lethargy, seizures, etc. Significance: Bee stings may lead to rhabdomyolysis and then cause acute renal failure. The resulting metabolic acidosis and electrolyte disorders (such as hyperkalemia) can affect the nervous system and cause these symptoms.4. Systemic inflammatory response syndrome (SIRS) or sepsis symptoms: altered mental state, disorientation, lethargy, coma, etc. Significance: Severe bee stings may cause systemic inflammatory response syndrome or sepsis. In these cases, inflammatory factors and toxins may cross the blood-brain barrier, leading to central nervous system involvement.5. Symptoms of immune-mediated nervous system injury: limb weakness, paresthesia, weakening or disappearance of reflexes, etc. Implications: Some patients may cause the immune system to attack their own nerve tissues, leading to demyelinating diseases such as Guillain-Barre syndrome (Guillain-Barre syndrome). Management method Emergency medical assessment: immediate systemic examination and neurological assessment. In emergency situations, stabilizing the patients vital signs is a top priority. Vital signs monitoring and support: Ensure the patients airway, respiratory and circulatory system stability. If necessary, makeWith oxygen or with mechanical ventilation to maintain adequate oxygenation. Laboratory and imaging tests: blood tests, including electrolytes, acid-base balance, liver and kidney function, blood glucose, etc., to exclude metabolic disorders. Cranial CT or MRI can help assess the presence of structural brain abnormalities or hemorrhage. Lumbar puncture (depending on the condition) may be used to rule out infection or inflammatory encephalopathy. Symptomatic treatment: If hypoxemia is suspected, give high-flow oxygen support immediately. If electrolyte disorders causes neurological symptoms, the electrolyte imbalance should be quickly corrected. For seizures, seizures were controlled with anti-seizure medication. Specific treatment: If neurotoxicity or immune-mediated neurological damage is suspected, treatment with steroids, immunosuppressants, or plasmapheresis may be required. The onset of neurological symptoms following a bee sting is a serious warning sign indicating the possibility of anaphylactic shock, neurotoxicity, metabolic disorders, or other systemic complications. Timely recognition of these symptoms and prompt adoption of appropriate treatment measures is critical to improving prognosis. | Make a comprehensive assessment of the patients clinical manifestations, medical history, laboratory findings, and the potential risk of complications. Here are some key considerations and guidelines: 1. Systemic anaphylaxis symptoms: including extensive urticaria, severe dyspnea, throat swelling, hypotension, confusion, syncope, etc. Guidelines: If a patient has anaphylactic shock or other severe systemic anaphylactic reaction, he or she must be hospitalized immediately for close monitoring and emergency intervention.2. Multiple or severe stings symptoms: multiple stings or local reactions are very serious, such as large area of swelling, increased pain, local necrosis, etc. Guidelines: Multiple stings may lead to cumulative toxin effects, increasing the risk of systemic reactions and organ damage. Such patients usually require hospitalization for observation.3. Symptoms of respiratory involvement: including signs of airway obstruction (such as laryngeal edema), bronchospasm, or shortness of breath. Guidelines: Any respiratory symptoms indicate a severe allergic reaction or a direct effect of bee venom, and hospitalization to ensure respiratory management and support.4. Symptoms of cardiovascular system instability: including hypotension, tachycardia or bradycardia, arrhythmia, etc. Guidelines: These symptoms may be signs of anaphylactic shock or heart damage and require hospital monitoring and treatment.5. Symptoms of abnormal renal function: decreased urine volume (oliguria or anuria), abnormal urine color (such as soy sauce color), and abnormal renal function indicators (such as increased creatinine). Guidelines: These symptoms may indicate acute kidney injury or rhabdomyolysis, requiring hospitalization for fluid management and possible dialysis treatment.6. Neurological symptoms: dizziness, altered consciousness, seizures, limb weakness, or paresthesia. Guidelines: Neurological symptoms indicate possible central nervous system or peripheral nerve involvement and require hospitalization for detailed evaluation and treatment.7. Abnormal laboratory examination results: serious laboratory abnormalities such as hypercreatine kinase (CK), hyperkalemia, hypocalcemia, acidosis, severe anemia, etc. Guidelines: These laboratory abnormalities may indicate potentially serious systemic problems, such as rhabdomyolysis, renal failure, or hemolytic anemia, as neededhospitalization.8. Comorbidities and overall health considerations: age, presence of chronic diseases (e. g., asthma, heart disease, diabetes), immune status (e. g., immunosuppression), etc. Guidelines: Elderly patients, immunocompromised patients with severe comorbidities, usually need hospitalization for observation and treatment due to their high risk of potential complications.9. Hospitalization conditions If the symptoms are mild: only local reaction, the symptoms are quickly relieved after initial treatment, and no other risk factors, patients may not need to be hospitalized, can be observed at home, and take medicine according to the doctors advice. Inpatient observation: If the symptoms are mild but developing or the patient is very psychologically anxious, the doctor may recommend short-term hospital observation to ensure safety. To determine whether bee stings need hospitalization is based on multiple factors, including the severity of symptoms, laboratory findings, past medical history, and the general health of the patient. Any patient who shows a systemic response, abnormal organ function, or has a high risk usually requires hospitalization to ensure timely and effective treatment and surveillance. |
|  | Special Population Management | Special Population Management | Special Population Management | Special Population Management | Special Population Management |
| Question | How can children be specially treated after bee stings? | What are the special nursing measures after bee stings in elderly patients? | What is the emergency treatment strategy after the pregnant mother bee sting? | What should patients with chronic diseases pay attention to after bee stings? | What is the processing point of bee sting after immune deficiency person? |
| Answer Engine 4 （ChatGPT4.0） | Children need special attention after bee stings because they may be more susceptible to severe reactions and have a limited ability to express symptoms. Here are the steps and precautions for handling child bee stings: 1. Stay away from the danger area immediately. First, take the child away from the colony or nest area to avoid being stung again.2. Check and remove the sting If it is a bee stings and the sting remains in the skin, gently scrape the sting with nails, credit card edge or blunt instrument as soon as possible to avoid squeezing the poison sac to reduce venom injection. Hernet stings usually leave no sting.3. Wash the wound thoroughly Wash the sting with gentle soap and water to remove toxins and bacteria and prevent infection.4. Cold compress in a cold bag or ice and apply it to the sting area for 10 to 20 minutes to relieve pain and swelling. Avoid applying ice cubes directly to the skin.5. Monitor allergic reactions for signs of allergic reactions, such as dyspnea, swollen throat, rash or generalized itching, swollen lips and tongue, dizziness, vomiting, etc. If any systemic symptoms develop, seek medical help immediately.6. Giving Antihistamines If a child develops local itching or mild swelling, the doctor may recommend the appropriate dose of antihistamine (e. g., diphenhydramine) to reduce these symptoms. Be sure to consult your doctor before using any medication.7. Pain Relief If the child feels pain, appropriate doses of painkillers (such as acetaminophen or ibuprofen) can be given to the child at the advice of the doctor.8 Keep calm and calm children Children may feel fear or anxiety after being stung. Parents or caregivers should remain calm and calm the children, telling them that everything is under control.9. Prevent scratching Tell children not to scratch the stung spots to avoid infection or further skin damage. If the itching is severe, reduce the symptoms by applying an anti-itching ointment or using a cold compress.10. Observe the childs symptoms closely for the next few hours to days. If any changes or symptoms worsen, seek medical attention immediately.11. Emergency treatment of severe allergic reactions if a child has a history of severe allergic reactions (such as anaphylactic shock) And carry an epinephrine autoinjector (such as Epipen), which should be used immediately and make an emergency call in case of severe reaction.12. If a child is stung by multiple bees, or is stung in sensitive areas like the face, neck, or throat, or if he has any systemic symptoms, seek medical attention for further evaluation and treatment as soon as possible.13 If a child has a known history of allergy, especially to insect venom, the doctor should be informed immediately so that they can quickly take appropriate measures.14. Prevention measures Teach children to stay away from beehives and avoid wearing bright colored clothes or using aromatic cosmetics, which may attract bees. Try to wear protective clothing for outdoor activities. Children are handled similar to adults, but with greater care and caution. Special attention to observe the early signs of allergic reaction, timely medical attention and comfort of children, is the key to ensure safe and rapid recovery. | Older patients require special care after bee stings because they tend to have chronic disease or a weak immune system and responses to drugs may differ from younger adults. The following are special care measures for elderly patients after bee stings: 1. Immediately assessment and treatment of sting removal: If it is a bee stings, check for the sting left in the skin and scrape the sting with nails and blunt edges (such as credit card edges) as soon as possible to avoid squeezing the sac. Clean the wound: Clean the wound thoroughly with gentle soap and water to prevent infection.2. Close monitoring of allergic reactions Older people may be more sensitive to bee venom, and allergic reactions may present as dyspnea, hypotension, systemic rash, or syncope. Watch closely for signs of systemic allergic reactions, especially swelling in the throat, chest pain, and shortness of breath. For any systemic symptoms, immediately use an adrenaline autoinjector (e. g. Epipen) and seek medical attention.3. Monitoring the cardiovascular system The elderly are usually at risk for cardiovascular disease, so special attention should be paid to symptoms such as hypotension, arrhythmia, chest pain or tachycardia. An ECG examination and blood pressure monitoring were performed when necessary.4. Manage pain and swelling: Cold compress: wrap a cold bag or ice to the stings to reduce swelling and pain. Pain medication: Use appropriate painkillers (such as acetaminophen) under the guidance of a doctor. Avoid using pain medication that may interact with the patients existing medication.5 Consider drug interactions Older patients may be taking multiple drugs, and the risk of drug interactions must be considered when using antihistamines or other therapeutic measures. Consult with a doctor or pharmacist if necessary.6. The elderly have weak immunity and higher risk of infection. Closely observe the wound for signs of infection, such as swelling, pus, fever, etc. If an infection is suspected, seek medical attention promptly and may require antibiotics.7. Evaluation and manage chronic diseases evaluate patients chronic diseases, such as diabetes and chronic cardiopulmonary diseases, to ensure that these diseases are well managed after bee stings and avoid complications.8 Renal function assessment The bee venom may affect renal function, particularly inIn the elderly patients. Urinary output and renal function indicators should be closely monitored, especially for patients with previous renal insufficiency.9. Mental comfort and psychological support Older people may feel extreme anxiety or panic about bee stings. Provide comfort and psychological support to help them stay calm and explain the nursing measures taken.10 Continuous monitoring of elderly patients may experience delayed reactions or secondary allergic reactions after initial treatment. Watch their condition closely, continue to monitor for any abnormal symptoms for several days, and ensure adequate follow-up.11 Maintaining proper hydration ensures adequate hydration in older patients, especially if at risk of hypotension or renal function.12. Education and prevention Teach patients and their families how to prevent future bee stings, including avoiding beehives, and wearing appropriate protective clothing outdoors. If the elderly have a known history of allergies, they are advised to carry an adrenaline autoinjector. Care of elderly patients after bee STings requires special attention to their systemic condition, including chronic disease, immune status, and risk of drug interactions. Close monitoring, timely treatment and psychological support are the key to ensure their safe recovery. | Pregnant women need to be particularly careful after bee stings, because not only to protect the health of the pregnant woman, but also to consider the safety of the fetus. The following are the emergency treatment strategies for pregnant women after bee stings: 1. Immediately stay away from the dangerous area and quickly take the pregnant woman away from the colony or honeycomb area to avoid further stings.2. Check and remove the sting If it is a bee sting, check if the sting remains in the skin. The sting was gently scraped with the nail or the edge of the credit card to avoid squeezing the sac to reduce venom injection. Hernet stings usually leave no sting. Clean the wound. Wash the wound thoroughly with mild soap and water to remove toxins and prevent infection.4. Apply a cold compress bag or wrap ice in a clean cloth and apply it to the sting area for 10 to 20 minutes to reduce swelling and pain. Avoid applying ice cubes directly to the skin to prevent frostbite.5. Closely monitor allergic reactions to observe whether the pregnant women have signs of allergic reactions, such as dyspnea, throat swelling, systemic urticaria, dizziness, syncope, etc. If any systemic symptoms, seek medical help immediately.6. Use epinephrine If a pregnant woman has a severe systemic allergic reaction (e. g., anaphylactic shock), immediately use an epinephrine autoinjector (e. g., Epipen), whose use is safe and life-saving. Seek medical attention immediately after use. Pregnant women should be careful to use drugs. Many common antihistamines and painkillers are caution during pregnancy. A physician should be consulted before drug use, especially given the potential impact of the drug on the fetus.8. Prevent infection Continue to observe the wound for signs of infection, such as swelling, fever, pus, etc. If signs of infection occur, seek medical attention immediately.9. Monitoring of fetal health should be closely monitored after bee stings, especially if a systemic reaction occurs or epinephrine is used. A doctor may recommend an ultrasound examination or other fetal monitoring measures to secure the fetus.10 Avoid stress and anxiety Bee stings may cause anxiety and tension in pregnant women, which is detrimental for the fetus. Try to be calm, and your family and friends should also give them comfort and support. If feeling excessive anxiety or panic,Should communicate with your doctor as soon as possible.11. Medical treatment and follow-up Even if the symptoms are mild, it is recommended to seek medical treatment as soon as possible after a bee sting. Physicians can assess potential risks and arrange further monitoring or treatment if necessary. Follow-up was recommended by the doctor to ensure that the health of the pregnant woman and the fetus is stable.12. Preventive measures Teach pregnant women how to prevent bee stings, including avoiding proximity to beehives and wearing appropriate protective clothing during outdoor activities. Postoperative management of bee stings in pregnant women requires special attention to the safety of them and the fetus. Timely wound cleaning, monitoring of allergic reactions, careful medication, and calm are key steps. If any doubts or symptoms occur, seek medical attention immediately and ensure follow-up care and monitoring under the guidance of your physician. | Patients with chronic diseases need special attention after bee stings because their bodies may be more susceptible to bee venom, or their pre-existing health conditions may aggravate the consequences of bee stings.1. Monitor systemic reactions and anaphylaxis: Observe closely for symptoms of systemic anaphylaxis, such as dyspnea, swollen throat, generalized rash, dizziness, or syncope. These responses may aggravate the effects of chronic disease. Response: In case of any severe allergic reaction, use an adrenaline autosyringe (e. g., Epipen) immediately and seek medical attention quickly.2. Pay attention to drug interactions Routine drugs: People with chronic diseases usually take multiple drugs, so pay attention to drug interactions when using antihistamines or other drugs that treat bee stings. Certain drugs may interact with the therapeutic drug that the patient is using, affecting efficacy or increasing the risk of adverse effects. Consulting a doctor: It is best to consult a doctor or pharmacist before using any new medication (e. g. pain medication, painkillers, antihistamines).3. Management of chronic diseases Cardiovascular diseases: Patients with heart disease, hypertension, or heart failure should pay special attention to cardiovascular symptoms, such as chest pain, palpitations, or abnormal blood pressure. Bee venom may trigger or aggravate these symptoms. Diabetes: Diabetic patients need to closely monitor their blood sugar levels, which may cause blood sugar fluctuations due to bee stings or treatment. Wound healing may also be slower, so special attention should be paid to the risk of infection. Respiratory diseases: Patients with asthma or chronic obstructive pulmonary disease (COPD) should be monitored closely, as bee stings may cause bronchospasm or aggravate dyspnea. Kidney disease: Patients with chronic kidney disease need monitoring for renal function, especially after multiple stings or severe reactions. Bee venom may cause acute kidney injury, requiring close monitoring of urine output and renal function indicators.4. Cold compress and pain relief cold compress: you can apply a cold compress bag or ice cubes wrapped in the cloth to cover the sting area, in order to reduce the pain and swelling. Avoid applying ice directly to your skin to prevent frostbite. Pain: The overall health of patients with chronic diseases should be considered when using painkillers (such as acetaminophen)Status, avoiding the use of drugs that may affect the liver and kidney function.5. Prevent infection Clean wounds: Use mild soap and water to clean the wound to prevent infection. Close observation: Patients with chronic diseases may have a weak immune system, so they should be closely observed at the wound for signs of infection (such as swelling, pus, fever, etc.). If signs of infection occur, seek medical attention immediately.6. Psychological support Patients with chronic diseases may be anxious or nervous about bee stings, especially for those with cardiovascular or respiratory diseases. Providing psychological support and comfort can help reduce stress and prevent disease aggravation.7. Timely medical attention systemic reaction: If the patient has any systemic symptoms (such as allergic reactions, chest pain, dyspnea, consciousness changes, etc.), seek medical attention immediately. Chronic disease management: Patients are advised to contact their primary care doctor after bee stings to discuss the need to adjust the treatment plan for the chronic disease, especially if there is a severe reaction or the use of emergency medication.8 Follow-up and long-term management Continue to monitor the health of patients, especially within days after bee stings. Some patients with chronic diseases may have a delayed response or fluctuations in their disease after their initial symptoms subside, so regular follow-up is very important. Patients with chronic disease need special attention after bee stings because their underlying health status may influence the response to bee stings. Timely monitoring, rational drug use, close observation of the changes of chronic disease symptoms and emergency treatment when necessary are necessary to ensure the safety and health of patients. | Immunocompromised patients (such as patients receiving immunosuppressive therapy, organ transplants, HIV infected patients, chemotherapy patients, etc.) need special care after bee stings because their immune system is less resistant to infection and inflammation and is more prone to complications. The following are the tips for immunocompromised bee stings: 1. Immediately remove the danger area away from the colony or nest to avoid further stings.2. Check and remove the sting If it is a bee sting, check if the sting remains in the skin. Gently wipe the sting with a fingernail, credit card edge, or blunt instrument to avoid squeezing the sac to reduce venom injection. Wash the wound thoroughly with gentle soap and water. This is particularly important for the immunocompromised, to reduce the risk of infection.4. Apply a cold compress bag or wrap ice in a clean cloth and apply it to the sting area for 10 to 20 minutes to relieve pain and swelling. Avoid applying ice cubes directly to your skin to prevent frostbite.5. Monitor closely for allergic reactions Immunocompromised people may respond more strongly to bee venom, so watch closely for signs of allergic reactions, such as dyspnea, swollen throat, generalized urticaria, dizziness, or syncope. If you have any systemic symptoms, seek medical attention immediately. If you have symptoms of anaphylactic shock, use an epinephrine autoinjector (e. g. Epipen) and seek medical attention as soon as possible.6. Prophylaxis and treatment of infections Antibiotic prophylaxis: Depending on the specific circumstances of immunocompromised individuals, doctors may recommend prophylactic antibiotics to prevent infection. If the wound shows any signs of infection (e. g., redness, pus, fever, etc.), antibiotic therapy should be initiated immediately. Regular observation: Closely observe the wound healing, and pay special attention to any signs of delayed healing or spread of infection.7. Use drugs with caution. Consider the interaction between immunosuppressive drugs and drugs for treating bee stings (such as antihistamines, pain killers). Before using any medication, consult a doctor or pharmacist to ensure safety.8. Management of underlying diseases The immunocompromised people usually have underlying diseases or are receiving special treatment. These underlying diseases or therapeutic devices need to be ensuredThe stroke was appropriately adjusted during the management of bee stings to avoid aggravation.9. Psychological support For immunocompromised health, they may panic or worry about bee stings. Providing psychological support and keeping calm can help to improve the response effect.10. Regular follow-up Within several days after stings, regular follow-up should be arranged to monitor disease progression and wound healing. If new symptoms or changes in your condition occur, contact your doctor immediately.11. Education and Prevention Education that immunocompromised people avoid environments that may cause bee stings, such as staying away from hives, wearing protective clothing while outdoors, and avoid using perfumes or cosmetics that may attract bees.12. Emergency plan If an immunocompromised person has a history of allergies or possible serious reactions, ensure that they carry an epinephrine autoinjector with them and tell their family members or caregivers how to use it in an emergency. The management of postbee stings in immunocompromised individuals requires special caution, with emphasis on preventing infection, monitoring allergic reactions and managing underlying diseases. Prompt medical intervention and continuous condition monitoring are essential to prevent serious complications. Family members and caregivers should also understand how to properly in an emergency to ensure safety of patients. |
|  | Medication Treatment | Medication Treatment | Medication Treatment | Medication Treatment | Medication Treatment |
| Question | What is the indication that uses antibiotic after bee sting? | What are the commonly used analgesic drugs after bee sting? | What is the basis of the choice of antihistamine drugs after a bee sting? | What is the principle of glucocorticoid use after bee sting? How to choose the dose? | How to undertake fill fluid treatment after bee sting? What are the precautions? |
| Answer Engine 4 （ChatGPT4.0） | No antibiotics are not routinely required after bee stings, because in most cases bee stings are sterile. However, in some specific cases, the use of antibiotics may be necessary to prevent or treat the infection. The following are the indications for the use of antibiotics after bee stings: 1. Signs of infection Local infection: Antibiotics are necessary if signs of infection occur on the site of bee stings, such as persistent redness, increased pain, fever, pus around the wound, and skin ulceration. Cellulitis: After bee stings, skin and subcutaneous tissue infection may cause cellulitis, manifested as swelling, tenderness, and fever, extended to the surrounding tissues. Systemic antibiotic therapy is required at this time.2. Prophylactic use of immunocompromised patients: For immunocompromised patients, such as immunosuppressive patients, organ transplant patients, or patients receiving chemotherapy, doctors may recommend prophylactic antibiotics due to a high risk of infection.3. High risk of infection in diabetic patients: Diabetic patients are prone to infection due to slow wound healing under hyperglycemia. Using antibiotics may be necessary if there are signs of infection or a high risk of infection.4. Risk of infection secondary to large area stings or multiple stings: When a patient is affected by multiple bee stings or stings, the skin barrier is severely damaged and the risk of secondary bacterial infection is increased. In this case, antibiotics may be required.5. Wound pollution Stings in special environments: If bee stings can pollute the environment (such as soil, sewage, etc.), or the patient tries to dispose of the wound itself, causing bacterial contamination, the use of antibiotics can prevent wound infection.6. History of recurrent infection: If a patient has a history of previous wound infection, especially after ststing, antibiotics may be required as prophylaxis.7. Persistent systemic symptoms fever and general malaise: Antibiotics are necessary if persistent fever, chills or other systemic symptoms after a bee sting, and systemic infection or sepsis is suspected. Selection of antibiotics The selection of antibiotics should be based on the type of infection, the patients past medical history, and the possible pathogen.frequently-usedAntibiotics may include: penicillin (e. g. amoxicillin): for common skin infections. Cephalins (e. g., cephalexin): for a broader spectrum of antimicrobial therapy. Clindamycin: Use in patients allergic to penicillin, or when an anaerobic bacterial infection is suspected. Erythromycin or other macrolides: as an alternative to patients with penicillin allergy. The decision to use antibiotics after bee stings should be based on clinical signs of infection, patient health status, and the risk of infection. Antibiotics are not a routine treatment for bee stings, but they can play an important role in certain situations, such as when symptoms of infection occur or when the patient is immunocompromised. Patients should use antibiotics under physician guidance to ensure the effectiveness and safety of the treatment. | Pain following a bee sting can often be effectively relieved by over-the-counter analgesic medication. The following are commonly used analgesic drugs: 1. Acetaminophen (Acetaminophen) brand name: Tylenol (Tylenol) Action: Acetaminophen is a commonly used analgesic, suitable for mild to moderate pain. It relieves pain by inhibiting prostaglandin synthesis in the brain, but is not anti-inflammatory. Suitable population: Acetaminophen is usually safe and suitable for most people, including pregnant women and patients with certain chronic diseases, but excessive use should be avoided to prevent liver damage.2. Ibuprofen (Ibuprofen) brand name: Advir (Advil), Merrill Lynch (Motrin) Effect: ibuprofen is a nonsteroidal anti-inflammatory drug (NSAID), which not only effectively relieves pain, but also has anti-inflammatory and antipyretic effects. It reduces the production of the inflammatory mediator prostaglandins by inhibiting the cyclooxygenase (COX) enzyme. Applicable population: for mild to moderate pain and accompanying inflammatory reactions such as redness and swelling. It is irritating to the gastrointestinal tract, take with meals and avoid long use. For patients with gastric ulcer, renal insufficiency or cardiovascular disease, doctors should be consulted before use.3. Naproxen (Naproxen) Brand name: Naproxen sodium (Aleve) Effect: Naproxen is also a non-steroidal anti-inflammatory drug in a similar mechanism to ibuprofen, but for a longer period and a longer interval between doses. Applicable population: suitable for relieving prolonged pain and inflammation. Similar to ibuprofen, attention is required to gastrointestinal side effects and potential effects on the kidney.4. Aspirin (Aspirin) effects: Aspirin is also a non-steroidal anti-inflammatory drug, with analgesic, anti-inflammatory and antipyretic effects. Reduce prostaglandin production by inhibiting the COX enzyme. Applicable population: It is used to relieve mild to moderate pain, but it is not recommended for children, adolescents, because it is more irritating to the gastrointestinal tract and may affect platelet function, or for patients with gastrointestinal diseases.5. Local analgesic lidocaine (Lidocaine): local anesthetic drugs, often used in the form of ointment or gel for local application, can anesthetize the skin in a short period of time, reduce pain. Benzocaine (Benzocaine): another local typeAnesthetic drugs, commonly used for mosquito bites and local pain relief after minor burns.6. Antihistamines Although antihistamines (such as diphenhydramine and loratadine) are mainly used to reduce allergic reactions, they can also reduce itching and swelling caused by bee stings to a certain extent, thereby indirectly relieving pain. Individual differences: different individuals have different responses to drugs, so the patients allergy history, underlying diseases and other medication conditions should be considered when choosing analgesic drugs. Avoid excess: Especially for acetaminophen, dose guidelines should be strictly observed to avoid liver injury. Consulting your doctor: For patients with chronic diseases, pregnant women, elderly patients, or chronic use of other drugs, consult your doctor before using analgesic drugs to avoid adverse drug interactions. Acetaminophen, ibuprofen, and naproxen are commonly used analgesic agents after bee stings to effectively relieve pain and inflammation. Topical pain medication can also be used to relieve local pain. When using these drugs, they should be selected according to the specific situation of the patient, and pay attention to the drug dosage and potential collateral work. | In the treatment after bee stings, antihistamines are mainly used to relieve allergic reactions caused by bee venom, such as itching, redness, and urticaria. When choosing the appropriate antihistamines, decide according to the specific patient, the characteristics of the drug and possible side effects. Antihistamines are usually divided into first-generation and second-generation antihistamines, which are similar in mechanism of action, but different in side effects and duration. First-generation antihistamine: Diphenhydramine (Diphenhydramine): a commonly used first-generation antihistamine that rapidly relieves itching and allergic symptoms. Chlorpheniramine (Chlorpheniramine): another first-generation antihistamine, often used in mild-to-moderate allergic reactions. Features: first-generation antihistamines usually act quickly, but easily cause side effects such as drowsiness and dry mouth, so they are more suitable for acute conditions that require rapid relief of symptoms, but may not be suitable for patients who need to stay awake for a long time. Second-generation antihistamine: loratadine (Loratadine): A commonly used second-generation antihistamine with few side effects and which does not usually cause lethargy. Cetirizine (Cetirizine): A faster second-generation antihistamine, effective in controlling pruritus and urticaria, but may cause mild drowsiness at high doses. Fexofenadine (Fexofenadine): minimal drowsiness and suitable for patients who need to stay awake. Characteristics: second generation antihistamines have a long time and fewer side effects, which are suitable for patients who need continuous control of symptoms.2. Acute and severity of symptoms Acute severe reactions: such as rapid development of severe itching, redness, urticaria, or systemic bee stings, allergic reactions, usually preferred first generation antihistamines, such as diphenhydramine, because they work faster and can relieve symptoms in a short period of time. Persistent mild-to-moderate response: Second-generation antihistamines (e. g., loratadine) may be more appropriate for mild symptoms rather than cilatadine, or if the patient needs to remain awake.3. Age: Children and the elderly may be more sensitive to the side effects of first-generation antihistamines and are usually recommendedSecond-generation antihistamines were chosen. Basic health: Patients with heart disease, glaucoma, prostatic prostate, asthma or chronic obstructive pulmonary disease (COPD) should be cautious about using first-generation antihistamines with anticholinergic side effects that may worsen the symptoms of these diseases. Occupational or daily activities: If a patient needs to drive, operate machines, or do work that requires high concentration, choose second-generation antihistamines that do not cause drowsiness.4. Drug interaction and medication: If the patient is taking other medications, such as sedatives, anxiolytic drugs, or alcohol, the sedative effect of first-generation antihistamines may be enhanced. Therefore, it is safer to choose second-generation antihistamines.5. Long-term use of drug tolerance: For patients who need to use antihistamines for a long time, if their symptoms last for several days or more, it is recommended to choose second-generation antihistamines that are better tolerated and do not cause sleepiness during the day.6. Specific clinical conditions during pregnancy and lactation: Special attention should be paid to the safety of drugs. Usually loratadine or cetirizine is a safe choice under the advice of a doctor, but according to the individual situation. In the selection of antihistamines after bee stings, comprehensive consideration should be based on the severity of symptoms, the individual patient, possible drug interactions, and daily activity needs. First-generation antihistamines are suitable for acute, severe symptom control, but may cause drowsiness; second-generation antihistamines are more suitable for mild or long-term symptom management with fewer side effects. Consultation is advised before use to ensure that the most appropriate drug prescription is selected. | The use of corticosteroids after bee stings is mainly used to manage severe allergic reactions, severe local inflammatory reactions, or to prevent further deterioration of allergic reactions. Glucocorticoids help to control symptoms and prevent complications by suppressing the immune response and reducing the release of inflammatory mediators. Here is the use of principles and dose selection guidelines: 1. Use principle indications: severe systemic allergic reaction: in anaphylactic shock or severe systemic allergic reaction (e. g., widespread urticaria, laryngeal edema, bronchospasm), glucocorticoid is usually combined with adrenaline and antihistamines, help relieve symptoms and prevent delayed reaction. Severe local inflammatory reaction: In patients with severe local swelling, significant pain, and large oedema, corticosteroids can help reduce local inflammation. Prevent delayed reaction: using glucocorticoids can prevent or reduce the possible delayed allergic reaction. Dose selection: adjusted by severity: Dose should be adjusted for symptom severity and the patients body weight. Usually, for acute severe reactions, short-term treatment with higher doses of glucocorticoids is recommended. Time of use: Short-term use: The use of corticosteroids is usually limited to the short-term (several days) to reduce the risk of long-term side effects. It is usually used in the acute phase for 1-3 days, and you will decide whether to gradually reduce the dose or stop the drug depending on the condition. Avoid long-term use: Since long-term use of glucocorticosteroids may cause a series of side effects (such as hyperglycemia, immunosuppression, osteoporosis, etc.), the time of use of glucocorticosteroids should be shortened after ststing. Individualized treatment: Medical history considerations: the patients medical history, such as diabetes mellitus, hypertension, infectious diseases, etc., should be considered when deciding on the use of glucocorticoids. If the patient has diabetes, the blood sugar should be closely monitored when using glucocorticoids. Adrenal function assessment: In patients with known adrenal insufficiency.2. Commonly used corticosteroids and dose prednisone (Prednisone): Oral administration: the common dose for adults is 40-60 mg / day, given in 1-2 times. Depending on the symptom improvement, it is usually gradual after 2-3 daysDose reduction, and drug withdrawal within 5-7 days. Children: adjust the dose according to body weight, usually 1-2 mg / kg / day, 1-2 times. Methylprednisolone (Methylprednisolone): Intravenous: for acute allergic reactions, 1-2 mg / kg in adults, once or in doses. Depending on the circumstances, the administration may be repeated after the initial dose. Oral administration: Similar dose and administration to prednisone, usually for subsequent treatment after more severe or systemic reactions. Dexamethasone (Dexamethasone): IV or orally: for acute response, usually at initial doses of 4-10 mg (adults), with repeated administration based on symptoms. Dexamethasone acts for a long time and is suitable for the short-term control of severe allergic reactions. Children: Dose was adjusted according to body weight, usually 0.1-0.2 mg / kg.3. Side effects monitoring Short-term use: Short-term use of glucocorticoids has fewer short-term side effects, but pay attention to possible hyperglycemia, hypertension and immunosuppression. Risk of long-term use: Long-term or high-dose use should be avoided, with tapering as required if necessary to prevent adrenal function inhibition and drug withdrawal response. The use of corticosteroids to manage severe allergic reactions and local severe inflammatory reactions after bee stings and their use should be based on the severity of symptoms and considering the individual patient condition and medical history. Short-term treatment is usually performed with higher doses, with the dose gradually reduced and withdrawn as symptoms resolve to avoid long-term side effects. When using corticosteroids, patients are closely monitored for response and side effects to critical levels. | The application of fluid rehydration therapy after bee stings is mainly used for the management of hypotension and impaired renal function due to anaphylactic shock, rhabdomyolysis or other complications. The objectives of rehydration therapy include maintenance of blood volume, correction of electrolyte imbalance, support of renal function and prevention of acute renal failure. The following are the guidelines and precautions for fluid rehydration treatment after bee stings: 1. Indications Anaphylactic shock: After bee stings, if the patient has severe systemic allergic reaction, hypotension, shock and other symptoms, fluid rehydration is a key part of emergency measures. Rhabdomyolysis: If bee stings cause rhabdomyolysis (soy-colored urine, high creatine kinase levels, etc.), rehydration can help prevent acute renal failure. Impaired renal function: In the early stages of renal impairment, fluid rehydration can help maintain renal perfusion and promote the excretion of toxins and metabolic waste.2. Selection of rehydration Saline (0.9% sodium chloride solution): preferred liquid: usually as the initial rehydration, suitable for blood volume supplementation in most cases. Applicable scenario: initial treatment and blood volume maintenance for anaphylactic shock. Lactate Ringers solution (Lactated Ringers Solution): Features: contains electrolytes (sodium, potassium, calcium, chlorine) and lactic acid, close to body fluids, can maintain acid-base balance. Application scenario: For patients who need massive rehydration and need to maintain electrolyte balance, such as rhabdomyolysis.5% Glucose solution (D5W): Use scenario: In some cases, a combined glucose solution may be required, especially in patients treated with combined hypoglycemia or requiring carbohydrate supplementation. But usually not as initial fluid but for maintenance or replenishment. Sodium bicarbonate solution: applicable scenario: for the treatment of acidosis caused by rhabdomyolysis. Alkalized urine also helps to reduce renal damage by myoglobin.3. rehydration volume and speed Initial rapid rehydration: When handling anaphylactic shock, rapid intravenous infusion of 1-2 liters of saline (adults) or rapid supplementation based on body weight (20 mL/kg for children) is recommended. Depending on the patients blood pressure and clinical response, you can repeat it if necessary. Maintain rehydration: The rate of rehydration should be based on the patients blood pressure, urine volume and electricityThe ute balance is adjusted. In patients with rhabdomyolysis, higher maintenance rehydration may be required to ensure that urine output is at least 200-300 mL per hour (adult). Individualized adjustment: The rehydration regimen should be individualized according to patient weight, age, severity and response.4. Note to monitor fluid balance: During the process of rehydration, the urine volume, blood pressure, heart rate, respiratory rate and body fluid status should be closely monitored to avoid excessive fluid (resulting in pulmonary edema) or insufficiency. Electrolyte monitoring: regularly test the electrolytes (sodium, potassium, calcium, etc.) and the acid-base balance in the blood, to avoid the electrolyte imbalance caused by fluid rehydration. Renal function assessment: Especially in patients with rhabdomyolysis, creatine kinase, blood urea nitrogen, serum creatinine and urine analysis should be closely monitored during rehydration to assess renal function and adjust treatment options. Avoid heart burden: For patients with cardiac insufficiency or renal insufficiency, the speed and amount of fluid replacement should be cautious to avoid excessive heart burden, leading to heart failure or pulmonary edema. Blood glucose monitoring: Blood glucose levels need to be monitored closely when using a glucose solution, especially in diabetic patients. Solution rehydration therapy is an important measure to manage anaphylactic shock, rhabdomyolysis and prevention of renal function injury after bee sting. Choosing the appropriate type, quantity and speed of rehydration and adjusting it according to the individual situation of patients can effectively improve the prognosis and reduce the occurrence of complications. Patients vital signs, electrolyte balance, and renal function should be closely monitored during fluid rehydration to ensure the safety and effectiveness of treatment. |
|  | Wound Treatment | Wound Treatment | Wound Treatment | Wound Treatment | Wound Treatment |
| Question | What are the characteristics of skin damage after bee sting and its clinical significance? | How to calculate the number of bee sting sting needles? | Do you need to remove the poison sting after a bee sting? | Is there a need for routine tetanus prevention after bee stings? | How does the cellulitis caused by bee sting handle? |
| Answer Engine 4 （ChatGPT4.0） | Skin damage following bee stings usually results from the direct action of bee venom and the subsequent triggered body immune response. The characteristics and severity of these reactions can vary from mild skin symptoms to severe systemic reactions, including: 1. erythema and swelling characteristics: erythema and swelling immediately after bee sting are the most common skin reactions. Significant red and swelling usually occurs in the injured area, sometimes with heat and tenderness. Clinical implications: This suggests that there is a local inflammatory response to toxins in bee venom (such as phospholipase A2 and bee venom peptide melittin) that activate local immune cells and inflammatory mediators.2. Pain characteristics: Bee stings are usually accompanied by significant pain, and the degree of pain may vary from person to person, ranging from mild tingling to severe pain. Clinical significance: pain is mainly caused by the toxins in bee venom directly acting on nerve endings and the inflammatory reaction. Pain is also an indicator of assessing the severity of the injury.3. Urticaria and pruritus characteristics: pruritus erythema or urticaria may occur in the stings or other body parts. Clinical implications: These symptoms indicate that the body has developed an allergic reaction to bee venom. Urticaria is due to the massive release of mediators such as histamine, which are released by immune cells such as mast cells after exposure to bee venom.4. Blicular characteristics: In a few cases, blisters may appear in the stings, indicating that the deep layer of the skin has been seriously damaged. Clinical implications: blister formation is often associated with more severe local reactions and may require closer observation and treatment to prevent infection or deeper tissue damage.5. Characteristics of skin necrosis: in rare cases, the stings site may appear necrosis, the skin is black, and the injury area dies. Clinical significance: Skin necrosis is a marker of severe local damage, which may cause severely blocked local blood circulation due to specific components in bee venom. This condition requires urgent medical intervention and may include surgical debridement.6. Characteristics of secondary infection: If the sting site is improperly handled or poorly cleaned, secondary infection may occur, manifested as increased swelling, pain, fever and pus discharge. Clinical significance: Secondary infection can aggravate the local damage, delay the healing process, and may occurInto a more serious health problem. Wounds should be managed promptly and treated with antibiotics if necessary. The characteristics of skin damage after bee sting and its clinical significance guide the measures of postinjury management and intervention. For all patients with bee stings, initial assessment and appropriate management are essential, and further medical evaluation and treatment are required for individuals exhibiting more severe symptoms. In dealing with bee stings, prevention of infection and monitoring of possible allergic reactions is heavy. | Identifying the number of stings (stings, i. e. the number of bee stings) is one of the important assessment factors, especially in the case of multiple stings. This is not only helpful to assess the potential toxin load, but also related to the choice of treatment options. Here are some methods and considerations for calculating the number of stings: 1. Direct observation sting inspection: For bee stings, because the bee loses its sting and eventually dies after the sting, it can be counted by checking for stings left on the skin. Check the sting site and search for the presence of stings and poison sac. Note: wasps or wasps do not lose their sting, so they can sting multiple times without leaving a sting. For this case, the calculation of the needle count relies more on the witness report or the patient feeling.2. Ask the witnesses or patient witness reports: If a sting is witnessed, ask the witnesses about the number of bees or wasps and the number of stings. Patient feedback: Ask the patient how they felt during the event, especially if they felt multiple stings or felt pain in different locations. The patient description can provide indirect information about the number of stings.3. Assess symptoms and signs of symptom severity: Multiple stings usually lead to more severe local or systemic reactions than a single sting. Assessment of the severity of symptoms (e. g., redness, pain, systemic reaction, etc.) can indirectly reflect the number of stings. Medical examination: Through medical examination, doctors can look for signs of multiple stings, including multiple local swelling points or areas of inflammation.4. Use of medical history information exposure: Know the patient at the time, such as whether they were up close to a hive or were accidentally stung by a bee. Exposure to colonies or near nests often implies a greater number of stings.5. Attention immediately: in the actual treatment of bee stings, even if the number of stings cannot be accurately determined, immediate measures should be taken to deal with stings, especially when there are multiple signs of stings. Monitoring and prevention of complications: independent of the number of stings, patients should be closely monitored for the development of symptoms, especially for possible allergic reactions or othersSerious complications, and timely intervention. Correct estimation of the number of stings may help the physician to assess the amount of toxin the patient may receive and potential treatment needs. In many cases, although accurate calculation cannot be made, a rough estimate can be obtained by the above method, so as to guide the subsequent medical line. | Yes, if the bee remains in the skin after a stings, it should be removed as soon as possible. Bees usually leave the sting and capsules in the wound when they sting, and the capsules continue to pump the venom into the wound, which can increase the severity of local symptoms and pain.1. How to properly remove the stinger: Do not squeeze the sac directly with your fingers or tweezers: This may lead to inject more toxins into the skin. Use a hard card or nail: use a credit card, cardboard, or nail to gently scrape along the skin surface to create the sting. This method may reduce the further injection of the toxin. Clean the wound: After removing the sting, the wound should be thoroughly cleaned with soap and water to reduce the risk of infection. Cold compress: Using an ice compress can help to reduce swelling and pain. Avoid scratching: Scratching wounds can aggravate the inflammatory response and increase the risk of infection.2. When special attention: If the sting is close to the eye, lips or other sensitive area, it is recommended to seek medical help as soon as possible. People allergic to bee venom should seek medical assistance immediately after the removal of the sting, as they may rapidly develop a systemic allergic reaction. Overall, it is important to remove the venom as soon as possible after a bee sting, which helps to reduce further absorption of the toxin and reduce the local response. Appropriate methods should be applied to avoid the pressure on the sac to prevent more toxin injection. If there is any serious reaction or uncertainty about how to safely remove the sting, professional medical help should be sought. | The need for tetanus vaccination after bee stings mainly depends largely on the patients vaccination history and the details of the stings. Tetanus is a serious infection caused by a toxin produced by Clostridium tetanus, which is usually found in soil, dust, and animal feces, and can enter the body through open wounds.1. Consideration of vaccination history: Whether adults and children receive the tetanus vaccine on time (usually part of the fifth or quadruple vaccine) is a key factor in assessing the need for additional vaccination. According to the Centers for Disease Control and Prevention (CDC) recommendations, adults should be vaccinated with a tetanus booster needle every 10 years. Stings condition: If a bee sting is caused through a contaminated object (e. g., after touching the wound with the dirty hand or touching the contaminated environment), this may increase the risk of tetanus infection. However, bee stings themselves do not directly increase the risk of tetanus because C. Tetanus is not commonly transmitted by a bee sting. Full vaccination is recommended and not more than 10 years: If a patients tetanus vaccination is the latest (i. e. it has been vaccinated in the past 10 years), vaccination for bee stings is usually not required. Unclear vaccination history or more than 10 years: If a patient is not sure of his vaccination or has been more than 10 years since the last vaccination, a tetanus booster dose is recommended. Dirty or severe wound: If the wound is particularly dirty or severe, doctors may recommend a tetanus booster shot to ensure safety, even if the inoculation was recorded in the last 10 years. Although bee stings themselves do not directly increase the risk of tetanus infection, it is wise to assess the need for tetanus booster needles given the possible contamination and vaccination status. It is always best practice to keep the wound clean and seek appropriate medical advice when managing any open wound. In case of doubt, or for questions regarding the prevention of tetanus after handling bee stings, consult a medical professional. | Cellulitis is a common skin infection associated with extensive redness, heat pain in the skin, and subcutaneous tissue, sometimes accompanied by fever and chills. After bee stings, it may develop into cellulitis if not managed properly. Here are the steps and recommendations for handling cellulitis: 1. Medical assessment Seek medical attention as early as possible: If a bee sting is suspected, seek medical help as soon as possible. Physicians usually make a diagnosis based on the appearance and symptoms of the skin, and may need further examination to rule out other potential causes.2. Drug therapy Oral antibiotics: The main treatment for cellulitis is the use of antibiotics. Physicians usually prescribe oral antibiotics, such as penicillin, cephalosporins, or other applicable antibiotics, depending on the patients allergy history and local antibiotic resistance status. Severe case management: For severe or rapidly expanding cellulitis, intravenous antibiotics may be required and monitored in hospital.3. Local care Cleaning and maintenance: It is very important to keep the wound area clean and dry. The wound can be gently cleaned with gentle soap and water. Cold compress: Using a cold compress can help to reduce inflammation and relieve pain. Clean cloth can be wrapped in ice and intermittently applied to the affected area. Elevate the affected limb: If cellulitis occurs in the arm or leg, raising the affected limb can help reduce swelling and pain.4. Pain management Use of over-the-counter pain medication: acetaminophen or nonsteroidal anti-inflammatory drugs (such as ibuprofen) can be used to control pain and reduce inflammation.5. Monitor symptom changes to observe symptoms: monitor whether the symptoms have improved, such as redness and pain, and whether the body temperature has returned to normal. Visit again when the symptoms worsen: If the symptoms do not improve within a few days or show signs of deterioration, such as persistent fever, swelling area expansion, pus, or feeling more uncomfortable, you should immediately return to the hospital for review.6. Preventive wound care: Care appropriately for all open wounds and avoid the use of any substances that may cause irritation. Avoid irritation: Avoid excessive use of the affected limb during wound healing and reduce pressure on the wound. Cellulitis is a serious infection that requires a prompt infectionOf medical intervention and appropriate care. Maintain good personal hygiene and follow the doctors instructions during the process. |
|  | Long-term Follow-up | Long-term Follow-up | Long-term Follow-up | Long-term Follow-up | Long-term Follow-up |
| Question | Which patients need long-term follow-up after discharge after bee stings? | What does the content of long-term follow-up after bee sting patient discharge have? | What does the health education content of the rehabilitation period after bee stings include? | How to follow-up and manage patients with secondary renal failure after bee sting? | How to undertake psychological guidance when bee sting injury patient leaves the hospital? |
| Answer Engine 4 （ChatGPT4.0） | Patients who require long-term follow-up after discharge often include individuals who experienced a severe response, had special health conditions, or potential complications. Long-term follow-up aims to ensure complete recovery of these patients, timely detection and management of possible follow-up problems. The following are several categories of patients to be considered for long-term follow-up: 1. Patients with severe or complex systemic anaphylactic shock or severe systemic anaphylactic reaction (e. g., extensive urticaria, laryngeal edema, severe dyspnea), should undergo long-term follow-up even if their symptoms are controlled in the short term. These patients may require allergen identification and evaluation, as well as possible immunotherapy or measures to avoid future exposure.2. Patients with chronic diseases or immunosuppression Patients with chronic diseases such as diabetes, cardiovascular disease, chronic kidney disease, or immune system disease may face a higher risk of complications after bee stings. Immunosuppressed patients (e. g. individuals receiving chemotherapy or immunosuppressive drugs) may be more sensitive to infection and require long-term monitoring and management.3 Patients who experience a severe local reaction If a patient experiences a severe local reaction after a bee sting, such as extensive swelling, persistent pain, or skin necrosis, it may require long-term monitoring of the healing process of the wound. This includes assessment of possible infection, wound healing, and further treatment that may be needed.4 Patients who require psychological support and who experience particularly painful or traumatic bee stings may need psychological support or psychotherapy to help them deal with fear or anxiety after the event. Follow-up can include assessment of the patients psychological status, providing or recommending mental health services if necessary.5 Multiple stings in patients with multiple stings may increase the risk of bee venom allergy. Such patients may need to undergo regular allergy testing and education on how to manage possible re-stings.6 If patients who fail to identify complete medical information fail to fully assess their medical history or possible risk factors at initial treatment, follow-up should be conducted to improve and assess their health status. Long-term follow-up after bee sting should be conducted for those individuals at high risk, especially thosePatients who experience severe reactions, have underlying health problems, or who may need further monitoring and treatment after initial treatment. With regular assessments and necessary interventions, potential health risks can be effectively managed to improve the quality of life of patients. | Long-term follow-up of bee stings patients after discharge is designed to ensure good wound recovery, to manage any potential long-term complications, and to assess and manage the allergic reactions associated with bee stings.1. Allergic status Assessment Allergic testing: For patients who have experienced severe allergic reactions, allergy testing may be required to confirm sensitivity to bee venom. Management plan: Based on the test results, physicians may recommend specific avoidance strategies or treatments, such as carrying an adrenaline autoinjector (such as Epipen) for possible future allergic reactions.2. Mental health support Mental state assessment: Considering that patients may experience anxiety or fear due to bee sting events, the patients mental health status should be assessed during the follow-up period. Psychological support services: If necessary, recommend psychological counseling or treatment to help patients deal with post-traumatic stress disorder (PTSD) or other psychological problems.3. Wound recovery monitoring wound examination: Monitor the wound healing process to ensure that there is no infection or other complications, such as necrosis or persistent inflammation. Wound care guidance: Provide appropriate knowledge of wound care to ensure that patients understand how to care for healing wounds.4. Monitoring drug efficacy and side effects Drug management: If patients need antibiotics, antihistamines or other drugs, monitor their efficacy and any potential side effects. Dose adjustment: Adjust the drug dose or treatment regimen based on the patients response and recovery.5. Function and activity to assess functional recovery: Assess the recovery of physical function, especially if bee sting affect the joints or movement. Rehabilitation advice: When necessary, provide physiotherapy or other rehabilitation services to help patients resume normal activities and quality of life.6. Long-term complications Monitoring chronic symptoms: monitor any possible chronic pain or persistent physical problems, such as pigmentation, scarring, or chronic pain. Follow-up schedule: Set an appropriate follow-up schedule according to the patient to ensure continuous monitoring and timely handling.7. Education and preventive measures: educate patients on how to avoid future bee stings, including notes during outdoor activitiesMatters and environmental management. Self-management education: Guide patients to identify early symptoms of allergic reactions and learn how to properly use emergency treatment drugs, such as the epinephrine auto-injector. Through these comprehensive follow-up measures, it can effectively support the recovery of bee sting patients, reduce the risk of further serious events, and improve the quality of life of patients. | The recovery period after bee stings is an important stage for patients, and appropriate health education can help them better manage their wounds, prevent complications, as well as avoid future bee stings. Here are some key health education items for the recovery period after bee stings: 1. Wound Care Clean Maintenance: Teach patients how to properly clean the wound, and gently clean the wound with gentle soap and water or doctor-recommended disinfectant to prevent infection. Observe symptoms: Teach the patient to identify signs of wound infection, such as increased swelling, persistent pain, increased exudate, fever, etc. If these symptoms occur, seek medical attention in time.2. Pain management Use of over-the-counter drugs: Guide patients on how to use over-the-counter pain medication (such as acetaminophen or non-steroidal anti-inflammatory drugs) to relieve pain. Cold compress application: Teach patients to use cold compress to reduce swelling and pain, especially in the first few days.3. Anaphylactic reaction management Understanding allergic symptoms: educate patients to identify possible allergic reaction symptoms, such as dyspnea, facial swelling, common rash, etc., and know when to seek emergency medical help. Emergency action plan: Provide an emergency action plan for patients with a history of severe allergies, including how to use an epinephrine autoinjector.4. Prevention of future bee stings environmental management: educate patients to avoid approaching the nests of bees or wasps, understand how to safely handle the surrounding environment, and reduce the bee attraction factors (such as floral fragrance, food residue, etc.). Personal protection: It is recommended to wear long-sleeved clothing and use tasteless personal care products outdoors, especially in seasons or areas where bees are active.5. Nutrition and Recovery Appropriate nutrition: Guide patients to maintain a balanced diet to support physical recovery, including adequate intake of protein, vitamins, and minerals. Be fully hydrated: Encourage patients to drink more water, especially if there is swelling or some painkillers are needed.6. Emotional and psychological support Emotional health: Bee stings may have an impact on patients emotional health, providing necessary support resources, such as psychological counseling, to help them deal with possible anxiety or fear.7. Regular review and follow-up: It is recommended that patients talk to the doctor regularly during recoveryThe treatment providers stay in touch to resolve possible problems in the rehabilitation process. With these health education content instructions, bee stings patients can more effectively manage their recovery process, reduce the risk of complications, and prevent future events of bee stings. These measures not only help improve patients self-care skills, but also enhance their awareness for potential health problems | Secondary renal failure after bee stings is a serious complication, usually with excessive release of myoglobin due to rhabdomyolysis (rhabdomyolysis), which affects renal function. Follow-up management of such patients requires particularly meticulous and systematic measures to monitor the progression and recovery of the condition and to prevent possible long-term complications. The following are the key steps for the follow-up management of patients with secondary renal failure after bee stings: 1. Close monitoring of renal function blood and urine tests: regular blood and urine tests, monitoring of renal function markers such as serum creatinine, blood urea nitrogen (BUN), and electrolyte levels (e. g., sodium and potassium). Evaluation of urine volume: Monitoring the patient for urine volume and urine nature, reduced urine volume may be an early sign of further deterioration of renal function.2. Manage kidney toxin load hydration therapy: Ensure that the patient is properly hydrated to help remove myoglobin and other toxins from the blood. The doctor may adjust the intake of intravenous or oral fluids, individually according to the patients renal function and fluid balance. Electrolyte balance: Monitor and adjust electrolyte levels, especially potassium and sodium, to prevent complications in the heart and other organs.3. Drug therapy Use Drugs: Use drugs as needed to control symptoms and manage complications, such as using diuretics to help promote urine production, or using drugs to adjust blood pressure and electrolyte balance.4. Avoid risk factors for kidney damage limiting drugs: Avoid the use of drugs that may further damage the kidneys, such as certain non-steroidal anti-inflammatory drugs (NSAIDs). Dietary management: Diet may need to be adjusted, such as limiting high potassium and phosphorus foods, ensuring adequate protein intake, and not paying excessive amounts.5. Long-term health monitoring regular tests: Even if the patient initially recovers from renal function, regular renal function tests are needed to monitor any long-term effects. Lifestyle adjustment: Guide the patient on the necessary lifestyle adjustment, including appropriate physical activity, healthy diet, and adequate rest.6. Psychological and social support for mental health: pay attention to the psychological state of patients, provide psychological support or counseling when necessary, to help them cope with the psychological burden related to symptoms. Education and support: support for patients and their familiesFor education on disease management, to help them understand the condition, treatment plan and the necessary precautions in daily life. Patients with secondary renal failure after bee stings require a comprehensive follow-up management, including regular renal function tests, appropriate drug therapy, lifestyle modification, and psychological and social support. Such management aims to maximize the recovery and maintenance of renal function, prevent complications, and improve the quality of life of patients. | Bee stings can have a significant psychological impact on patients, especially after experiencing severe allergic reactions or multiple stings. Psychological guidance at discharge is an important step in helping patients cope with possible anxiety, fear, or other psychological problems. Here are some effective psychological guidance strategies: 1. Assessment of psychological status is included in the discharge plan to determine if they have signs of anxiety, depression or post-traumatic stress disorder (PTSD). Ask patients about their feelings about bee stings and their treatment process, and focus on their concerns about similar situations in the future.2. Provide information and education on detailed information about bee stings, including possible physical reactions, how to handle an emergency, and measures to prevent repeated stings. Explain the common psychological reactions after bee stings and let patients know that their feelings are normal, not isolated.3 Develop coping strategies to teach patients basic stress management techniques, such as deep breathing, progressive muscle relaxation or meditation, to help them calm themselves when they feel anxious or panic. Patients are advised to practice these skills in their daily lives to enhance their ability to cope with future stressful situations.4 Develop an emergency action plan to help patients develop an explicit emergency action plan, including in what circumstances and how to use the epinephrine autoinjector, and when to seek medical help. Ensuring that patients and their families are aware and able to implement this program can reduce their uneasiness and improve their self-confidence.5 Support networks to encourage patients to share their experiences and feelings with family, friends or support groups to build a social network to support them. Provide relevant support group information to give patients the opportunity to communicate with others with similar experiences.6. Professional psychological support If patients show persistent psychological distress or have an impact on their daily functions, they should be recommended for professional psychological counseling or treatment. Provide locally available mental health resources, including contact information for psychotherapists and counseling services.7 Follow up schedule regular follow-up to assess psychological recovery and further needs. Delivery was continued during the follow-up periodPsychological support and the necessary intervention to ensure that patients are able to effectively manage their emotions and fears. Through these psychological guidance measures, it can help bee sting patients to better adapt to their daily life after discharge, manage their mental health, and reduce the possible psychological distress in the future. This is an integral part of the rehabilitation process and is crucial to the patients long-term recovery and quality of life. |
|  | Public Health and Prevention | Public Health and Prevention | Public Health and Prevention | Public Health and Prevention | Public Health and Prevention |
| Question | How to carry out public health education in areas with high incidence of wasp sting? | What are the high-incidence seasons of bee stings and the high-risk groups? | What content should the emergency treatment plan for mass bee stings include? | What are the environmental assessment and improvement measures after wasp stings? | Is it recommended for the removal and management of beehives in areas with a high incidence of bee stings? How to implement it? |
| Answer Engine 4 （ChatGPT4.0） | Public health education in areas with high prevalence of wasp stings is an important preventive measure designed to reduce stings events and improve the coping capacity of community members. Such educational activities should cover wasp identification, stinging prevention strategies, first aid measures, and when to seek medical help. The following steps for effective public health education: 1. Increase cognitive recognition education for wasps: Provide identification information about different types of wasps (such as Asian bumblebees, etc.), including their appearance, common habitats, and behavioral patterns. Ecological awareness: to educate the public to understand the role of wasps in the natural environment, and what kind of behaviors may cause wasps to attack.2. Popularization of precautions to avoid attracting wasps: Teach the public to avoid using perfume and wearing bright clothes, and not to throw away food scraps outside, which may attract wasps. Environmental management: Show residents how to safely manage the outdoor environment, such as regularly checking and removing areas that may become wasp nests. Protection measures: Educate the public to take personal protective measures when participating in outdoor activities, especially in grass, forest and other places where wasps may be encountered.3. Training of first aid skills Urgent action: Teach the public to take immediate actions after wasp stings, such as staying away from the stings, to avoid causing more wasp attacks. First aid measures: Provide detailed first aid steps, including how to safely remove stings, cold compress application, use of antihistamines, etc. Use of epinephrine: Provide training and education on the use of epinephrine autoinjectors for individuals with severe allergic reactions.4. Introduction of local medical resources Medical facility information: provide information on locally available medical resources, including emergency medical services, the location of hospitals and clinics. Emergency contact: Ensure that all community members know the phone number and ways to seek help in case of a bee sting emergency.5. Community participation and feedback: Organize community activities, such as lectures, seminars and field drills, so that residents can participate directly in the learning process. Feedback mechanism: Establish a feedback mechanism that allows community members to share their experiences and make suggestions for improvement to improve future education plans.6. Multilingual educational materials inclusive materials: Ensure that educational materials cover all community members, including multilingual versions, ensuring that non-native speakers also understand and receive educational content. These comprehensive educational strategies can effectively improve the communitys ability to respond to wasp threats, reduce stings, and ensure that the public can receive timely and effective help in such emergencies. | The high-incidence season of bee stings and high-risk groups are important public health information. Understanding these can help to take preventive measures to reduce stings. The following is detailed information about the high incidence season of bee stings and high-risk groups: 1. The high incidence season of bee stings is usually closely related to the climate and the activity cycle of insects such as bees and wasps. Specifically: Late spring to early autumn: This is the time when bees and wasps are most active, with temperatures warm, flowers in full bloom, and insects come out to find food and build nests. Summer: Especially in warm and humid climates, summer is the peak of insect activity, when bee stings are particularly frequent.2. High-risk groups Some people are more vulnerable to bee stings due to their specific activity habits or occupational characteristics: outdoor workers: farmers, gardeners, construction workers, forestry and park managers, etc., these people are more exposed to bees or wasp bees because they work outdoors for a long time. Outdoor activists: Hikers, campers, athletes, and other people who often operate in the wild, especially in the untapped natural environment. Children: They may not pay attention to the presence of hives when playing outdoors, or cause swarm attacks due to curiosity. People with a history of bee allergy: People with allergies to bee venom can rapidly develop severe systemic allergic reactions, especially at high risk. Residents living in active areas of honeybees: People living close to hives or insect habitats have an increased risk of stings due to increased range and density of bees.3. Preventive measures are particularly important for these high-risk groups and during the peak season, the following precautions: Wear protective clothing: Wear long-sleeved clothing, trousers and hats for bees or wasps. Avoid perfumes or perfume products: These products may attract bees or wasps. Be careful with food and drink management: cover your food and drinks when eating outdoors to avoid attracting insects. Learn about first aid: Learn how to deal with bee stings and use adrenaline autoinjectors, especially for people with allergies. Monitor childrens activities: ensure that children are outdoorsAvoid approaching possible hives or insect colonies. Reducing the risk and severity of bee stings can be significantly achieved by understanding the high-incidence season and high-risk groups, and by taking corresponding preventive and protective measures. | Group bee stings require a detailed set of emergency contingency plans to ensure a rapid and effective response and injury mitigation. Such plans should be detailed in response processes, resource allocation, personnel training and post-assessment. The following are the key elements of building an emergency treatment plan for mass bee stings: 1. Risk assessment and prevention Identify high risk areas: identify and mark those sites that become high risk areas because bees are active. Public education: to raise public awareness of the risk of bee sting through educational activities, and to teach ways to avoid bee sting and basic self-protection measures.2. Emergency response plan Establish communication chain: ensure that there is a clear communication system, when the relevant personnel (such as emergency personnel, medical institutions, security personnel) can be quickly notified. Rapid evacuation plan: Develop an effective on-site evacuation program to quickly move victims from dangerous areas to safe areas. On-site medical support: configure emergency equipment such as first aid kit and adrenaline autoinjectors and ensure trained medical or emergency personnel on site.3. Resource allocation Medical resources: Ensure that sufficient medical resources are available to handle mass bee sting injuries, including antihistamines, pain management drugs, epinephrine, and other emergency medical supplies that may be needed. Transportation resources: Prepare appropriate transportation to quickly transport severely injured patients to the hospital when needed.4. Training and drill Training plan: Conduct regular training for all personnel involved (including first aid personnel, safety personnel, teaching staff, etc.), to let them understand the emergency treatment procedures of bee stings and the correct way to use the treatment equipment. Regular drills: Regular simulation exercises are conducted to test the effectiveness of the emergency response plan and the coordination ability of all parties.5. Post-hoc treatment and psychological support follow-up: All injured people are followed-up to ensure that they receive the necessary medical and psychological support. Mental health support: Provide mental health support to victims to help them deal with possible psychological trauma.6. Evaluation and improvement of event analysis: After the end of the event, a comprehensive assessment of the implementation of the response plan should be conducted. Improvement plan: based on the evaluationRESULTS Adjust and improve contingency plans to improve the efficiency and effectiveness of the future response to similar events. Through these comprehensive measures, we can ensure that in group bee stings, it can be handled in an orderly and effective manner to minimize the injury and provide a comprehensive branch for the affected people. | Environmental assessment and improvement measures after wasp stings are designed to reduce the risk of future stings, and to protect the safety of people, here are some key measures: 1. Identify the location of wasp nests, especially near houses or in frequently active areas.2. Remove removal: hire professional pest control services to remove the nest safely. Do not try to remove the nest by yourself, as the wrong treatment may irritate the wasps and increase the risk of being attacked.3. Improve the environment: Remove elements of the environment that may attract wasps, such as open food, trash cans and pet food. Keep orchards and gardens well maintained and avoid excessive fruit accumulation or flower blooms, which may attract wasps.4. Use an insect repellent or trap device: use non-lethal traps or insect repellent in areas where wasps occur frequently to reduce their number. Install wasp traps, especially in outdoor activity areas.5. Strengthen personal protection measures: wear long-sleeved clothing and trousers for outdoor activities to reduce the exposed skin area. Use PPE such as hats and gloves, especially when handling gardens or orchards.6. Regular monitoring and education: regularly check for recurrent wasp nests around the house. Educate family members and staff to identify the species of wasps and take precautions. Through these measures, the threat and possible future stings can be effectively reduced. | In areas with high incidence of stings, it is necessary to recommend removal and management of nests to reduce the risk of sting and protect peoples safety. Here are some recommended steps on how to implement honeycomb removal and management: 1. Assess and identify first, determine the exact location of the honeycomb and the species of the bee. Different species of bees (e. g. wasps, wasps, bees) may require different treatment methods. To assess the honeycomb size, activity and their specific threats to the surrounding environment.2. Professional removal Contact professional pest control services for honeycomb removal. The professional team has the appropriate tools, techniques, and safeguards to do the work safely and effectively. The optimal time for honeycomb removal is usually at dawn or dusk, when the bees are less active.3. Use proper protective equipment to ensure that all operators wear proper protective clothing, including heavy clothing, gloves, hats, masks, and glasses. Using a smosmoker or other non-aggressive methods to calm the bees and make the removal process more peaceful. After removing the honeycomb, measures should be taken to prevent bees from building nests again in the same place. This may include patching all openings and removing environmental factors that attract bee species. Certain natural or chemical bee repellents can be sprayed around the site to reduce the return of bees.5. Monitor and maintain the processed areas to ensure that no new honeycomb are formed. Continuing to educate and remind community residents about how to identify and avoid bee risk.6 The environmentally friendly approach considers the ecological balance and prioritizes the migration of honeybee nests to a suitable natural environment rather than direct destruction. Work with local conservation agencies or bee breeders to seek the possibility of moving bees safely. By implementing the above steps, the hive problem can be effectively managed while reducing the negative impact on the environment and honey bee populations as much as possible. |
