# Supplementary material for: Clinical Management of Wasp Stings Using Large Language Models: Cross-Sectional Evaluation Study
Source: J Med Internet Res. 2025 Jun 4;27:e67489. doi: 10.2196/67489 (PMC12177424; doi:10.2196/67489)
Supplement: Multimedia Appendix 5 [file jmir_v27i1e67489_app5.docx]

| **Table 1** Scores for accuracy for each engine in each category | | | | | | | | | | | | | | | | | | |  |
| --- | --- | --- | --- | --- | --- | --- | --- | --- | --- | --- | --- | --- | --- | --- | --- | --- | --- | --- | --- |
|  | **ERNIE Bot 3.5**  **（N=8）** | | **ERNIE Bot 4.0**  **（N=8）** | | ***P value*** | | **Claude Pro**  **（N=8)** | | **ChatGPT4.0**  **(N=8)** | | ***P value*** | | **ERNIE Bot 3.5**  **（N=8）** | | **Claude Pro**  **（N=8)** | | ***P value*** | |  |
| Basic Knowledge |  | |  | |  | |  | |  | |  | |  | |  | |  | |  |
| Mean（SD） | 21.625（1.923） | | 20.0（1.195） | | 0.041 | | 20.875（3.182） | | 19.125（1.356） | | 0.078 | | 21.625（1.923） | | 20.875（3.182） | | 0.019 | |  |
| Median（Min，Max） | 21.5（19，24） | | 19.5（19，22） | |  | | 20.5（15，25） | | 19.5（17，21） | |  | | 21.5（19，24） | | 20.5（15，25） | |  | |  |
| Early Management |  | |  | |  | |  | |  | |  | |  | |  | |  | |  |
| Mean（SD） | 17.875（1.808） | | 16.875（1.642） | | 0.121 | | 21.75（3.495） | | 19.25（2.55） | | 0.020 | | 17.875（1.808） | | 21.75（3.495） | | 0.071 | |  |
| Median（Min，Max） | 18.0（15，20） | | 17.0（15，20） | |  | | 22.0（15，25） | | 20.0（14，23） | |  | | 18.0（15，20） | | 22.0（15，25） | |  | |  |
| Allergy Management |  | |  | |  | |  | |  | |  | |  | |  | |  | |  |
| Mean（SD） | 19.0（1.69） | | 17.875（1.959） | | 0.129 | | 23.25（1.832） | | 19.25（2.375） | | 0.011 | | 19.0（1.69） | | 23.25（1.832） | | 0.733 | |  |
| Median（Min，Max） | 18.5（17，22） | | 18.0（14，21） | |  | | 24.0（20，25） | | 18.5（17，24） | |  | | 18.5（17，22） | | 24.0（20，25） | |  | |  |
| Complication Management |  | |  | |  | |  | |  | |  | |  | |  | |  | |  |
| Mean（SD） | 18.875（1.458） | | 18.125（1.642） | | 0.161 | | 24.0（1.927） | | 18.375（1.598） | | 0.011 | | 18.875（1.458） | | 24.0（1.927） | | 0.336 | |  |
| Median（Min，Max） | 19.0（17，21） | | 18.5（16，20） | |  | | 25（20，25） | | 19.0（16，20） | |  | | 19.0（17，21） | | 25（20，25） | |  | |  |
| Severity Assessment |  | |  | |  | |  | |  | |  | |  | |  | |  | |  |
| Mean（SD） | 19.0（1.512） | | 16.75（1.832） | | 0.017 | | 23.75（1.753） | | 20.375（1.598） | | 0.018 | | 19.0（1.512） | | 23.75（1.753） | | 0.031 | |  |
| Median（Min，Max） | 18.0（18，22） | | 16.0（15，20） | |  | | 24.5（20，25） | | 20.0（19，24） | |  | | 18.0（18，22） | | 24.5（20，25） | |  | |  |
| Special Population Management |  | |  | |  | |  | |  | |  | |  | |  | |  | |  |
| Mean（SD） | 17.875（1.458） | | 16.75（1.909） | | 0.084 | | 22.875（2.357） | | 20.0（1.927） | | 0.026 | | 17.875（1.458） | | 22.875（2.357） | | 0.017 | |  |
| Median（Min，Max） | 18.0（16，20） | | 16.5（14，20） | |  | | 23.5（20，25） | | 20.0（17，24） | |  | | 18.0（16，20） | | 23.5（20，25） | |  | |  |
| Pharmacological Treatment |  | |  | |  | |  | |  | |  | |  | |  | |  | |  |
| Mean（SD） | 18.625（1.768） | | 17.375（2.134） | | 0.109 | | 22.875（2.295） | | 20.5（1.927） | | 0.056 | | 18.625（1.768） | | 22.875（2.295） | | 0.043 | |  |
| Median（Min，Max） | 19.0（15，20） | | 18.0（15，20） | |  | | 23.0（19，25） | | 20.0（19，25） | |  | | 19.0（15，20） | | 23.0（19，25） | |  | |  |
| Wound Management |  | |  | |  | |  | |  | |  | |  | |  | |  | |  |
| Mean（SD） | 17.625（2.134） | | 16.5（2.878） | | 0.066 | | 22.375（1.923） | | 20.125（1.808） | | 0.019 | | 17.625（2.134） | | 22.375（1.923） | | 0.027 | |  |
| Median（Min，Max） | 17.0（15，21） | | 15.5（12，20） | |  | | 22.5（20，25） | | 20.0（18，23） | |  | | 17.0（15，21） | | 22.5（20，25） | |  | |  |
| Long-term Follow-up |  | |  | |  | |  | |  | |  | |  | |  | |  | |  |
| Mean（SD） | 19.875（2.357） | | 18.5（2.07） | | 0.084 | | 23.125（1.642） | | 20.625（1.408） | | 0.017 | | 19.875（2.357） | | 23.125（1.642） | | 0.461 | |  |
| Median（Min，Max） | 20.0（15，23） | | 19.0（16，22） | |  | | 23.0（20，25） | | 20.0（20，24） | |  | | 20.0（15，23） | | 23.0（20，25） | |  | |  |
| Public Health and Prevention |  | |  | |  | |  | |  | |  | |  | |  | |  | |  |
| Mean（SD） | 19.75（2.188） | | 17.875（2.532） | | 0.026 | | 24.0（1.69） | | 20.0（2.204） | | 0.017 | | 19.75（2.188） | | 24.0（1.69） | | 0.891 | |  |
| Median（Min，Max） | 20.0（15，23） | | 18.0（15，22） | |  | | 24.5（20，25） | | 20.0（18，25） | |  | | 20.0（15，23） | | 24.5（20，25） | |  | |  |
| **Table 2** Scores for completeness for each engine in each category | | | | | | | | | | | | | | | | | | | |
|  | | **ERNIE Bot 3.5**  **（N=8）** | | **ERNIE Bot 4.0**  **（N=8）** | | ***P value*** | | **Claude Pro**  **（N=8)** | | **ChatGPT4.0**  **(N=8)** | | ***P value*** | | **ERNIE Bot 3.5（N=8）** | | **Claude Pro**  **（N=8)** | | ***P value*** | |
| Basic Knowledge | |  | |  | |  | |  | |  | |  | |  | |  | |  | |
| Mean（SD） | | 21.875（2.642） | | 19.0（1.414） | | 0.011 | | 21.125（2.696） | | 18.625（1.847） | | 0.018 | | 21.875（2.642） | | 21.125（2.696） | | 0.011 | |
| Median（Min，Max） | | 22.0（17，25） | | 19.0（16，21） | |  | | 21.0（18，25） | | 19.0（16，21） | |  | | 22.0（17，25） | | 21.0（18，25） | |  | |
| Early Management | |  | |  | |  | |  | |  | |  | |  | |  | |  | |
| Mean（SD） | | 18.75（1.165） | | 16.375（1.302） | | 0.027 | | 22.625（2.774） | | 19.0（1.927） | | 0.012 | | 18.75（1.165） | | 22.625（2.774） | | 0.915 | |
| Median（Min，Max） | | 18.5（17，20） | | 17.0（14，18） | |  | | 23.5（18，25） | | 19.5（16，22） | |  | | 18.5（17，20） | | 23.5（18，25） | |  | |
| Allergy Management | |  | |  | |  | |  | |  | |  | |  | |  | |  | |
| Mean（SD） | | 19.125（2.167） | | 17.75（2.053） | | 0.024 | | 23.5（1.604） | | 19.125（2.532） | | 0.012 | | 19.125（2.167） | | 23.5（1.604） | | 1 | |
| Median（Min，Max） | | 19.5（15，22） | | 18.0（15，21） | |  | | 24.0（20，25） | | 18.5（16，24） | |  | | 19.5（15，22） | | 24.0（20，25） | |  | |
| Complication Management | |  | |  | |  | |  | |  | |  | |  | |  | |  | |
| Mean（SD） | | 18.875（1.458） | | 17.5（2.00） | | 0.088 | | 24.125（1.808） | | 17.5（1.927） | | 0.011 | | 18.875（1.458） | | 24.125（1.808） | | 0.112 | |
| Median（Min，Max） | | 19.0（17，21） | | 17.0（15，20） | |  | | 25.0（20，25） | | 18.0（15，20） | |  | | 19.0（17，21） | | 25.0（20，25） | |  | |
| Severity Assessment | |  | |  | |  | |  | |  | |  | |  | |  | |  | |
| Mean（SD） | | 18.75（1.753） | | 16.5（2.00） | | 0.017 | | 24.0（1.69） | | 20.125（1.959） | | 0.011 | | 18.75（1.753） | | 24.0（1.69） | | 0.026 | |
| Median（Min，Max） | | 18.5（16，22） | | 15.5（15，20） | |  | | 24.5（20，25） | | 20.0（16，23） | |  | | 18.5（16，22） | | 24.5（20，25） | |  | |
| Special Population Management | | | | | | | | | | | | | | | | | | | |
| Mean（SD） | | 17.75（1.669） | | 16.625（1.847） | | 0.066 | | 23.625（2.066） | | 19.625（2.669） | | 0.011 | | 17.75（1.669） | | 23.625（2.066） | | 0.027 | |
| Median（Min，Max） | | 18.0（15，20） | | 16.0（15，20） | |  | | 25.0（20，25） | | 20.0（15，24） | |  | | 18.0（15，20） | | 25.0（20，25） | |  | |
| Pharmacological Treatment | |  | |  | |  | |  | |  | |  | |  | |  | |  | |
| Mean（SD） | | 17.625（2.326） | | 17.0（2.268） | | 0.317 | | 23.875（1.808） | | 20.375（2.20） | | 0.018 | | 17.625（2.326） | | 23.875（1.808） | | 0.026 | |
| Median（Min，Max） | | 18.0（15，20） | | 16.5（15，20） | |  | | 25.0（20，25） | | 20.0（17，25） | |  | | 18.0（15，20） | | 25.0（20，25） | |  | |
| Wound Management | |  | |  | |  | |  | |  | |  | |  | |  | |  | |
| Mean（SD） | | 17.625（2.264） | | 16.625（2.264） | | 0.066 | | 22.5（2.33） | | 19.625（1.768） | | 0.017 | | 17.625（2.264） | | 22.5（2.33） | | 0.043 | |
| Median（Min，Max） | | 17.5（15，21） | | 15.0（15，20） | |  | | 23.5（18，25） | | 20.0（16，22） | |  | | 17.5（15，21） | | 23.5（18，25） | |  | |
| Long-term Follow-up | |  | |  | |  | |  | |  | |  | |  | |  | |  | |
| Mean（SD） | | 19.625（2.446） | | 17.5（2.563） | | 0.043 | | 23.25（2.435） | | 21.0（1.852） | | 0.018 | | 19.625（2.446） | | 23.25（2.435） | | 0.131 | |
| Median（Min，Max） | | 20.0（15，23） | | 16.5（15，22） | |  | | 24.0（18，25） | | 21.0（18，24） | |  | | 20.0（15，23） | | 24.0（18，25） | |  | |
| Public Health and Prevention | |  | |  | |  | |  | |  | |  | |  | |  | |  | |
| Mean（SD） | | 19.5（2.138） | | 16.875（2.696） | | 0.018 | | 24.25（1.753） | | 19.25（2.964） | | 0.017 | | 19.5（2.138） | | 24.25（1.753） | | 0.609 | |
| Median（Min，Max） | | 20.0（16，23） | | 15.5（15，22） | |  | | 25.0（20，25） | | 19.0（15，25） | |  | | 20.0（16，23） | | 25.0（20，25） | |  | |
